# Enantioselective nickel-catalyzed arylative and alkenylative intramolecular 1,2-allylations of tethered allene–ketones†

**DOI:** 10.1039/c9sc05246a

**Published:** 2020-01-21

**Authors:** Riccardo Di Sanza, Thi Le Nhon Nguyen, Naeem Iqbal, Stephen P. Argent, William Lewis, Hon Wai Lam

**Affiliations:** The Glaxo Smith Kline Carbon Neutral Laboratories for Sustainable Chemistry, University of Nottingham Jubilee Campus, Triumph Road Nottingham NG7 2TU UK hon.lam@nottingham.ac.uk; School of Chemistry, University of Nottingham University Park Nottingham NG7 2RD UK

## Abstract

The enantioselective nickel-catalyzed reaction of tethered allene–ketones with (hetero)arylboronic acids or potassium vinyltrifluoroborate is described. Carbonickelation of the allene gives allylnickel species, which undergo cyclization by 1,2-allylation to produce chiral tertiary-alcohol-containing aza- and carbocycles in high diastereo- and enantioselectivities.

## Abstract 2

Chiral tertiary-alcohol-containing aza- and carbocycles are produced in high diastereo- and enantioselectivities by the nickel-catalyzed reaction of tethered allene–ketones with (hetero)arylboronic acids or potassium vinyltrifluoroborate.

## Graphical abstract

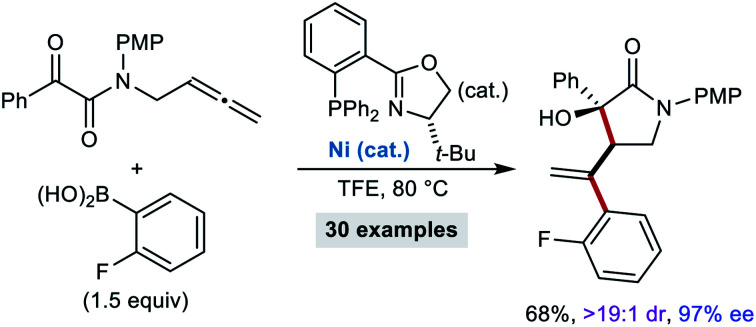

Pyrrolidin-2-ones with a tertiary alcohol at C3 are common structures in natural products such as pramanicin,^1^ norsecurinamine A,^2^ and cytochalasin Z_10_,^3^ and also appear in compounds with herbicidal activity^4^ (Fig. 1). Therefore, new methods for the preparation of these chiral structures in enantiomerically enriched form are valuable. Although numerous catalytic enantioselective reactions to give the benzannulated derivatives of these compounds (oxindoles with a C3 tertiary alcohol) have been described,^5,6^ it is surprising that, to our knowledge, methods for the corresponding pyrrolidin-2-ones are currently limited to palladium-catalyzed hydroxylations of cyclic β-ketoesters,^7*a*^ chromium-catalyzed cyclizations of enamides onto ketones,^7*b*^ and iridium-catalyzed (3 + 2) annulations of α-ketoamides with 1,3-dienes.^7*c*^

**Fig. 1:**
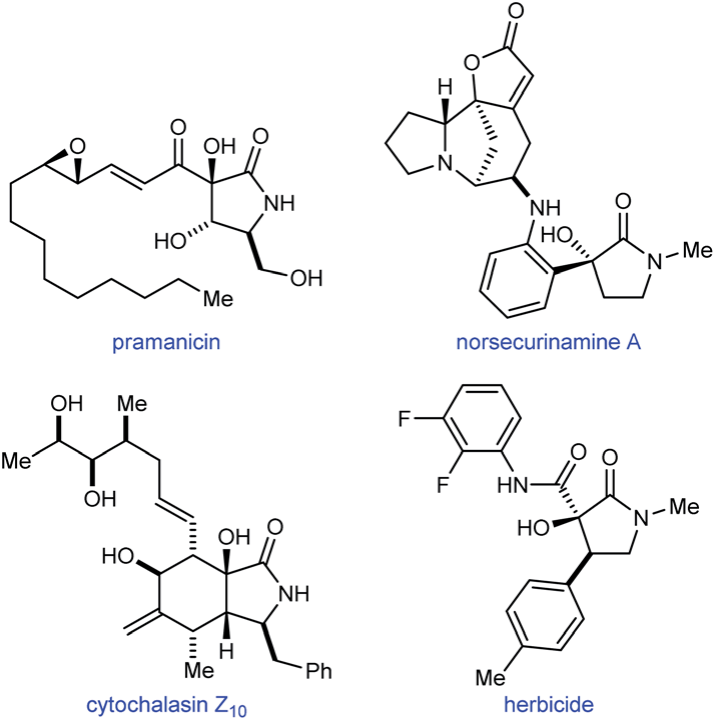
The occurrence of pyrrolidin-2-ones with a C3-tertiary alcohol in natural products and herbicidal compounds.

On the basis of our recent work in nickel-catalyzed arylative cyclizations involving allenes as substrates (Schemes 1A and 1B),^8^ it occurred to us that chiral pyrrolidin-2-ones with a C3 tertiary alcohol might be prepared by the nickel-catalyzed reaction of tethered allene– α-ketoamides **1** with arylboronic acids (Scheme 1C).^9,10^ Nickel-catalyzed addition of an arylboronic acid to the allene would give an intermediate allylnickel species,^8^ which could exist as several interconverting σ- and π-allyl isomers (representative structures **A** and **A′** are shown), which could then engage in enantio- and diastereoselective nucleophilic allylation^11^ of the ketone to give pyrrolidin-2-one **2**.^12–14^ While enantioselective metal-catalyzed nucleophilic allylations of carbonyl compounds are well-known,^15^ the closest approach to that shown in Scheme 1C is work described by groups of Tsukamoto^16*a*^ and Lu^16*b*^ to give other types of hetero- and carbocycles. However, their work used palladium catalysis and mainly tethered allene-aldehydes as the substrates and the single example of arylative cyclization of a tethered allene–ketone was only modestly enantioselective.^16*a*^ These results are perhaps unsurprising given that ketones are significantly less reactive than aldehydes, and the smaller steric differences between the two substituents flanking the carbonyl group in ketones compared with aldehydes means that achieving high enantioselectivities in nucleophilic additions is usually more challenging. Accordingly, solving this issue through the development of enantioselective arylative cyclizations of tethered allene–ketones using cheaper nickel catalysts to give products with tertiary alcohols is a worthwhile objective.

**Scheme 1:**
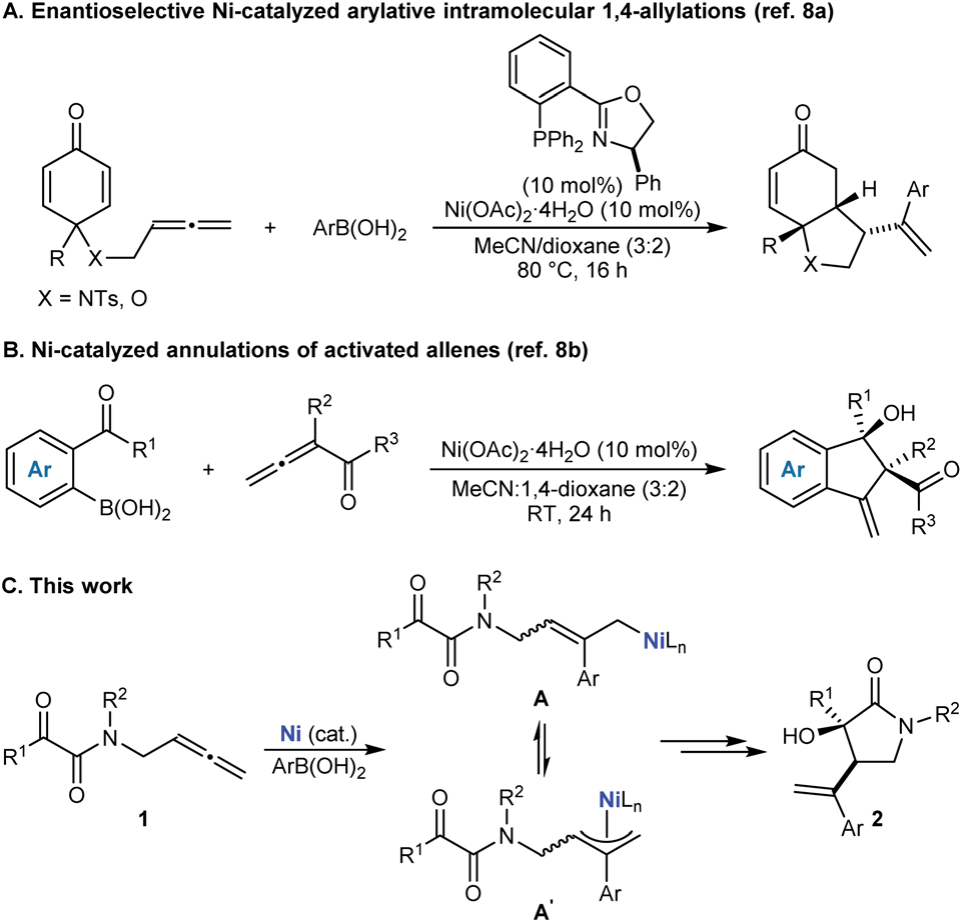
Nickel-catalyzed arylative cyclizations of allenes.

This study was initiated with the reaction of tethered allene– α-ketoamide **1a**, PhB(OH)_2_ (1.5 equiv.), and Ni(OAc)_2_·4H_2_O (5 mol%) in MeCN/1,4-dioxane (3 : 2) at 80 °C for 24 h, which gave racemic **2a** in 32% yield as determined by ^1^H NMR analysis, and as one observable diastereomer (Table 1, entry 1). Repeating this reaction with the addition of 5 mol% of the *P*,*N*-ligand **L1** increased the yield of **2a** to 77% (entry 2). The chiral phosphinooxazoline (*S*)-i-Pr-PHOX (**L2**) gave **2a** in 55% yield and 90% ee (entry 3).^17^ Improved results were obtained with (*S*)-*t*-Bu-PHOX (**L3**) (78% yield and 98% ee, entry 4), while (*R*)-Ph-PHOX (**L4**) gave results similar to **L2** (entry 5, compare with entry 3). With **L3** as the ligand, changing the mixed solvent system to MeCN or 1,4-dioxane alone offered noimprovement (entries 6 and 7). Finally, we tested TFE (2,2,2-trifluoroethanol) as the solvent,^9*b*,*c*^ which gave **2a** in almost quantitative yield after filtration of the crude reaction mixture through plug of a silica gel, with only a slight decrease in enantioselectivity (entry 8). On the basis of these results, the conditions of entry 8 were selected for subsequent experiments.

**Table 1:** Evaluation of reaction conditions^a^

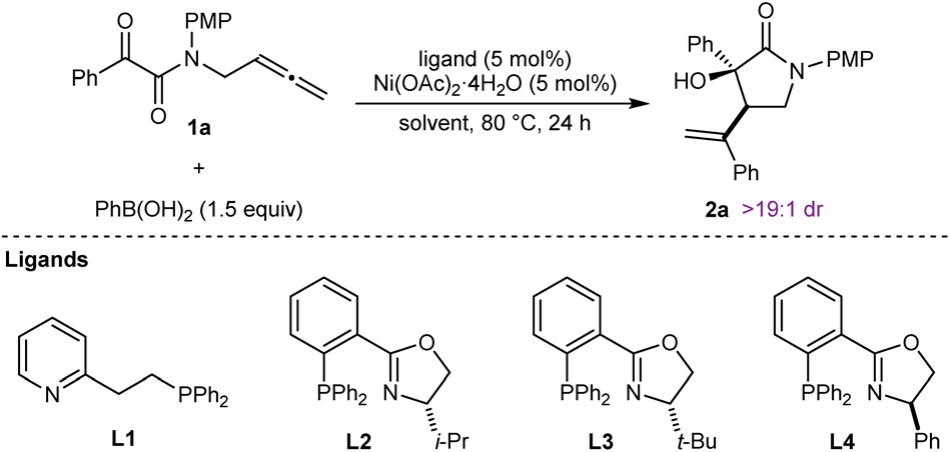				
Entry	Ligand	Solvent(s)	Yield^b^ (%)	ee^c^ (%)
1	—	MeCN/1,4-dioxane (3 : 2)	32	—
2	**L1**	MeCN/1,4-dioxane (3 : 2)	77	—
3	**L2**	MeCN/1,4-dioxane (3 : 2)	55	90
4	**L3**	MeCN/1,4-dioxane (3 : 2)	78	98
5	**L4**	MeCN/1,4-dioxane (3 : 2)	61	−91^d^
6	**L3**	MeCN	78	98
7	**L3**	1,4-Dioxane	59	98
8	**L3**	TFE	>99	96

a Reactions were conducted using 0.10 mmol of **1a**.

b Determined by ^1^H NMR analysis of the crude reactions using 1,3,5-dimethoxybenzene as an internal standard.

c Determined by HPLC analysis on a chiral stationary phase.

d The major product was the enantiomer of **2a**. PMP = *para*-methoxyphenyl.

The scope of this process in the enantioselective synthesis of chiral pyrrolidin-2-ones was then explored (Table 2). Pleasingly, various tethered allene–α-ketoamides **1a–1f** reacted successfully with a range of (hetero)arylboronic acids to give pyrrolidin-2-ones **2a–2q** as single observable diastereomers (with only a single exception) in up to >99% ee. Notably, with TFE as the solvent, the products were in many cases obtained in essentially quantitative yields after filtration of the crude reaction mixtures through silica gel, and purification by column chromatography was often not required. Regarding the ketone substituent of the substrate, phenyl (**2a** and **2g–2q**), 2-furyl (**2b**), simple alkyl (**2c** and **2d**), and branched alkyl (**2e**) groups are tolerated. Changing the nitrogen substituent to a benzyl group was possible, though the product **2f** was obtained in a lower 87% ee. However, replacing TFE with MeCN as the solvent gave **2f** in >99% ee but in a lower yield. As well as PhB(OH)_2_ (**2a–2f**), the reaction is compatible with other arylboronic acids, as shown by the reaction of **1a** with various *para*- (**2g–2i**), *meta*- (**2j**, **2k**, and **2o**), *ortho*- (**2l–2n**), and disubstituted (**2o** and **2p**) phenylboronic acids with acetoxy (**2g**), halide (**2h**, **2j**, **2m**, **2o**, and **2p**), vinyl (**2i**), cyano (**2k**), or methyl (**2l** and **2o**) groups. A heteroarylboronic acid also reacted smoothly to give **2q** in 93% yield and 98% ee. This example was the only instance where the minor diastereomer was detected (7 : 1 dr as determined by ^1^H NMR analysis of the crude reaction mixture). 2-Aminophenylboronic acid pinacol ester also reacted with **1a** to give **2n** in 48% yield and 56% ee.

**Table 2:** Enantioselective synthesis of chiral pyrrolidin-2-ones^a^

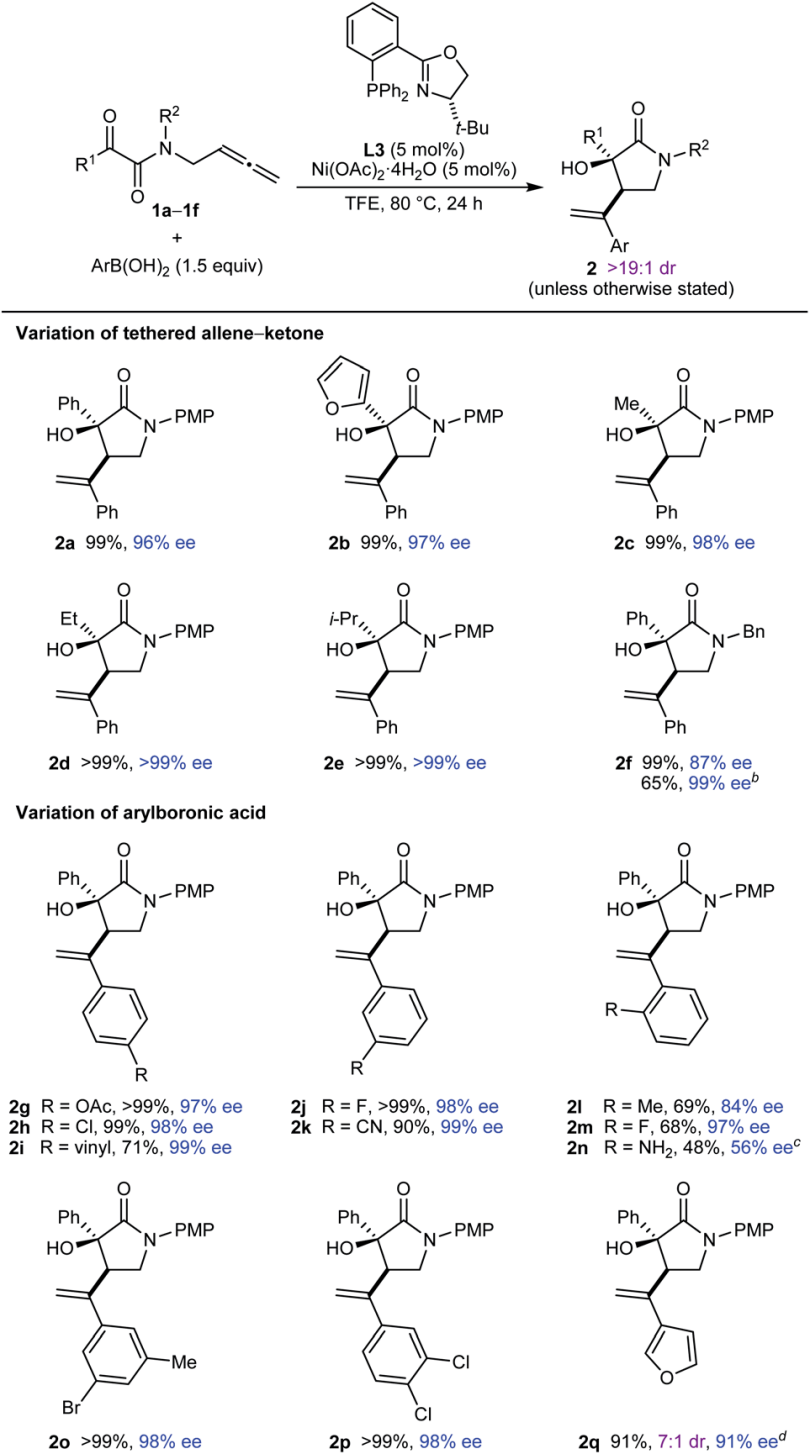

a Reactions were conducted using 0.30 mmol of **1**. Yields are of isolated products. Diastereomeric ratios were determined by ^1^H NMR analysis of the crude reaction mixtures. Enantiomeric excesses were determined by HPLC analysis on a chiral stationary phase.

b Conducted in MeCN instead of TFE.

c Conducted using the pinacol boronate instead of the boronic acid.

d The diastereomeric ratio of the crude reaction mixture was 7 : 1. Isolated as a 7 : 1 mixture of inseparable diastereomers.

The reaction also worked well with substrate **1g**, in which the nitrogen atom is unprotected, to give **2r** in 90% yield as a single observable diastereomer and 99% ee (eqn (1)).1
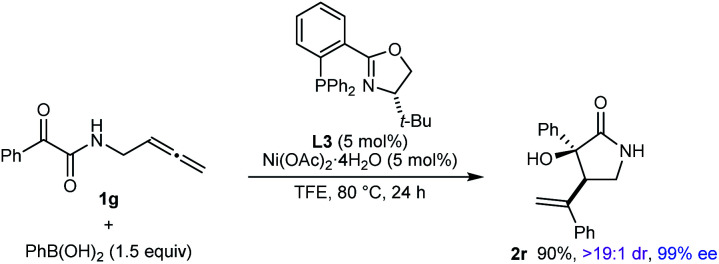


Notably, the process is not limited to the use of (hetero)arylboron reagents, as shown by the reaction of **1a** with potassium vinyltrifluoroborate to give 1,3-diene-containing pyrrolidin-2-one **3** in 65% yield and 93% ee (eqn (2)).2
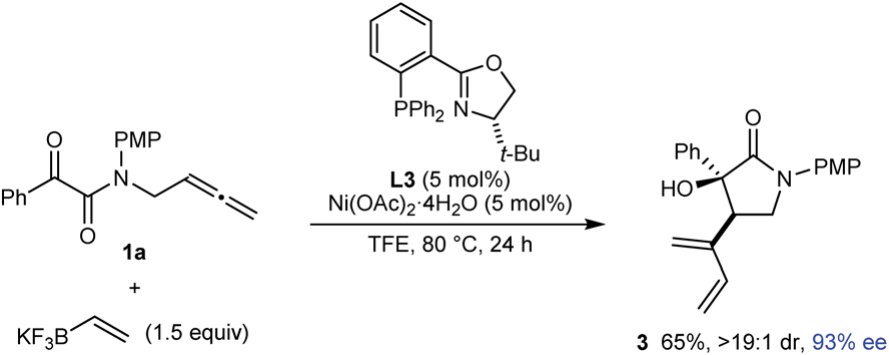


Pleasingly, this process is also not restricted to the preparation of chiral pyrrolidin-2-ones; it can be employed in the synthesis of other types of aza- and carbocycles, which were obtained in up to 96% yield and in 75–99% ee (Table 3). In several cases, superior results were obtained using MeCN in place of TFE as the solvent (**5c**, **5f–5h**, **5j**, and **5k**). Various pyrrolidines (**5a–5e**), cyclopentanes (**5f–5h**), piperidines (**5i** and **5j**), and a cyclohexane (**5k**) were successfully prepared using this chemistry. For the azacyclic products, the method is compatible with tosyl (**5a**, **5b**, **5i**, and **5j**), 4-methoxyphenyl (**5c** and **5d**), and 4-chlorophenyl (**5e**) substituents on the nitrogen atom. A comparison of the reactions producing **5a** and **5c**, and of **5b** and **5e**, shows the enantioselectivity is dependent on the nature of the nitrogen substituent but rationalization of these observations is difficult at the present time. Regarding the ketone substituent in the substrate, methyl (**5b**, **5e**, **5j**, and **5k**), *tert*-butyl (**5d**), and phenyl (**5a**, **5c**, and **5f–5i**) groups are tolerated, and increasing the size of this group had a beneficial effect on the enantioselectivity.

**Table 3:** Further exploration of substrate scope^a^

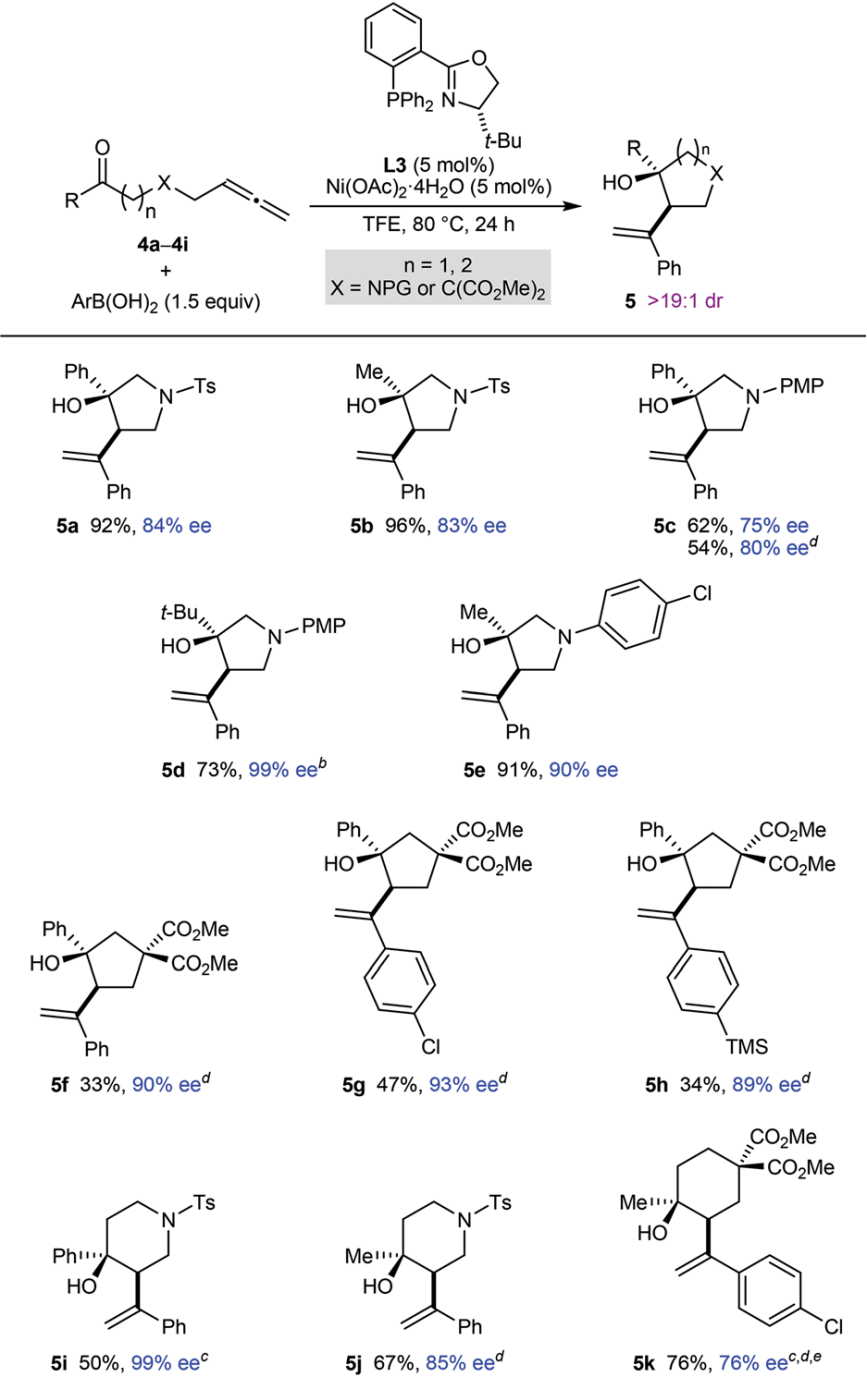

a Yields are of isolated products. Diastereomeric ratios were determined by ^1^H NMR analysis of the crude reaction mixtures. Enantiomeric excesses were determined by HPLC analysis on a chiral stationary phase.

b A 7.7 : 1 inseparable mixture of **5d** and the starting allene **4d** was obtained (the yield of **5d** has been adjusted accordingly).

c The reaction time was 48 h.

d Using MeCN as the solvent in place of TFE.

e Conducted using 10 mol% each of Ni(OAc)_2_·4H_2_O and **L3**.

Substrates that did not undergo arylative cyclization, but which only returned unreacted starting materials, are shown in Fig. 2. The failure of **4j** to cyclize indicates that an amide, sulfonamide, aniline, or a malonyl group at the allylic position of the tether connecting the allene and the ketone is important for reactivity. To probe whether these features aid cyclization by increasing the proportion of reactive conformers by the geminal disubstituent^18^ or related^19^ effects, we examined substrate **4k**, which contains a geminal dimethyl group. Because **4k** also failed to cyclize, we speculate that the aforementioned nitrogen-containing groups or a malonyl group in the tether aid reactivity not by a geminal disubstituent or related effect, but perhaps by being able to act as a directing group and coordinating to catalytic nickel species to aid arylnickelation of the allene.

**Fig. 2:**
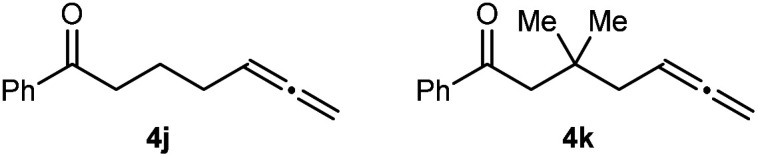
Substrates that do not undergo arylative cyclization.

A possible catalytic cycle for these reactions, using **1a** and PhB(OH)_2_ as representative reaction partners, is shown in Scheme 2. After formation of the chiral nickel complex **6**, which in principle could have acetate, hydroxide, or 2,2,2-trifluoroethoxide ligands resulting from the species present in the reaction, transmetalation with PhB(OH)_2_ gives phenylnickel species **7** with the phenyl ligand *trans* to the π-accepting phosphorus atom rather than the stronger σ-donating nitrogen atom. Phenylnickelation of the internal alkene of substrate **1a**, directed by the amide carbonyl group,^20^ would give σ-allylnickel species **8**, which could interconvert with alternative allylnickel species (*E*)-**9** and (*Z*)-**9** by a series of σ–π–σ isomerizations. The stereochemical outcome of the reactions is consistent with intramolecular nucleophilic allylation through a cyclic six-membered chair-like conformation in the cationic *Z*-σ-allylnickel species **10a**, which is formed by substitution of the anionic oxygen ligand of (*Z*)-**9** from the nickel center by coordination of the ketone.^21^ After reassociation of an anionic oxygen ligand, the resulting nickel alkoxide **11** can then undergo protonolysis to release pyrrolidin-2-one **2a** and regenerate **6**. Nucleophilic allylation through the diastereomeric *Z*-σ-allylnickel species **10b** to give the minor enantiomer *ent*-**2a** is likely to be disfavored because of unfavorable non-bonding interactions between the *tert*-butyl group of the chiral ligand and a phenyl group. Although we currently favor the pathway shown in Scheme 2, nucleophilic allylation through alternative, five-coordinate nickel complexes cannot be excluded.

**Scheme 2:**
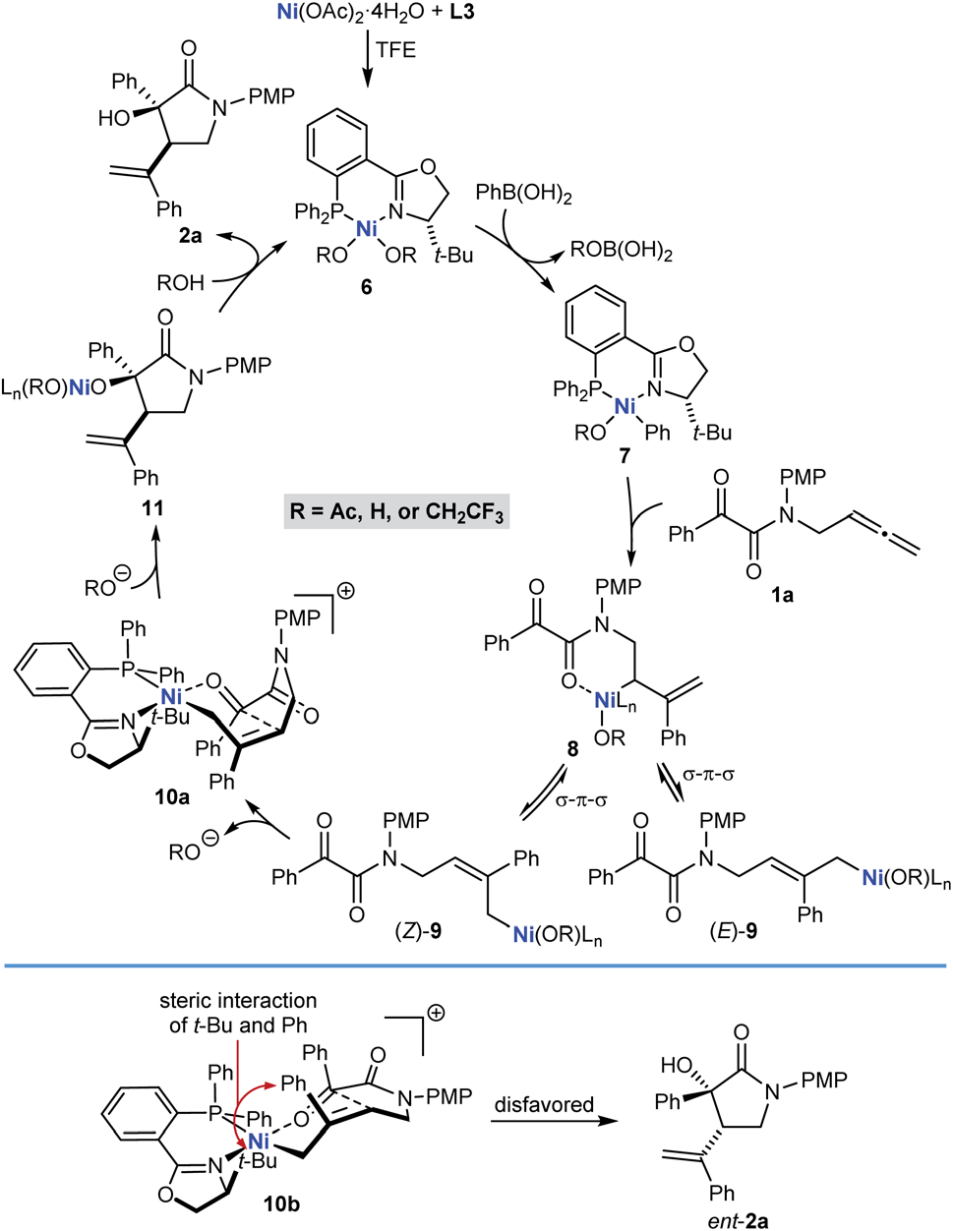
Possible catalytic cycle.

Further transformations of a representative product **2a** are shown in Scheme 3. First, removal of the 4-methoxyphenyl protecting group from the nitrogen atom of a representative product **2a** was readily accomplished in using CAN in MeCN/H_2_O (1 : 1) at −10 °C for 30 min to give **2r** in 98% yield. Alternatively, oxidative cleavage of the alkene of **2a** using K_2_[OsO_2_(OH)_4_] and NaIO_4_ gave **12** in 91% yield.

**Scheme 3:**
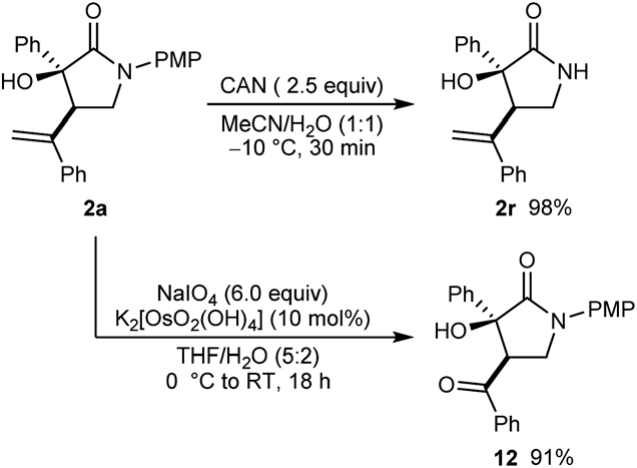
Further transformations of **2a**.

The results herein demonstrate the utility of allylnickel species generated by the carbonickelation of allenes to engage in stereoselective nucleophilic 1,2-additions to ketones to form a range of tertiary-alcohol-containing aza- and carbocycles in high diastereo- and enantioselectivities. Compared with related palladium-catalyzed reactions reported previously, which focused on tethered allene-aldehydes as the substrates,^16^ this nickel-catalyzed process demonstrates that less reactive ketones can serve as effective electrophiles to enable the synthesis of a range of tertiary-alcohol-containing aza- and carbocycles. Further applications of enantioselective catalytic nucleophilic allylations of allylnickel species will be reported in due course.^22^

## Conflicts of interest

There are no conflicts to declare.

## Supplementary Material

SC-011-C9SC05246A-s001

SC-011-C9SC05246A-s002
