# Supplementary material for: Enantioselective nickel-catalyzed arylative and alkenylative intramolecular 1,2-allylations of tethered allene–ketones
Source: Chem Sci. 2020 Jan 21;11(9):2401–6. doi: 10.1039/c9sc05246a (PMC8157472; doi:10.1039/c9sc05246a)
Supplement: SC-011-C9SC05246A-s001 [file SC-011-C9SC05246A-s001.pdf]

Electronic Supplementary Material (ESI) for Chemical Science.
This journal is © The Royal Society of Chemistry 2020



# Enantioselective nickel-catalyzed arylative and alkenylative intramolecular 1,2-allylations of tethered allene–ketones

Riccardo Di Sanza,[†,a,b] Thi Le Nhon Nguyen,[†,a,b] Naeem Iqbal,[a,b] Stephen P. Argent,[§,b] William Lewis,[§,b] and Hon Wai Lam*,[a,b]

[†] These authors contributed equally

[§] To whom enquiries regarding X-ray crystallography should be addressed

[a] *The GlaxoSmithKline Carbon Neutral Laboratories for Sustainable Chemistry, University of Nottingham, Jubilee Campus, Triumph Road, Nottingham, NG7 2TU, United Kingdom*

and

[b] *School of Chemistry, University of Nottingham, University Park, Nottingham, NG7 2RD, United Kingdom*

**Contents**

General Information....2
Preparation of Tethered Allene–Ketones....3
Enantioselective Nickel-Catalyzed Arylative and Alkenylative Intramolecular Allylations....17
Further Transformations....35
NMR Spectra....37
HPLC Traces....90
References....119

**General Information**

All air-sensitive reactions were carried out under an inert atmosphere using oven-dried apparatus. 2,2,2-Trifluoroethanol (TFE) was purchased from Alfa Aesar and used as received. MeCN was dried and purified by passage through activated alumina columns using a solvent purification system. All commercially available reagents were used as received unless otherwise stated. Petroleum ether refers to Sigma-Aldrich product 24587 (petroleum ether boiling point 40–60 °C). Thin layer chromatography (TLC) was performed on Merck DF Alufoilien 60F254 0.2 mm precoated plates. Compounds were visualized by exposure to UV light or by dipping the plates into solutions of potassium permanganate or vanillin followed by gentle heating. Column chromatography was carried out using silica gel (Fisher Scientific 60 Å particle size 35-70 micron or Fluorochem 60 Å particle size 40-63 micron). Melting points were recorded on a Gallenkamp melting point apparatus and are uncorrected. The solvent of recrystallization is reported in parentheses. Infrared (IR) spectra were recorded on a Bruker platinum ALPHA FTIR spectrometer on the neat compound using the attenuated total refraction technique. NMR spectra were acquired on Bruker Ascend 400 or Ascend 500 spectrometers. $^{1}$H and $^{13}$C NMR spectra were referenced to external tetramethylsilane via the residual protonated solvent ($^{1}$H) or the solvent itself ($^{13}$C). $^{19}$F NMR spectra were referenced through the solvent lock ($^{2}$H) signal according to the IUPAC-recommended secondary referencing method following Bruker protocols. All chemical shifts are reported in parts per million (ppm). For $CDCl_3$, the shifts are referenced to 7.26 ppm for $^{1}$H NMR spectroscopy and 77.16 ppm for $^{13}$C NMR spectroscopy. Abbreviations used in the description of resonances are: s (singlet), d (doublet), t (triplet), q (quartet), quin (quintet), sept (septet), br (broad) and m (multiplet) Coupling constants ($J$) are quoted to the nearest 0.1 Hz. $^{13}$C NMR assignments were made using the DEPT sequence with secondary pulses at 90° and 135°. High-resolution mass spectra were recorded using electrospray ionization (ESI). X-ray diffraction data were collected at 120 K on an Agilent SuperNova diffractometer using CuKα radiation. Chiral HPLC analysis was performed on an Agilent 1290 series instrument using 4.6 × 250 mm columns. 2-[2-(Diphenylphosphino)ethyl]pyridine was used as an achiral ligand to obtain authentic racemic compounds.

## Preparation of Tethered Allene–Ketones

## Preparation of Tethered Allene–Ketone 1a

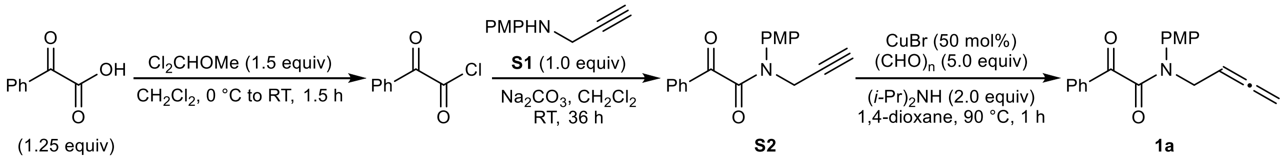


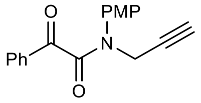

***N*-(4-Methoxyphenyl)-2-oxo-2-phenyl-*N*-(prop-2-yn-1-yl)acetamide (S2).** To a solution of phenylglyoxylic acid (4.69 g, 31.3 mmol) in $CH_2Cl_2$ (25 mL) at 0 °C under inert atmosphere was added dichloromethyl methyl ether (3.39 mL, 37.5 mmol) dropwise. The mixture was stirred at room temperature for 1.5 h and then diluted with $CH_2Cl_2$ (80 mL). $Na_2CO_3$ (26.3 g, 250 mmol) was added, followed by a solution of propargylamine **S1**[1] (4.03 g, 25.0 mmol) in $CH_2Cl_2$ (20 mL). The mixture was stirred at room temperature for 36 h, quenched with $H_2O$ (100 mL), and extracted with $CH_2Cl_2$ (2 × 50 mL). The combined organic layers were dried ($Na_2SO_4$), filtered, and concentrated *in vacuo*. Purification of the residue by column chromatography (30% EtOAc/petroleum ether) gave *alkyne* **S2** (6.99 g, 95%) as a white solid. $R_f$ = 0.36 (30% EtOAc/petroleum ether); m.p. 83–85 °C ($Et_2O$); IR 3309, 3275, 2975, 2840, 1651 (C=O), 1508, 1434, 1255, 1240, 1216, 1167, 1020, 944, 837, 713, 619, 582, 540 $cm^{-1}$; $^1$H NMR (400 MHz, $CDCl_3$) δ 7.85–7.82 (2H, m, Ar**H**), 7.60–7.55 (1H, m, Ar**H**), 7.46–7.41 (2H, m, Ar**H**), 7.17–7.13 (2H, m, Ar**H**), 6.76–6.72 (2H, m, Ar**H**), 4.63 (2H, d, *J* = 2.5 Hz, NC**H**$_2$), 3.73 (3H, s, OC**H**$_3$), 2.31 (1H, t, *J* = 2.5 Hz, ≡C**H**); $^{13}$C NMR (101 MHz, $CDCl_3$) δ 190.5 (C), 166.9 (C), 159.7 (C), 134.5 (CH), 133.5 (C), 131.4 (C), 129.9 (2 × CH), 129.5 (2 × CH), 128.9 (2 × CH), 114.7 (CH), 78.1 (C), 73.2 (CH), 55.5 ($CH_3$), 38.2 ($CH_2$); HRMS (ESI) Exact mass calculated for $[C_{18}H_{16}NO_3]^+$ $[M + H]^+$: 294.1125, found: 294.1132.

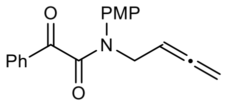

***N*-(Buta-2,3-dien-1-yl)-*N*-(4-methoxyphenyl)-2-oxo-2-phenylacetamide (1a).** To a solution of alkyne **S2** (5.86 g, 20.0 mmol) in 1,4-dioxane (100 mL) at room temperature under inert atmosphere was added paraformaldehyde (3.00 g, 100 mmol), CuBr (1.43 g, 10.0 mmol), and diisopropylamine (5.61 mL, 40.0 mmol). The reaction was heated at 90 °C for 1 h, cooled to room temperature, filtered through a pad of celite using EtOAc as eluent, and concentrated *in vacuo*. Purification of the residue by column chromatography (20% EtOAc/petroleum ether) gave *allene* **1a** (2.82 g, 46%) as a 10:1 mixture of rotamers as a pale yellow solid. $R_f$ = 0.42 (30% EtOAc/petroleum ether); m.p. 50–53 °C (EtOAc); IR 2958, 2926, 1960 (C=C=C), 1656 (C=O), 1592, 1509, 1450, 1427, 1297, 1242, 1211, 1169, 1025, 945, 838, 814, 756,

732, 717, 688, 656, 550, 445 cm$^{-1}$; $^{1}$H NMR (400 MHz, $CDCl_3$) *major rotamer*: δ 7.87–7.84 (2H, m, Ar**H**), 7.59–7.55 (1H, m, Ar**H**), 7.46–7.41 (2H, m, Ar**H**), 7.08–7.04 (2H, m, Ar**H**), 6.74–6.70 (2H, m, Ar**H**), 5.32 (1H, quin, *J* = 6.6 Hz, $CH_2C$**H**=), 4.81 (2H, dt, *J* = 6.6, 2.7 Hz, =C$\mathbf{H_2}$), 4.45 (2H, dt, *J* = 6.6, 2.7 Hz, NC$\mathbf{H_2}$), 3.72 (3H, s, OC$\mathbf{H_3}$); *minor rotamer*: δ 8.07–8.05 (2H, m, Ar**H**), 7.69–7.64 (1H, m, Ar**H**), 7.54–7.52 (2H, m, Ar**H**), 7.33–7.30 (2H, m, Ar**H**), 7.00–6.96 (2H, m, Ar**H**), 5.14 (1H, quin, *J* = 6.5 Hz, $NCH_2C$**H**), 4.59 (2H, dt, *J* = 6.6, 2.7 Hz, =C$\mathbf{H_2}$), 4.20 (2H, dt, *J* = 6.5, 2.7 Hz, NC$\mathbf{H_2}$CH), 3.84 (3H, s, OC$\mathbf{H_3}$); $^{13}$C NMR (101 MHz, $CDCl_3$) *major rotamer*: δ 209.8 (C), 191.0 (C), 167.1 (C), 159.4 (C), 134.3 (CH), 133.6 (C), 132.1 (C), 129.8 (2 × CH), 129.5 (2 × CH), 128.9 (2 × CH), 114.6 (2 × CH), 85.9 (CH), 77.0 ($CH_2$), 55.5 ($CH_3$), 47.9 ($CH_2$); *observable signals of minor rotamer*: δ 130.1 (2 × CH), 129.1 (2 × CH), 128.3 (2 × CH), 114.8 (CH); HRMS (ESI) Exact mass calculated for $[C_{19}H_{18}NO_3]^+$ $[M+H]^+$: 308.1281, found: 308.1278.

**Preparation of Tethered Allene–Ketone 1b**

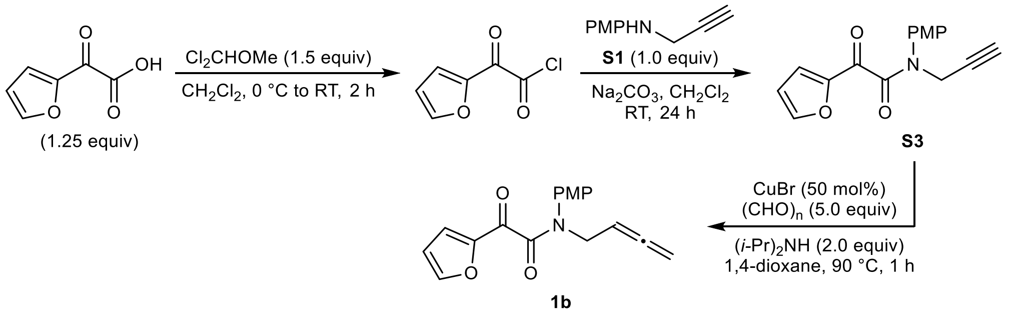


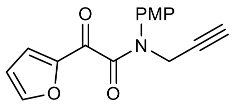


**2-(Furan-2-yl)-*N*-(4-methoxyphenyl)-2-oxo-*N*-(prop-2-yn-1-yl)acetamide (S3).** To a solution of α-oxo-2-furanacetic acid (1.09 g, 7.76 mmol) in $CH_2Cl_2$ (15 mL) at 0 °C under inert atmosphere was added dichloromethyl methyl ether (0.84 mL, 9.31 mmol) dropwise. The mixture was stirred at room temperature for 2 h and then diluted with $CH_2Cl_2$ (5 mL). $Na_2CO_3$ (6.58 g, 62.1 mmol) was added, followed by a solution of propargylamine **S1**[1] (1.00 g, 6.21 mmol) in $CH_2Cl_2$ (5 mL). The mixture was stirred at room temperature for 24 h, quenched with $H_2O$ (20 mL), and extracted with $CH_2Cl_2$ (2 × 20 mL). The combined organic layers were dried ($Na_2SO_4$), filtered, and concentrated *in vacuo*. Purification of the residue by column chromatography (30% EtOAc/petroleum ether) gave *alkyne* **S3** (1.74 g, 99%) as a pale yellow solid. $R_f$ = 0.29 (30% EtOAc/petroleum ether); m.p. 55–59 °C ($Et_2O$); IR 3283, 3125, 2835, 1640 (C=O), 1509, 1459, 1390, 1214, 1164, 1023, 928, 881, 801, 768, 724, 617, 585, 540, 408 cm$^{-1}$; $^{1}$H NMR (400 MHz, $CDCl_3$) δ 7.63 (1H, dd, *J* = 1.7, 0.8 Hz, Ar**H**), 7.27–7.26 (1H, m, Ar**H**), 7.21–7.17 (2H, m, Ar**H**), 6.81–6.77 (2H, m, Ar**H**), 6.54 (1H, dd, *J* = 3.6, 1.7 Hz, Ar**H**), 4.58 (2H, d, *J* = 2.5 Hz, NC$\mathbf{H_2}$), 3.75 (3H, s, OC$\mathbf{H_3}$), 2.28 (1H, t, *J* = 2.5 Hz, ≡C**H**); $^{13}$C NMR (101 MHz, $CDCl_3$)

δ 165.4 (C), 159.8 (C), 150.3 (C), 148.4 (2 × CH), 131.4 (C), 129.6 (2 × CH), 121.4 (C), 114.7 (2 ×2 CH), 112.9 (CH), 77.9 (C), 73.2 (CH), 55.5 ($CH_3$), 38.4 ($CH_2$); HRMS (ESI) Exact mass calculated for $[C_{16}H_{13}NNaO_4]^+$ $[M+Na]^+$: 306.0737, found: 306.0740.

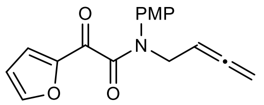


***N*-(Buta-2,3-dien-1-yl)-2-(furan-2-yl)-N-(4-methoxyphenyl)-2-oxoacetamide (1b).** To a solution of alkyne **S3** (708 mg, 2.50 mmol) in 1,4-dioxane (12.5 mL) at room temperature under inert atmosphere was added paraformaldehyde (375 mg, 12.5 mmol), CuBr (287 mg, 2.00 mmol), and diisopropylamine (0.70 mL, 5.0 mmol). The reaction was heated at 90 °C for 1 h, cooled to room temperature, filtered through a pad of celite using EtOAc as eluent, and concentrated *in vacuo*. Purification of the residue by column chromatography (40% EtOAc/petroleum ether) gave *allene* **1b** (293 mg, 39%) as a 10:1 mixture of rotamers as a pale yellow solid. $R_f$ = 0.41 (40% EtOAc/petroleum ether); m.p. 79–81 °C ($Et_2O$); IR 3116, 1967 (C=C=C), 1650 (C=O), 1557, 1456, 1436, 1388, 1248, 1165, 1023, 960, 839, 805, 751, 670, 622, 592, 548, 437 $cm^{-1}$; $^1H$ NMR (400 MHz, $CDCl_3$) *major rotamer*: δ 7.61 (1H, dd, *J* = 1.6, 0.7 Hz, Ar**H**), 7.28–7.25 (1H, m, Ar**H**), 7.12–7.08 (2H, m, Ar**H**), 6.79–6.75 (2H, m, Ar**H**), 6.53 (1H, dd, *J* = 3.6, 1.7 Hz, Ar**H**), 5.28 (1H, quin, *J* = 6.7 Hz, $CH_2C$**H**=), 4.78 (2H, dt, *J* = 6.6, 2.6 Hz, =C**H**$_2$), 4.40 (2H, dt, *J* = 6.6, 2.6 Hz, NC**H**$_2$), 3.75 (3H, s, OC**H**$_3$); *minor rotamer*: δ 7.75 (1H, dd, *J* = 1.7, 0.7 Hz, Ar**H**), 7.43 (1H, dd, *J* = 3.6, 0.8 Hz, Ar**H**), 7.28–7.27 (2H, m, Ar**H**), 6.98–6.94 (2H, m, Ar**H**), 6.63 (1H, dd, *J* = 3.6, 1.6 Hz, ArC**H**), 5.20 (1H, quin, *J* = 6.5 Hz, $CH_2C$**H**=), 4.66 (2H, dt, *J* = 6.6, 2.7 Hz, =C**H**$_2$), 4.29 (2H, dt, *J* = 6.4, 2.7 Hz, NC**H**$_2$), 3.83 (3H, s, OC**H**$_3$); $^{13}C$ NMR (101 MHz, $CDCl_3$) *major rotamer*: δ 209.8 (C), 178.3 (C), 165.7 (C), 159.5 (C), 150.5 (C), 148.2 (CH), 132.2 (C), 129.6 (2 × CH), 121.1 (CH), 114.7 (2 × CH), 112.8 (CH), 85.7 (CH), 76.9 ($CH_2$), 55.5 ($CH_3$), 48.1 ($CH_2$); *observable signals of minor rotamer*: δ 148.9 (CH), 128.1 (CH), 114.7 (CH), 113.1 (CH), 110.1 (CH), 87.2 (CH), 55.6 ($CH_3$), 50.4 ($CH_2$); HRMS (ESI) Exact mass calculated for $[C_{17}H_{16}NO_4]^+$ $[M+H]^+$: 298.1074, found: 298.1077.

***N*-(Buta-2,3-dien-1-yl)-4-methoxyaniline (S5)**

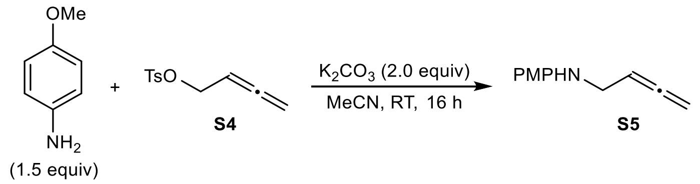


To a solution of *p*-anisidine (3.14 g, 25.5 mmol) in MeCN (68 mL) at room temperature under inert atmosphere was added allylic tosylate **S4**[2] (3.82 g, 17.0 mmol) and $K_2CO_3$ (4.70 g, 34.0 mmol), and the mixture was stirred at room temperature for 16 h. The mixture was partitioned between $Et_2O$ (50 mL) and saturated aqueous $NaHCO_3$ solution (50 mL), and the organic layer was separated and

washed with saturated aqueous $NaHCO_3$ solution (2 × 30 mL), dried ($Na_2SO_4$), filtered, and concentrated *in vacuo*. Purification of the residue by column chromatography (20% EtOAc/petroleum ether) gave *allene* **S5** (1.50 g, 50%) as a pale yellow oil. $R_f$ = 0.48 (20% EtOAc/petroleum ether); IR 3387 (NH), 2831, 1954, 1617, 1509, 1463, 1407, 1294, 1232, 1178, 1116, 1034, 847, 817, 517 $cm^{-1}$; $^1$H NMR (400 MHz, $CDCl_3$) δ 6.83–6.78 (2H, m, Ar**H**), 6.64–6.60 (2H, m, Ar**H**), 5.33–5.25 (1H, m, $NCH_2C$**H**), 4.83 (2H, dt, *J* = 6.6, 3.3 Hz, =C**H**$_2$), 3.76 (3H, s, OC**H**$_3$), 3.73 (2H, dt, *J* = 6.2, 3.2 Hz, NC**H**$_2$); $^{13}$C NMR (101 MHz, $CDCl_3$) δ 208.3 (C), 152.4 (C), 142.0 (C), 114.9 (2 × CH), 114.7 (2 × CH), 88.9 (CH), 77.0 ($CH_2$), 55.9 ($CH_3$), 43.3 ($CH_2$); HRMS (ESI) Exact mass calculated for $[C_{11}H_{14}NO]^+$ $[M+H]^+$: 176.1070, found: 176.1071.

### *N*-(Buta-2,3-dien-1-yl)-*N*-(4-methoxyphenyl)-2-oxopropanamide (1c)

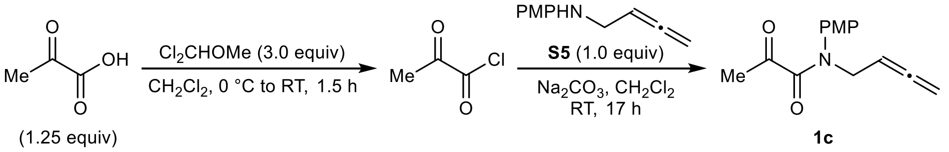


To a solution of pyruvic acid (95.1 mg, 1.08 mmol) in $CH_2Cl_2$ (1 mL) at 0 °C under inert atmosphere was added dichloromethyl methyl ether (117 μL, 1.30 mmol) dropwise. The mixture was stirred at room temperature for 1.5 h and then diluted with $CH_2Cl_2$ (3.5 mL). $Na_2CO_3$ (922 mg, 8.70 mmol) was added, followed by a solution of allenylamine **S5** (152 mg, 0.87 mmol) in $CH_2Cl_2$ (0.5 mL). The mixture was stirred at room temperature for 17 h, quenched with $H_2O$ (5 mL), and extracted with $CH_2Cl_2$ (2 × 5 mL). The combined organic layers were dried ($Na_2SO_4$), filtered, and concentrated *in vacuo*. Purification of the residue by column chromatography (40% EtOAc/petroleum ether) gave *allene* **1c** (139 mg, 65%) as a 14:1 mixture of rotamers as a white solid. $R_f$ = 0.42 (40% EtOAc/petroleum ether); m.p. 38–39 °C ($Et_2O$); IR 3010, 2923, 1955 (C=C=C), 1707, 1641 (C=O), 1509, 1433, 1364, 1302, 1248, 1228, 1164, 1053, 1028, 945, 845, 731, 629, 580, 549, 497, 461, 428 $cm^{-1}$; $^1$H NMR (400 MHz, $CDCl_3$) *major rotamer*: δ 7.13–7.09 (2H, m, Ar**H**), 6.89–6.85 (2H, m, Ar**H**), 5.21 (1H, quin, *J* = 6.6 Hz, $CH_2C$**H**=), 4.76 (2H, dt, *J* = 6.6, 2.7 Hz, =C**H**$_2$), 4.30 (2H, dtd, *J* = 6.6, 2.7 Hz, NC**H**$_2$), 3.80 (3H, s, OC**H**$_3$), 2.17 (3H, s, C**H**$_3$C=O); *minor rotamer*: δ 7.19 (2H, d, *J* = 8.9 Hz, Ar**H**), 6.92 (2H, d, *J* = 8.9 Hz, Ar**H**), 5.28–5.26 (1H, m, $CH_2C$**H**=), 4.82–4.81 (2H, m, =C**H**$_2$), 4.27–4.26 (2H, m, NC**H**$_2$), 3.80 (3H, s, OC**H**$_3$), 2.49 (3H, s, C**H**$_3$C=O); $^{13}$C NMR (101 MHz, $CDCl_3$) *major rotamer*: δ 209.7 (C), 198.2 (C), 167.3 (C), 159.6 (C), 132.4 (C), 129.2 (2 × CH), 114.8 (2 × CH), 85.7 (CH), 76.9 ($CH_2$), 55.6 ($CH_3$), 47.9 ($CH_2$), 28.0 ($CH_3$); *observable signals of minor rotamer*: δ 128.0 (2 × CH), 114.6 (2 × CH), 88.0 (CH), 78.0 ($CH_2$), 49.9 ($CH_2$), 27.7 ($CH_3$); HRMS (ESI) Exact mass calculated for $[C_{14}H_{16}NO_3]^+$ $[M+H]^+$: 246.1125, found: 246.1127.

### *N*-(Buta-2,3-dien-1-yl)-N-(4-methoxyphenyl)-2-oxobutanamide (1d)

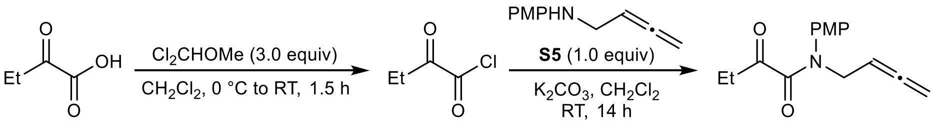


(1.25 equiv) **1d**

To a solution of 2-ketobutyric acid (383 mg, 3.75 mmol) in $CH_2Cl_2$ (1.5 mL) at 0 °C under inert atmosphere was added dichloromethyl methyl ether (0.41 mL, 4.5 mmol) dropwise. The mixture was stirred for 5 h at room temperature and then diluted with $CH_2Cl_2$ (8 mL). $K_2CO_3$ (2.07 g, 15.0 mmol) was added followed by a solution of allenylamine **S5** (263 mg, 1.50 mmol) in $CH_2Cl_2$ (0.5 mL). The mixture was stirred at room temperature for 14 h, quenched with $H_2O$ (50 mL), and extracted with $CH_2Cl_2$ (2 × 50 mL). The combined organic layers were dried ($Na_2SO_4$), filtered, and concentrated *in vacuo*. Purification of the residue by column chromatography (20% EtOAc/petroleum ether) gave *allene* **1d** (332 mg, 85%) as a 14:1 mixture of rotamers as a pale yellow amorphous solid. $R_f$ = 0.30 (20% EtOAc/petroleum ether); IR 2981, 1955 (C=C=C), 1714, 1645 (C=O), 1509, 1441, 1404, 1295, 1248, 1217, 1171, 1116, 1028, 835, 725, 613, 544 $cm^{-1}$; $^1$H NMR (400 MHz, $CDCl_3$) *major rotamer*: δ 7.13-7.08 (2H, m, Ar**H**), 6.88-6.84 (2H, m, Ar**H**), 5.21 (1H, quin, *J* = 6.6 Hz, $CH_2C$**H**=), 4.75 (2H, dt, *J* = 6.7, 2.7 Hz, =C**H**$_2$), 4.29 (2H, dt, *J* = 6.6, 2.7 Hz, NC**H**$_2$), 3.80 (3H, s, OC**H**$_3$), 2.52 (2H, q, *J* = 7.3 Hz, $CH_3$C**H**$_2$), 0.91 (3H, t, *J* = 7.3 Hz, C**H**$_3CH_2$); *minor rotamer*: δ 7.19–7.11 (2H, m, Ar**H**), 6.93–6.91 (2H, m, Ar**H**), 5.24–5.20 (1H, m, $CH_2C$**H**=), 4.79 (2H, dt, *J* = 6.4, 2.8 Hz, =C**H**$_2$), 4.23 (2H, dt, *J* = 6.0, 2.8 Hz, NC**H**$_2$), 3.81 (3H, s, OC**H**$_3$), 2.89 (2H, q, *J* = 7.2 Hz, $CH_3$C**H**$_2$), 1.18 (3H, t, *J* = 7.2 Hz, C**H**$_3CH_2$); $^{13}$C NMR (101 MHz, $CDCl_3$) *major rotamer*: δ 209.7 (C), 201.6 (C), 167.7 (C), 159.5 (C), 132.4 (C), 129.3 (2 × CH), 114.7 (2 × CH), 85.7 (CH), 76.9 ($CH_2$), 55.6 ($CH_3$), 47.9 ($CH_2$), 33.8 ($CH_2$), 6.9 ($CH_3$); *observable signals of minor rotamer*: δ 209.1 (C), 128.1 (2 × CH), 114.6 (2 × CH), 87.7 (CH), 77.8 ($CH_2$), 55.6 ($CH_3$), 50.0 ($CH_2$), 33.4 ($CH_2$), 7.1 ($CH_3$); HRMS (ESI) Exact mass calculated for $[C_{15}H_{18}NO_3]^+$ $[M+H]^+$: 260.1281, found: 260.1286.

### *N*-(Buta-2,3-dien-1-yl)-N-(4-methoxyphenyl)-3-methyl-2-oxobutanamide (1e)

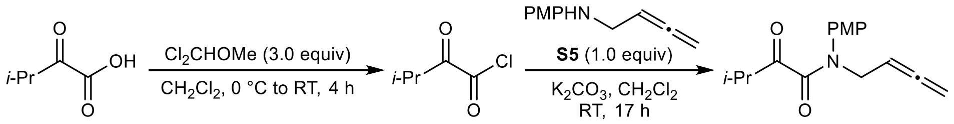


(1.25 equiv) **1e**

To a solution of 3-methyl-2-oxobutyryl acid (730 mg, 6.29 mmol) in $CH_2Cl_2$ (6.5 mL) at 0 °C under inert atmosphere was added dichloromethyl methyl ether (0.68 mL, 7.5 mmol) dropwise. The mixture was stirred at room temperature for 4 h and then diluted with $CH_2Cl_2$ (10 mL). $K_2CO_3$ (3.46 g, 25.0 mmol) was added followed by a solution of allenylamine **S5** (438 mg, 2.50 mmol) in $CH_2Cl_2$ (0.5 mL). The mixture was stirred at room temperature for 17 h, quenched with $H_2O$ (10 mL), and

extracted with $CH_2Cl_2$ (2 × 10 mL). The combined organic layers were dried ($Na_2SO_4$), filtered, and concentrated *in vacuo*. Purification of the residue by column chromatography (20% EtOAc/petroleum ether) gave *allene* **1e** (205 mg, 30%) as a 14:1 mixture of rotamers as a pale yellow oil. $R_f$ = 0.39 (20% EtOAc/petroleum ether); IR 2971, 1956 (C=C=C), 1712, 1644 (C=O), 1510, 1463, 1441, 1296, 1248, 1217, 1027, 836, 730, 619, 554 $cm^{-1}$; $^1$H NMR (400 MHz, $CDCl_3$) *major rotamer*: δ 7.14–7.10 (2H, m, Ar**H**), 6.87–6.83 (2H, m, Ar**H**), 5.22 (1H, quin, *J* = 6.6 Hz, $CH_2$C**H**=), 4.75 (2H, dt, *J* = 6.7, 2.6 Hz, =C**H**$_2$), 4.30 (2H, dt, *J* = 6.7, 2.7 Hz, NC**H**$_2$), 3.80 (3H, s, OC**H**$_3$), 2.78 (1H, sept, *J* = 6.9 Hz, $(CH_3)_2$C**H**), 0.98 (6H, d, *J* = 7.0 Hz, (C**H**$_3$)$_2$CH); *minor rotamer*: δ 7.18–7.16 (2H, m, Ar**H**), 6.94–6.92 (2H, m, Ar**H**), 5.22 (1H, quin, *J* = 6.6 Hz, $CH_2$C**H**=), 4.74–4.72 (2H, m, =C**H**$_2$), 4.19–4.17 (2H, m, NC**H**$_2$), 3.81 (3H, s, OC**H**$_3$), 3.27 (1H, sept, *J* = 7.0 Hz, $(CH_3)_2$C**H**), 1.23 (6H, d, *J* = 7.0 Hz, (C**H**$_3$)$_2$CH); $^{13}$C NMR (101 MHz, $CDCl_3$) *major rotamer*: δ 209.7 (C), 204.5 (C), 167.4 (C), 159.4 (C), 132.4 (C), 129.7 (2 × CH), 114.5 (2 × CH), 85.8 (CH), 76.9 ($CH_2$), 55.55 ($CH_3$), 48.1 ($CH_2$), 38.1 (CH), 17.3 (2 × $CH_3$); *observable signals of minor rotamer*: δ 209.4 (C), 128.3 (2 × CH), 114.7 (2 × CH), 87.1 (CH), 77.4 ($CH_2$), 55.59 ($CH_3$), 50.0 ($CH_2$), 37.8 (CH), 17.2 (2 × $CH_3$); HRMS (ESI) Exact mass calculated for $[C_{16}H_{20}NO_3]^+$ $[M+H]^+$: 274.1438, found: 274.1442.

**Preparation of Tethered Allene–Ketone 1f**

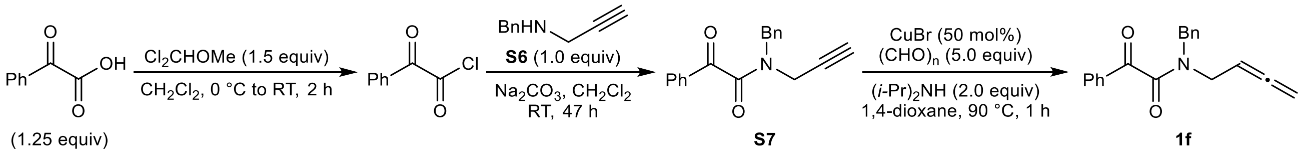


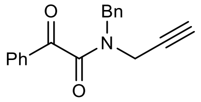


***N*-Benzyl-2-oxo-2-phenyl-*N*-(prop-2-yn-1-yl)acetamide (S7).** To a solution of phenylglyoxylic acid (2.82 g, 18.8 mmol) in $CH_2Cl_2$ (20 mL) at 0 °C under inert atmosphere was added dichloromethyl methyl ether (2.04 mL, 22.5 mmol) dropwise. The mixture was stirred at room temperature for 1.5 h and then diluted with $CH_2Cl_2$ (50 mL). $Na_2CO_3$ (15.9 g, 150 mmol) was added followed by a solution of propargylamine **S6**[3] (2.18 g, 15.0 mmol) in $CH_2Cl_2$ (5 mL). The reaction mixture was stirred at room temperature for 47 h, quenched with $H_2O$ (30 mL), and extracted with $CH_2Cl_2$ (2 × 30 mL). The combined organic layers were dried ($Na_2SO_4$), filtered, and concentrated *in vacuo* to leave *alkyne* **S7** (4.15 g, >99%) as a 1.7:1 mixture of rotamers as a pale yellow oil. $R_f$ = 0.46 (30% EtOAc/petroleum ether); IR 3287, 2978, 1817, 1678, 1643 (C=O), 1440, 1256, 1203, 1175, 947, 722, 696, 660, 596, 460 $cm^{-1}$; $^1$H NMR (400 MHz, $CDCl_3$) *major rotamer*: δ 8.00-7.98 (2H, m, Ar**H**), 7.67-7.63 (2H, m, Ar**H**), 7.54-7.49 (3H, m, Ar**H**), 7.34-7.31 (3H, m, Ar**H**), 4.53 (2H, s, C**H**$_2$Ph), 4.24 (2H, d, *J* = 2.5 Hz, C**H**$_2$C≡), 2.35 (1H, t, *J* = 2.5 Hz, ≡C**H**); *minor rotamer*; δ 8.14-8.12 (1H, m, Ar**H**), 8.00-7.98 (2H, m, Ar**H**), 7.54-7.49 (1H, m, Ar**H**), 7.48-7.46 (1H, m, Ar**H**), 7.41-7.37 (4H, m, Ar**H**), 7.34-7.31 (1H, m, Ar**H**), 4.89 (2H, s,

C**H**$_2$Ph), 3.91 (2H, d, *J* = 2.5 Hz, C**H**$_2$C≡), 2.27 (1H, t, *J* = 2.5 Hz, ≡C**H**); $^{13}$C NMR (101 MHz, $CDCl_3$) *major rotamer*: δ 190.8 (C), 166.9 (C), 135.1 (CH), 134.2 (C), 133.1 (C), 130.0 (CH), 129.8 (2 × CH), 129.2 (2 × CH), 128.9 (2 × CH), 128.4 (2 × CH), 77.4 (C), 73.1 (CH), 50.3 ($CH_2$), 32.2 ($CH_2$); *minor rotamer*: δ 190.7 (C), 167.0 (C), 135.2 (C), 135.0 (CH), 133.0 (C), 130.7 (CH), 129.1 (2 × CH), 129.0 (CH), 128.9 (CH), 128.7 (2 × CH), 128.5 (CH), 128.2 (CH), 77.0 (C), 74.1 (CH), 46.8 ($CH_2$), 36.3 ($CH_2$); HRMS (ESI) Exact mass calculated for $[C_{18}H_{16}NO_2]^+$ $[M+H]^+$: 278.1176, found: 278.1176.

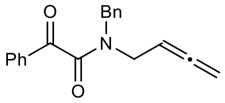

***N*-Benzyl-*N*-(buta-2,3-dien-1-yl)-2-oxo-2-phenylacetamide (1f).** To a solution of alkyne **S7** (693 mg, 2.50 mmol) in 1,4-dioxane (12.5 mL) at room temperature under inert atmosphere was added paraformaldehyde (375 mg, 12.5 mmol), CuBr (287 mg, 2.00 mmol), and diisopropylamine (0.70 mL, 5.0 mmol). The reaction was heated at 90 °C for 1 h, cooled to room temperature, filtered through a pad of celite using EtOAc as eluent, and concentrated *in vacuo*. Purification of the residue by column chromatography (20% EtOAc/petroleum ether) gave *allene* **1f** (348 mg, 48%) as a 1.3:1 mixture of rotamers as a colorless oil. $R_f$ = 0.52 (30% EtOAc/petroleum ether); IR 3063, 2929, 1955 (C=C=C), 1677, 1637 (C=O), 1595, 1446, 1360, 1316, 1259, 1202, 1175, 950, 850, 722, 699, 613, 519, 459 $cm^{-1}$; $^1$H NMR (400 MHz, $CDCl_3$) *major rotamer*: δ 8.02–7.97 (2H, m, Ar**H**), 7.67–7.62 (2H, m, Ar**H**), 7.53–7.49 (2H, m, Ar**H**), 7.39–7.25 (4H, m, Ar**H**), 5.04 (1H, quin, *J* = 6.6 Hz, $CH_2$C**H**=), 4.77 (2H, s, C**H**$_2$Ph), 4.74 (2H, dt, *J* = 6.6, 2.7 Hz, =C**H**$_2$), 3.74 (2H, dt, *J* = 6.6, 2.7 Hz, C**H**$_2$CH=); *minor rotamer*: δ 8.02-7.97 (2H, m, Ar**H**), 7.53–7.49 (2H, m, Ar**H**), 7.39–7.25 (6H, m, Ar**H**), 5.23 (1H, quin, *J* = 6.5 Hz, $CH_2$C**H**=), 4.87 (2H, dt, *J* = 6.6, 2.8 Hz, =C**H**$_2$), 4.42 (2H, s, C**H**$_2$Ph), 4.04 (2H, dt, *J* = 6.5, 2.8 Hz, C**H**$_2$CH=); $^{13}$C NMR (101 MHz, $CDCl_3$) *major rotamer*: δ 209.6 (C), 191.2 (C), 167.3 (C), 135.0 (C), 134.8 (CH), 133.3 (C), 129.9 (2 × CH), 129.1 (2 × CH), 129.0 (2 × CH), 128.8 (2 × CH), 128.3 (CH), 86.1 (CH), 77.2 ($CH_2$), 46.9 ($CH_2$), 45.7 ($CH_2$); *observable signals of minor rotamer*: δ 209.7 (C), 191.4 (C), 167.2 (C), 136.1 (C), 135.0 (CH), 129.9 (2 × CH), 129.1 (2 × CH), 128.9 (2 × CH), 128.3 (2 × CH), 128.0 (CH), 110.1 (C), 85.6 (CH), 77.4 ($CH_2$), 50.7 ($CH_2$), 41.8 ($CH_2$); HRMS (ESI) Exact mass calculated for $[C_{19}H_{18}NO_2]^+$ $[M+H]^+$: 292.1332, found: 292.1333.

**Preparation of Tethered Allene–Ketone 1g**

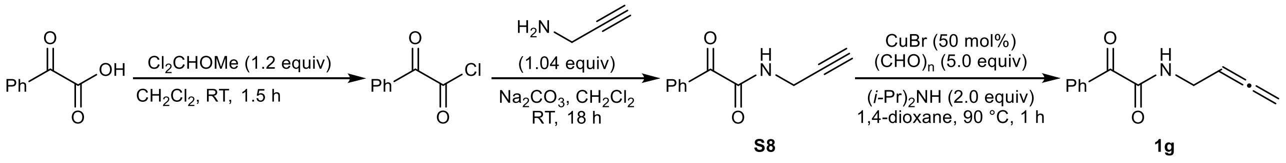


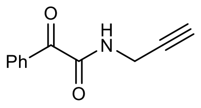

**2-Oxo-2-phenyl-N-(prop-2-yn-1-yl)acetamide (S8).** To a solution of phenylglyoxylic acid (450 mg, 3.00 mmol) in $CH_2Cl_2$ (3 mL) under inert atmosphere was added dichloromethyl methyl ether (0.33 mL, 3.60 mmol) dropwise. The mixture was stirred at room temperature for 1.5 h and then diluted with $CH_2Cl_2$ (6 mL). $Na_2CO_3$ (2.11 g, 20.0 mmol) was added, followed by a solution of propargylamine (0.20 mL, 3.12 mmol) in $CH_2Cl_2$ (1 mL). The mixture was stirred at room temperature for 18 h, quenched with $H_2O$ (10 mL), and extracted with $CH_2Cl_2$ (2 × 10 mL). The combined organic layers were dried ($Na_2SO_4$), filtered, and concentrated *in vacuo*. Purification of the residue by column chromatography (15% EtOAc/petroleum ether) gave *alkyne* **S8** (475 mg, 85%) as a white solid. $R_f$ = 0.23 (15% EtOAc/petroleum ether); m.p. 87–88 °C ($Et_2O$); IR 3274 (NH), 1678 (C=O), 1643, 1593, 1550, 1216, 1176, 675, 621, 559, 464 $cm^{-1}$; $^1H$ NMR (400 MHz, $CDCl_3$) δ 8.35 (2H, d, *J* = 7.6 Hz, Ar**H**), 7.64 (1H, t, *J* = 7.4 Hz, Ar**H**), 7.49 (2H, t, *J* = 7.7 Hz, Ar**H**), 7.28 (1H, br s, N**H**), 4.19 (2H, dd, *J* = 5.4, 2.4 Hz, NC**H**$_2$), 2.30 (1H, t, *J* = 2.2 Hz, ≡C**H**); $^{13}C$ NMR (101 MHz, $CDCl_3$) δ 186.9 (C), 161.3 (C), 134.8 (CH), 133.2 (C), 131.4 (2 × CH), 128.7 (2 × CH), 78.5 (C), 72.5 (CH), 29.4 ($CH_2$); HRMS (ESI) Exact mass calcd for $[C_{11}H_9NO_2Na]^+$ $[M + Na]^+$: 210.0525, found: 210.0529.

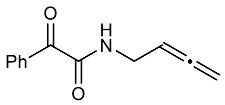

***N*-(Buta-2,3-dien-1-yl)-2-oxo-2-phenylacetamide (1g).** To a solution of alkyne **S8** (407 mg, 2.20 mmol) in 1,4-dioxane (11 mL) at room temperature under inert atmosphere was added paraformaldehyde (327 mg, 10.9 mmol), CuBr (158 mg, 1.10 mmol), and diisopropylamine (0.62 mL, 4.40 mmol). The reaction was heated at 90 °C for 1 h, cooled to room temperature, filtered through a pad of celite using EtOAc as eluent, and concentrated *in vacuo*. Purification of the residue by column chromatography (30% EtOAc/petroleum ether) gave *allene* **1g** (112 mg, 25%) as a pale yellow amorphous solid. $R_f$ = 0.47 (30% EtOAc/petroleum ether); IR 3375 (NH), 1960, 1655 (C=O), 1514, 1449, 1277, 1201, 1178, 864, 746, 690, 615, 494 $cm^{-1}$; $^1H$ NMR (400 MHz, $CDCl_3$) δ 8.37–8.31 (2H, m, Ar**H**), 7.66–7.59 (1H, m, Ar**H**), 7.48 (2H, t, *J* = 7.7 Hz, Ar**H**), 7.22 (1H, br s, N**H**), 5.28 (1H, quin, *J* = 6.4 Hz, $CH_2$C**H**=), 4.90 (2H, dt, *J* = 6.5, 3.2 Hz, =C**H**$_2$), 3.99 (2H, app tt, *J* = 6.0, 3.2 Hz, NC**H**$_2$); $^{13}C$ NMR (101 MHz, $CDCl_3$) δ 208.4 (C), 187.6 (C), 161.6 (C), 134.6 (CH), 133.4 (C), 131.3 (2 × CH), 128.6 (2 × CH), 87.2 (CH), 78.2 ($CH_2$), 37.6 ($CH_2$); HRMS (ESI) Exact mass calcd for $[C_{12}H_{12}NO_2]^+$ $[M+H]^+$: 202.0863, found: 202.0866.

**$N$-(Buta-2,3-dien-1-yl)-4-methyl-$N$-(2-oxo-2-phenylethyl)benzenesulfonamide (4a)[4]**

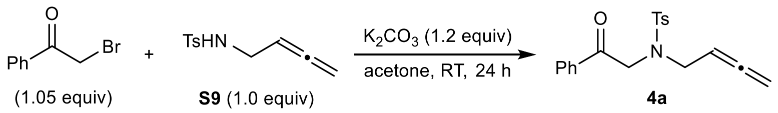

To a solution of sulfonamide **S9**[5] (400 mg, 1.79 mmol) in acetone (10 mL) was added $K_2CO_3$ (297 mg, 2.15 mmol) followed by 2-bromoacetophenone (374 mg, 1.88 mmol), and the resulting suspension was stirred at room temperature for 24 h. The reaction was quenched with $H_2O$ (10 mL) and the acetone was removed under reduced pressure. The resulting aqueous phase was extracted with EtOAc (3 × 10 mL) and the combined organic layers were washed with brine (10 mL), dried ($MgSO_4$), filtered, and concentrated *in vacuo*. Purification of the residue by column chromatography (5% EtOAc/petroleum ether to 20% EtOAc/petroleum ether) gave *allene* **4a** (470 mg, 78%) as a white solid that displayed spectroscopic data consistent with those reported previously.[4] $R_f$ = 0.82 (20% EtOAc/petroleum ether); m.p. 69–72 °C ($Et_2O$); IR 2961, 1956 (C=C=C), 1733, 1686 (C=O), 1596, 1450, 1321, 1296, 1193, 1173, 991, 847, 685, 538 $cm^{-1}$; $^1H$ NMR (500 MHz, $CDCl_3$) δ 7.96–7.91 (2H, m, Ar**H**), 7.76 (2H, d, $J$ = 8.3 Hz, Ar**H**), 7.62–7.58 (1H, m, Ar**H**), 7.50–7.47 (2H, m, Ar**H**), 7.31 (2H, d, $J$ = 8.1 Hz, Ar**H**), 4.99 (1H, quin, $J$ = 6.9 Hz, $CH_2C$**H**=), 4.77 (2H, s, O=CC**H**$_2$), 4.63 (2H, dt, $J$ = 6.6, 2.4 Hz, =C**H**$_2$), 3.94 (2H, dt, $J$ = 7.3, 2.5 Hz, NC**H**$_2$CH), 2.43 (3H, s, C**H**$_3$); $^{13}C$ NMR (126 MHz, $CDCl_3$) δ 209.9 (C), 194.0 (C), 143.6 (C), 137.0 (C), 135.2 (C), 133.9 (CH), 129.8 (2 × CH), 129.0 (2 × CH), 128.1 (2 × CH), 127.6 (2 × CH), 85.8 (CH), 76.4 ($CH_2$), 52.1 ($CH_2$), 47.4 ($CH_2$), 21.7 ($CH_3$); HRMS (ESI) Exact mass calculated for $[C_{19}H_{20}NO_3S]^+$ $[M+H]^+$: 342.1158, found: 342.1169.

**$N$-(Buta-2,3-dien-1-yl)-4-methyl-$N$-(2-oxopropyl)benzenesulfonamide (4b)[6]**

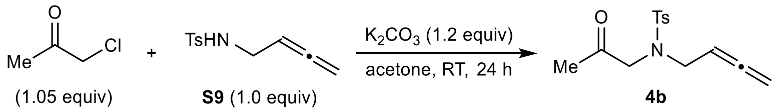

To a solution of sulfonamide **S9**[5] (558 mg, 2.50 mmol) in acetone (10 mL) was added $K_2CO_3$ (691 mg, 5.00 mmol) followed by chloroacetone (209 µL, 2.63 mmol), and the resulting suspension was stirred at room temperature for 24 h. The reaction was quenched with $H_2O$ (10 mL) and the acetone was removed under reduced pressure. The resulting aqueous phase was extracted with EtOAc (3 × 10 mL) and the combined organic layers were washed with brine (10 mL), dried ($MgSO_4$), filtered, and concentrated *in vacuo*. Purification of the residue by column chromatography (5% EtOAc/petroleum ether to 20% EtOAc/petroleum ether) gave *allene* **4b** (542 mg, 78%) as a colorless oil that displayed spectroscopic data consistent with those reported previously.[6] $R_f$ = 0.32 (20% EtOAc/petroleum ether); IR 2924, 2928, 2967, 1955 (C=C=C), 1733 (C=O), 1597, 1416, 1153, 1098, 986, 851, 760, 658, 545, 527 $cm^{-1}$; $^1H$ NMR (500 MHz, $CDCl_3$) δ 7.70 (2H, d, $J$ = 8.3 Hz, Ar**H**), 7.31 (2H, d, $J$ = 8.1 Hz, Ar**H**), 4.97 (1H, q, $J$ = 7.0 Hz, $CH_2C$**H**=), 4.71 (2H, dt, $J$ = 6.6, 2.4 Hz, =C**H**$_2$),

3.93 (2H, s, O=CC**H**$_2$), 3.83 (2H, dt, *J* = 7.4, 2.4 Hz, NC**H**$_2$CH), 2.43 (3H, s, C**H**$_3$C=O), 2.21 (3H, s, ArC**H**$_3$); $^{13}$C NMR (126 MHz, $CDCl_3$) δ 210.1 (C), 204.3 (C), 143.9 (C), 136.3 (C), 129.9 (2 × CH), 127.6 (2 × CH), 85.5 (CH), 76.6 ($CH_2$), 56.0 ($CH_2$), 48.2 ($CH_2$), 27.2 ($CH_3$), 21.7 ($CH_3$); HRMS (ESI) Exact mass calculated for $[C_{14}H_{17}NaNO_3S]^+$ $[M+Na]^+$: 302.0821, found: 302.0822.

**2-[Buta-2,3-dien-1-yl(4-methoxyphenyl)amino]-1-phenylethan-1-one (4c)**

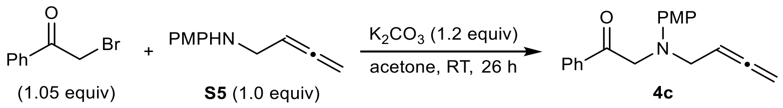


To a solution of allenylamine **S5** (351 mg, 2.00 mmol) in acetone (10 mL) was added $K_2CO_3$ (553 mg, 4.00 mmol) followed by bromoacetophenone (478 mg, 2.40 mmol), and the resulting suspension was stirred at room temperature for 26 h. The reaction was quenched with $H_2O$ (10 mL) and the acetone was removed under reduced pressure. The resulting aqueous phase was extracted with EtOAc (3 × 10 mL) and the combined organic layers were washed with brine (10 mL), dried ($Na_2SO_4$), filtered, and concentrated *in vacuo*. Purification of the residue by column chromatography (10% EtOAc/petroleum ether) gave *allene* **4c** (469 mg, 80%) as a colorless oil. $R_f$ = 0.41 (10% EtOAc/petroleum ether); IR 2930, 2827, 1954 (C=C=C), 1693 (C=O), 1515, 1263, 1219, 1177, 1033, 966, 846, 805, 757, 721, 692, 519, 504 cm$^{-1}$; $^1$H NMR (400 MHz, $CDCl_3$) δ 8.02–8.00 (2H, m, Ar**H**), 7.63–7.58 (1H, m, Ar**H**), 7.51–7.47 (2H, m, Ar**H**), 6.82–6.78 (2H, m, Ar**H**), 6.69–6.65 (2H, m, Ar**H**), 5.24 (1H, quin, *J* = 6.5 Hz, $CH_2$C**H**=), 4.74 (2H, dt, *J* = 6.8, 2.8 Hz, =C**H**$_2$), 4.71 (2H, s, O=CC**H**$_2$), 4.03 (2H, dt, *J* = 6.1, 2.8 Hz, NC**H**$_2$CH), 3.74 (3H, s, OC**H**$_3$); $^{13}$C NMR (101 MHz, $CDCl_3$) δ 209.2 (C), 196.9 (C), 152.4 (C), 143.0 (C), 135.7 (C), 133.6 (CH), 128.9 (2 × CH), 128.0 (2 × CH), 115.2 (2 × CH), 114.9 (2 × CH), 87.2 (CH), 76.1 ($CH_2$), 57.7 ($CH_2$), 55.8 ($CH_3$), 51.6 ($CH_2$); HRMS (ESI) Exact mass calculated for $[C_{19}H_{20}NO_2]^+$ $[M+H]^+$: 294.1489, found: 294.1495.

**1-[Buta-2,3-dien-1-yl(4-methoxyphenyl)amino]-3,3-dimethylbutan-2-one (4d)**

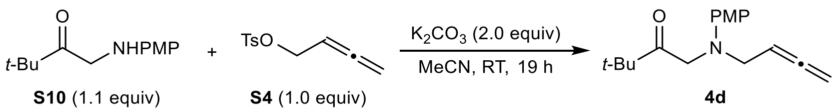


To a solution of aniline **S10**[7] (487 mg, 2.20 mmol) in MeCN (8 mL) was added $K_2CO_3$ (553 mg, 4.00 mmol) followed by allenyl tosylate **S4**[2] (448 mg, 2.00 mmol), and the resulting suspension was stirred at room temperature for 19 h. The reaction was partitioned between $Et_2O$ (10 mL) and saturated aqueous $NaHCO_3$ solution (10 mL). The aqueous layer was separated and extracted with $Et_2O$ (3 × 10 mL), and the combined organic layers were washed with saturated aqueous $NaHCO_3$ solution (2 × 10 mL), dried ($Na_2SO_4$), filtered, and concentrated *in vacuo*. Purification of the residue by column chromatography (20% EtOAc/petroleum ether) gave *allene* **4d** (333 mg, 61%) as a pale yellow oil.

$R_f$ = 0.47 (20% EtOAc/petroleum ether); IR 2968, 1954 (C=C=C), 1716, 1673 (C=O), 1511, 1463, 1364, 1243, 1178, 1032, 835, 812, 730, 549 $cm^{-1}$; $^{1}H$ NMR (400 MHz, $CDCl_3$) δ 6.79–6.77 (2H, m, Ar**H**), 6.60–6.58 (2H, m, Ar**H**), 5.17 (1H, quin, *J* = 6.6 Hz, $CH_2C$**H**=), 4.73 (2H, dt, *J* = 6.6, 2.8 Hz, =C**H**$_2$), 4.27 (2H, s, O=CC**H**$_2$), 3.90 (2H, dt, *J* = 6.6, 2.8 Hz, NC**H**$_2$CH), 3.73 (3H, s, OC**H**$_3$), 1.22 (9H, s, C(C**H**$_3$)$_3$); $^{13}C$ NMR (101 MHz, $CDCl_3$) δ 212.4 (C), 209.2 (C), 152.2 (C), 143.2 (C), 114.9 (2 × CH), 114.8 (2 × CH), 87.4 (CH), 75.9 ($CH_2$), 55.8 ($CH_3$), 55.5 ($CH_2$), 51.2 ($CH_2$), 43.5 (C), 26.7 (3 × $CH_3$); HRMS (ESI) Exact mass calculated for $[C_{17}H_{23}NNaO_2]^+$ $[M+Na]^+$: 296.1621, found: 296.1619.

**1-[Buta-2,3-dien-1-yl(4-chlorophenyl)amino]propan-2-one (4e)**

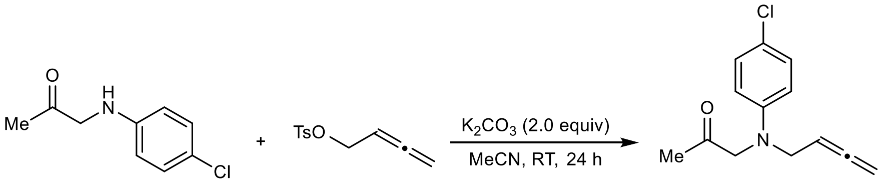

**S11** (1.1 equiv) **S4** (1.0 equiv) **4e**

To a solution of aniline **S11**[7] (302 mg, 1.65 mmol) in MeCN (6 mL) was added $K_2CO_3$ (415 mg, 3.00 mmol) followed by allenyl tosylate **S4**[2] (336 mg, 1.50 mmol), and the resulting suspension was stirred at room temperature for 24 h. The reaction was partitioned between $Et_2O$ (5 mL) and saturated aqueous $NaHCO_3$ solution (5 mL). The aqueous layer was separated and extracted with $Et_2O$ (3 × 5 mL), and the combined organic layers were washed with saturated aqueous $NaHCO_3$ solution (2 × 5 mL), dried ($Na_2SO_4$), filtered, and concentrated *in vacuo*. Purification of the residue by column chromatography (20% EtOAc/petroleum ether) gave *allene* **4e** (234 mg, 66%) as a yellow oil. $R_f$ = 0.36 (20% EtOAc/petroleum ether); IR 2961, 1954 (C=C=C), 1727 (C=O), 1596, 1497, 1352, 1226, 1160, 1097, 961, 847, 808, 655, 508 $cm^{-1}$; $^{1}H$ NMR (400 MHz, $CDCl_3$) δ 7.17–7.13 (2H, m, Ar**H**), 6.54–6.50 (2H, m, Ar**H**), 5.17 (1H, quin, *J* = 6.6 Hz, $CH_2C$**H**=), 4.78 (2H, dt, *J* = 6.6, 2.9 Hz, =C**H**$_2$), 4.00-3.98 (4H, m, O=CC**H**$_2$ and NC**H**$_2$CH), 2.16 (3H, s, C**H**$_3$); $^{13}C$ NMR (101 MHz, $CDCl_3$) δ 209.2 (C), 207.7 (C), 146.6 (C), 129.2 (2 × CH), 122.6 (C), 113.9 (2 × CH), 86.4 (CH), 76.9 ($CH_2$), 61.4 ($CH_2$), 51.1 ($CH_2$), 27.2 ($CH_3$); HRMS (ESI) Exact mass calculated for $[C_{13}H_{15}ClNO]^+$ $[M+H]^+$: 236.0837, found: 236.0836.

**Dimethyl 2-(buta-2,3-dien-1-yl)-2-(2-oxo-2-phenylethyl)malonate (4f)**

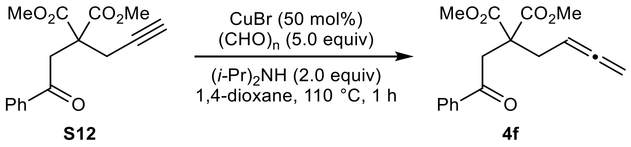


To a stirred solution of alkyne **S12**[8] (1.00 g, 3.50 mmol) in 1,4-dioxane (20 mL) at room temperature under inert atmosphere was added paraformaldehyde (530 mg, 17.7 mmol), CuBr (251 mg, 1.75 mmol), and diisopropylamine (0.98 mL, 12.0 mmol) The reaction was heated at 110 °C for 1 h, cooled to room temperature, diluted with EtOAc, filtered through a pad of silica using EtOAc (200 mL) as eluent, and concentrated *in vacuo*. Purification of the residue by column chromatography (5% EtOAc/petroleum ether to 10% EtOAc/petroleum ether) gave *allene* **4f** (200 mg, 18%) as a colorless oil. $R_f$ = 0.31 (10% EtOAc/petroleum ether); IR 2953, 1954 (C=C=C), 1732 (C=O), 1684 (C=O), 1596, 1434, 1356, 1283, 1199, 1068, 1002, 848, 749, 689, 554 $cm^{-1}$; $^1H$ NMR (400 MHz, $CDCl_3$) δ 8.02–7.91 (2H, m, Ar**H**), 7.61–7.55 (1H, m, Ar**H**), 7.51–7.43 (2H, m, Ar**H**), 4.97 (1H, tt, *J* = 8.1, 6.6 Hz, $CH_2C\mathbf{H}$=), 4.51 (2H, dt, *J* = 6.6, 2.4 Hz, =$C\mathbf{H}_2$), 3.76 (6H, s, 2 × $CO_2C\mathbf{H}_3$), 3.75 (2H, s, O=$CC\mathbf{H}_2$), 2.83 (2H, dt, *J* = 8.1, 2.4 Hz, $C\mathbf{H}_2C$=); $^{13}C$ NMR (101 MHz, $CDCl_3$) δ 210.2 (C), 196.8 (C), 171.0 (2 × C), 136.7 (C), 133.6 (CH), 128.8 (2 × CH), 128.2 (2 × CH), 84.8 (CH), 74.9 ($CH_2$), 55.7 (C), 53.0 (2 × $CH_3$), 41.4 ($CH_2$), 32.9 ($CH_2$); HRMS (ESI) Exact mass calculated for $[C_{17}H_{18}NaO_5]^+$ $[M+Na]^+$: 325.1046, found: 325.1051.

***N*-(Buta-2,3-dien-1-yl)-4-methyl-*N*-(3-oxo-3-phenylpropyl)benzenesulfonamide (4g)**

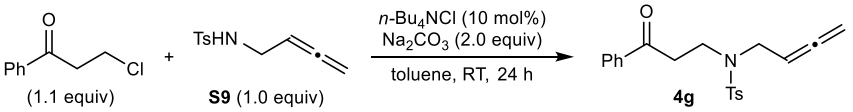


To a suspension of sulfonamide **S9**[5] (446 mg, 2.00 mmol), *n*-$Bu_4NCl$ (59 mg, 0.21 mmol), and $Na_2CO_3$ (424 mg, 4.00 mmol) in toluene (16 mL) at 0 °C and was added 3-chloropropiophenone (371 mg, 2.20 mmol) portionwise and the mixture was stirred at room temperature for 24 h. The reaction was quenched with saturated aqueous $NH_4Cl$ solution (20 mL) and extracted with EtOAc (3 × 10 mL). The combined organic layers were washed with brine (2 × 10 mL), dried ($Na_2SO_4$), filtered, and concentrated *in vacuo*. Purification of the residue by column chromatography (5% EtOAc/petroleum ether to 20% EtOAc/petroleum ether) gave *allene* **4g** (618 mg, 87%) as a white solid. $R_f$ = 0.59 (20% EtOAc/petroleum ether); m.p 73–75 °C ($Et_2O$); IR 2983, 1965 (C=C=C), 1680 (C=O), 1596, 1429, 1321, 1209, 1149, 1092, 995, 936, 861, 838, 808, 740, 685, 538, 509, 426 $cm^{-1}$; $^1H$ NMR (400 MHz, $CDCl_3$) δ 7.96–7.94 (2H, m, Ar**H**), 7.73–7.70 (2H, m, Ar**H**), 7.61–7.56 (1H, m, Ar**H**), 7.50-7.45 (2H, m, Ar**H**), 7.32–7.28 (2H, m, Ar**H**), 4.97 (1H, quin, *J* = 6.9 Hz, $CH_2C\mathbf{H}$=), 4.70 (2H, dt, *J* = 6.6, 2.5 Hz, =$C\mathbf{H}_2$), 3.90 (2H, dt, *J* = 7.1, 2.5 Hz, $NC\mathbf{H}_2CH$), 3.59–3.56 (2H, m, $CH_2C\mathbf{H}_2N$), 3.41–3.37 (2H,

m, O=CC**H**$_2$), 2.42 (3H, s, ArC**H**$_3$); $^{13}$C NMR (101 MHz, $CDCl_3$) δ 209.6 (C), 198.5 (C), 143.6 (C), 136.7 (C), 136.6 (C), 133.6 (CH), 129.9 (2 × CH), 128.8 (2 × CH), 128.2 (2 × CH), 127.4 (2 ×CH), 86.3 (CH), 76.7 ($CH_2$), 48.3 ($CH_2$), 43.2 ($CH_2$), 39.0 ($CH_2$), 21.7 ($CH_3$); HRMS (ESI) Exact mass calculated for $[C_{20}H_{22}NO_3S]^+$ $[M+H]^+$: 356.1315, found: 356.1316.

### *N*-(Buta-2,3-dien-1-yl)-4-methyl-*N*-(3-oxobutyl)benzenesulfonamide (4h)

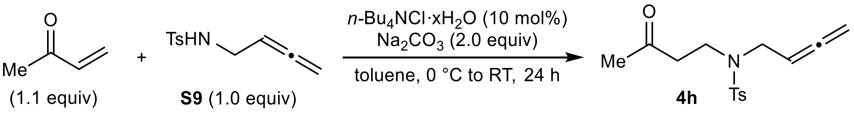


To a suspension of sulfonamide **S9**[5] (1.12 g, 5.00 mmol), $Na_2CO_3$ (1.06 g, 10.0 mmol), and *n*-$Bu_4NCl\cdot xH_2O$ (148 mg, *ca.* 0.500 mmol) in toluene (150 mL) at 0 °C was added methyl vinyl ketone (458 μL, 5.50 mmol) dropwise and the mixture was stirred at room temperature for 24 h. The reaction was quenched with saturated aqueous $NH_4Cl$ solution (20 mL) and extracted with EtOAc (20 mL). The combined organic layers were washed with brine (20 mL), dried ($MgSO_4$), filtered, and concentrated *in vacuo*. Purification of the residue by column chromatography (5% EtOAc/petroleum ether to 20% EtOAc/petroleum ether) gave *allene* **4h** (542 mg, 78%) as a colorless oil. $R_f$ = 0.24 (20% EtOAc/petroleum ether); IR 2924, 1954 (C=C=C), 1713 (C=O), 1598, 1336, 1154, 1097, 847, 815, 727, 656, 545 cm$^{-1}$; $^1$H NMR (400 MHz, $CDCl_3$) δ 7.68 (2H, d, *J* = 8.3 Hz, Ar**H**), 7.30 (2H, d, *J* = 8.0 Hz, Ar**H**), 4.90 (1H, quin, *J* = 6.9 Hz, $CH_2$C**H**=), 4.70 (2H, dt, *J* = 6.5, 2.5 Hz, =C**H**$_2$), 3.83 (2H, dt, *J* = 7.0, 2.5 Hz, NC**H**$_2$CH), 3.39–3.36 (2H, m, O=CC$H_2$C**H**$_2$), 2.86–2.82 (2H, m, O=CC**H**$_2$), 2.42 (3H, s, ArC**H**$_3$), 2.15 (3H, s, C**H**$_3$C=O); $^{13}$C NMR (101 MHz, $CDCl_3$) δ 209.6 (C), 207.0 (C), 143.6 (C), 136.6 (C), 129.9 (2 × CH), 127.4 (2 × CH), 86.1 (CH), 76.6 ($CH_2$), 48.0 ($CH_2$), 43.6 ($CH_2$), 42.4 ($CH_2$), 30.4 ($CH_3$), 21.7 ($CH_3$); HRMS (ESI) Exact mass calculated for $[C_{15}H_{20}NO_3S]^+$ $[M+H]^+$: 294.1158, found: 294.1163.

### Dimethyl 2-(buta-2,3-dien-1-yl)-2-(3-oxobutyl)malonate (4i)

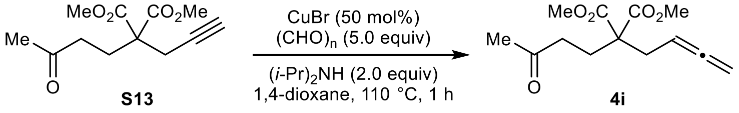


To a stirred solution of alkyne **S13**[9] (1.40 g, 6.00 mmol) in 1,4-dioxane (30 mL) at room temperature under inert atmosphere was added paraformaldehyde (900 mg, 30.0 mmol), CuBr (430 mg, 3.00 mmol), and diisopropylamine (1.7 mL, 12.0 mmol) The reaction was heated at 110 °C for 1 h, cooled to room temperature, diluted with EtOAc (50 mL), filtered through a pad of silica using EtOAc (200 mL) as eluent, and concentrated *in vacuo*. Purification of the residue by column chromatography (5% EtOAc/pentane to 20% EtOAc/pentane) gave *allene* **4i** (423 mg, 23%) as a colorless oil. $R_f$ = 0.25 (30% EtOAc/petroleum ether); IR 2954, 1955 (C=C=C), 1729 (C=O), 1435, 1372, 1198, 1093, 1044,

915, 848 $cm^{-1}$; $^{1}H$ NMR (500 MHz, $CDCl_3$) δ 4.95 (1H, tt, *J* = 8.0, 6.7 Hz, $CH_2C\mathbf{H}$=), 4.67 (2H, dt, *J* = 6.7, 2.4 Hz, =$C\mathbf{H_2}$), 3.72 (6H, s, 2 × $OC\mathbf{H_3}$), 2.60 (2H, dt, *J* = 8.0, 2.5 Hz, $C\mathbf{H_2}CH$=), 2.48–2.44 (2H, m, O=$CC\mathbf{H_2}$), 2.23–2.15 (2H, m, O=$CCH_2C\mathbf{H_2}$), 2.13 (3H, s, $C\mathbf{H_3}$C=O); $^{13}C$ NMR (126 MHz, $CDCl_3$) δ 210.2 (C), 207.3 (C), 171.4 (2 × C), 84.2 (CH), 74.9 ($CH_2$), 57.1 (C), 52.7 (2 × $CH_3$), 38.7 ($CH_2$), 33.2 ($CH_2$), 30.1 ($CH_3$), 26.6 ($CH_2$); HRMS (ESI) Exact mass calculated for $[C_{13}H_{19}NO_5]^+$ $[M+H]^+$: 255.1227, found: 255.1227.

**1-Phenylhepta-5,6-dien-1-one (4j)**

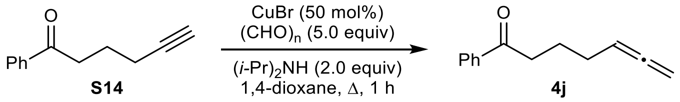


To a solution of alkyne **S14**[10] (517 mg, 3.00 mmol), in 1,4-dioxane (15 mL) at room temperature under inert atmosphere was added paraformaldehyde (455 mg, 15.0 mmol), CuBr (215 mg, 1.50 mmol), and diisopropylamine (0.8 mL, 6.0 mmol). The reaction was heated at reflux for 1 h, cooled to room temperature, diluted with EtOAc (20 mL), filtered through a short plug of silica using EtOAc as eluent, and concentrated *in vacuo*. Purification of the residue by column chromatography (10% EtOAc/petroleum ether) gave *allene* **4j** (309 mg, 55%) as a yellow oil. $R_f$ = 0.14 (10% $Et_2O$/petroleum ether); IR 2936, 1954 (C=C=C), 1681 (C=O), 1597, 1580, 1447, 1226, 1365, 1000, 841 $cm^{-1}$; $^{1}H$ NMR ($CDCl_3$, 400 MHz) δ 8.00–7.92 (2H, m, Ar**H**), 7.59–7.52 (1H, m, Ar**H**), 7.50–7.42 (2H, m, Ar**H**), 5.12 (1H, quin, *J* = 6.7 Hz, $CH_2C\mathbf{H}$=), 4.67 (2H, dt, *J* = 6.5, 3.2 Hz, =$C\mathbf{H_2}$), 3.02 (2H, t, *J* = 7.3 Hz, O=$CC\mathbf{H_2}$), 2.15–2.08 (2H, m, $C\mathbf{H_2}CH$=), 1.89 (2H, quin, *J* = 7.3 Hz, $CH_2C\mathbf{H_2}CH_2$); $^{13}C$ NMR ($CDCl_3$, 101 MHz) δ 208.8 (C), 200.3 (C), 137.2 (C), 133.1 (CH), 128.7 (2 × CH), 128.2 (2 × CH), 89.5 (CH), 75.2 ($CH_2$), 37.9 ($CH_2$), 27.9 ($CH_2$), 23.7 ($CH_2$); HRMS (ESI) Exact mass calculated for $[C_{13}H_{15}O]^+$ $[M+H]^+$: 187.1117, found: 187.1121.

**3,3-Dimethyl-1-phenylhepta-5,6-dien-1-one (4k)**

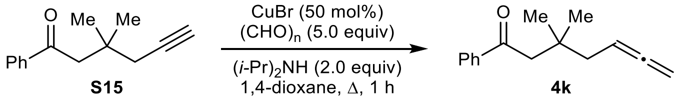


To a solution of alkyne **S15**[10] (801 mg, 4.00 mmol) in 1,4-dioxane (20 mL) at room temperature under inert atmosphere was added paraformaldehyde (606 mg, 20.0 mmol), CuBr (287 mg, 2.00 mmol), and diisopropylamine (1.1 mL, 8.0 mmol). The reaction was heated at reflux for 1 h, cooled to room temperature, diluted with EtOAc (20 mL), filtered through a short plug of silica using EtOAc as eluent, and concentrated *in vacuo*. Purification of the residue by column chromatography (5% EtOAc/pentane to 20% EtOAc/pentane) gave *allene* **4k** (174 mg, 20%) as a yellow oil. $R_f$ = 0.41 (10% EtOAc/petroleum ether); IR 2957, 1953 (C=C=C) 1672 (C=O), 1596, 1579, 1466, 1357, 1222,

1006, 747 cm$^{-1}$; $^{1}$H NMR ($CDCl_3$, 500 MHz) δ 7.96–7.89 (2H, m, Ar**H**), 7.62–7.50 (1H, m, Ar**H**), 7.45 (2H, dd, *J* = 8.4, 7.0 Hz, Ar**H**), 5.09 (1H, tt, *J* = 8.1, 6.6 Hz, $CH_2C$**H**=), 4.59 (2H, dt, *J* = 6.7, 2.4 Hz, =C**$H_2$**), 2.89 (2H, s, O=CC**$H_2$**), 2.14 (2H, dt, *J* = 8.1, 2.4 Hz, C**$H_2$**CH=), 1.07 (6H, s, 2 × C**$H_3$**); $^{13}$C NMR ($CDCl_3$, 126 MHz) δ 210.1 (C), 200.3 (C), 138.7 (C), 132.9 (CH), 128.6 (2 × CH), 128.3 (2 × CH), 86.2 (CH), 73.7 ($CH_2$), 47.6 ($CH_2$), 41.8 ($CH_2$), 34.9 (C), 27.5 (2 × $CH_3$); HRMS (ESI) Exact mass calculated for $[C_{15}H_{19}O]^+$ $[M+H]^+$: 215.1430, found: 215.1431.

## Enantioselective Nickel-Catalyzed Arylative and Alkenylative Intramolecular Allylations

### General Procedure

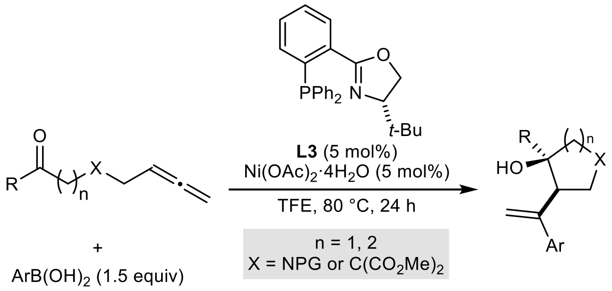


To an oven-dried microwave vial charged with a magnetic stirrer, $Ni(OAc)_2·4H_2O$ (3.7 mg, 15 μmol), (*S*)-$^{t}$BuPHOX (**L3**, 5.8 mg, 15 μmol), and boronic acid (0.450 mmol) were added. The vial was sealed and flushed with nitrogen or argon for 10 min. TFE (1.5 mL) was added, the solution was immerged in an oil bath pre-heated to 80 °C and stirred for 10 min. The allene (0.300 mmol) was added to a separate vial that was sealed and flushed with argon for 10 min. TFE (0.75 mL) was added to the allene and the resulting solution was added dropwise to the one containing the first vial containing the chiral nickel complex. The vial originally containing the substrate was rinsed with additional TFE (0.75 mL) and the rinsing solution was transferred to the first microwave vial *via* syringe. The reaction mixture was stirred at 80 °C for 24 h, cooled to room temperature, diluted with EtOAc (5 mL), filtered through a short pad of silica (3 cm height × 2 cm wide) using EtOAc (20 mL) as eluent, and concentrated *in vacuo*. If necessary, the crude mixture was purified by column chromatography to give the title compound.

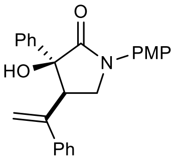


**(3*S*,4*R*)-3-Hydroxy-1-(4-methoxyphenyl)-3-phenyl-4-(1-phenylvinyl)pyrrolidin-2-one (2a).** The General Procedure was followed using allene **1a** (92.2 mg, 0.300 mmol) and phenylboronic acid (54.9 mg, 0.450 mmol). Filtration through a silica pad without purification by column chromatography gave the title compound (115 mg, 99%) as a white solid. $R_f$ = 0.26 (30% EtOAc/petroleum ether); m.p. 125-127 °C ($Et_2O$); IR 3417 (OH), 2954, 1682 (C=O), 1584, 1508, 1397, 1245, 1179, 1057, 828 cm$^{-1}$; $[\alpha]_D^{20}$ +16.0 (*c* 1.00, $CHCl_3$);

$^{1}$H NMR (400 MHz, $CDCl_3$) δ 7.61–7.55 (2H, m, Ar**H**), 7.41–7.37 (2H, m, Ar**H**), 7.36–7.20 (8H, m, Ar**H**), 6.98–6.92 (2H, m, Ar**H**), 5.51 (1H, s, =C**H**$_2$), 5.35 (1H, s, =C**H**$_2$), 4.03–3.92 (2H, m, C**H**$_2$N), 3.83 (3H, s, OC**H**$_3$), 3.79–3.73 (1H, m, C**H**$CH_2$), 3.20 (1H, s, O**H**); $^{13}$C NMR (101 MHz, $CDCl_3$) δ 173.1 (C), 157.3 (C), 146.1 (C), 142.1 (C), 141.9 (C), 131.9 (C), 128.6 (2 × CH), 128.3 (2 × CH), 128.1 (CH), 127.6 (CH), 126.9 (2 × CH), 125.6 (2 × CH), 121.9 (2 × CH), 115.9 ($CH_2$), 114.4 (2 × CH), 81.2 (C), 55.6 ($CH_3$), 51.1 ($CH_2$), 49.9 (CH); HRMS (ESI) Exact mass calculated for $[C_{25}H_{24}NO_3]^+$ $[M+H]^+$: 386.1751, found: 386.1756; Enantiomeric excess was determined by HPLC with a Chiralpak IC column (80:20 *iso*-hexane:*i*-PrOH, 1.0 mL/min, 254 nm, 25 °C) $t_r$ (minor) = 25.9 min, $t_r$ (major) = 33.3 min, 96% ee.

Slow diffusion of petroleum ether into a solution of **2a** in EtOAc gave crystals that were suitable for X-ray crystallography:

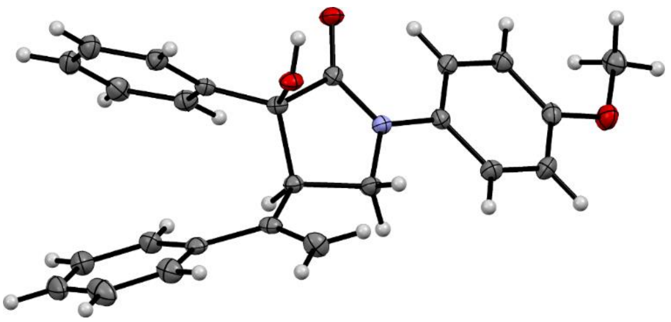

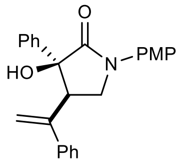


**2a**
CCDC 1945864
Flack parameter = 0.05(8)

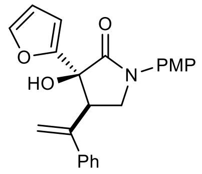


**(3*S*,4*R*)-3-(Furan-2-yl)-3-hydroxy-1-(4-methoxyphenyl)-4-(1-phenylvinyl)pyrrolidine-2-one (2b).** The General Procedure was followed using allene **1b** (89.2 mg, 0.300 mmol) and phenylboronic acid (54.9 mg, 0.450 mmol). Filtration through a silica pad without purification by column chromatography gave the title compound (112 mg, 99%) as a white amorphous solid. $R_f$ = 0.41 (40% EtOAc/petroleum ether); $[\alpha]_D^{25}$ +14.8 (*c* 0.27, $CHCl_3$); IR 3366 (OH), 2955, 1681 (C=O), 1509, 1440, 1399, 1298, 1245, 1150, 1029, 1005, 886, 828, 776, 735, 699, 595, 567, 522 cm$^{-1}$; $^{1}$H NMR (400 MHz, $CDCl_3$) δ 7.54–7.52 (2H, m, Ar**H**), 7.39–7.35 (3H, m, Ar**H**), 7.31–7.24 (3H, m, Ar**H**), 6.93–6.90 (2H, m, Ar**H**), 6.36 (1H, dd, *J* = 3.3, 0.9 Hz, Ar**H**), 6.29 (1H, dd, *J* = 3.3, 1.8 Hz, ArC**H**), 5.53 (1H, s, =C**H**$_2$), 5.31 (1H, s, =C**H**$_2$), 4.11–4.04 (2H, m, C**H**$_2$N), 3.94 (1H, dd, *J* = 8.7, 3.1 Hz, C**H**$CH_2$), 3.80 (3H, s, OC**H**$_3$), 3.54 (1H, s, O**H**); $^{13}$C NMR (101 MHz, $CDCl_3$) δ 170.8 (C), 157.2 (C), 153.5 (C), 145.7 (C), 142.8 (CH), 141.7 (C), 131.9 (C), 128.3 (2 × CH), 127.6 (CH), 126.7 (2 × CH), 121.8 (2 × CH), 115.5 ($CH_2$), 114.3 (2 × CH), 110.6 (CH), 107.8 (CH), 77.4 (C), 55.6 ($CH_3$), 51.3 ($CH_2$), 46.2 (CH); HRMS (ESI) Exact mass calculated for $[C_{23}H_{22}NO_4]^+$ $[M+H]^+$: 376.1543, found: 376.1542; Enantiomeric

excess was determined by HPLC with a Chiralpak AD-H column (60:40 *iso*-hexane:*i*-PrOH, 1.5 mL/min, 254 nm, 25 °C) $t_r$ (major) = 25.2 min, $t_r$ (minor) = 35.5 min, 97% ee.

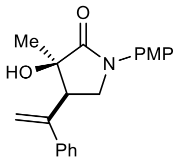


**(3*R*,4*R*)-3-Hydroxy-1-(4-methoxyphenyl)-3-methyl-4-(1-phenylvinyl)pyrrolidin-2-one (2c).** The General Procedure was followed using allene **1c** (73.6 mg, 0.300 mmol) and phenylboronic acid (54.9 mg, 0.450 mmol). Filtration through a silica pad without purification by column chromatography gave the title compound (96 mg, 99%) as a colorless solid. $R_f$ = 0.21 (30% EtOAc/petroleum ether); m.p. 122–125 °C ($Et_2O$); $[\alpha]_D^{25}$ +7.41 (*c* 0.54, $CHCl_3$); IR 3348 (OH), 2979, 1688 (C=O), 1510, 1469, 1391, 1288, 1244, 1175, 1097, 1030, 944, 889, 827, 774, 700, 596, 555, 524 $cm^{-1}$; $^1H$ NMR (400 MHz, $CDCl_3$) δ 7.53–7.49 (2H, m, Ar**H**), 7.43–7.40 (2H, m, Ar**H**), 7.37–7.26 (3H, m, Ar**H**), 6.92–6.88 (2H, m, Ar**H**), 5.48 (1H, s, =C**H**$_2$), 5.32 (1H, s, =C**H**$_2$), 3.92 (2H, app d, *J* = 6.4 Hz, C**H**$_2$N), 3.80 (3H, s, OC**H**$_3$), 3.44–3.40 (1H, m, C**H**CH$_2$), 3.00 (1H, s, O**H**), 1.41 (3H, s, C**H**$_3$C); $^{13}C$ NMR (101 MHz, $CDCl_3$) δ 174.0 (C), 156.9 (C), 145.9 (C), 142.4 (C), 132.1 (C), 128.4 (2 × CH), 127.7 (CH), 126.9 (2 × CH), 121.7 (2 × CH), 115.5 ($CH_2$), 114.2 (2 × CH), 76.4 (C), 55.5 ($CH_3$), 51.0 ($CH_2$), 48.1 (CH), 24.4 ($CH_3$); HRMS (ESI) Exact mass calculated for $[C_{20}H_{22}NO_3]^+$ $[M+H]^+$: 324.1594, found: 324.1597; Enantiomeric excess was determined by HPLC with a Chiralpak IC column (80:20 *iso*-hexane:*i*-PrOH, 1.0 mL/min, 254 nm, 25 °C) $t_r$ (major) = 26.7 min, $t_r$ (minor) = 31.3 min, 98% ee.

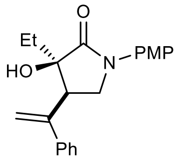


**(3*R*,4*R*)-3-Ethyl-3-hydroxy-1-(4-methoxyphenyl)-4-(1-phenylvinyl)pyrrolidin-2-one (2d).** The General Procedure was followed using allene **1d** (77.8 mg, 0.300 mmol) and phenylboronic acid (54.9 mg, 0.450 mmol). Filtration through a silica pad without purification by column chromatography gave the title compound (101 mg, >99%) as a colorless oil. $R_f$ = 0.10 (20% EtOAc/petroleum ether); $[\alpha]_D^{25}$ +18.6 (*c* 0.43, $CHCl_3$); IR 3550 (OH), 2964, 1682 (C=O), 1514, 1488, 1404, 1253, 1157, 1031, 1018, 909, 873, 823, 781, 746, 695, 575, 526, 463 $cm^{-1}$; $^1H$ NMR (400 MHz, $CDCl_3$) δ 7.54–7.50 (2H, m, Ar**H**), 7.44–7.41 (2H, m, Ar**H**), 7.36–7.26 (3H, m, Ar**H**), 6.93–6.89 (2H, m, Ar**H**), 5.45 (1H, s, =C**H**$_2$), 5.27 (1H, s, =C**H**$_2$), 4.00–3.95 (1H, m, C**H**$_2$N), 3.86 (1H, dd, *J* = 10.0, 4.0 Hz, C**H**$_2$N), 3.80 (3H, s, OC**H**$_3$), 3.52 (1H, dd, *J* = 7.0, 4.0 Hz, C**H**CH$_2$), 2.93 (1H, br m, O**H**), 1.85 (1H, dt, *J* = 14.8, 7.5 Hz, CH$_3$C**H**$_2$), 1.71 (1H, dt, *J* = 14.5, 7.4 Hz, CH$_3$C**H**$_2$), 1.00 (3H, t, *J* = 7.4 Hz, C**H**$_3$CH$_2$); $^{13}C$ NMR (101 MHz, $CDCl_3$) δ 173.8 (C), 156.9 (C), 147.0 (C), 142.4 (C), 132.1 (C), 128.3 (2 × CH), 127.7 (CH), 126.8 (2 × CH), 121.7 (2 × CH), 115.2 ($CH_2$), 114.2 (2 × CH), 79.4 (C), 55.5 ($CH_3$), 51.4 ($CH_2$), 45.0 (CH), 30.3 ($CH_2$), 8.3 ($CH_3$); HRMS (ESI) Exact mass calculated for $[C_{21}H_{24}NO_3]^+$ $[M+H]^+$: 338.1751, found:

338.1751; Enantiomeric excess was determined by HPLC with a Chiralpak AS-H column (90:10 *iso*-hexane:*i*-PrOH, 1.0 mL/min, 254 nm, 25 °C) $t_r$ (major) = 32.1 min, $t_r$ (minor) = 49.1 min, >99% ee.

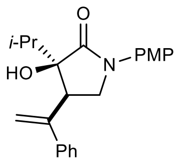


**(3*R*,4*R*)-3-Hydroxy-3-isopropyl-1-(4-methoxyphenyl)-4-(1-phenylvinyl)pyrrolidin-2-one (2e).** The General Procedure was followed using allene **1e** (82.0 mg, 0.300 mmol) and phenylboronic acid (54.9 mg, 0.450 mmol). Filtration through a silica pad without purification by column chromatography gave the title compound (105 mg, >99%) as a colorless oil. $R_f$ = 0.23 (20% EtOAc/petroleum ether); $[\alpha]^{25}_{D}$ +14.3 (*c* 0.84, $CHCl_3$); IR 3422 (OH), 2960, 1681 (C=O), 1510, 1465, 1440, 1401, 1291, 1245, 1178, 1159, 1031, 906, 828, 796, 729, 701, 566, 527 $cm^{-1}$; $^{1}$H NMR (400 MHz, $CDCl_3$) δ 7.53–7.49 (2H, m, Ar**H**), 7.44–7.41 (2H, m, Ar**H**), 7.36–7.26 (3H, m, Ar**H**), 6.94–6.89 (2H, m, Ar**H**), 5.43 (1H, s, =C**H**$_2$), 5.24 (1H, s, =C**H**$_2$), 4.00 (1H, dd, *J* = 10.3, 7.3 Hz, C**H**$_2$N), 3.80 (3H, s, OC**H**$_3$), 3.79–3.65 (1H, m, C**H**$_2$N), 3.60 (1H, dd, *J* = 7.3, 2.6 Hz, C**H**CH$_2$), 2.80 (1H, s, O**H**), 2.11 (1H, hept, *J* = 6.8 Hz, (CH$_3$)$_2$C**H**), 1.06 (3H, d, *J* = 6.9 Hz, (C**H**$_3$)$_2$CH), 1.01 (3H, d, *J* = 6.8 Hz, (C**H**$_3$)$_2$CH); $^{13}$C NMR (101 MHz, $CDCl_3$) δ 174.1 (C), 157.0 (C), 148.3 (C), 142.4 (C), 131.9 (C), 128.3 (2 × CH), 127.6 (CH), 127.0 (2 × CH), 121.8 (2 × CH), 115.3 ($CH_2$), 114.2 (2 × CH), 81.6 (C), 55.5 ($CH_3$), 52.3 ($CH_2$), 43.4 (CH), 35.0 (CH), 17.4 ($CH_3$), 16.7 ($CH_3$); HRMS (ESI) Exact mass calculated for $[C_{22}H_{26}NO_3]^+$ $[M+H]^+$: 352.1907, found: 352.1909; Enantiomeric excess was determined by HPLC with a Chiralpak IC column (90:10 *iso*-hexane:*i*-PrOH, 1.0 mL/min, 254 nm, 25 °C) $t_r$ (major) = 31.0 min, $t_r$ (minor) = 33.9 min, >99% ee.

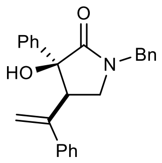


**(3*S*,4*R*)-1-Benzyl-3-hydroxy-3-phenyl-4-(1-phenylvinyl)pyrrolidin-2-one (2f).** *Using TFE as the solvent:* The General Procedure was followed using allene **1f** (87.4 mg, 0.300 mmol) and phenylboronic acid (54.9 mg, 0.450 mmol). Purification by passing the compound through a short pad of silica using pentane as eluent gave the title compound (110 mg, 99%) as a colorless solid. $R_f$ = 0.29 (30% EtOAc/petroleum ether); m.p. 158–162 °C ($Et_2O$); $[\alpha]^{25}_{D}$ –31.6 (*c* 0.38, $CHCl_3$); IR 3326 (OH), 3060, 2912, 1683 (C=O), 1483, 1436, 1340, 1258, 1024, 950, 895, 741, 695, 659, 589, 510, 465 $cm^{-1}$; $^{1}$H NMR (400 MHz, $CDCl_3$) δ 7.40–7.31 (5H, m, Ar**H**), 7.30–7.23 (5H, m, Ar**H**), 7.17–7.12 (3H, m, Ar**H**), 7.10–7.07 (2H, m, Ar**H**), 5.43 (1H, d, *J* = 0.3 Hz, =C**H**$_2$), 5.17 (1H, s, =C**H**$_2$), 4.70 (1H, d, *J* = 14.5 Hz, C**H**$_2$Ph), 4.51 (1H, d, *J* = 14.5 Hz, C**H**$_2$Ph), 3.61 (1H, td, *J* = 6.5, 0.9 Hz, C**H**CH$_2$), 3.47 (2H, app dd, *J* = 6.4, 1.2 Hz, CHC**H**$_2$), 2.88 (1H, s, O**H**); $^{13}$C NMR (101 MHz, $CDCl_3$) δ 174.2 (C), 145.1 (C), 142.0 (C), 141.8 (C), 135.8 (C), 128.9 (2 × CH), 128.5 (2 × CH), 128.2 (2 × CH), 128.0 (2 × CH), 127.9 (CH), 127.6 (CH), 127.3 (CH), 126.8 (2 × CH), 125.5 (2 × CH), 116.3 ($CH_2$), 80.2 (C), 50.5 (CH), 49.0 ($CH_2$),

47.3 ($CH_2$); HRMS (ESI) Exact mass calculated for $[C_{25}H_{24}NO_2]^+$ $[M+H]^+$: 370.1802, found: 370.1803; Enantiomeric excess was determined by HPLC with a Chiralpak AD-H column (90:10 *iso*-hexane:*i*-PrOH, 1.0 mL/min, 254 nm, 25 °C) $t_r$ (major) = 19.4 min, $t_r$ (minor) = 22.2 min, 87% ee.

*Using MeCN as the solvent*: A modification of the General Procedure was followed using allene **1f** (87.4 mg, 0.30 mmol) and phenylboronic acid (54.9 mg, 0.45 mmol) but using MeCN in place of TFE as the solvent. Purification by column chromatography (20% EtOAc/petroleum ether) gave the title compound (71.8 mg, 65%) as a colorless solid. $[\alpha]_D^{25}$ –34.8 (*c* 0.46, $CHCl_3$); Enantiomeric excess was determined by HPLC with a Chiralpak AD-H column (90:10 *iso*-hexane:*i*-PrOH, 1.0 mL/min, 254 nm, 25 °C); $t_r$ (major) = 19.8 min, $t_r$ (minor) = 22.8 min, 99% ee.

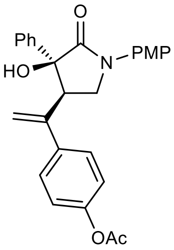


**4-{1-[(3*R*,4*S*)-4-Hydroxy-1-(4-methoxyphenyl)-5-oxo-4-phenylpyrrolidin-3-yl]vinyl}phenyl acetate (2g).** The General Procedure was followed using allene **1a** (92.2 mg, 0.300 mmol) and 4-acetoxyphenylboronic acid (81.0 mg, 0.450 mmol). Purification by column chromatography (5% EtOAc/pentane to 30% EtOAc/pentane) gave the title compound (133 mg, >99%) as a white solid. $R_f$ = 0.26 (30% EtOAc/petroleum ether); m.p. 57-59 °C ($Et_2O$); IR 3365 (OH), 2935, 1752 (C=O), 1686 (C=O), 1510, 1247, 1194, 908, 829, 697 $cm^{-1}$; $[\alpha]_D^{28}$ +16.0 (*c* 1.00, $CHCl_3$); $^1H$ NMR (400 MHz, $CDCl_3$) δ 7.62–7.53 (2H, m, Ar**H**), 7.42–7.34 (2H, m, Ar**H**), 7.34–7.21 (5H, m, Ar**H**), 6.98–6.88 (4H, m, Ar**H**), 5.48 (1H, s, =C**H**$_2$), 5.34 (1H, s, =C**H**$_2$), 3.99–3.92 (2H, m, C**H**$_2$N), 3.82 (3H, s, OC**H**$_3$), 3.70 (1H, dd, *J* = 6.5, 4.9 Hz, C**H**CH$_2$), 3.31 (1H, s, O**H**), 2.28 (2H, s, CC**H**$_3$); $^{13}C$ NMR (101 MHz, $CDCl_3$) δ 173.0 (C), 169.5 (C), 157.2 (C), 150.1 (C), 145.1 (C), 142.0 (C), 139.6 (C), 131.9 (C), 128.6 (2 × CH), 128.1 (CH), 128.0 (2 × CH), 125.6 (2 × CH), 121.9 (2 × CH), 121.3 (2 × CH), 116.2 ($CH_2$), 114.4 (2 × CH), 81.1 (C), 55.6 ($CH_3$), 51.1 ($CH_2$), 50.0 (CH), 21.2 ($CH_3$); HRMS (ESI) Exact mass calculated for $[C_{27}H_{25}NNaO_5]^+$ $[M+Na]^+$: 466.1625, found: 466.1619; Enantiomeric excess was determined by HPLC with a Chiralpak OD-H column (90:10 *iso*-hexane:EtOH, 1.0 mL/min, 210 nm, 25 °C) $t_r$ (minor) = 29.0 min, $t_r$ (major) = 37.0 min, 97% ee.

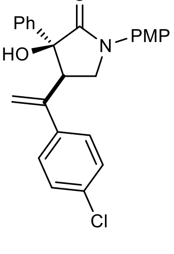


**(3*S*,4*R*)-4-[1-(4-Chlorophenyl)vinyl]-3-hydroxy-1-(4-methoxyphenyl)-3-phenylpyrrolidin-2-one (2h).** The General Procedure was followed using allene **1a** (92.2 mg, 0.300 mmol) and 4-chlorophenylboronic acid (70.4 mg, 0.450 mmol). Filtration through a silica pad without purification by column chromatography gave the title compound (125 mg, 99%) as a white solid. $R_f$ = 0.21 (30% EtOAc/petroleum ether); m.p. 54-56 °C ($Et_2O$); IR 3401 (OH), 2905, 1672 (C=O), 1511, 1489, 1395, 1247, 1102, 830,

516 $cm^{-1}$; $[\alpha]_D^{20}$ +12.0 (*c* 1.00, $CHCl_3$); $^1H$ NMR (400 MHz, $CDCl_3$) δ 7.58–7.54 (2H, m, Ar**H**), 7.34–7.26 (5H, m, Ar**H**), 7.18–7.13 (4H, m, Ar**H**), 6.95–6.91 (2H, m, Ar**H**), 5.45 (1H, s, =C**H**$_2$), 5.33 (1H, s, =C**H**$_2$), 3.95 (2H, d, *J* = 5.9 Hz, C**H**$_2$N), 3.82 (3H, s, OC**H**$_3$), 3.67 (1H, t, *J* = 5.9 Hz, C**H**$CH_2$), 3.60 (1H, s, O**H**); $^{13}C$ (101 MHz, $CDCl_3$) δ 173.1 (C), 157.2 (C), 144.6 (C), 141.9 (C), 140.4 (C), 133.3 (C), 131.8 (C), 128.5 (2 × CH), 128.3 (2 × CH), 128.2 (2 × CH), 128.0 (CH), 125.6 (2 × CH), 121.9 (2 × CH), 116.4 ($CH_2$), 114.3 (2 × CH), 81.1 (C), 55.6 ($CH_3$), 50.8 ($CH_2$), 50.0 (CH); HRMS (ESI) Exact mass calculated for $[C_{25}H_{23}ClNO_3]^+$ $[M+H]^+$: 420.1361, found: 420.1362; Enantiomeric excess was determined by HPLC with a Chiralpak IC column (80:20 *iso*-hexane:*i*-PrOH, 1.0 mL/min, 254 nm, 25 °C) $t_r$ (minor) = 22.1 min, $t_r$ (major) = 29.3 min, 98% ee.

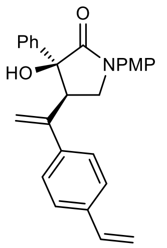


**(3*S*,4*R*)-3-Hydroxy-1-(4-methoxyphenyl)-3-phenyl-4-[1-(4-vinylphenyl)-vinyl]pyrrolidin-2-one (2i).** The General Procedure was followed using allene **1a** (92.2 mg, 0.300 mmol) and 4-vinylphenylboronic acid (66.6 mg, 0.450 mmol). Purification by column chromatography (5% EtOAc/pentane to 30% EtOAc/pentane) gave the title compound (87.6 mg, 71%) as a white solid. $R_f$ = 0.26 (30% EtOAc/petroleum ether); m.p. 117-119 °C ($Et_2O$); IR 3404 (OH), 2921, 1684 (C=O), 1508, 1397, 1244, 1179, 1027, 828, 752, 710 $cm^{-1}$; $[\alpha]_D^{21}$ +8.0 (*c* 1.00, $CHCl_3$); $^1H$ NMR (400 MHz, $CDCl_3$) δ 7.62–7.56 (2H, m, Ar**H**), 7.42–7.37 (2H, m, Ar**H**), 7.37–7.20 (7H, m, Ar**H**), 7.00–6.90 (2H, m, Ar**H**), 6.68 (1H, dd, *J* = 17.6, 10.9 Hz, C**H**=$CH_2$), 5.72 (1H, dd, *J* = 17.6, 0.9 Hz, CH=C**H**$_2$), 5.53 (1H, s, C=C**H**$_2$), 5.33 (1H, s, C=C**H**$_2$), 5.24 (1H, dd, *J* = 10.9, 0.9 Hz, CH=C**H**$_2$), 4.05–3.91 (2H, m, C**H**$_2$N), 3.83 (3H, s, OC**H**$_3$), 3.79–3.72 (1H, m, C**H**$CH_2$), 3.10 (1H, s, O**H**); $^{13}C$ NMR (101 MHz, $CDCl_3$) δ 173.1 (C), 157.3 (C), 145.7 (C), 142.1 (C), 141.4 (C), 136.9 (C), 136.4 (CH), 131.9 (C), 128.7 (2 × CH), 128.1 (CH), 127.0 (2 × CH), 126.2 (2 × CH), 125.6 (2 × CH), 121.9 (2 × CH), 115.5 ($CH_2$), 114.4 (2 × CH), 114.0 ($CH_2$), 81.2 (C), 55.7 ($CH_3$), 51.1 ($CH_2$), 49.6 (CH); HRMS (ESI) Exact mass calculated for $[C_{27}H_{26}NO_3]^+$ $[M+H]^+$: 412.1907, found: 412.1908; Enantiomeric excess was determined by HPLC with a Chiralpak IC column (80:20 *iso*-hexane:*i*-PrOH, 1.0 mL/min, 254 nm, 25 °C) $t_r$ (minor) = 26.5 min, $t_r$ (major) = 33.3 min, 99% ee.

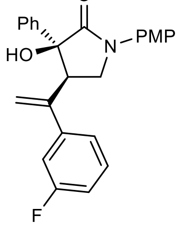


**(3*S*,4*R*)-4-[1-(4-Fluorophenyl)vinyl]-3-hydroxy-1-(4-methoxyphenyl)-3-phenylpyrrolidin-2-one (2j).** The General Procedure was followed using allene **1a** (92.2 mg, 0.300 mmol) and 3-fluorophenylboronic acid (63.0 mg, 0.450 mmol). Filtration through a silica pad without purification by column chromatography gave the title compound (121 mg, >99%) as a white solid. $R_f$ = 0.17 (30% EtOAc/petroleum ether); m.p. 120-121 °C ($Et_2O$); IR 3378 (OH), 2925, 1672 (C=O), 1580, 1512,

1247, 1064, 829, 695, 528 cm$^{-1}$; $[\alpha]_D^{22}$ +28.0 (*c* 1.00, $CHCl_3$); $^1$H NMR (400 MHz, $CDCl_3$) δ 7.64–7.54 (2H, m, Ar**H**), 7.40–7.34 (2H, m, Ar**H**), 7.34–7.25 (3H, m, Ar**H**), 7.19 (1H, td, *J* = 8.0, 6.0 Hz, Ar**H**), 7.04 (1H, ddd, *J* = 7.8, 1.7, 1.0 Hz, Ar**H**), 7.00–6.88 (4H, m, Ar**H**), 5.51 (1H, s, =C**H**$_2$), 5.36 (1H, s, =C**H**$_2$), 4.03–3.92 (2H, m, C**H**$_2$N), 3.83 (3H, s, OC**H**$_3$), 3.73–3.67 (1H, m, C**H**CH$_2$), 3.21 (1H, s, O**H**); $^{13}$C (101 MHz, $CDCl_3$) δ 173.0 (C), 162.7 (d, $J_{C-F}$ = 245.6 Hz, C), 157.3 (C), 145.0 (d, $J_{C-F}$ = 2.1 Hz, C), 144.3 (d, $J_{C-F}$ = 7.6 Hz, C), 141.9 (C), 131.9 (C), 129.7 (d, $J_{C-F}$ = 8.3 Hz, CH), 128.7 (2 × CH), 128.2 (CH), 125.5 (2 × CH), 122.6 (d, $J_{C-F}$ = 2.8 Hz, CH), 121.8 (2 × CH), 116.6 ($CH_2$), 114.41 (2 × CH), 114.38 (d, $J_{C-F}$ = 21.1 Hz, CH), 114.0 (d, $J_{C-F}$ = 22.0 Hz, CH), 81.2 (C), 55.7 ($CH_3$), 51.0 ($CH_2$), 49.8 (CH); HRMS (ESI) Exact mass calculated for $[C_{25}H_{22}FNNaO_3]^+$ $[M+Na]^+$: 426.1476, found: 426.1479; Enantiomeric excess was determined by HPLC with a Chiralpak IC column (80:20 *iso*-hexane:*i*-PrOH), 1.0 mL/min, 254 nm, 25 °C) $t_r$ (minor) = 19.7 min, $t_r$ (major) = 25.1 min, 98% ee.

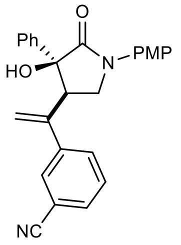


**3-{1-[(3*R*,4*S*)-4-Hydroxy-1-(4-methoxyphenyl)-5-oxo-4-phenylpyrrolidin-3-yl]vinyl}benzonitrile (2k).** The General Procedure was followed using allene **1a** (92.2 mg, 0.300 mmol) and 3-cyanophenylboronic acid (66.1 mg, 0.450 mmol). Purification by column chromatography (5% EtOAc/pentane to 50% EtOAc/pentane) gave the title compound (111 mg, 90%) as a white solid. $R_f$ = 0.18 (30% EtOAc/petroleum ether); m.p. 76-78 °C ($Et_2O$); IR 3349 (OH), 2931, 2228 (CN), 1679 (C=O), 1510, 1398, 1246, 1180, 829, 697 cm$^{-1}$; $[\alpha]_D^{20}$ +16.0 (*c* 1.00, $CHCl_3$); $^1$H NMR (400 MHz, $CDCl_3$) δ 7.59–7.53 (2H, m, Ar**H**), 7.47–7.43 (2H, m, Ar**H**), 7.43–7.40 (1H, m, Ar**H**), 7.32–7.25 (6H, m, Ar**H**), 6.97–6.91 (2H, m, Ar**H**), 5.47 (1H, s, =C**H**$_2$), 5.38 (1H, s, =C**H**$_2$), 3.97 (2H, d, *J* = 5.9 Hz, C**H**$_2$N), 3.81 (3H, s, OC**H**$_3$), 3.65 (1H, td, *J* = 5.9, 1.0 Hz, C**H**CH$_2$), 3.24 (1H, s, O**H**); $^{13}$C NMR (101 MHz, $CDCl_3$) δ 172.9 (C), 157.3 (C), 143.9 (C), 143.1 (C), 141.6 (C), 131.7 (C), 131.5 (CH), 130.9 (CH), 130.7 (CH) ,129.0 (CH), 128.6 (2 × CH), 128.3 (CH), 125.5 (2 × CH), 121.9 (2 × CH), 118.8 (CN), 117.9 ($CH_2$), 114.4 (2 × CH), 112.3 (C), 81.1 (C), 55.6 ($CH_3$), 50.6 ($CH_2$), 50.1 (CH); HRMS (ESI) Exact mass calculated for $[C_{26}H_{23}N_2O_3]^+$ $[M+H]^+$: 411.1703, found: 411.1707; Enantiomeric excess was determined by HPLC with a Chiralpak OD-H column (80:20 *iso*-hexane:EtOH, 1.0 mL/min, 254 nm, 25 °C) $t_r$ (minor) = 35.2 min, $t_r$ (major) = 50.2 min, 99% ee.

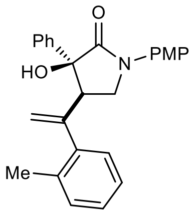


**(3*S*,4*R*)-3-Hydroxy-1-(4-methoxyphenyl)-3-phenyl-4-[1-(*o*-tolyl)vinyl]pyrrolidin-2-one (2l).** The General Procedure was followed using allene **1a** (92.2 mg, 0.300 mmol) and 2-methylphenylboronic acid (61.2 mg, 0.450 mmol). Purification by column chromatography (5% EtOAc/pentane to 30%

EtOAc/pentane) gave the title compound (83.2 mg, 69%) as a white solid. $R_f$ = 0.43 (30% EtOAc/petroleum ether); m.p. 147-149 °C ($CHCl_3$); IR 3373 (OH), 2914, 1673 (C=O), 1511, 1444, 1245, 1032, 962, 828, 616 $cm^{-1}$; $[\alpha]_D^{25}$ –10.0 (*c* 1.00, $CHCl_3$); $^1$H NMR (400 MHz, $CDCl_3$) δ 7.60–7.48 (2H, m, Ar**H**), 7.31–7.21 (5H, m, Ar**H**), 7.15–7.01 (4H, m, Ar**H**), 7.00–6.88 (2H, m, Ar**H**), 5.49 (1H, d, *J* =1.1 Hz, =C**H**$_2$), 5.21 (1H, d, *J* =1.0 Hz, =C**H**$_2$), 3.95–3.87 (2H, m, C**H**$_2$N), 3.82 (3H, s, OC**H**$_3$), 3.63–3.52 (1H, m, C**H**$CH_2$), 3.25 (1H, s, O**H**), 2.25 (3H, s, ArC**H**$_3$); $^{13}$C NMR (101 MHz, $CDCl_3$) δ 173.3 (C), 157.2 (C), 145.3 (C), 142.0 (C), 141.7 (C), 134.8 (C), 132.0 (C), 130.2 (CH), 128.9 (CH), 128.4 (2 × CH), 127.9 (CH), 127.2 (CH), 125.7 (2 × CH), 125.5 (CH), 122.0 (2 × CH), 117.8 ($CH_2$), 114.3 (2 × CH), 81.3 (C), 55.6 ($CH_3$), 51.1 (CH), 50.6 ($CH_2$), 20.2 ($CH_3$); HRMS (ESI) Exact mass calculated for $[C_{26}H_{26}NO_3]^+$ $[M+H]^+$: 400.1907, found: 400.1905; Enantiomeric excess was determined by HPLC with a Chiralpak IC column (90:10 *iso*-hexane:*i*-PrOH, 1.0 mL/min, 254 nm, 25 °C) $t_r$ (minor) = 39.3 min, $t_r$ (major) = 47.3 min, 84% ee.

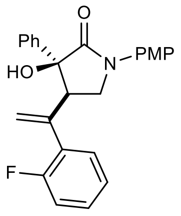


**(3*S*,4*R*)-4-[1-(2-Fluorophenyl)vinyl]-3-hydroxy-1-(4-methoxyphenyl)-3-phenylpyrrolidin-2-one (2m).** The General Procedure was followed using allene **1a** (92.2 mg, 0.300 mmol) and 2-fluorophenylboronic acid (63.0 mg, 0.450 mmol). Purification by column chromatography (5% EtOAc/pentane to 30% EtOAc/pentane) gave the title compound (82.3 mg, 68%) as a white solid. $R_f$ = 0.22 (30% EtOAc/petroleum ether); m.p. 167-170 °C ($Et_2O$); IR 3398 (OH), 2844, 1672 (C=O), 1513, 1486, 1303, 1245, 1230, 831, 758 $cm^{-1}$; $[\alpha]_D^{20}$ –12.0 (*c* 1.00, $CHCl_3$); $^1$H NMR (400 MHz, $CDCl_3$) δ 7.58–7.50 (2H, m, Ar**H**), 7.31–7.18 (6H, m Ar**H**), 7.16 (1H, dddd, *J* = 8.2, 7.2, 5.2, 1.9 Hz, Ar**H**), 7.03 (1H, td, *J* = 7.5, 1.2 Hz, Ar**H**), 6.96–6.90 (2H, m, Ar**H**), 6.84 (1H, ddd, *J* = 10.8, 8.2, 1.2 Hz, Ar**H**), 5.46 (1H, s, =C**H**$_2$), 5.38 (1H, s, =C**H**$_2$), 4.03 (1H, dd, *J* = 9.8, 5.5 Hz, C**H**$_2$N), 3.97 (1H, dd, 9.8, 6.6 Hz, C**H**$_2$N), 3.82 (3H, s, OC**H**$_3$), 3.77 (1H, ddd, *J*=6.6, 5.5, 1.0 Hz, C**H**$CH_2$), 3.09 (1H, s, O**H**); $^{13}$C NMR (101 MHz, $CDCl_3$) δ 173.1 (C), 159.5 (d, $J_{C\text{-}F}$ = 245.1 Hz, C), 157.2 (C), 141.3 (d, $J_{C\text{-}F}$ = 55.8 Hz, C), 132.0 (C), 131.0 (d, $J_{C\text{-}F}$ = 3.7 Hz, CH), 129.7 (d, $J_{C\text{-}F}$ = 14.0 Hz, C), 129.2 (d, $J_{C\text{-}F}$ = 8.6 Hz, CH), 128.4 (2 × CH), 127.9 (CH), 125.7 (2 × CH), 124.1 (d, *J* = 3.6 Hz, CH), 122.0 (2 × CH), 119.2 ($CH_2$), 115.4 (d, $J_{C\text{-}F}$ = 22.7 Hz, CH), 114.3 (2 × CH), 110.1 (C), 81.2 (C), 55.7 ($CH_3$), 50.4 (d, $J_{C\text{-}F}$ = 2.8 Hz, CH), 50.3 ($CH_2$); HRMS (ESI) Exact mass calculated for $[C_{25}H_{23}FNO_3]^+$ $[M+H]^+$: 404.1656, found: 404.1652; Enantiomeric excess was determined by HPLC with a Chiralpak IC column (85:15 *iso*-hexane:*i*-PrOH, 1.0 mL/min, 254 nm, 25 °C) $t_r$ (minor) = 27.5 min, $t_r$ (major) = 32.9 min, 97% ee.

**(3*S*,4*R*)-4-[1-(2-Aminophenyl)vinyl]-3-hydroxy-1-(4-methoxyphenyl)-3-phenylpyrrolidin-2-one (2n)**

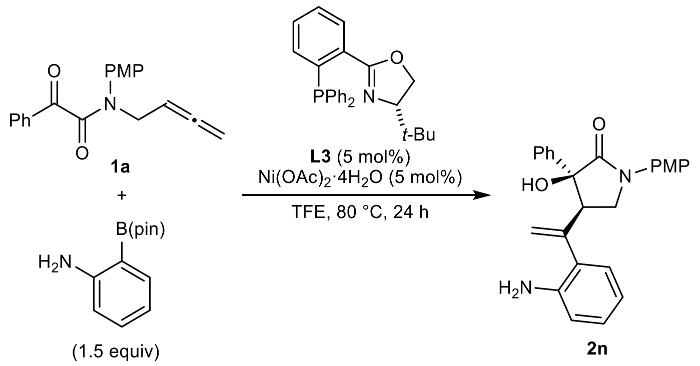


A modification of the General Procedure (in that the pinacol boronate was used instead of the boronic acid) was followed using allene **1a** (92.2 mg, 0.300 mmol) and 2-aminophenylboronic acid pinacol ester (98.6 mg, 0.450 mmol). Purification by column chromatography (20% EtOAc/pentane to 50% EtOAc/pentane) gave the title compound (57.2 mg, 48%) as a white solid. $R_f$ = 0.13 (30% EtOAc/petroleum ether); m.p. 68-70 °C ($CHCl_3$); IR 3364 (OH), 2927, 1657 (C=O), 1615, 1510, 1325, 1121, 1071, 928, 695 $cm^{-1}$; $[\alpha]_D^{24}$ –6.00 (*c* 1.00, $CHCl_3$); $^1H$ NMR (400 MHz, $CDCl_3$) δ 7.47 (2H, dd, *J* = 9.2, 2.7 Hz, Ar**H**), 7.40–7.34 (2H, m, Ar**H**), 7.32–7.21 (3H, m, Ar**H**), 7.01 (1H, td, *J* =7.7, 1.5 Hz, Ar**H**), 6.94–6.86 (3H, m, Ar**H**), 6.67–6.59 (2H, m, Ar**H**), 5.45 (1H, d, *J* =1.5 Hz, =C**H**$_2$), 5.30 (1H, d, *J* =1.2 Hz, =C**H**$_2$), 4.03 (3H, br s, N**H**$_2$ and O**H**), 3.97–3.84 (2H, m, C**H**$_2$N), 3.81 (3H, s, OC**H**$_3$), 3.70–3.63 (1H, m, C**H**$CH_2$); $^{13}C$ NMR (101 MHz, $CDCl_3$) δ 173.1 (C), 157.2 (C), 143.9 (C), 143.1 (C), 141.9 (C), 132.0 (C), 129.1 (CH), 128.54 (C), 128.51 (2 × CH), 128.46 (CH), 128.0 (CH), 125.7 (2 × CH), 122.1 (2 × CH), 118.8 ($CH_2$), 118.7 (CH), 116.4 (CH), 114.3 (2 × CH), 81.4 (C), 55.6 ($CH_3$), 51.5 (CH), 50.4 ($CH_2$); HRMS (ESI) Exact mass calculated for $[C_{25}H_{25}N_2O_3]^+$ $[M+H]^+$: 401.1860, found: 401.1862; Enantiomeric excess was determined by HPLC with a Chiralcel OD-H column (70:30 *iso*-hexane:*i*-PrOH, 1.5 mL/min, 254 nm, 25 °C) $t_r$ (major) = 12.6 min, $t_r$ (minor) = 18.3 min, 56% ee.

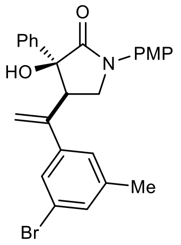


**(3*S*,4*R*)-4-[1-(3-Bromo-5-methylphenyl)vinyl]-3-hydroxy-1-(4-methoxy-phenyl)-3-phenylpyrrolidin-2-one (2o).** The General Procedure was followed using allene **1a** (92.2 mg, 0.300 mmol) and 3-methyl-5-bromophenylboronic acid (96.7 mg, 0.450 mmol). Filtration through a silica pad without purification by column chromatography gave the title compound (143 mg, >99%) as a white solid. $R_f$ = 0.31 (30% EtOAc/petroleum ether); m.p. 59-62 °C ($Et_2O$); IR 3359 (OH), 2916, 1678 (C=O), 1510, 1442, 1246, 1180, 1140, 1030, 827 $cm^{-1}$; $[\alpha]_D^{25}$ +20.0 (*c* 1.00, $CHCl_3$); $^1H$ NMR (400 MHz, $CDCl_3$) δ 7.61–7.55 (2H, m, Ar**H**), 7.36–7.21 (5H, m, Ar**H**), 7.15 (2H, dd, *J* = 1.6, 0.8 Hz, Ar**H**), 6.96–6.91 (2H, m, Ar**H**), 6.84 (1H, td, *J* = 1.5, 0.8 Hz, Ar**H**), 5.48 (1H, s, =C**H**$_2$), 5.36 (1H, d, *J* = 0.9 Hz, =C**H**$_2$), 4.02–

3.90 (2H, m, C**H**$_2$N), 3.82 (3H, s, OC**H**$_3$), 3.64 (1H, t, $J$ = 6.2 Hz C**H**CH$_2$), 3.45 (1H, s, O**H**), 2.19 (3H, d, $J$ = 0.8 Hz, ArC**H**$_3$); $^{13}$C NMR (101 MHz, CDCl$_3$) δ 173.0 (C), 157.2 (C), 144.3 (C), 143.7 (C), 141.8 (C), 139.8 (C), 131.9 (C), 131.0 (CH), 128.5 (2 × CH), 128.1 (CH), 127.1 (CH), 126.4 (CH), 125.6 (2 × CH), 122.1 (C), 121.9 (2 × CH), 116.8 (CH$_2$), 114.4 (2 × CH), 81.1 (C), 55.6 (CH$_3$), 50.9 (CH$_2$), 50.0 (CH), 21.2 (CH$_3$); HRMS (ESI) Exact mass calculated for $[C_{26}H_{25}BrNO_3]^+$ $[M+H]^+$: 478.1012, found: 478.1023; Enantiomeric excess was determined by HPLC with a Chiralpak IC column 90:10 (*iso*-hexane:*i*-PrOH, 1.0 mL/min, 254 nm, 25 °C) $t_r$ (minor) = 41.2 min, $t_r$ (major) = 54.2 min, 98% ee.

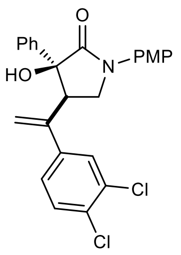


**(3*S*,4*R*)-4-[1-(3,4-Dichlorophenyl)vinyl]-3-hydroxy-1-(4-methoxyphenyl)-3-phenylpyrrolidin-2-one (2p).** The General Procedure was followed using allene **1a** (92.2 mg, 0.300 mmol) and 3,4-dichlorophenylboronic acid (85.9 mg, 0.450 mmol). Filtration through a silica pad without purification by column chromatography gave the title compound (136 mg, >99%) as a white solid. $R_f$ = 0.27 (30% EtOAc/petroleum ether); m.p. 61-64 °C (Et$_2$O); 3348 (OH), 2954, 1677 (C=O), 1509, 1469, 1396, 1244, 1027, 825, 696 cm$^{-1}$; $[\alpha]_D^{25}$ +8.0 (*c* 1.00, CHCl$_3$); $^1$H NMR (400 MHz, CDCl$_3$) δ 7.62–7.53 (2H, m, Ar**H**), 7.32–7.23 (7H, m, Ar**H**), 7.05 (1H, dd, $J$ = 8.3, 2.2 Hz, Ar**H**), 6.97–6.91 (2H, m, Ar**H**), 5.47 (1H, s, =C**H**$_2$), 5.35 (1H, d, $J$ = 0.9 Hz, =C**H**$_2$), 3.96 (2H, d, $J$ = 5.9 Hz, C**H**$_2$N), 3.83 (3H, s, OC**H**$_3$), 3.66–3.58 (1H, m, C**H**CH$_2$), 3.42 (1H, s, O**H**); $^{13}$C NMR (101 MHz, CDCl$_3$) δ 173.0 (C), 157.3 (C), 143.7 (C), 142.0 (C), 141.7 (C), 132.2 (C), 131.8 (C), 131.4 (C), 130.0 (CH), 129.0 (CH), 128.6 (2 × CH), 128.2 (CH), 126.3 (CH), 125.5 (2 × CH), 121.9 (2 × CH), 117.2 (CH$_2$), 114.4 (2 × CH), 81.1 (C), 55.6 (CH$_3$), 50.7 (CH$_2$), 50.0 (CH); HRMS (ESI) Exact mass calculated for $[C_{25}H_{22}Cl_2NO_3]^+$ $[M+H]^+$: 454.0971, found: 454.0972; Enantiomeric excess was determined by HPLC with a Chiralpak IC column (80:20 *iso*-hexane:*i*-PrOH, 1.0 mL/min, 254 nm, 25 °C) $t_r$ (minor) = 19.2 min, $t_r$ (major) = 25.3 min, 98% ee.

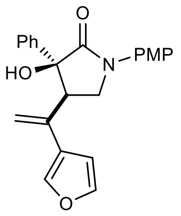


**(3*S*,4*R*)-4-[1-(Furan-3-yl)vinyl]-3-hydroxy-1-(4-methoxyphenyl)-3-phenylpyrrolidin-2-one (2q).** The General Procedure was followed using allene **1a** (92.2 mg, 0.300 mmol) and 3-furylboronic acid (50.4 mg, 0.450 mmol). Purification by column chromatography (5% EtOAc/pentane to 30% EtOAc/pentane) gave the title compound (103 mg, 91%) as a white solid as a 7:1 mixture of diastereomers. $R_f$ = 0.22 (30% EtOAc/petroleum ether); m.p. 160-162 °C (Et$_2$O); IR 3309 (OH), 2949, 1687 (C=O), 1510, 1396, 1291, 1273, 1214, 906, 698 cm$^{-1}$; $[\alpha]_D^{25}$ +48.0 (*c* 1.00, CHCl$_3$); $^1$H NMR (400 MHz, CDCl$_3$) *major diasteromer*: δ 7.70–7.59 (2H, m, Ar**H**), 7.49–7.42 (2H, m, Ar**H**), 7.42–7.31 (4H, m,

Ar**H**), 7.15 (1H, t, *J* = 1.2 Hz, Ar**H**), 7.05–6.94 (2H, m, Ar**H**), 6.48 (1H, dd, *J* = 1.9, 0.9 Hz, Ar**H**), 5.54 (1H, s, =C**H**$_2$), 5.23 (1H, s, =C**H**$_2$), 4.05–3.93 (2H, m, C**H**$_2$N), 3.85 (3H, s, OC**H**$_3$), 3.57–3.49 (1H, m, C**H**CH$_2$), 3.23 (1H, s, O**H**); *characteristic signals for the minor diastereomer*: 7.14 (1H, t, *J* = 1.2 Hz, Ar**H**), 3.35 (1H, s, O**H**); $^{13}$C NMR (101 MHz, CDCl$_3$) *major diasteromer*: δ 172.9 (C), 157.3 (C), 143.3 (CH), 142.4 (C), 139.5 (CH), 136.5 (C), 131.9 (C), 128.8 (2 × CH), 128.3 (CH), 127.2 (C), 125.5 (2 × CH), 121.8 (2 × CH), 114.4 (2 × CH), 113.6 (CH$_2$), 108.8 (CH), 80.8 (C), 55.6 (CH$_3$), 50.7 (CH$_2$), 49.9 (CH); HRMS (ESI) Exact mass calculated for $[C_{23}H_{22}NO_4]^+$ $[M+H]^+$: 376.1543, found: 376.1542; Enantiomeric excess was determined by HPLC with a Chiralpak ODH column (85:15 *iso*-hexane:*i*-PrOH, 1.0 mL/min, 254 nm, 25 °C) $t_r$ (minor) = 18.1 min, $t_r$ (major) = 20.9 min, 91% ee.

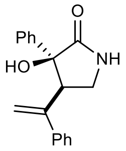


**(3*S*,4*R*)-3-Hydroxy-3-phenyl-4-(1-phenylvinyl)pyrrolidin-2-one (2r).** A modification of the General Procedure (in that the reaction time was 40 h rather than 24 h) was followed using allene **1g** (60.4 mg, 0.300 mmol) and phenylboronic acid (54.9 mg, 0.450 mmol). Purification by column chromatography (8% MeOH/CH$_2$Cl$_2$) gave the title compound (75.3 mg, 90%) as a white solid. R$_f$ = 0.37 (8% MeOH/CH$_2$Cl$_2$); m.p. 200–204 °C (CH$_2$Cl$_2$); $[\alpha]_D^{25}$ +36.4 (*c* 0.22, (CH$_3$)$_2$CO); IR 3263 (OH), 1692 (C=O), 1403, 1279, 1128, 931, 795, 745, 692, 602, 549, 514, 429 cm$^{-1}$; $^1$H NMR (400 MHz, (CD$_3$)$_2$SO) δ 8.16 (1H, s, N**H**), 7.23–7.10 (5H, m, Ar**H**), 7.09–6.96 (5H, m, Ar**H**), 5.88 (1H, s, O**H**), 5.34 (1H, d, *J* = 1.2 Hz, =C**H**$_2$), 5.31 (1H, s, =C**H**$_2$), 3.57 (1H, t, *J* = 7.8 Hz, C**H**CH$_2$), 3.48-3.38 (2H, m, NC**H**$_2$); $^{13}$C NMR (101 MHz, (CD$_3$)$_2$SO) δ 176.1 (C), 144.2 (C), 142.8 (C), 142.1 (C), 127.6 (2 × CH), 127.1 (2 × CH), 126.7 (CH), 126.4 (CH), 126.3 (2 × CH), 126.1 (2 × CH), 116.2 (CH$_2$), 78.6 (C), 52.1 (CH), 43.6 (CH$_2$); HRMS (ESI) Exact mass calcd for $[C_{18}H_{17}NNaO_2]^+$ $[M+Na]^+$: 302.1151, found: 302.1144. Enantiomeric excess was determined by HPLC with a Chiralpak IC column (70:30 *iso*-hexane:*i*-PrOH, 1.0 mL/min, 254 nm, 25 °C); $t_r$ (major) = 10.5 min, $t_r$ (minor) = 18.8 min, 99% ee.

**(3*S*,4*R*)-4-(Buta-1,3-dien-2-yl)-3-hydroxy-1-(4-methoxyphenyl)-3-phenylpyrrolidin-2-one (3)**

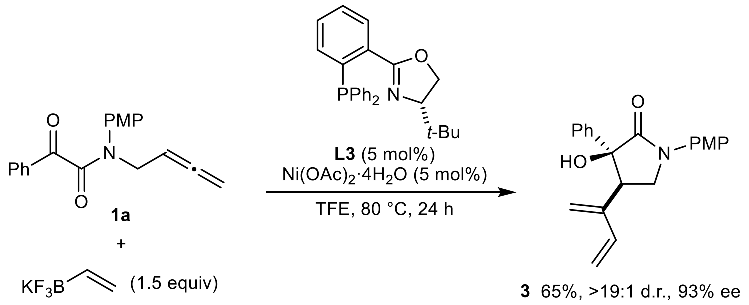


A modification of the General Procedure (in that potassium vinyltrifluoroborate was used instead of a boronic acid) was followed using allene **1a** (92.2 mg, 0.300 mmol) and potassium

vinyltrifluoroborate (60.3 mg, 0.450 mmol). Purification by column chromatography (5% EtOAc/pentane to 30% EtOAc/pentane) gave the title compound (83.4 mg, 65%) as a white oil. $R_f$ = 0.26 (30% EtOAc/petroleum ether); IR 3369 (OH), 2931, 1681 (C=O), 1593, 1510, 1441, 1399, 1245, 1180, 828 $cm^{-1}$; $[\alpha]_D^{25}$ +92.0 (*c* 1.00, $CHCl_3$); $^1H$ NMR (400 MHz, $CDCl_3$) δ 7.69–7.61 (2H, m, Ar**H**), 7.47–7.40 (2H, m, Ar**H**), 7.40–7.28 (3H, m, Ar**H**), 7.00–6.93 (2H, m, Ar**H**), 6.41 (1H, ddd, *J* = 17.5, 11.0, 0.8 Hz, $CH_2$=C**H**), 5.36 (1H, s, C**H**$_2$=C), 5.20 (1H, d, *J* = 1.0 Hz, C**H**$_2$=C), 5.12 (1H, d, *J* = 17.5 Hz, C**H**$_2$=CH), 5.04 (1H, dd, *J* = 11.0, 0.9 Hz, CHC**H**$_2$), 3.93 (1H, dd, *J* = 10.0, 6.8 Hz, C**H**$_2$N), 3.88–3.84 (1H, m, C**H**$_2$N), 3.83 (3H, s, OC**H**$_3$), 3.53 (1H, dd, *J* = 6.8, 4.0 Hz, C**H**$CH_2$N), 3.18 (1H, s, O**H**); $^{13}C$ NMR (101 MHz, $CDCl_3$) δ 173.1 (C), 157.2 (C), 143.3 (C), 142.3 (C), 138.8 (CH), 131.9 (C), 128.8 (2 × CH), 128.2 (CH), 125.5 (2 × CH), 121.7 (2 × CH), 117.2 ($CH_2$), 114.6 ($CH_2$), 114.4 (2 × CH), 80.8 (C), 55.6 ($CH_3$), 50.7 ($CH_2$), 46.3 (CH); HRMS (ESI) Exact mass calculated for $[C_{21}H_{22}NO_3]^+$ $[M+H]^+$: 336.1594, found: 336.1589; Enantiomeric excess was determined by HPLC with a Chiralcel OD-H column (90:10 *iso*-hexane:*i*-PrOH), 1.0 mL/min, 230 nm, 25 °C) $t_r$ (minor) = 16.7 min, $t_r$ (major) = 20.6 min, 93% ee.

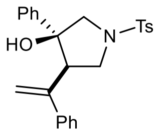


**(3*S*,4*R*)-3-Phenyl-4-(1-phenylvinyl)-1-tosylpyrrolidin-3-ol (5a).** The General Procedure was followed using allene **4a** (102 mg, 0.300 mmol) and phenylboronic acid (54.9 mg, 0.450 mmol). Purification by column chromatography (20% EtOAc/petroleum ether) gave the title compound (116 mg, 92%) as a colorless amorphous solid. $R_f$ = 0.33 (20% EtOAc/petroleum ether); $[\alpha]_D^{25}$ –36.4 (*c* 0.33, $CHCl_3$); IR 3486 (OH), 2894, 1632, 1595, 1489, 1446, 1292, 1137, 1102, 907, 818, 752, 682, 639, 595, 544, 516, 470 $cm^{-1}$; $^1H$ NMR (400 MHz, $CDCl_3$) δ 7.81–7.78 (2H, m, Ar**H**), 7.38–7.35 (2H, m, Ar**H**), 7.17–7.13 (2H, m, Ar**H**), 7.11–7.01 (6H, m, Ar**H**), 6.88–6.84 (2H, m, Ar**H**), 5.32 (1H, s, =C**H**$_2$), 5.15 (1H, s, =C**H**$_2$), 3.92 (1H, dd, *J* = 9.3, 7.2 Hz, CHC**H**$_2$), 3.76 (1H, d, *J* = 11.4 Hz, CC**H**$_2$N), 3.75–3.70 (1H, m, C**H**$CH_2$), 3.64 (1H, d, *J* = 11.4 Hz, CC**H**$_2$N), 3.54 (1H, dd, *J* = 11.4, 9.3 Hz, CHC**H**$_2$), 2.47 (3H, s, ArC**H**$_3$), 2.26 (1H, s, O**H**); $^{13}C$ NMR (101 MHz, $CDCl_3$) δ 143.9 (C), 143.7 (C), 141.6 (C), 140.8 (C), 134.0 (C), 129.8 (2 × CH), 128.1 (2 × CH), 128.0 (2 × CH), 127.7 (2 × CH), 127.4 (CH), 127.3 (CH), 126.6 (2 × CH), 125.1 (2 × CH), 117.3 ($CH_2$), 80.4 (C), 62.8 ($CH_2$), 53.6 (CH), 51.4 ($CH_2$), 21.7 ($CH_3$); HRMS (ESI) Exact mass calculated for $[C_{25}H_{26}NO_3S]^+$ $[M+H]^+$: 420.1628, found: 420.1631; Enantiomeric excess was determined by HPLC with a Chiralpak AD-H column (90:10 *iso*-hexane:*i*-PrOH, 1.0 mL/min, 254 nm, 25 °C) $t_r$ (minor) = 23.0 min, $t_r$ (major) = 31.9 min, 84% ee.

Slow diffusion of petroleum ether into a solution of **5a** in EtOAc gave crystals that were suitable for X-ray crystallography:

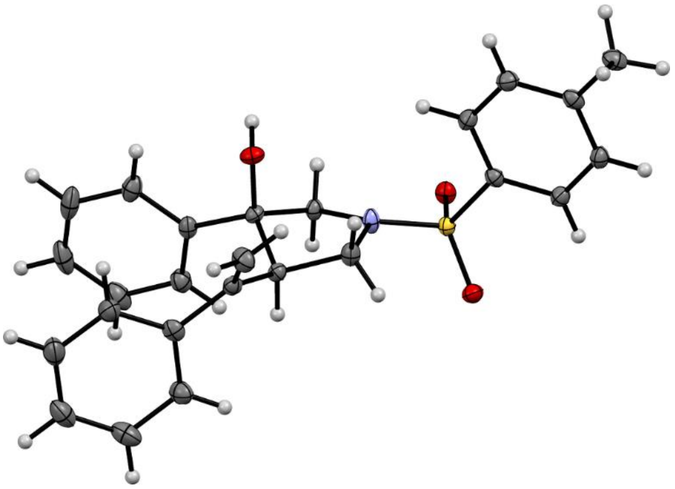

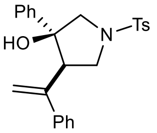

**5a**
CCDC 1945865
Flack parameter –0.008(5)

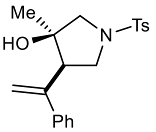

**(3*R*,4*R*)-3-Methyl-4-(1-phenylvinyl)-1-tosylpyrrolidin-3-ol (5b).** The General Procedure was followed using allene **4b** (83.8 mg, 0.300 mmol) and phenylboronic acid (54.9 mg, 0.450 mmol). Purification by column chromatography (20% EtOAc/petroleum ether) gave the title compound (103 mg, 96%) as a colorless oil. $R_f$ = 0.38 (20% EtOAc/petroleum ether); $[\alpha]_D^{25}$ –80.0 (*c* 0.35, $CHCl_3$); IR 3512 (OH), 2968, 1626, 1598, 1493, 1380, 1152, 1092, 934, 812, 778, 704, 664, 588, 547 cm$^{-1}$; $^1$H NMR (500 MHz, $CDCl_3$) δ 7.77 (2H, d, *J* = 8.2 Hz, Ar**H**), 7.34 (2H, d, *J* = 8.2 Hz, Ar**H**), 7.33–7.24 (5H, m, Ar**H**), 5.46 (1H, s, =C**H**$_2$), 5.15 (1H, s, =C**H**$_2$), 3.75 (1H, dd, *J* = 9.5, 7.3 Hz, C**H**CH$_2$), 3.47–3.42 (2H, m, one of CHC**H**$_2$ and one of CC**H**$_2$N), 3.35 (1H, d, *J* = 11.0 Hz, CC**H**$_2$N), 3.17 (1H, dd, *J* = 11.2, 7.3 Hz, CHC**H**$_2$), 2.45 (3H, s, ArC**H**$_3$), 1.53 (1H, s, O**H**), 0.94 (3H, s, C**H**$_3$COH); $^{13}$C NMR (126 MHz, $CDCl_3$) δ 144.0 (C), 143.6 (C), 142.5 (C), 134.3 (C), 129.8 (2 × CH), 128.8 (2 × CH), 128.1 (CH), 127.7 (2 × CH), 126.6 (2 × CH), 116.8 ($CH_2$), 77.0 (C), 60.7 ($CH_2$), 52.0 (CH), 51.4 ($CH_2$), 25.0 ($CH_3$), 21.7 ($CH_3$); HRMS (ESI) Exact mass calculated for $[C_{20}H_{24}NO_3S]^+$ $[M+H]^+$: 358.1471, found: 358.1474; Enantiomeric excess was determined by HPLC with a Chiralcel OD-H column (90:10 *iso*-hexane:*i*-PrOH, 1.0 mL/min, 254 nm, 25 °C) $t_r$ (minor) = 27.2 min, $t_r$ (major) = 30.6 min, 83% ee.

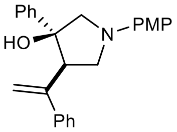

**(3*S*,4*R*)-1-(4-Methoxyphenyl)-3-phenyl-4-(1-phenylvinyl)pyrrolidin-3-ol (5c).** The General Procedure was followed using allene **4c** (87.9 mg, 0.300 mmol) and phenylboronic acid (54.9 mg, 0.450 mmol). Purification by column chromatography (10% EtOAc/pentane) gave the title compound (69.1 mg, 62%) as a pale yellow solid. $R_f$ = 0.47 (20% EtOAc/petroleum ether); m.p. 170–174 °C ($Et_2O$); $[\alpha]_D^{25}$ –21.1 (*c* 0.19, $CHCl_3$); IR 3529 (OH), 2906, 1620, 1511, 1471, 1445, 1371, 1346, 1270, 1238, 1178, 1109, 1038, 898, 815, 766, 664, 596, 511, 410 cm$^{-1}$; $^1$H NMR (400 MHz, $CDCl_3$) δ 7.40–7.37 (2H, m, Ar**H**), 7.21–7.16 (2H, m, Ar**H**), 7.15–7.10 (4H, m, Ar**H**), 7.07–7.03 (2H, m, Ar**H**), 6.90–6.86 (2H, m, Ar**H**), 6.58–6.53 (2H, m, Ar**H**), 5.46

(1H, d, *J* = 0.8 Hz, =C**H**$_2$), 5.41 (1H, s, =C**H**$_2$), 4.01–3.96 (1H, m, C**H**CH$_2$), 3.83 (1H, d, *J* = 10.0 Hz, CC**H**$_2$N), 3.80–3.74 (1H, m, CHC**H**$_2$), 3.78 (3H, s, OC**H**$_3$), 3.68 (1H, dd, *J* = 10.1, 9.0 Hz, CHC**H**$_2$), 3.63 (1H, d, *J* = 10.4 Hz, CC**H**$_2$N), 2.49 (1H, s, O**H**); $^{13}$C NMR (101 MHz, $CDCl_3$) δ 151.4 (C), 145.8 (C), 142.8 (C), 142.5 (C), 142.4 (C), 128.1 (4 × CH), 127.4 (CH), 127.1 (CH), 126.8 (2 × CH), 125.4 (2 × CH), 116.8 ($CH_2$), 115.2 (2 × CH), 112.6 (2 × CH), 80.7 (C), 64.5 ($CH_2$), 56.1 ($CH_3$), 53.8 (CH), 52.6 ($CH_2$); HRMS (ESI) Exact mass calculated for $[C_{25}H_{26}NO_2]^+$ $[M+H]^+$: 372.1958, found: 372.1962; Enantiomeric excess was determined by HPLC with a Chiralpak AD-H column (90:10 *iso*-hexane:*i*-PrOH, 1.0 mL/min, 254 nm, 25 °C) $t_r$ (major) = 28.8 min, $t_r$ (minor) = 37.0 min, 75% ee.

*Using MeCN as the solvent*: A modification of the General Procedure was followed using allene **4c** (87.9 mg, 0.30 mmol) and phenylboronic acid (54.9 mg, 0.45 mmol) but using MeCN in place of TFE as the solvent. Purification by column chromatography (20% EtOAc/petroleum ether) gave the title compound (60.4 mg, 54%) as a pale yellow solid. $[\alpha]_D^{25}$ –28.6 (*c* 0.28, $CHCl_3$); Enantiomeric excess was determined by HPLC with a Chiralpak AD-H column (90:10 *iso*-hexane:*i*-PrOH, 1.0 mL/min, 254 nm, 25 °C); $t_r$ (major) = 29.1 min, $t_r$ (minor) = 37.3 min, 80% ee.

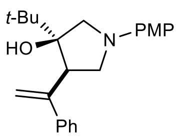


**(3*S*,4*R*)-3-(*tert*-Butyl)-1-(4-methoxyphenyl)-4-(1-phenylvinyl)pyrrolidin-3-ol (5d).** The General Procedure was followed using allene **xx** (81.9 mg, 0.300 mmol) and phenylboronic acid (54.9 mg, 0.450 mmol). Purification by column chromatography (1% EtOAc/$CH_2Cl_2$) gave a 7.7:1 inseparable mixture of the title compound and unreacted allene **4d** (85.2 mg, 73% yield of **5d**, adjusted for the presence of unreacted **4d**), as a colorless oil. $R_f$ = 0.50 (20% EtOAc/petroleum ether); $[\alpha]_D^{25}$ –47.1 (*c* 0.68, $CHCl_3$); IR 2965, 1953, 1716, 1673, 1511, 1465, 1365, 1240, 1179, 1036, 974, 905, 812, 775, 710, 547 cm$^{-1}$; $^1$H NMR (400 MHz, $CDCl_3$) δ 7.42–7.26 (5H, m, Ar**H**), 6.88–6.86 (2H, m, Ar**H**), 6.61–6.54 (2H, m, Ar**H**), 5.55 (1H, s, =C**H**$_2$), 5.54 (1H, s, =C**H**$_2$), 3.78–3.74 (1H, m, one of CHC**H**$_2$), 3.77 (3H, s, OC**H**$_3$), 3.67–3.53 (3H, m, CC**H**$_2$N and two of C**H**C**H**$_2$), 3.27 (1H, d, *J* = 10.3 Hz, CC**H**$_2$N), 2.12 (1H, s, O**H**), 0.86 (9H, s, C(C**H**$_3$)$_3$); $^{13}$C NMR (101 MHz, $CDCl_3$) δ 151.3 (C), 147.4 (C), 143.5 (C), 142.8 (C), 128.7 (2 × CH), 127.7 (CH), 126.8 (2 × CH), 117.4 ($CH_2$), 115.2 (2 × CH), 112.6 (2 × CH), 85.0 (C), 58.7 ($CH_2$), 56.7 ($CH_2$), 56.1 ($CH_3$), 46.8 (CH), 37.3 (C), 26.2 (3 × $CH_3$); HRMS (ESI) Exact mass calculated for $[C_{23}H_{30}NO_2]^+$ $[M+H]^+$: 352.2271, found: 352.2272; Enantiomeric excess was determined by HPLC with a Chiralpak AD-H column (90:10 *iso*-hexane:*i*-PrOH, 1.0 mL/min, 254 nm, 25 °C) $t_r$ (major) = 11.5 min, $t_r$ (minor) = 12.9 min, 99% ee.

Recrystallization of **5d** from EtOAc gave crystals that were suitable for X-ray crystallography:

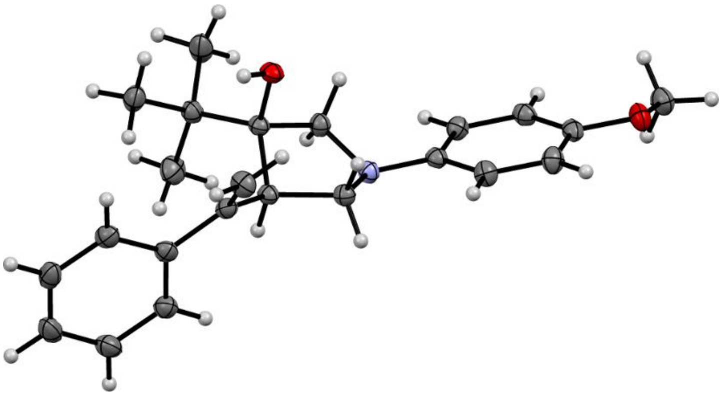

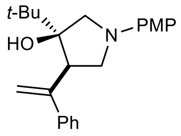


**5d**
CCDC 1945866
Flack parameter –0.06(8)

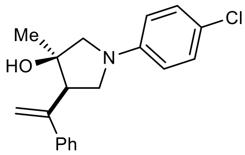


**(3*R*,4*R*)-1-(4-Chlorophenyl)-3-methyl-4-(1-phenylvinyl)pyrrolidin-3-ol (5e).** The General Procedure was followed using allene **4e** (70.7 mg, 0.300 mmol) and phenylboronic acid (54.9 mg, 0.450 mmol). Purification by column chromatography (10% EtOAc/pentane) gave the title compound (89.3 mg, 91%) as a pale yellow solid. $R_f$ = 0.43 (20% EtOAc/petroleum ether); m.p. 90–94 °C ($Et_2O$); $[\alpha]_D^{25}$ –53.3 (*c* 0.30, $CHCl_3$); IR 3546 (OH), 2974, 2845, 1629, 1596, 1498, 1471, 1376, 1324, 1184, 1120, 939, 908, 813, 778, 703, 647, 600, 509, 458 $cm^{-1}$; $^1H$ NMR (400 MHz, $CDCl_3$) δ 7.44–7.32 (5H, m, Ar**H**), 7.21–7.17 (2H, m, Ar**H**), 6.49–6.45 (2H, m, Ar**H**), 5.60 (1H, d, *J* = 0.6 Hz, =C**H**$_2$), 5.41 (1H, s, =C**H**$_2$), 3.68–3.59 (2H, m, CHC**H**$_2$), 3.49–3.38 (3H, m, CC**H**$_2$N and C**H**CH$_2$), 1.94 (1H, s, O**H**), 1.17 (3H, s, C**H**$_3$); $^{13}C$ NMR (101 MHz, $CDCl_3$) δ 146.1 (C), 145.2 (C), 143.1 (C), 129.0 (2 × CH), 128.7 (2 × CH), 127.9 (CH), 126.7 (2 × CH), 120.6 (C), 116.4 ($CH_2$), 112.4 (2 × CH), 77.1 (C), 61.4 ($CH_2$), 52.1 (CH), 51.9 ($CH_2$), 25.4 ($CH_3$); HRMS (ESI) Exact mass calculated for $[C_{19}H_{21}ClNO]^+$ $[M+H]^+$: 314.1306, found: 314.1302; Enantiomeric excess was determined by HPLC with a Chiralpak AD-H column (90:10 *iso*-hexane:*i*-PrOH, 1.0 mL/min, 254 nm, 25 °C) $t_r$ (major) = 11.0 min, $t_r$ (minor) = 25.1 min, 90% ee.

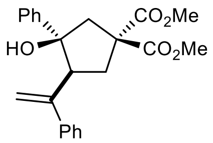


**Dimethyl (3*S*,4*S*)-3-hydroxy-3-phenyl-4-(1-phenylvinyl)cyclopentane-1,1-dicarboxylate (5f).** A modification of the General Procedure (in that MeCN was used as the solvent in place of TFE) was followed using allene **4f** (90.7 mg, 0.300 mmol) and phenylboronic acid (54.9 mg, 0.450 mmol). Purification by column chromatography (5% EtOAc/petroleum ether to 20% EtOAc/petroleum ether) gave the title compound (37.1 mg, 33%) as a colorless oil. $R_f$ = 0.41 (30% EtOAc/petroleum ether); $[\alpha]_D^{20}$ +0.28 (*c* 1.00, $CHCl_3$); IR 3520 (OH), 2953, 1727 (C=O), 1494, 1434, 1251, 1198, 1168, 1033, 907, 756, 697, 550 $cm^{-1}$; $^1H$ NMR (500 MHz, $CDCl_3$) δ 7.31–7.27 (2H, m, Ar**H**), 7.13–7.07 (2H, m, Ar**H**), 7.06–7.01 (4H, m, Ar**H**), 6.95–6.89 (2H, m, Ar**H**), 5.34–5.33 (2H, m, C=C**H**$_2$), 3.81 (3H, s, C**H**$_3$), 3.80 (3H, s, C**H**$_3$), 3.73 (1H, dd, *J* = 12.5, 7.2 Hz, C**H**CH$_2$), 2.89 (1H, dd, *J* = 13.6, 12.5 Hz, CHC**H**$_2$), 2.87–2.80 (2H, m, HOCC**H**$_2$), 2.69 (1H, dd, *J* = 13.6, 7.2 Hz, CHC**H**$_2$) 2.47 (1H, s, O**H**); $^{13}C$ NMR (126 MHz, $CDCl_3$) δ 173.5 (C),

173.0 (C), 146.7 (C), 143.4 (C), 142.5 (C), 128.0 (2 × CH), 127.9 (2 × CH), 127.1 (CH), 126.79 (2 × CH), 126.77 (CH), 125.2 (2 × CH), 116.5 ($CH_2$), 82.3 (C), 57.2 (C), 54.8 (CH), 53.24 ($CH_3$), 53.17 ($CH_3$), 50.6 ($CH_2$), 38.5 ($CH_2$); HRMS (ESI) Exact mass calculated for $[C_{23}H_{24}NaO_5]^+$ $[M+Na]^+$: 403.1516, found: 403.1523. Enantiomeric excess was determined by HPLC with a Chiralpak OD-H column (90:10 *iso*-hexane:*i*-PrOH, 1.0 mL/min, 254 nm, 25 °C) $t_r$ (major) = 9.1 min, $t_r$ (minor) = 10.5 min, 90% ee.

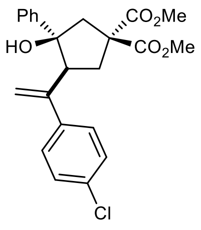


**Dimethyl (3*S*,4*S*)-4-[1-(4-chlorophenyl)vinyl]-3-hydroxy-3-phenylcyclopentane-1,1-dicarboxylate (5g).** A modification of the General Procedure (in that MeCN was used as the solvent in place of TFE) was followed using allene **4f** (90.7 mg, 0.300 mmol) and 4-chlorophenylboronic acid (70.4 mg, 0.450 mmol). Purification by column chromatography (5% EtOAc/pentane to 40% EtOAc/pentane) gave the title compound (57.9 mg, 47%) as a colorless oil. $R_f$ = 0.42 (30% EtOAc/petroleum ether); IR 3523 (OH), 2953, 1726 (C=O), 1622, 1491, 1434, 1250, 1099, 964, 731 $cm^{-1}$; $[\alpha]_D^{25}$ +40.0 (*c* 1.00, $CHCl_3$) ; $^1$H NMR (400 MHz, $CDCl_3$) δ 7.20–7.15 (2H, m, Ar**H**), 7.06–6.94 (3H, m, Ar**H**), 6.93–6.86 (2H, m, Ar**H**), 6.77–6.70 (2H, m, Ar**H**), 5.27 (1H, s, =C**H**$_2$), 5.25 (1H, s, =C**H**$_2$), 3.73 (3H, s, C**H**$_3$), 3.72 (3H, s, C**H**$_3$), 3.62–3.50 (1H, m, C**H**$CH_2$), 2.78 (1H, dd, *J* = 13.6, 12.5 Hz, CHC**H**$_2$), 2.76 (2H, br s, HOCC**H**$_2$), 2.60 (1H, dd, *J* = 13.6, 7.3 Hz, CHC**H**$_2$), 2.36 (1H, s, O**H**); $^{13}$C NMR (101 MHz, $CDCl_3$) δ 173.4 (C), 172.9 (C), 145.5 (C), 143.2 (C), 140.9 (C), 132.9 (C), 128.1 (2 × CH), 128.00 (2 × CH), 127.95 (2 × CH), 126.8 (CH), 125.1 (2 × CH), 117.0 ($CH_2$), 82.3 (C), 57.1 (C), 55.0 (CH), 53.25 ($CH_3$), 53.19 ($CH_3$), 50.5 ($CH_2$), 38.3 ($CH_2$); HRMS (ESI) Exact mass calculated for $[C_{23}H_{24}ClO_5]^+$ $[M+H]^+$: 415.1307, found: 415.1305; Enantiomeric excess was determined by HPLC with a Chiralpak AD-H column (90:10 *iso*-hexane:*i*PrOH, 1.0 mL/min, 254 nm, 25 °C) $t_r$ (minor) = 12.3 min, $t_r$ (major) = 21.2 min, 93% ee.

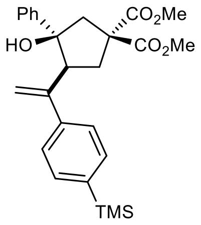


**Dimethyl (3*S*,4*S*)-3-hydroxy-3-phenyl-4-{1-[4-(trimethylsilyl)phenyl]-vinyl}cyclopentane-1,1-dicarboxylate (5h).** A modification of the General Procedure (in that MeCN was used as the solvent in place of TFE) was followed using allene **4f** (90.7 mg, 0.300 mmol) and 4-(trimethylsilyl)phenylboronic acid (87.3 mg, 0.450 mmol). Purification by column chromatography (5% EtOAc/pentane to 20% EtOAc/pentane) gave the title compound (46.0 mg, 34%) as a colorless oil. $R_f$ = 0.56 (30% EtOAc/petroleum ether); IR 3523 (OH), 2953, 1729 (C=O), 1597, 1495, 1247, 1168, 1092, 908, 826 $cm^{-1}$; $[\alpha]_D^{25}$ +32.0 (*c* 1.00, $CHCl_3$); $^1$H NMR (400 MHz, $CDCl_3$) δ 7.26 (2H, d, *J* = 7.2 Hz, Ar**H**), 7.20–7.13 (2H, m, Ar**H**), 7.11–7.03 (2H, m, Ar**H**), 7.03–6.97 (1H, m, Ar**H**), 6.89–6.83

(2H, m, Ar**H**), 5.36 (1H, s, =C**H**$_2$), 5.34 (1H, s, =C**H**$_2$), 3.81 (3H, s, C**H**$_3$), 3.79 (3H, s, C**H**$_3$), 3.70 (1H, dd, *J* = 12.4, 7.2 Hz, C**H**CH$_2$), 2.89 (1H, dd, *J* = 13.6, 12.5 Hz, CHC**H**$_2$), 2.84 (2H, s, HOCC**H**$_2$), 2.68 (1H, dd, *J* = 13.6, 7.3 Hz, CHC**H**$_2$), 2.44 (1H, br s, O**H**), 0.20 (9H, s, Si(C**H**$_3$)$_3$); $^{13}$C NMR (101 MHz, CDCl$_3$) δ 173.5 (C), 172.9 (C), 146.7 (C), 143.5 (C), 142.7 (C), 139.0 (C), 132.9 (2 × CH), 127.9 (2 × CH), 126.7 (CH), 126.2 (2 × CH), 125.2 (2 × CH), 116.5 (CH$_2$), 82.3 (C), 57.1 (C), 54.9 (CH), 53.22 (CH$_3$), 53.15 (CH$_3$), 50.6 (CH$_2$), 38.3 (CH$_2$), –1.1 (3 × CH$_3$); HRMS (ESI) Exact mass calculated for $[C_{26}H_{33}O_5Si]^+$ $[M+H]^+$: 453.2092, found: 453.2083; Enantiomeric excess was determined by HPLC with a Chiralpak AS-H column (97:3 *iso*-hexane:*i*PrOH, 1.0 mL/min, 254 nm, 25 °C) $t_r$ (major) = 11.3 min, $t_r$ (minor) = 13.2 min, 89% ee.

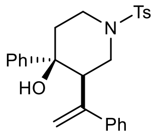


**(3*R*,4*S*)-4-Phenyl-4-(1-phenylvinyl)-1-tosylpiperidin-4-ol (5i).** A modification of the General Procedure (in that the reaction time was 48 h rather than 24 h) was followed using allene **4g** (107 mg, 0.300 mmol) and phenylboronic acid (54.9 mg, 0.450 mmol). Purification by column chromatography (10% EtOAc/pentane) gave the title compound (65.6 mg, 50%) as a colorless solid. $R_f$ = 0.18 (20% EtOAc/petroleum ether); m.p. 151–152 °C (Et$_2$O); $[\alpha]_D^{25}$ +107 (*c* 0.15, CHCl$_3$); IR 3548 (OH), 2980, 1599, 1444, 1341, 1159, 1091, 1043, 988, 917, 846, 814, 742, 693, 659, 573, 547, 434 cm$^{-1}$; $^1$H NMR (400 MHz, CDCl$_3$) δ 7.76–7.72 (2H, m, Ar**H**), 7.39–7.37 (2H, m, Ar**H**), 7.07–6.95 (8H, m, Ar**H**), 6.75–6.73 (2H, m, Ar**H**), 5.14 (1H, s, =C**H**$_2$), 5.03 (1H, s, =C**H**$_2$), 3.91 (1H, ddd, *J* = 11.5, 4.1, 1.9 Hz, C**H**$_2$N), 3.78 (1H, ddt, *J* = 11.6, 4.5, 2.0 Hz, C**H**$_2$N), 3.53 (1H, dd, *J* = 12.1, 4.0 Hz, C**H**$_2$N), 2.93–2.81 (2H, m, C**H**$_2$N and C**H**CH$_2$), 2.48 (3H, s, ArC**H**$_3$), 2.24–2.19 (1H, m, C**H**$_2$CH$_2$N), 2.15 (1H, d, *J* = 2.2 Hz, O**H**), 1.80 (1H, dt, *J* = 13.8, 2.5 Hz, C**H**$_2$CH$_2$N); $^{13}$C NMR (101 MHz, CDCl$_3$) δ 148.9 (C), 144.9 (C), 143.8 (C), 143.6 (C), 133.8 (C), 130.0 (2 × CH), 127.92 (2 × CH), 127.87 (2 × CH), 127.8 (2 × CH), 127.0 (CH), 126.9 (CH), 126.4 (2 × CH), 124.7 (2 × CH), 116.0 (CH$_2$), 72.5 (C), 49.9 (CH), 47.2 (CH$_2$), 42.5 (CH$_2$), 39.0 (CH$_2$), 21.7 (CH$_3$); HRMS (ESI) Exact mass calculated for $[C_{26}H_{28}NO_3S]^+$ $[M+H]^+$: 434.1784, found: 434.1792; Enantiomeric excess was determined by HPLC with a Chiralpak IC column (90:10 *iso*-hexane:*i*-PrOH, 1.0 mL/min, 254 nm, 25 °C) $t_r$ (major) = 33.3 min, $t_r$ (minor) = 36.0 min, 99% ee.

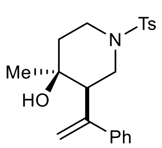


**(3*R*,4*S*)-4-Methyl-4-(1-phenylvinyl)-1-tosylpiperidin-4-ol (5j).** A modification of the General Procedure (in that MeCN was used as the solvent in place of TFE) was followed using allene **4h** (88.0 mg, 0.300 mmol) and phenylboronic acid (54.9 mg, 0.450 mmol). Purification by column chromatography (5% EtOAc/petroleum ether to 20% EtOAc/petroleum ether) gave the title compound (74.5 mg, 67%) as a sticky amorphous solid. $R_f$ = 0.20 (20% EtOAc/petroleum ether); $[\alpha]_D^{25}$ +36.0 (*c* 1.00, CHCl$_3$); IR 3493 (OH), 2927, 2869, 1716,

1597, 1448, 1337, 1155, 1089, 907, 737, 547 cm$^{-1}$; $^{1}$H NMR (500 MHz, $CDCl_3$) δ 7.69–7.67 (2H, m, Ar**H**), 7.37–7.26 (7H, m), 5.41 (1H, s, =C**H**$_2$), 5.04 (1H, s, =C**H**$_2$), 3.73 (1H, ddd, *J* = 11.3, 3.9, 2.0 Hz, C**H**$_2$N), 3.67 (1H, ddt, *J* = 11.5, 4.8, 2.3 Hz, C**H**$_2$N), 2.98 (1H, dd, *J* = 12.1, 3.9 Hz, C**H**$_2$N), 2.75–2.67 (2H, m, C**H**$_2$N and C**H**$CH_2$), 2.45 (3H, s, ArC**H**$_3$), 1.81–1.67 (2H, m, C**H**$_2$$CH_2$N), 1.52 (1H, s, O**H**), 0.94 (3H, s, C**H**$_3$COH); $^{13}$C NMR (126 MHz, $CDCl_3$) δ 148.3 (C), 143.7 (C), 143.6 (C), 133.7 (C), 129.9 (2 × CH), 128.8 (2 × CH), 127.9 (CH), 127.8 (2 × CH), 126.4 (2 × CH), 115.6 ($CH_2$), 68.7 (C), 49.0 (CH), 47.1 ($CH_2$), 42.3 ($CH_2$), 38.3 ($CH_2$), 29.8 ($CH_3$), 21.7 ($CH_3$); HRMS (ESI) Exact mass calculated for $[C_{21}H_{26}NO_3S]^+$ $[M+H]^+$: 372.1628, found: 372.1627; Enantiomeric excess was determined by HPLC with a Chiralpak AS-H column (90:10 *iso*-hexane:*i*-PrOH, 1.5 mL/min, 210 nm, 25 °C) $t_r$ (major) = 29.3 min, $t_r$ (minor) = 35.2 min, 85% ee.

**Dimethyl (3*S*,4*S*)-3-[1-(4-chlorophenyl)vinyl]-4-hydroxy-4-methylcyclohexane-1,1-dicarboxylate (5k)**

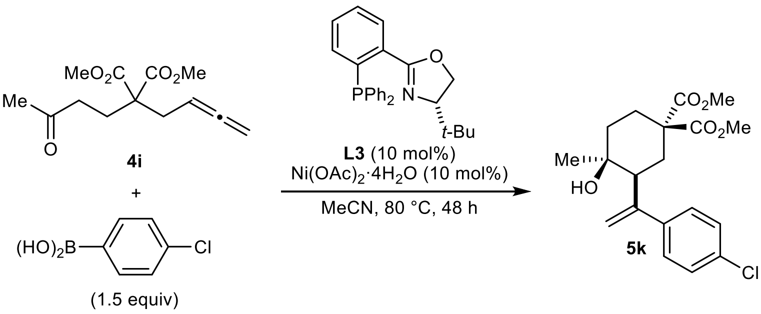


To an oven-dried microwave vial charged with a magnetic stirrer, $Ni(OAc)_2 \cdot 4H_2O$ (7.5 mg, 30 μmol), (*S*)-$^{t}$BuPHOX (**L3**, 11.6 mg, 30 μmol), and 4-chlorophenylboronic acid (70.4 mg, 0.450 mmol) were added. The vial was sealed and flushed with nitrogen or argon for 10 min. MeCN (1.5 mL) was added, the solution was immerged in an oil bath pre-heated to 80 °C and stirred for 10 min. Allene **4i** (76.3 mg, 0.300 mmol) was added to a separate vial that was sealed and flushed with argon for 10 min. MeCN (0.75 mL) was added to the allene and the resulting solution was added dropwise to the one containing the first vial containing the chiral nickel complex. The vial originally containing the substrate was rinsed with additional MeCN (0.75 mL) and the rinsing solution was transferred to the first microwave vial *via* syringe. The reaction mixture was stirred at 80 °C for 48 h, cooled to room temperature, diluted with EtOAc (5 mL), filtered through a short pad of silica (3 cm height × 2 cm wide) using EtOAc (20 mL) as eluent, and concentrated *in vacuo*. Purification of the residue by column chromatography (5% EtOAc/pentane to 20% EtOAc/pentane) gave the title compound (83.2 mg, 76%) as a yellow oil. $R_f$ = 0.29 (30% EtOAc/petroleum ether); IR 3538 (OH), 2952, 1721 (C=O), 1489, 1431, 1302, 1226, 1155, 1119, 893 cm$^{-1}$; $[\alpha]_D^{25}$ +88.0 (*c* 1.00, $CHCl_3$); $^{1}$H NMR (400 MHz, $CDCl_3$) δ 7.31–7.27 (4H, m, Ar**H**), 5.40 (1H, s, =C**H**$_2$), 5.27 (1H, s, =C**H**$_2$), 3.81 (3H, s, OC**H**$_3$), 3.73

(3H, s, OC**H**$_3$), 2.82 (1H, dd, *J* = 13.2, 3.4 Hz, C**H**CH$_2$), 2.33 (1H, ddd, *J* =13.3, 3.5, 2.2 Hz, CHC**H**$_2$), 2.28–2.13 (3H, m, CHC**H**$_2$ and C**H**$_2$), 1.74 (1H, ddd, *J* = 14.3, 3.9, 2.9 Hz, C**H**$_2$), 1.61 (1H, br s, O**H**), 1.45 (1H, ddd, *J* =14.4, 13.4, 4.8 Hz, C**H**$_2$), 0.90 (3H, s, C**H**$_3$COH); $^{13}$C NMR (101 MHz, CDCl$_3$) δ 172.4 (C), 171.9 (C), 149.7 (C), 143.2 (C), 133.4 (C), 128.8 (2 × CH), 127.6 (2 × CH), 115.7 (CH$_2$), 69.7 (C), 55.5 (C), 52.9 (CH$_3$), 52.7 (CH$_3$), 46.8 (CH), 36.7 (CH$_2$), 33.6 (CH$_2$), 30.0 (CH$_3$), 26.5 (CH$_2$); HRMS (ESI) Exact mass calculated for $[C_{19}H_{24}ClO_5]^+$ $[M+H]^+$: 367.1307, found: 367.1307; Enantiomeric excess was determined by HPLC with a Chiralpak IC column (95:5 *iso*-hexane:*i*-PrOH, 1.0 mL/min, 210 nm, 25 °C) $t_r$ (minor) = 14.5 min, $t_r$ (major) = 28.9 min, 76% ee.

## Further Transformations

### (3*S*,4*R*)-3-Hydroxy-3-phenyl-4-(1-phenylvinyl)pyrrolidin-2-one (2r)

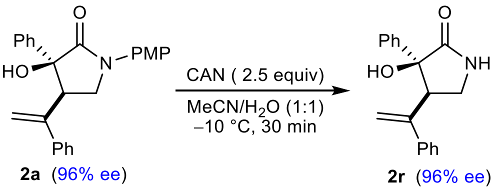


To a solution of pyrrolidin-2-one **2a** (77.0 mg, 0.20 mmol, 96% ee) in MeCN (4 mL) at −10 °C was added a solution of CAN (274 mg, 0.50 mmol) in $H_2O$ (4 mL) dropwise. The reaction was stirred for 30 min at this temperature, diluted with $H_2O$ (20 mL), and extracted with EtOAc (2 × 15 mL). The combined organic layers were dried ($Na_2SO_4$), filtered, and concentrated *in vacuo*. Purification of the residue by column chromatography (8% MeOH/$CH_2Cl_2$) gave the *lactam* **2r** (54.7 mg, 98%) as a white solid that displayed spectrosocopic data consistent with those reported above. $[\alpha]_D^{25}$ +32.0 (*c* 0.25, $(CH_3)_2CO$). Enantiomeric excess was determined by HPLC with a Chiralpak IC column (70:30 *iso*-hexane:*i*-PrOH, 1.0 mL/min, 254 nm, 25 °C); $t_r$ (major) = 10.5 min, $t_r$ (minor) = 18.8 min, 96% ee.

### (3*S*,4*R*)-4-Benzoyl-3-hydroxy-1-(4-methoxyphenyl)-3-phenylpyrrolidin-2-one (12)

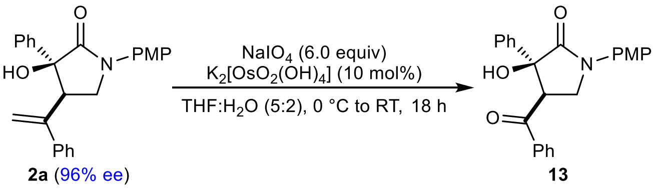


To a mixture of **2a** (77.0 mg, 0.200 mmol) and $NaIO_4$ (257 mg, 1.20 mmol) in THF (4 mL) and $H_2O$ (1.6 mL) at 0 °C was added $K_2[OsO_2(OH)_4]$ (7.4 mg, 0.020 mmol) in one portion and the mixture was stirred for 3 h at 0 °C, and then allowed to stand for 18 h at room temperature. The mixture was filtered to remove the white solid, and the filtrate was concentrated *in vacuo*. EtOAc (30 mL) was

added and the mixture was washed with $H_2O$ (2 × 15 mL), saturated aqueous $NaHCO_3$ solution (15 mL), and brine (15 mL), and then dried ($Na_2SO_4$), filtered, and concentrated *in vacuo*. Purification of the residue by column chromatography (40% EtOAc/cyclohexane) gave the *ketone* **12** (70.6 mg, 91%) as a white solid. $R_f$ = 0.38 (40% EtOAc/cyclohexane); m.p. 160−162 °C ($Et_2O$); $[\alpha]_D^{25}$ −22.6 (*c* 0.53, $CHCl_3$); IR 3377, 2981, 1675 (C=O), 1511, 1246, 1224, 1180, 1030, 829, 687, 623, 522 $cm^{-1}$; $^1H$ NMR (400 MHz, $CDCl_3$) δ 7.90–7.84 (2H, m, Ar**H**), 7.72–7.65 (2H, m, Ar**H**), 7.62–7.55 (1H, m, Ar**H**), 7.53–7.34 (7H, m, Ar**H**), 7.02–6.94 (2H, m, Ar**H**), 4.46 (1H, dd, *J* = 7.0, 2.8 Hz, NC**H**$_2$), 4.36 (1H, dd, *J* = 10.0, 2.8 Hz, NC**H**$_2$), 3.96 (1H, dd, *J* = 10.0, 6.9 Hz, C**H**$CH_2$), 3.84 (3H, s, OC**H**$_3$), 3.48 (1H, s, O**H**); $^{13}C$ NMR (101 MHz, $CDCl_3$) δ 197.0 (C), 172.0 (C), 157.6 (C), 142.2 (C), 137.3 (C), 133.7 (CH), 131.8 (C), 129.1 (2 × CH), 128.74 (2 × CH), 128.69 (2 × CH), 128.63 (CH), 125.0 (2 × CH), 122.5 (2 × CH), 114.5 (2 × CH), 80.6 (C), 55.7 ($CH_3$), 50.5 (CH), 47.9 ($CH_2$); HRMS (ESI) Exact mass calcd for $[C_{24}H_{21}NO_4Na]^+$ $[M + Na]^+$: 410.1363, found: 410.1359.

## NMR Spectra

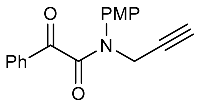


**S2**

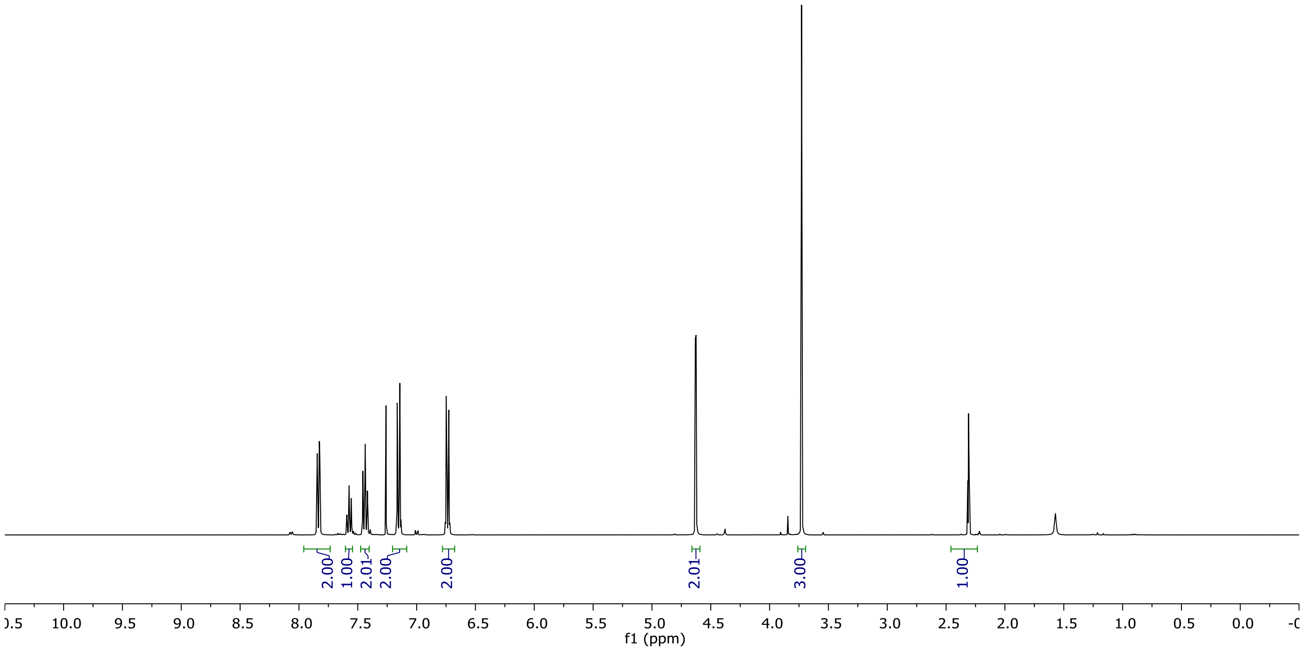


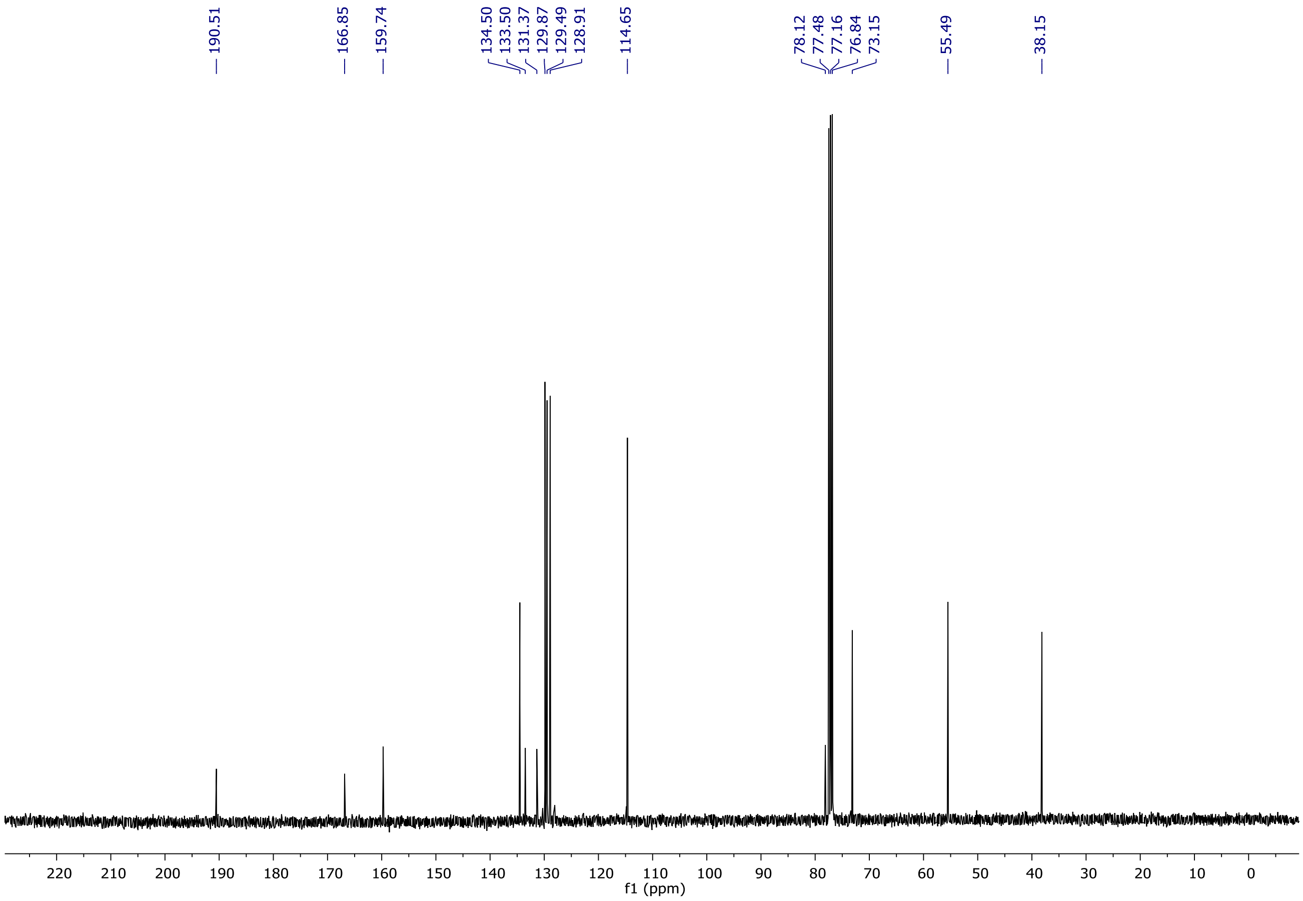

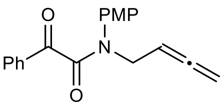


**1a**
10:1 mixture of rotamers

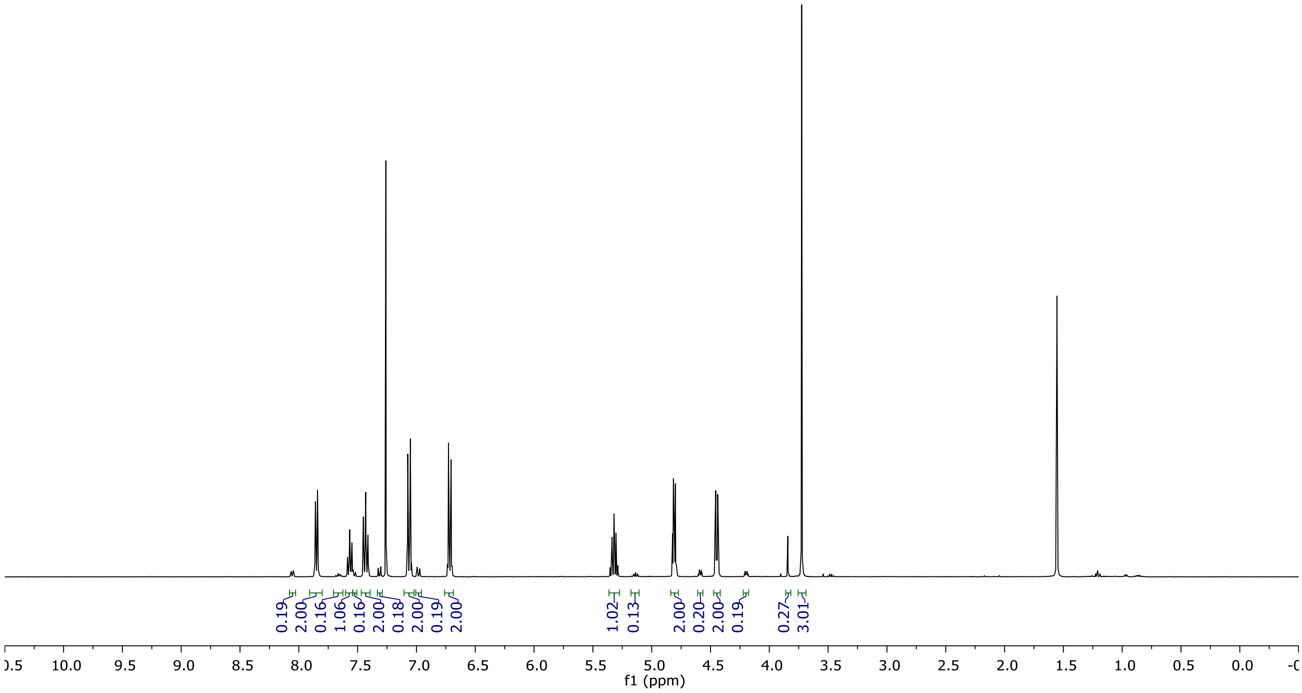


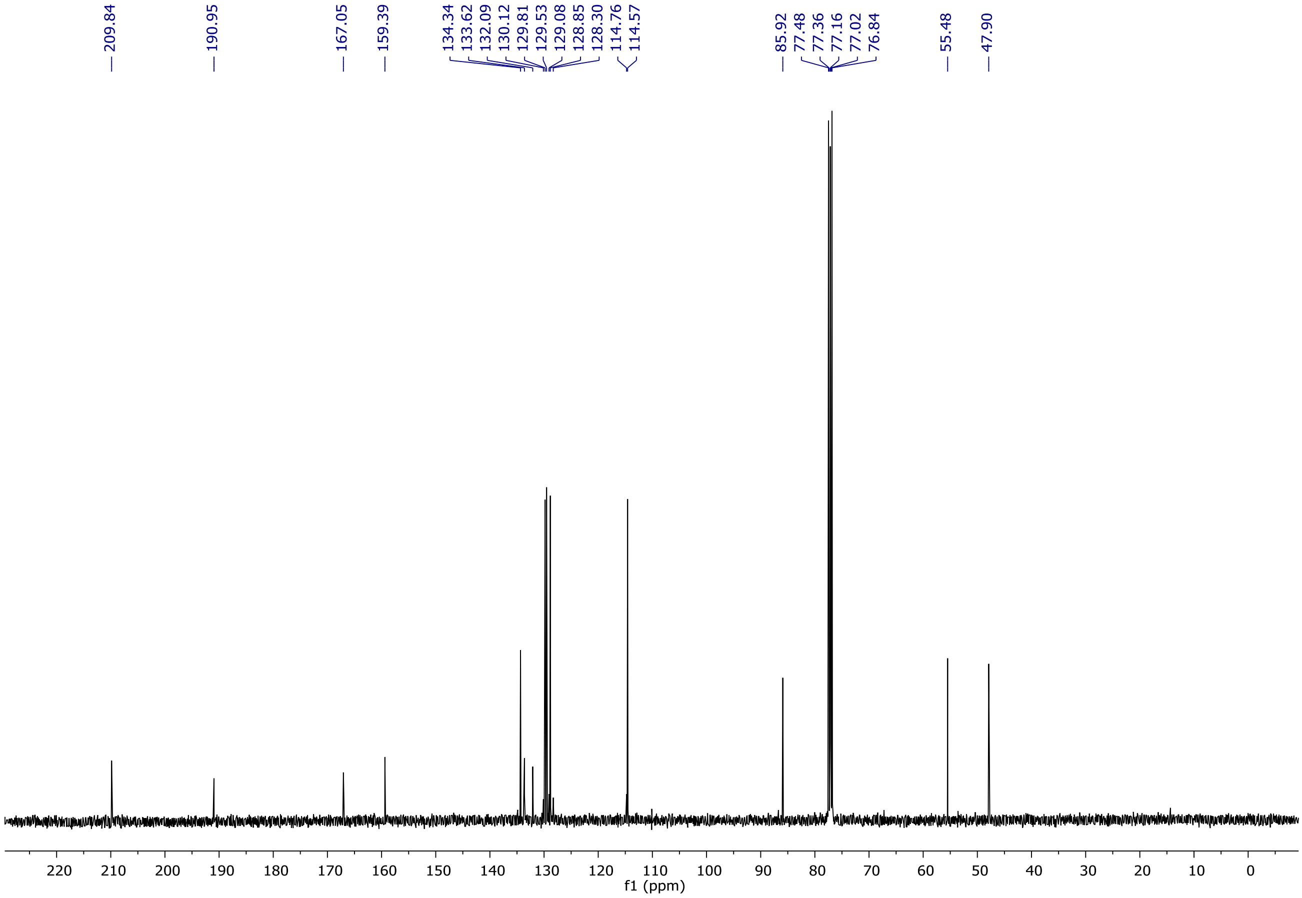

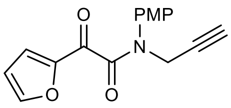
O
PMP
N
O
O

**S3**

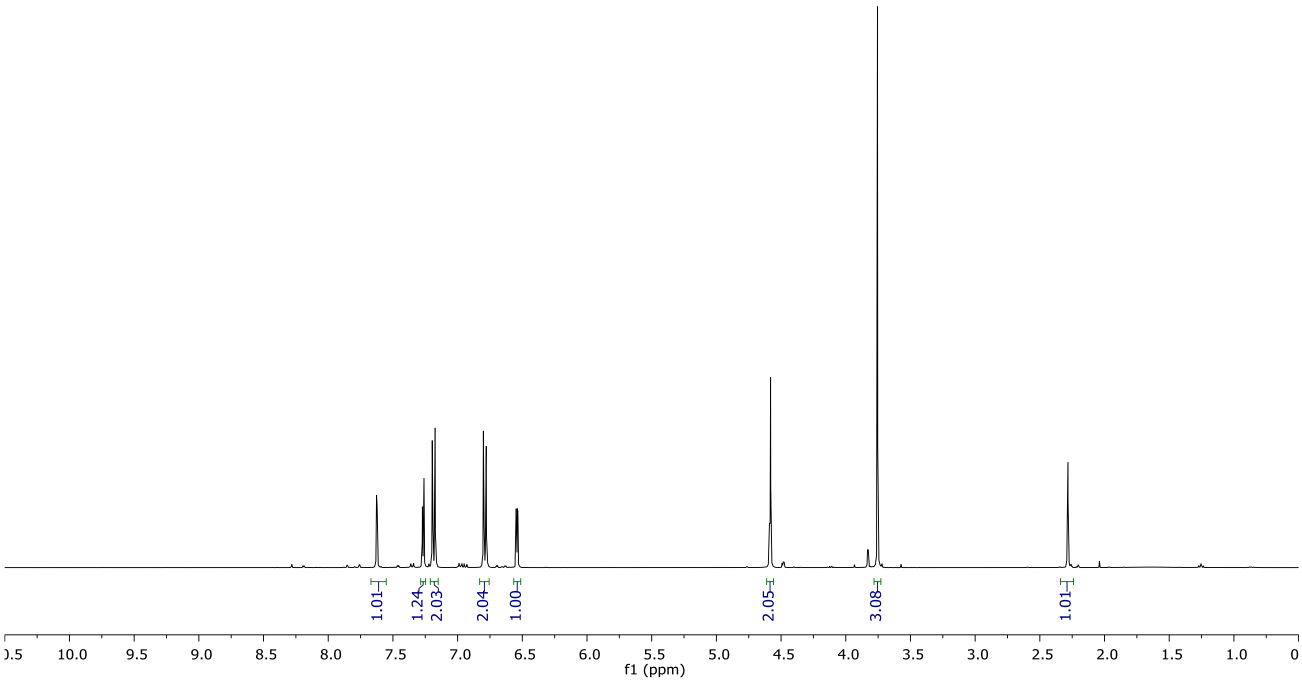
1.01
1.24
2.03
2.04
1.00
2.05
3.08
1.01
f1 (ppm)

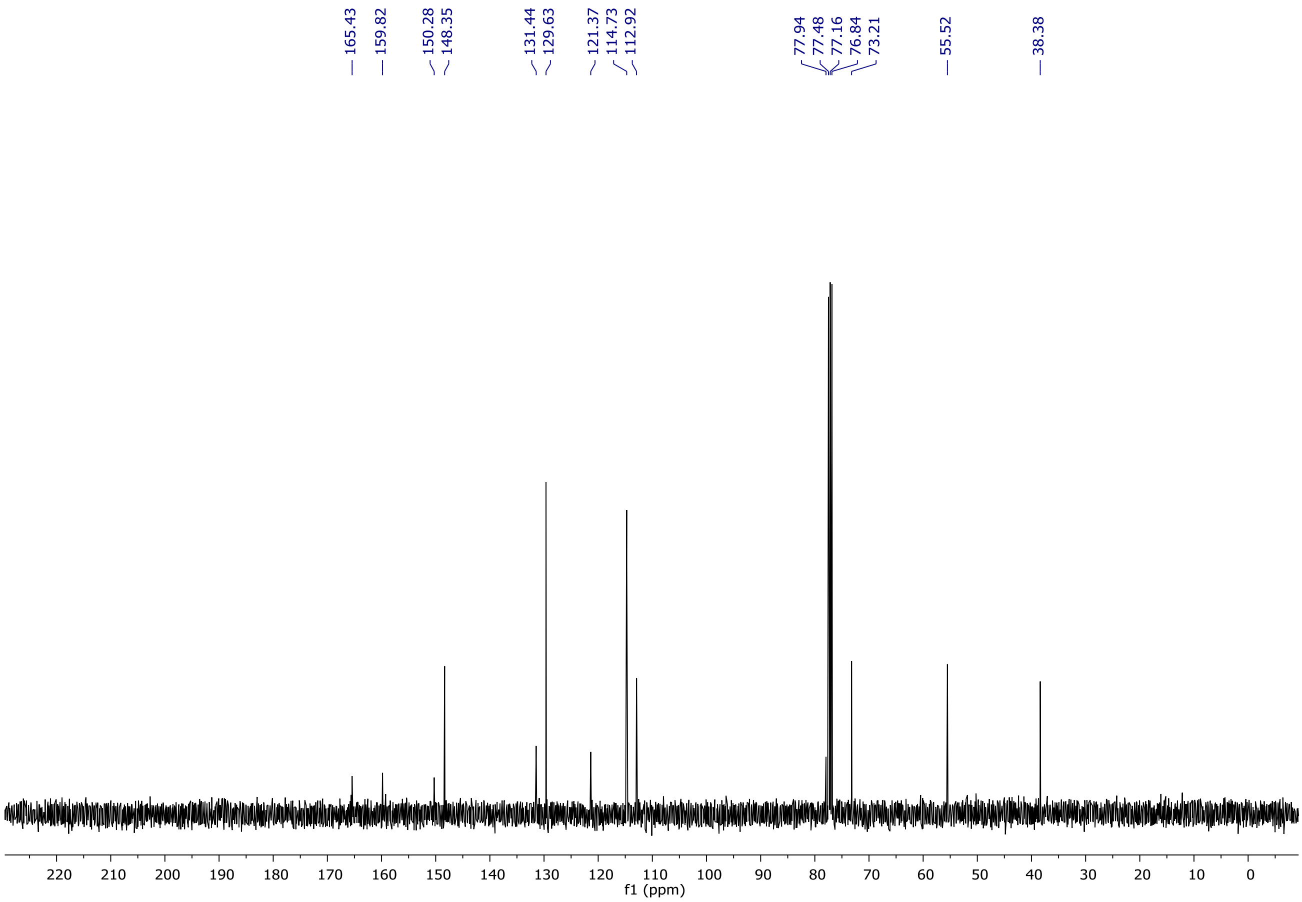
165.43
159.82
150.28
148.35
131.44
129.63
121.37
114.73
112.92
77.94
77.48
77.16
76.84
73.21
55.52
38.38
f1 (ppm)

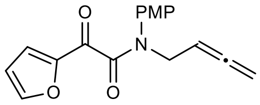


**1b**
10:1 mixture of rotamers

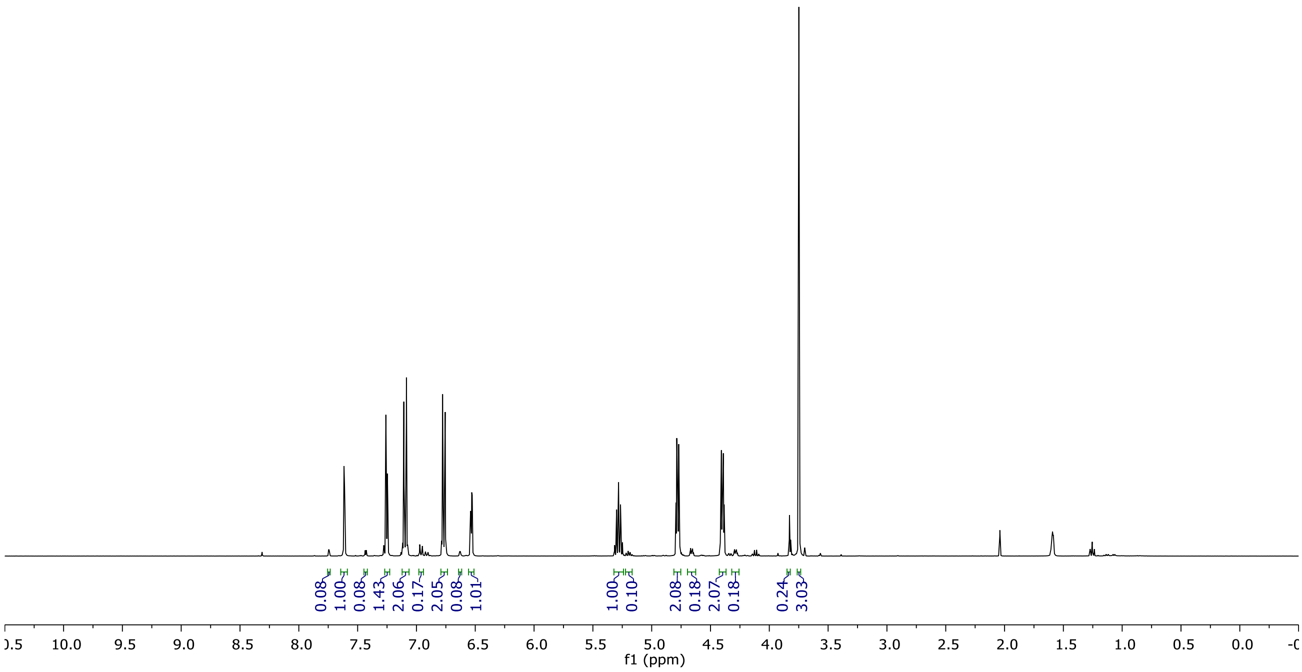


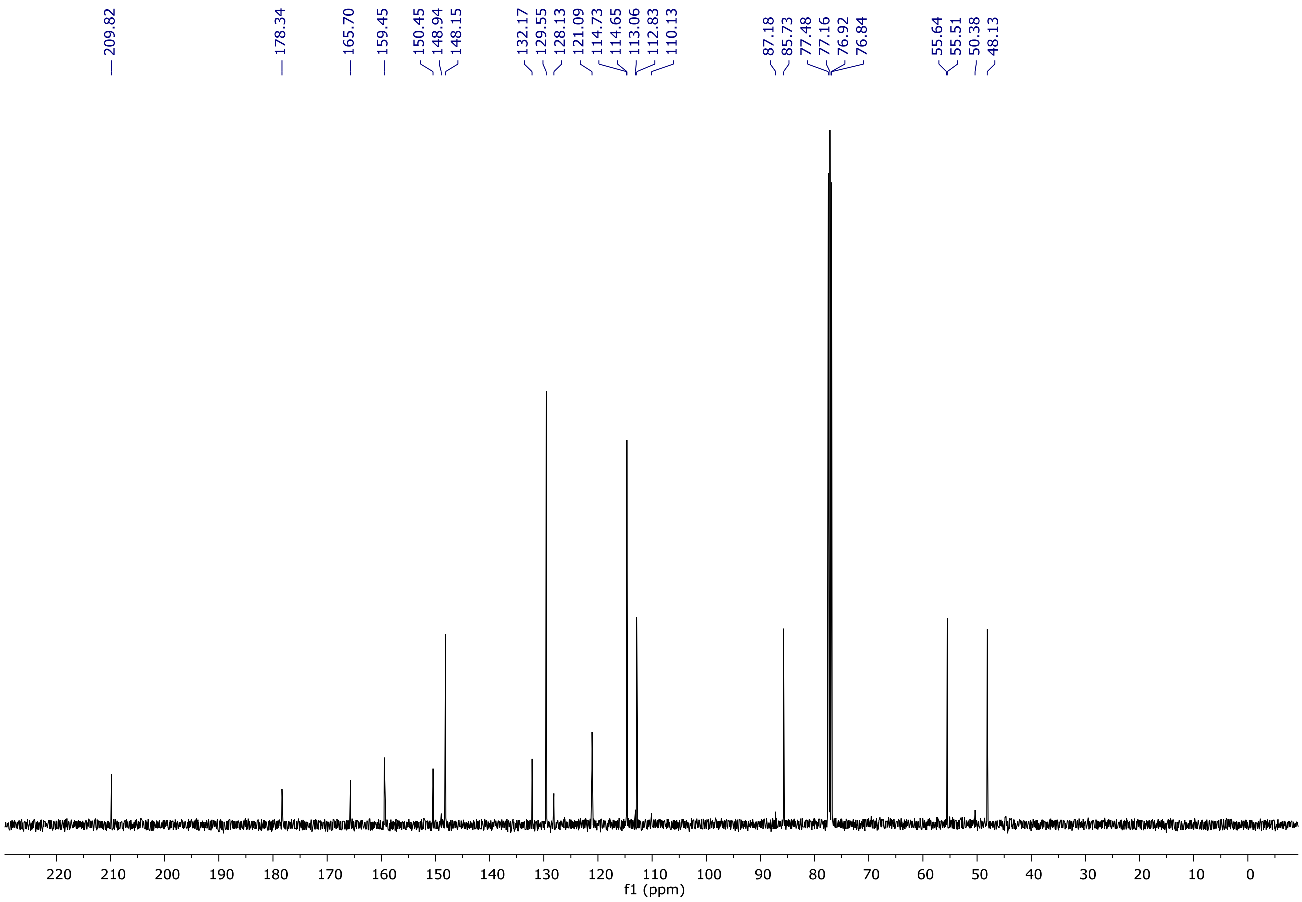

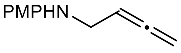
PMPHN

**S5**

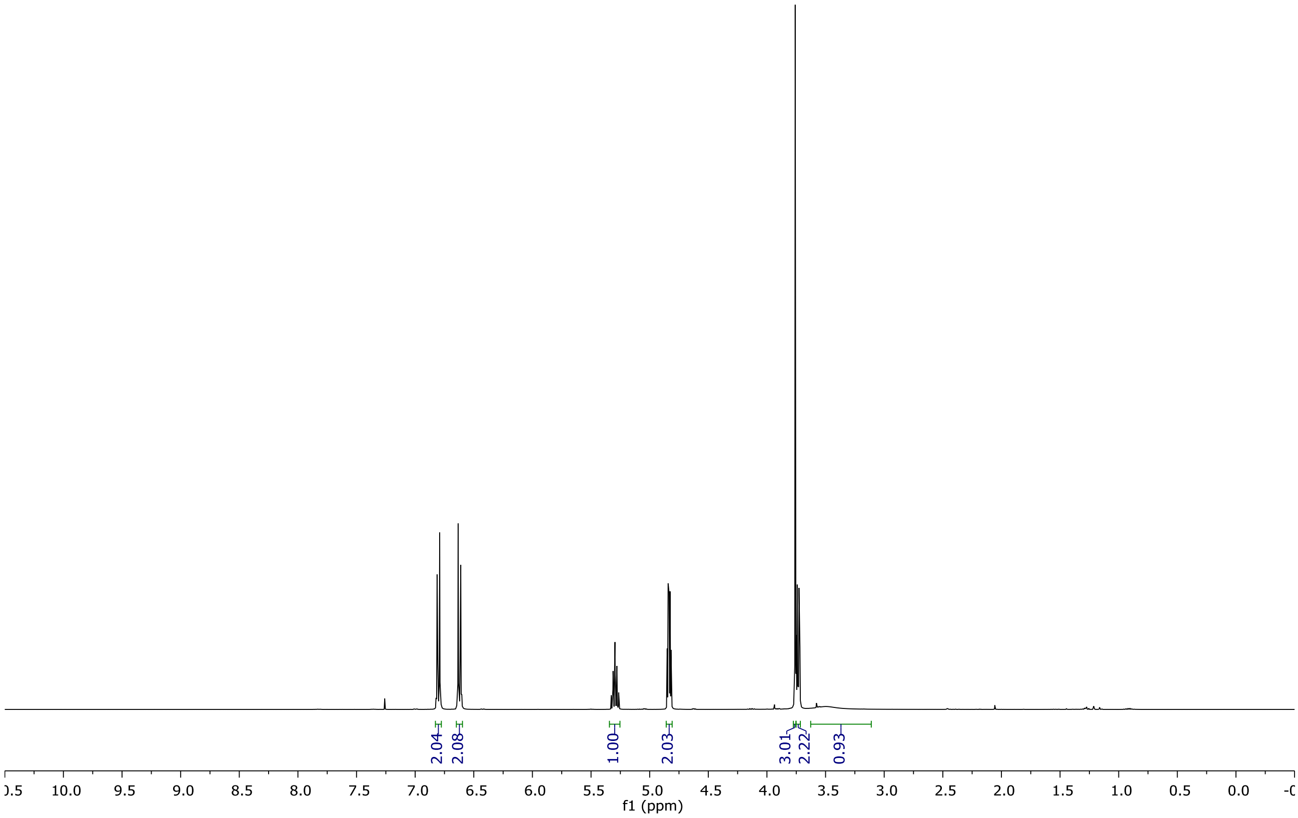
2.04
2.08
1.00
2.03
3.01
2.22
0.93
f1 (ppm)

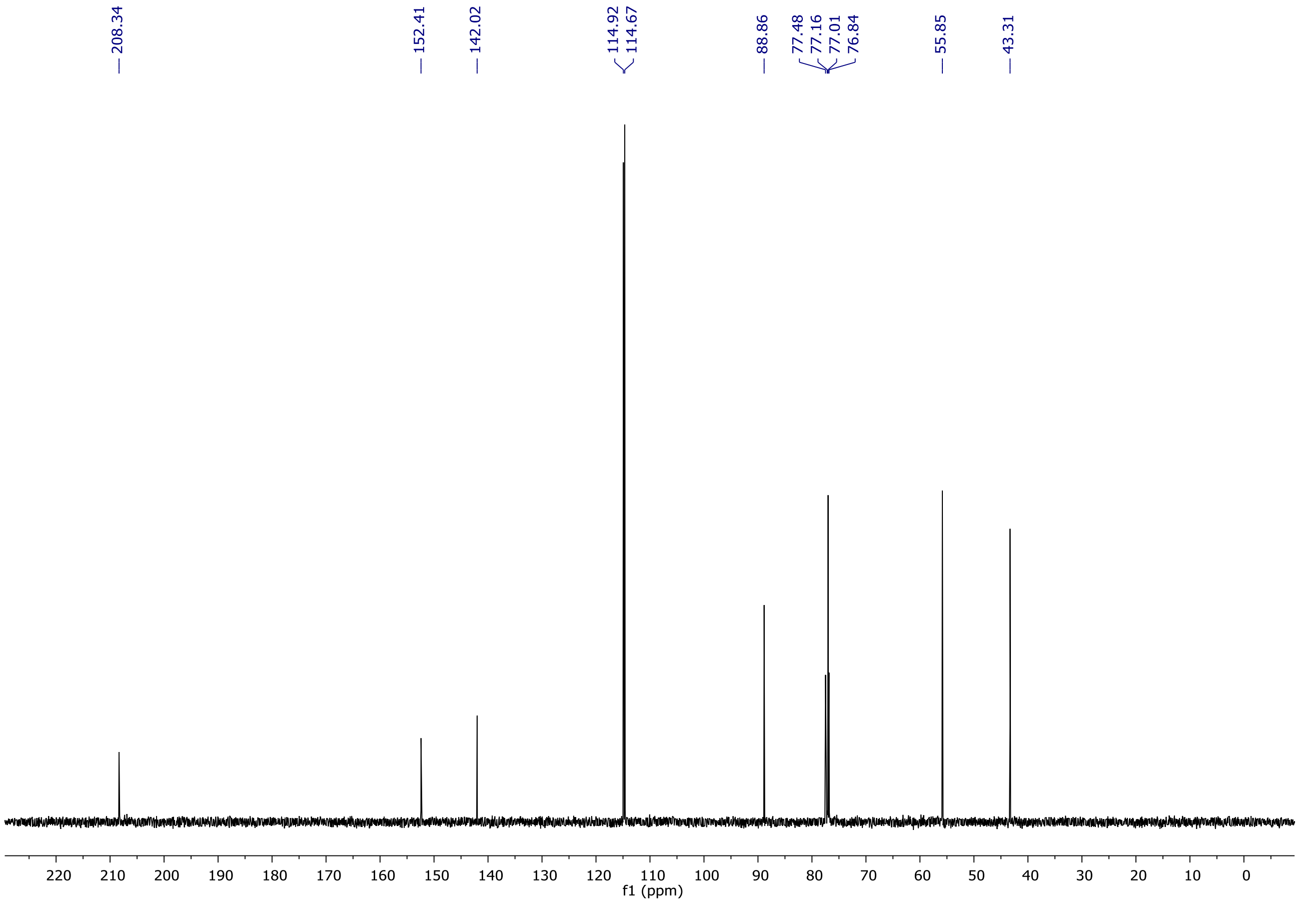
208.34
152.41
142.02
114.92
114.67
88.86
77.48
77.16
77.01
76.84
55.85
43.31
f1 (ppm)

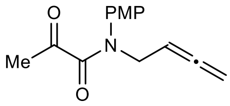


**1c**
14:1 mixture of rotamers

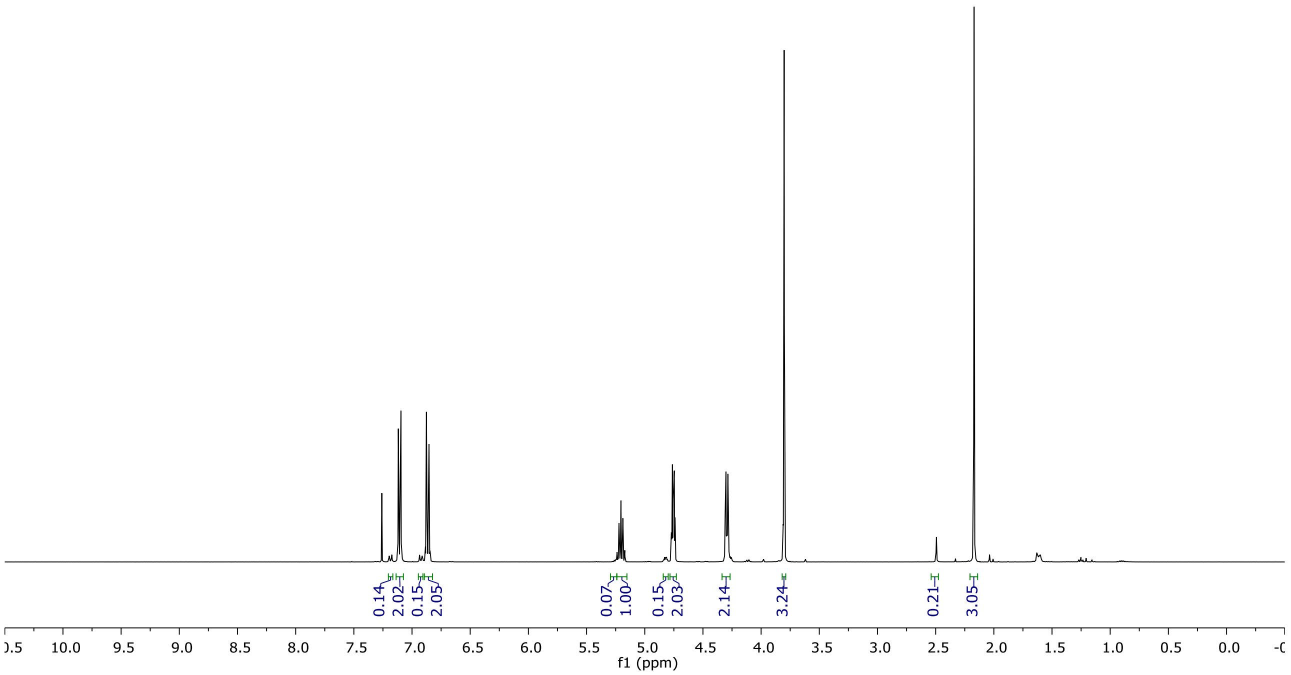


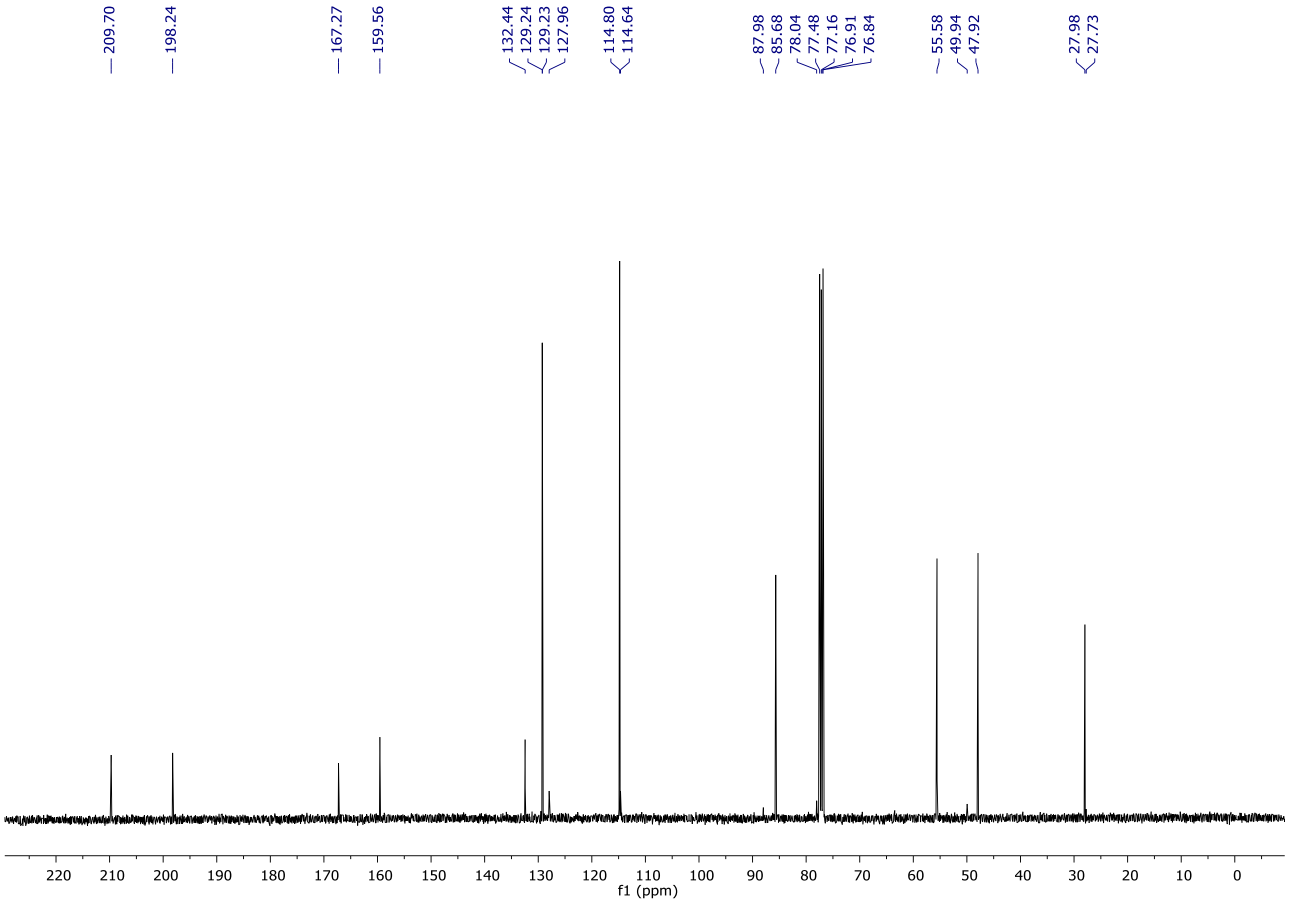

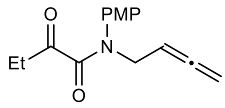


**1d**
14:1 mixture of rotamers

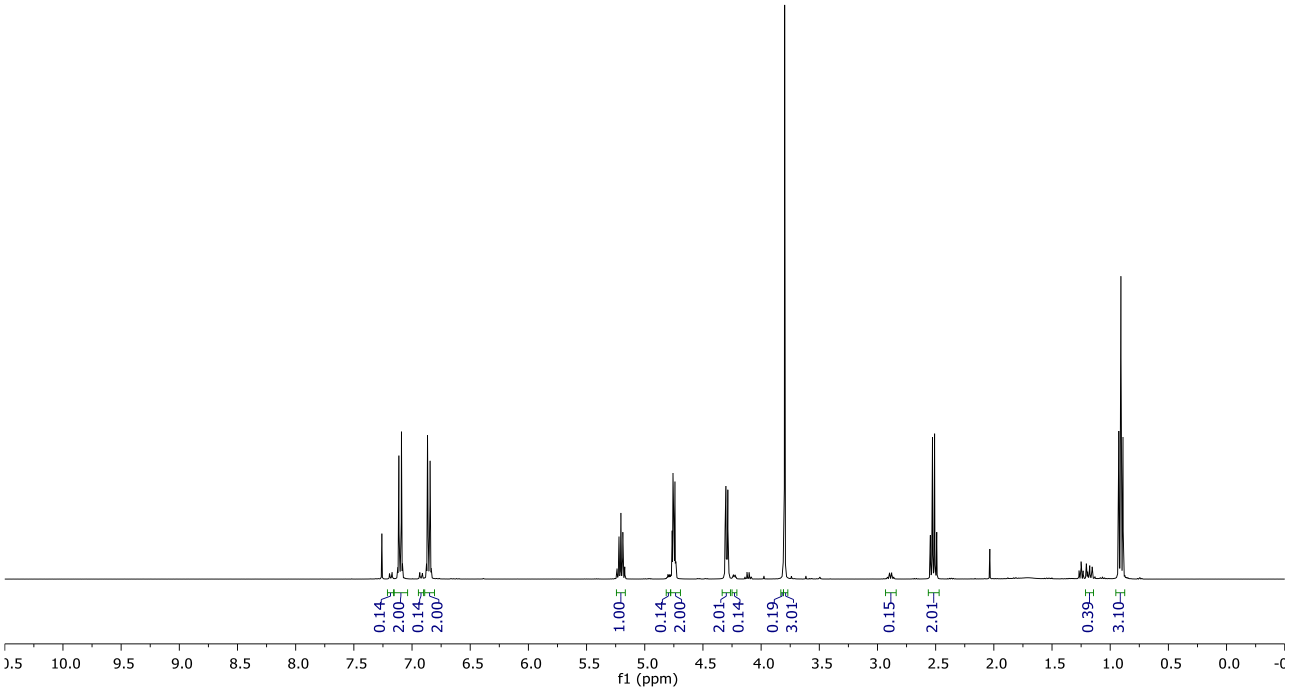


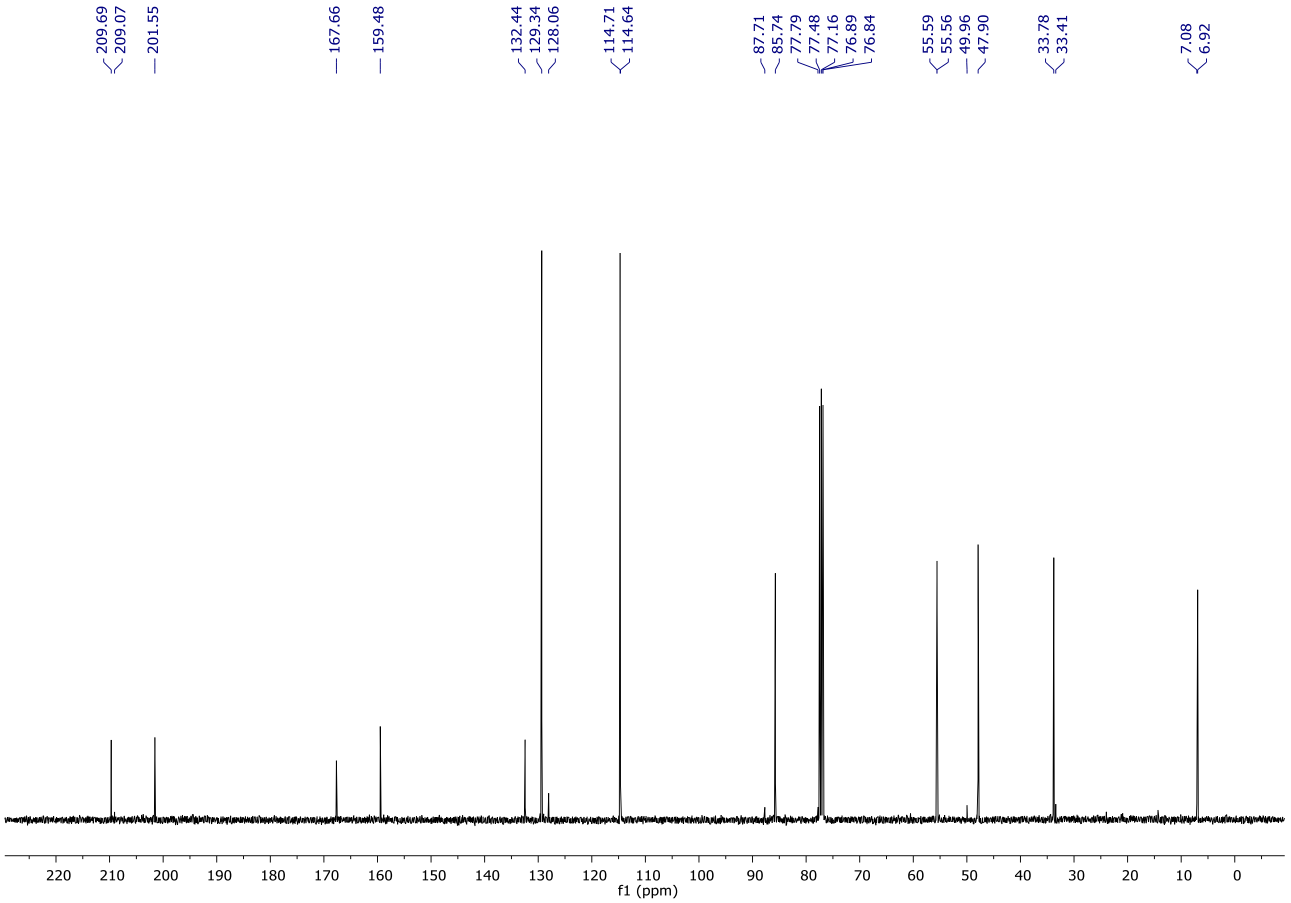

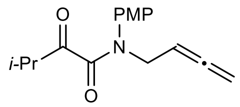


**1e**
14:1 mixture of rotamers

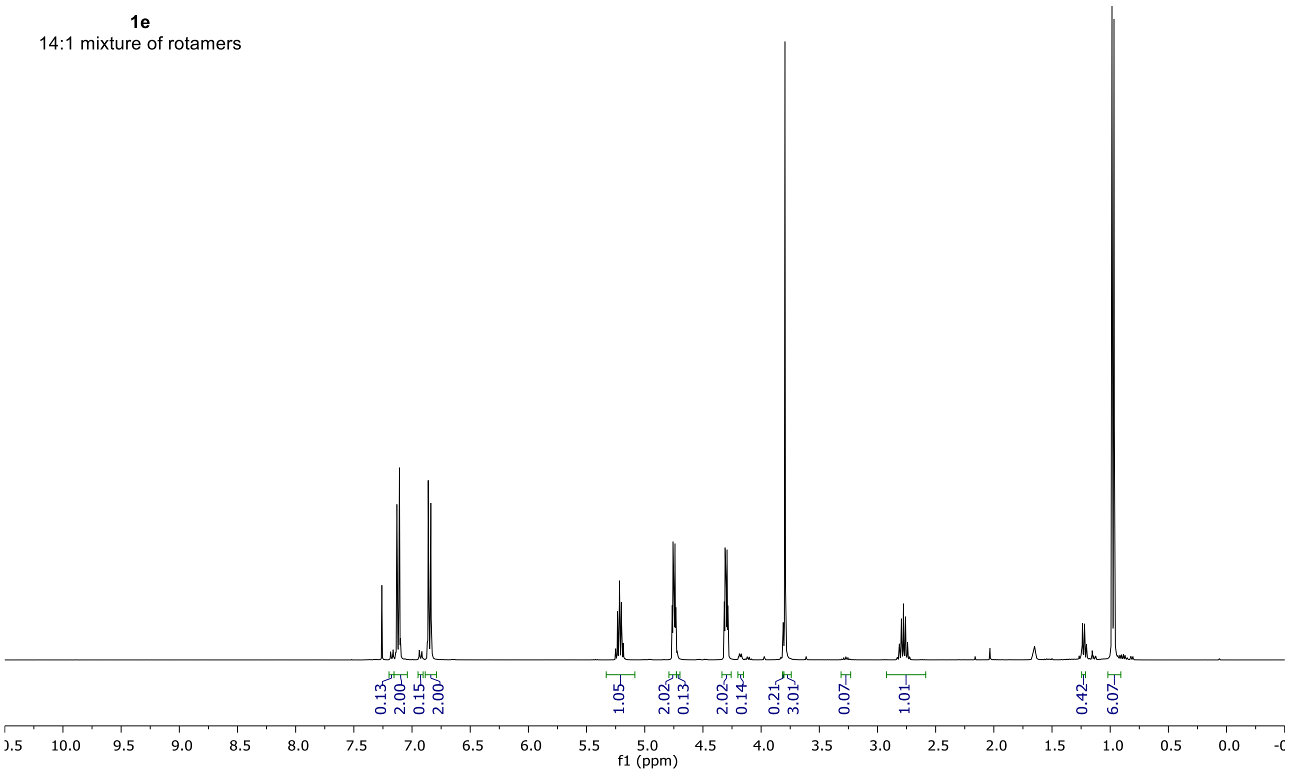


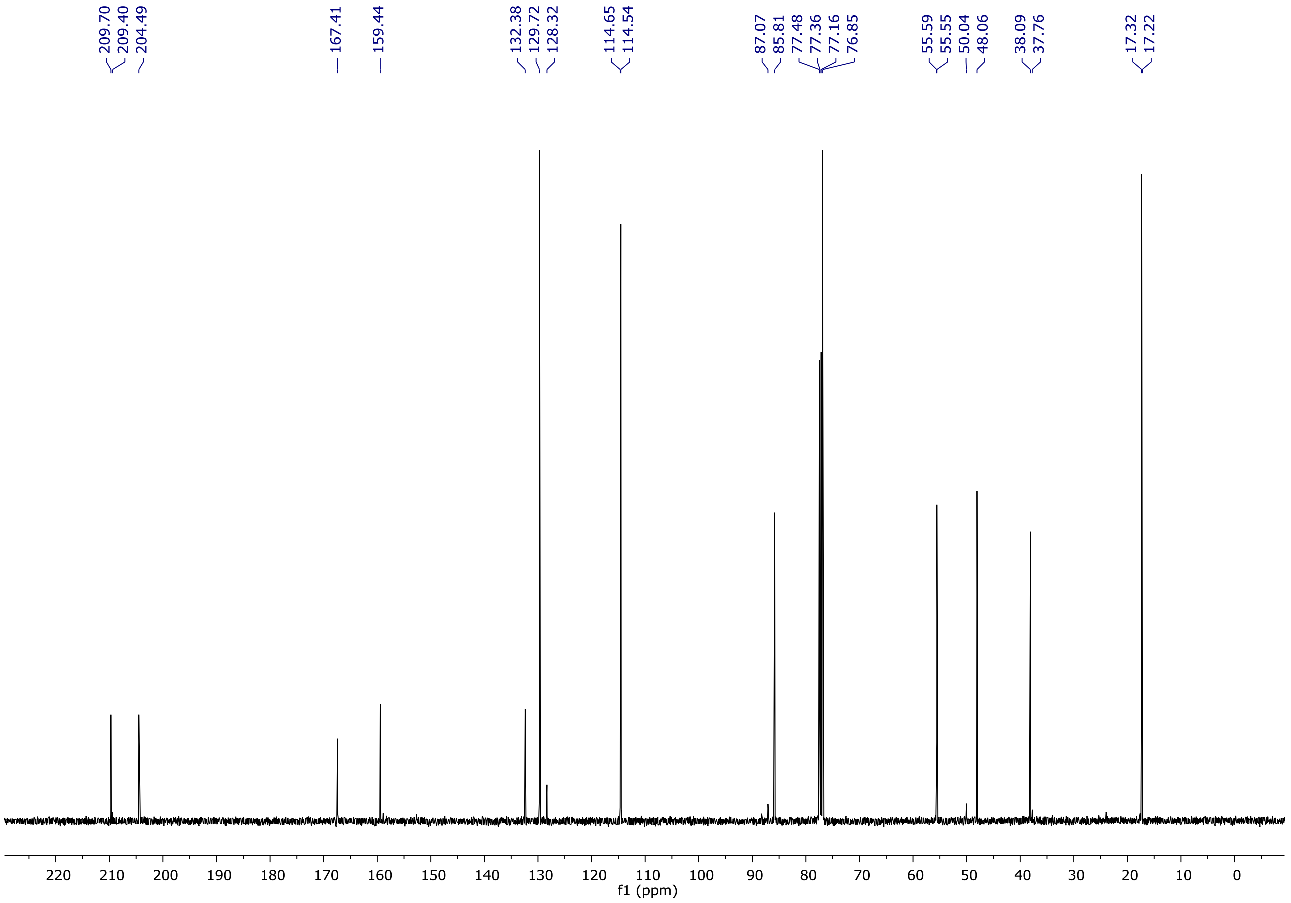

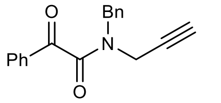


**S7**
1.7:1 mixture of rotamers

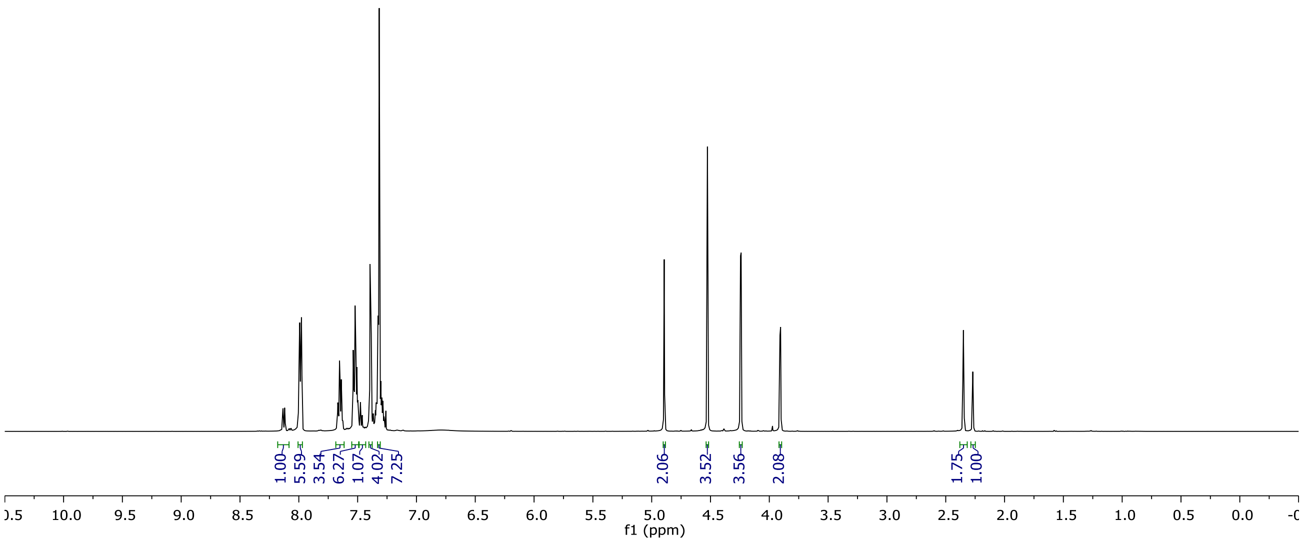


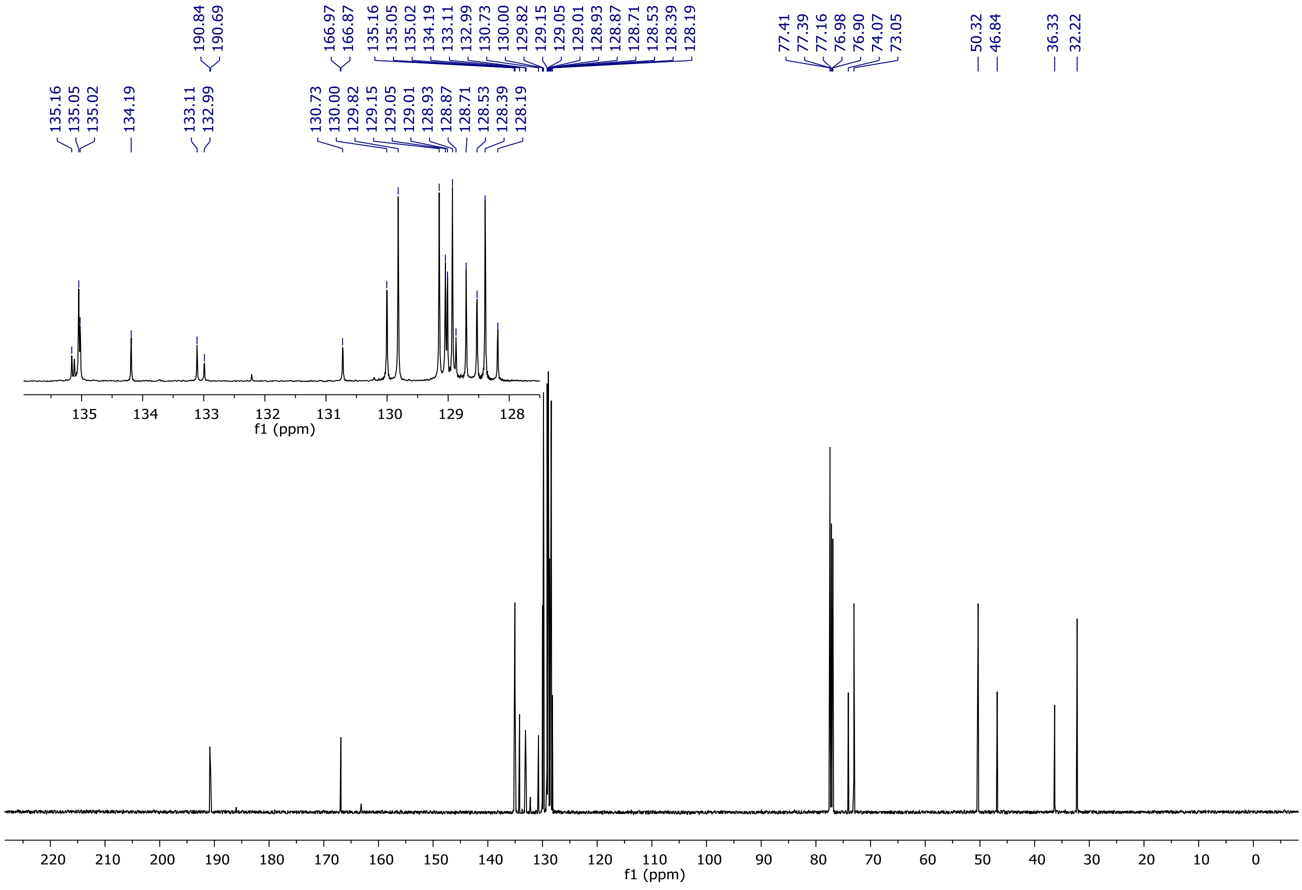

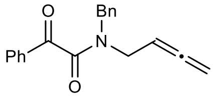
O
Bn
N
Ph
O

**1f**

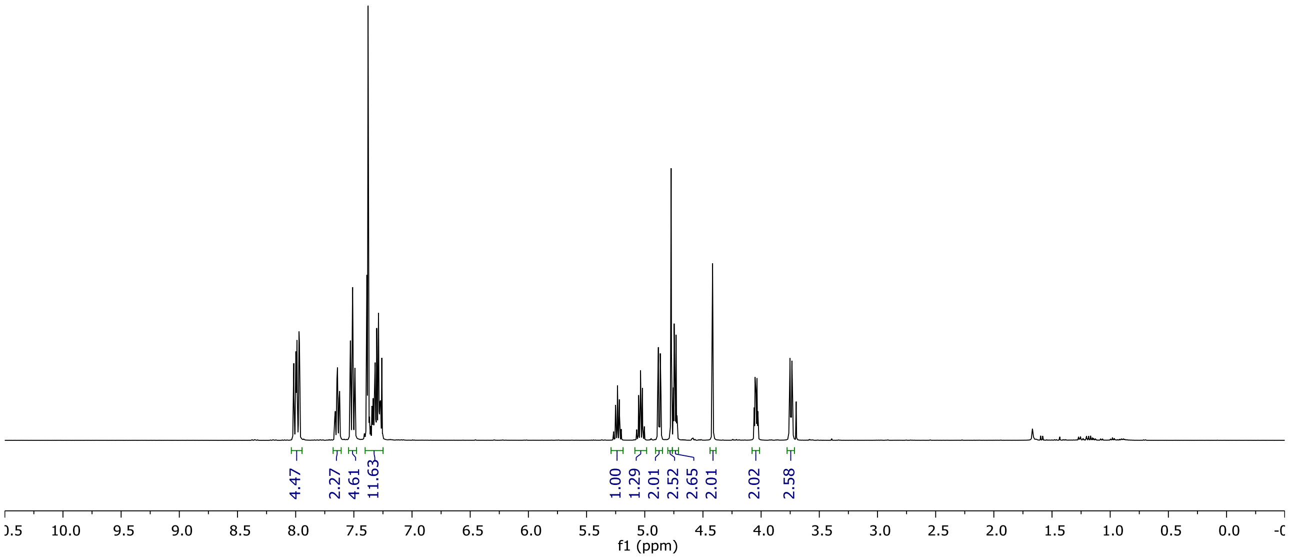
4.47
2.27
4.61
11.63
1.00
1.29
2.01
2.52
2.65
2.01
2.02
2.58
f1 (ppm)

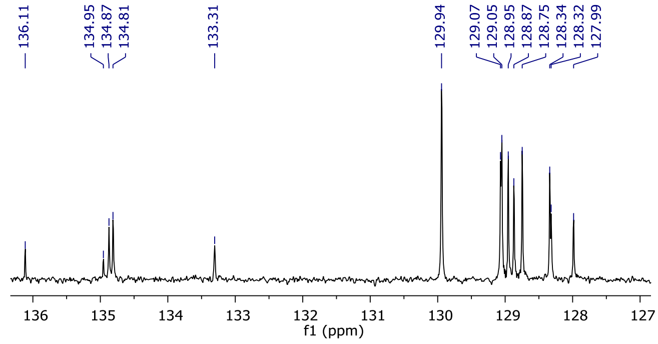
136.11
134.95
134.87
134.81
133.31
129.94
129.07
129.05
128.95
128.87
128.75
128.34
128.32
127.99
f1 (ppm)

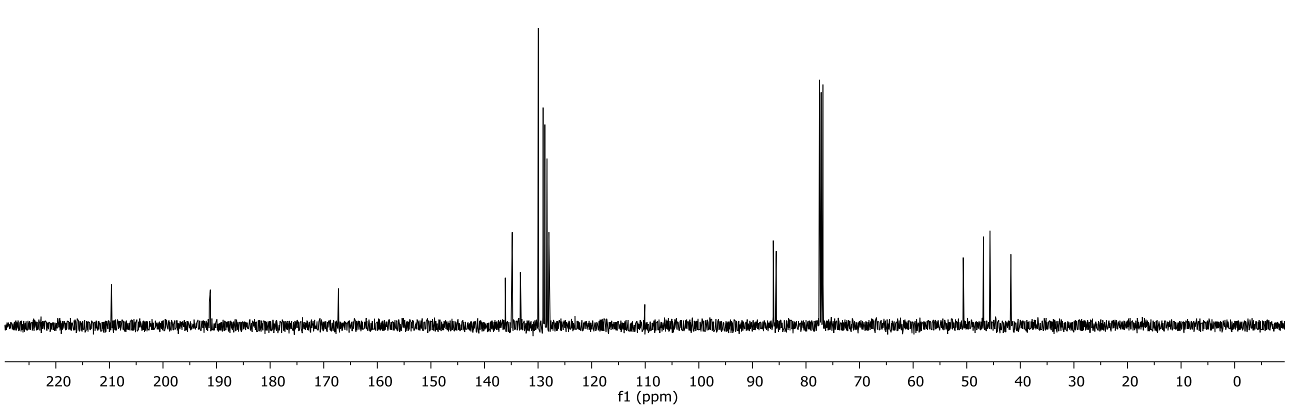
209.65
209.61
191.35
191.16
167.26
167.21
136.11
134.95
134.87
134.81
133.31
129.94
129.07
129.05
128.95
128.87
128.75
128.34
128.32
127.99
110.09
86.10
85.55
77.48
77.36
77.22
77.16
76.84
50.65
46.88
45.66
41.76
f1 (ppm)

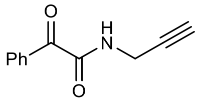
O
H
N
Ph
O

**S8**

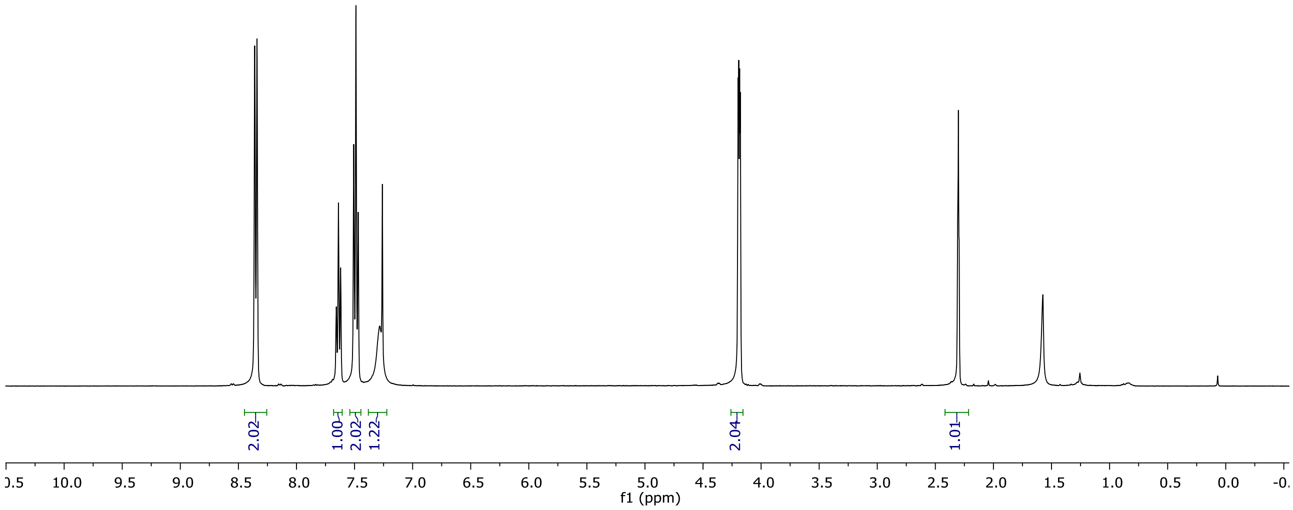
2.02
1.00
2.02
1.22
2.04
1.01
f1 (ppm)

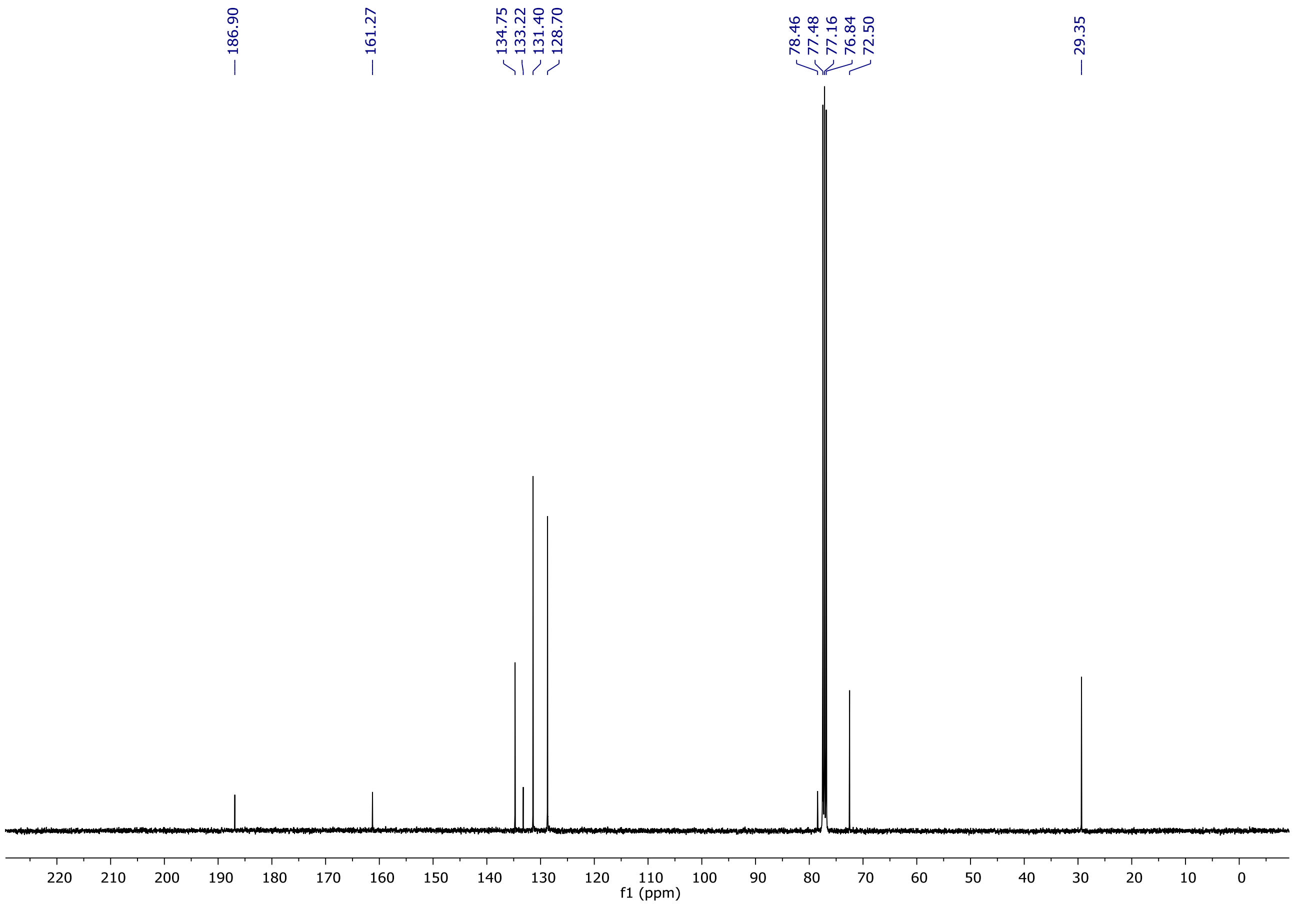
186.90
161.27
134.75
133.22
131.40
128.70
78.46
77.48
77.16
76.84
72.50
29.35
f1 (ppm)

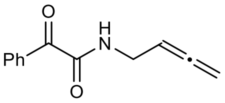
O
H
N
Ph
O

**1g**

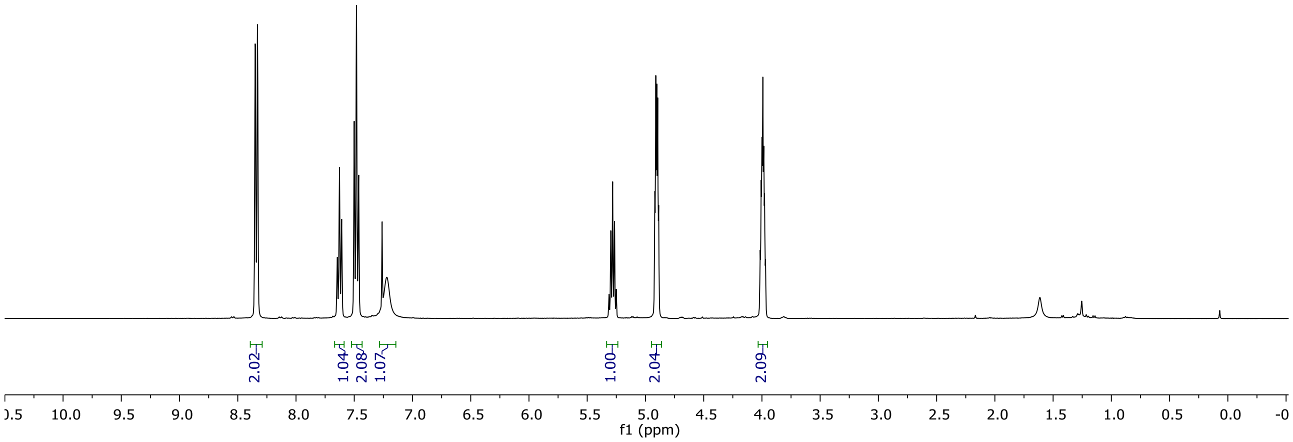
2.02
1.04
2.08
1.07
1.00
2.04
2.09
f1 (ppm)

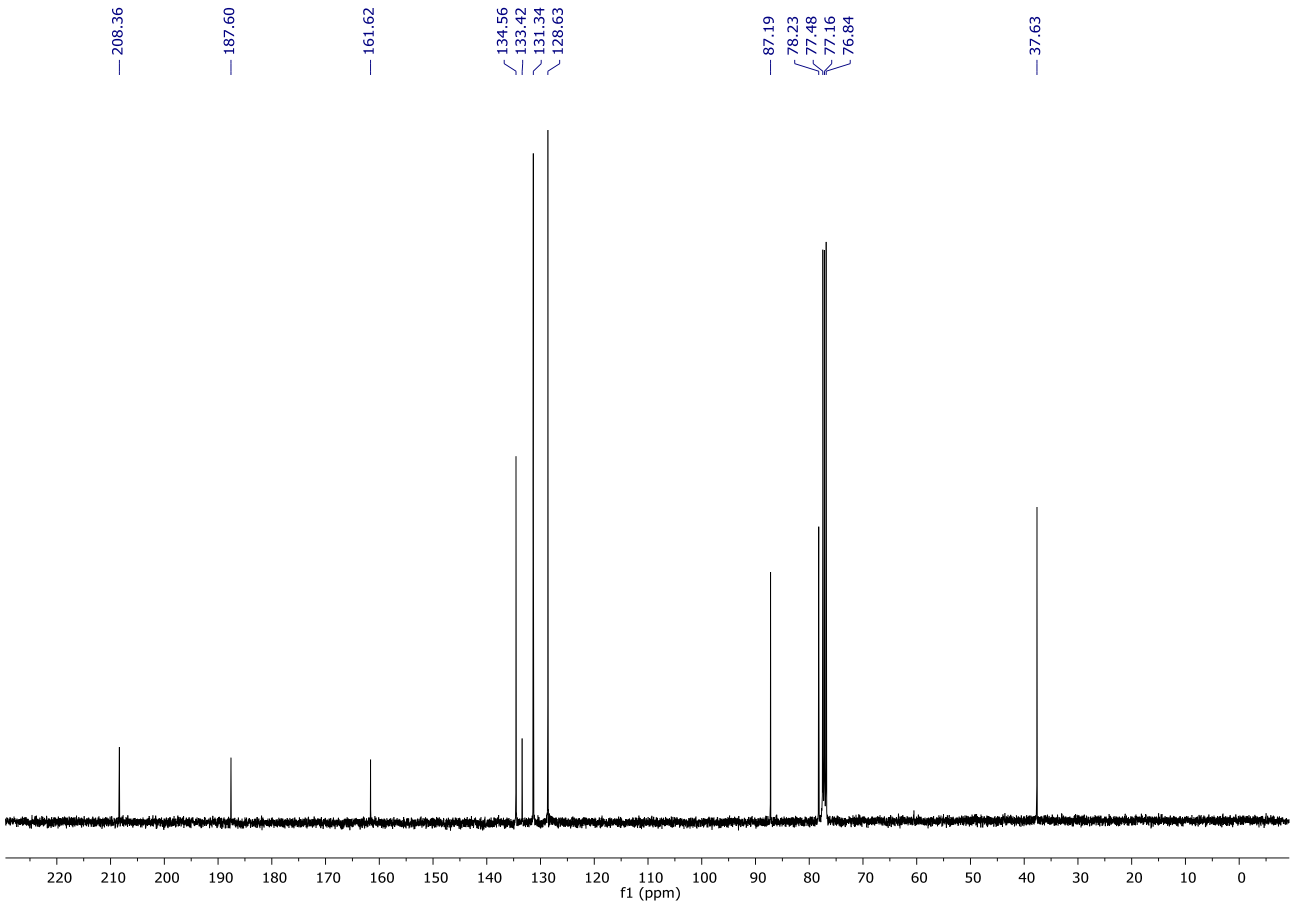
208.36
187.60
161.62
134.56
133.42
131.34
128.63
87.19
78.23
77.48
77.16
76.84
37.63
f1 (ppm)

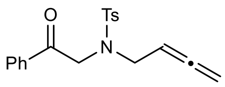
O
Ts
N
Ph

**4a**

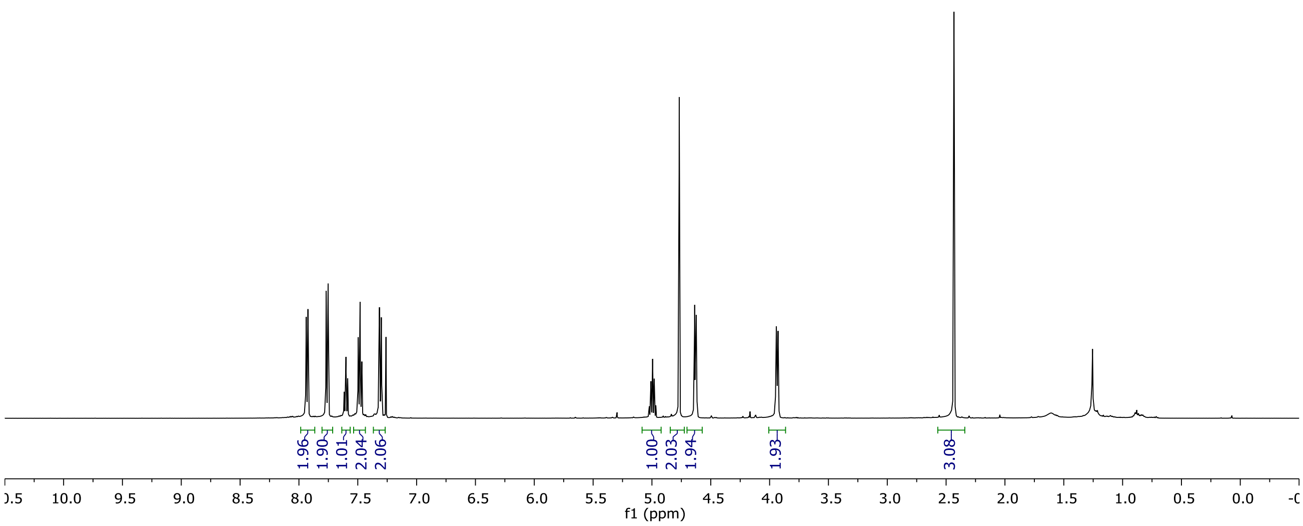
1.96
1.90
1.01
2.04
2.06
1.00
2.03
1.94
1.93
3.08
f1 (ppm)

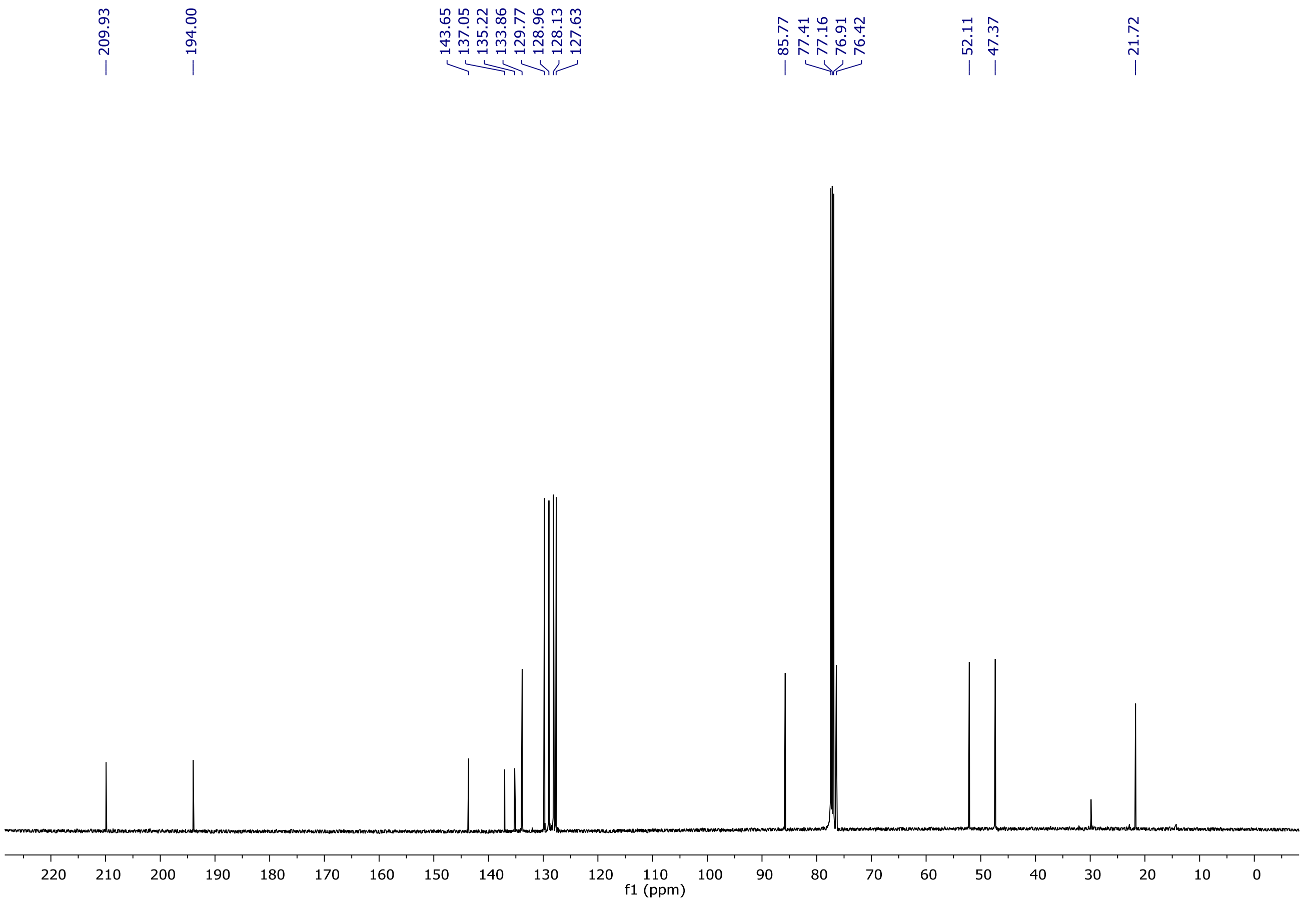
209.93
194.00
143.65
137.05
135.22
133.86
129.77
128.96
128.13
127.63
85.77
77.41
77.16
76.91
76.42
52.11
47.37
21.72
f1 (ppm)

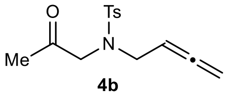
O
Ts
N
Me

**4b**

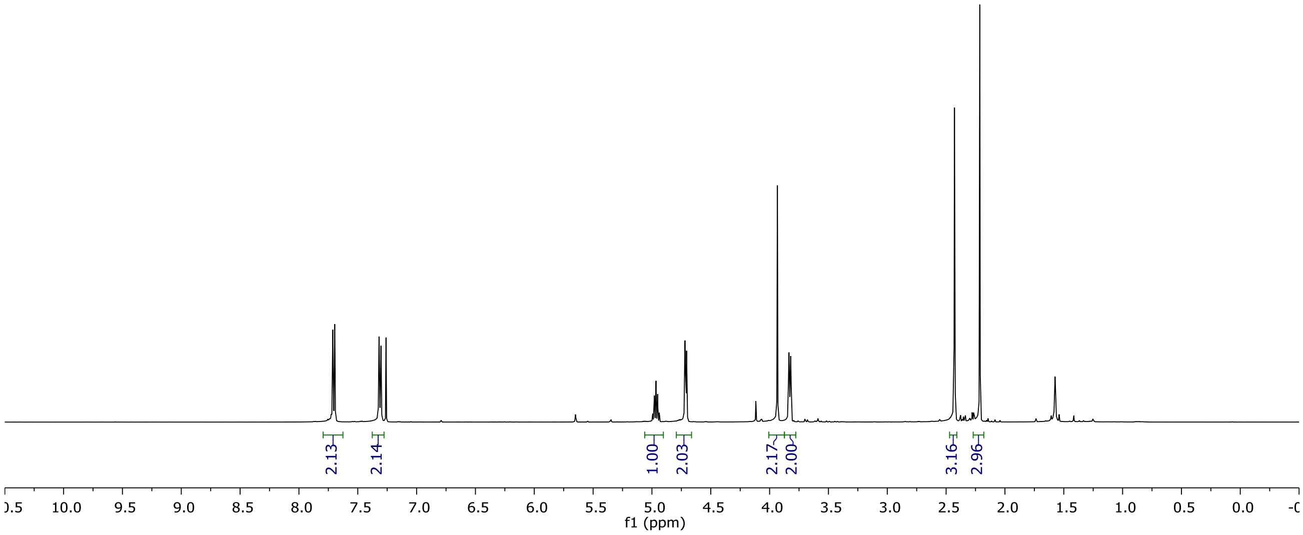
2.13
2.14
1.00
2.03
2.17
2.00
3.16
2.96
f1 (ppm)

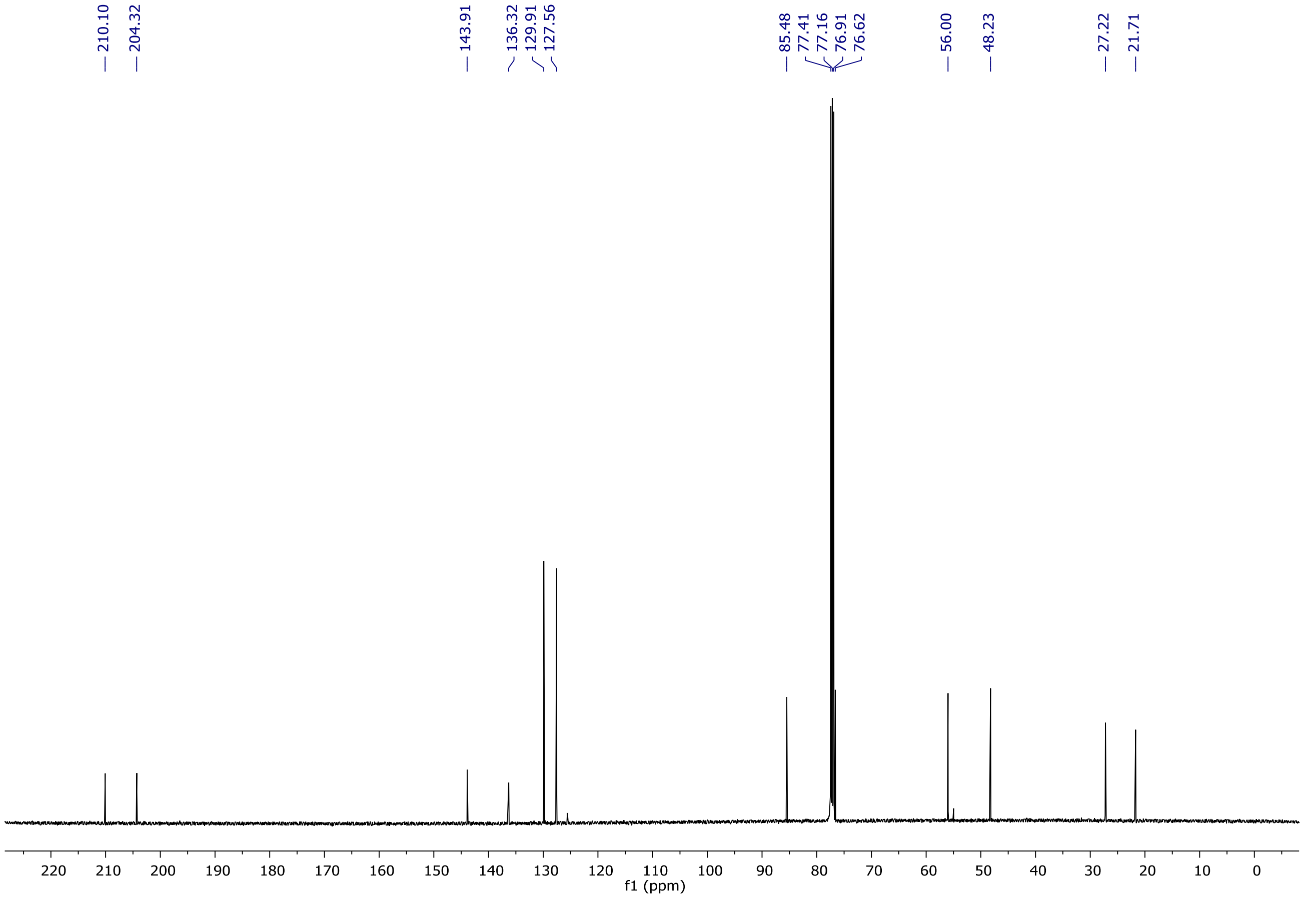
210.10
204.32
143.91
136.32
129.91
127.56
85.48
77.41
77.16
76.91
76.62
56.00
48.23
27.22
21.71
f1 (ppm)

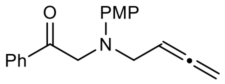
O
PMP
N
Ph

**4c**

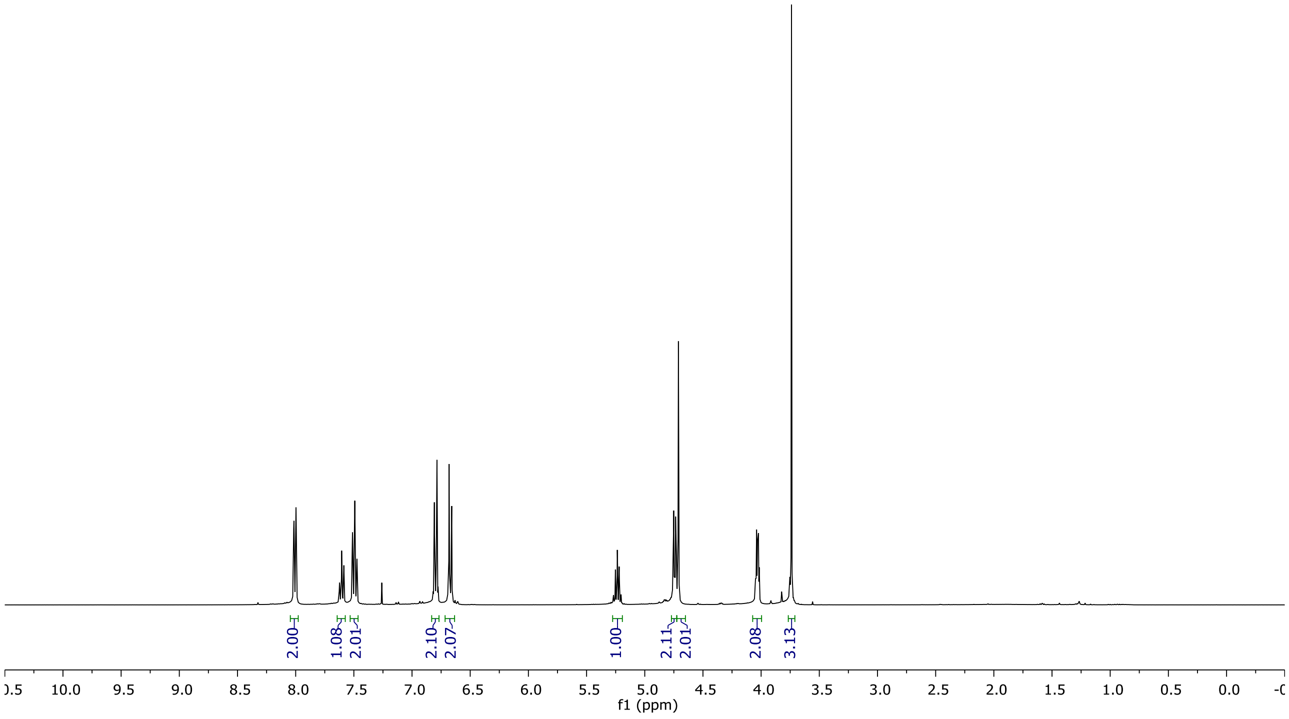
2.00
1.08
2.01
2.10
2.07
1.00
2.11
2.01
2.08
3.13
f1 (ppm)

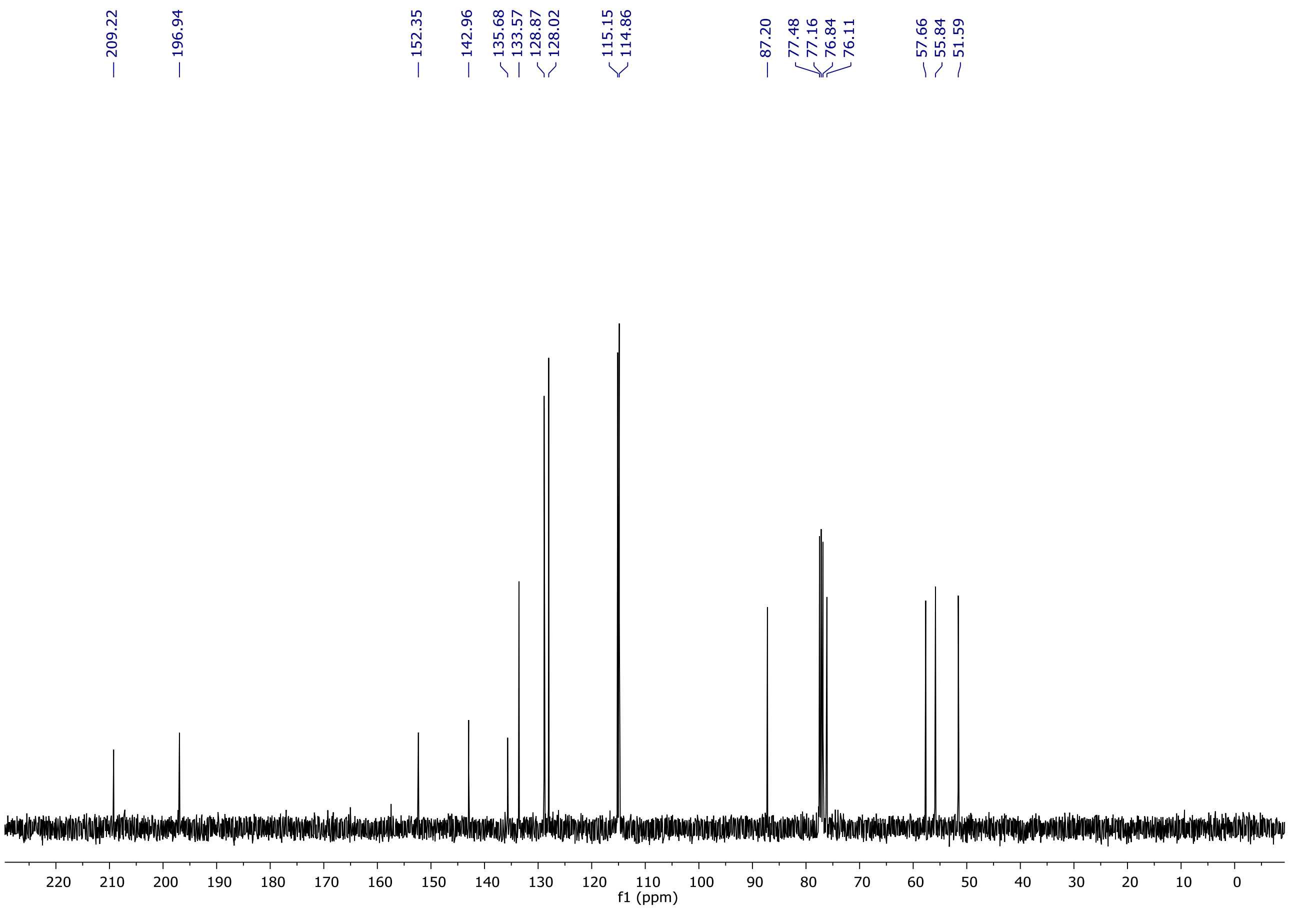
209.22
196.94
152.35
142.96
135.68
133.57
128.87
128.02
115.15
114.86
87.20
77.48
77.16
76.84
76.11
57.66
55.84
51.59
f1 (ppm)

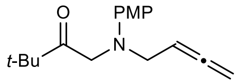
O
PMP
N
t-Bu

**4d**

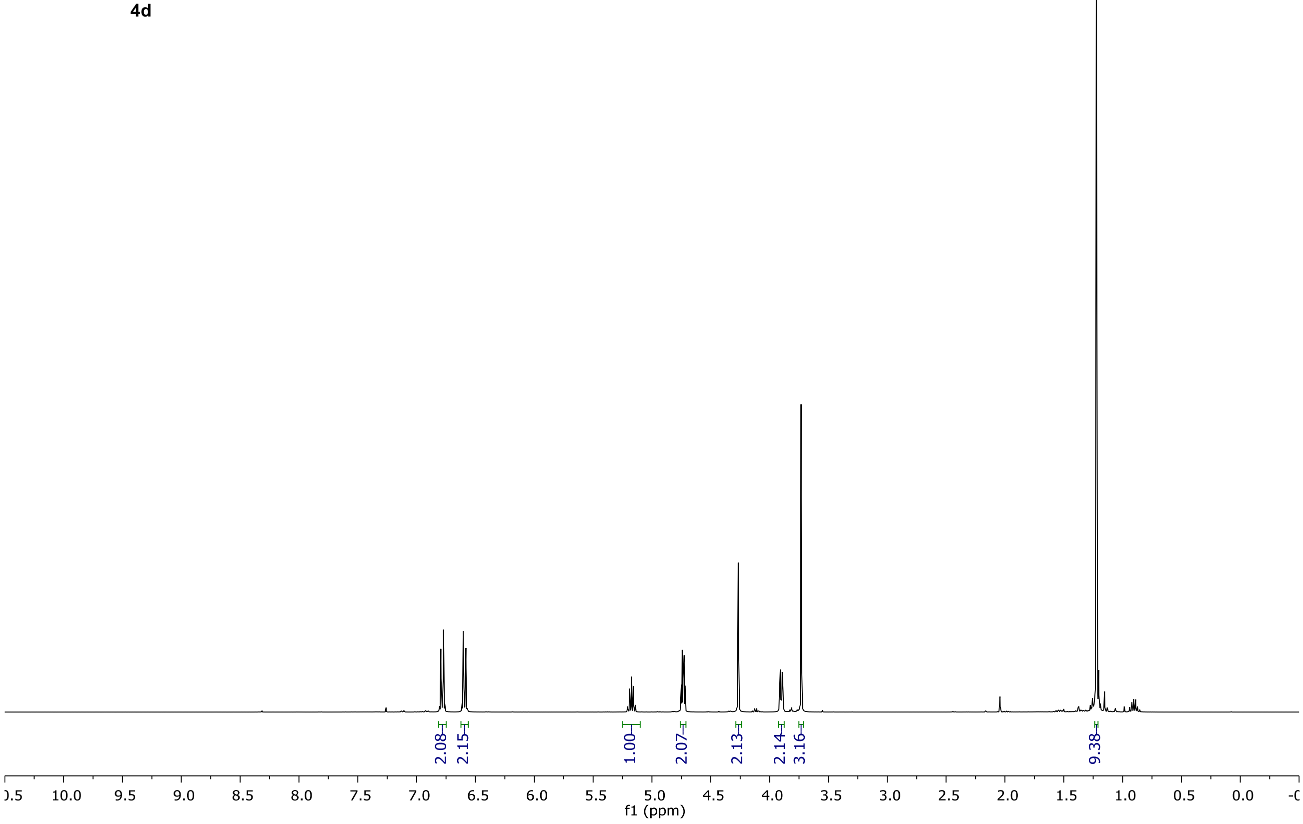
2.08
2.15
1.00
2.07
2.13
2.14
3.16
9.38
f1 (ppm)

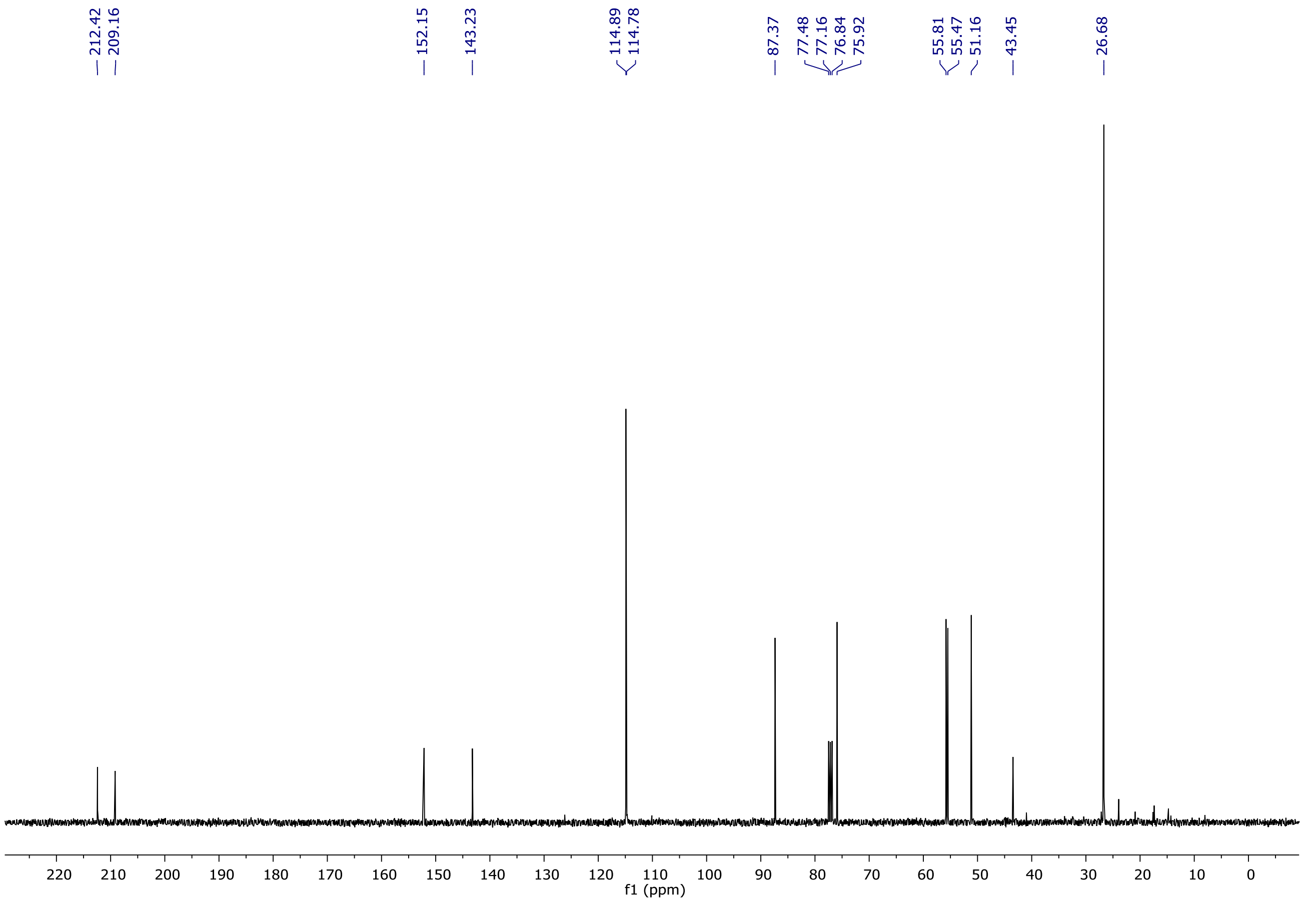
212.42
209.16
152.15
143.23
114.89
114.78
87.37
77.48
77.16
76.84
75.92
55.81
55.47
51.16
43.45
26.68
f1 (ppm)

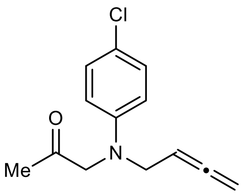
Cl
O
Me
N

**4e**

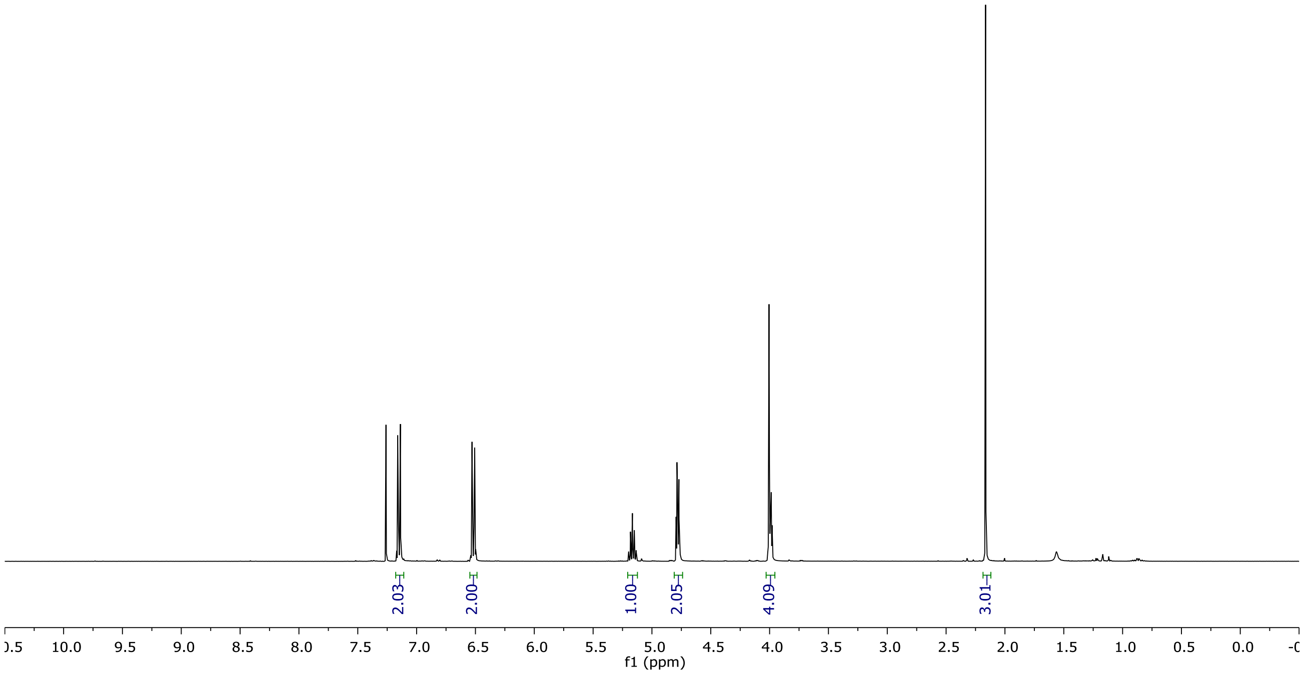
2.03
2.00
1.00
2.05
4.09
3.01
f1 (ppm)

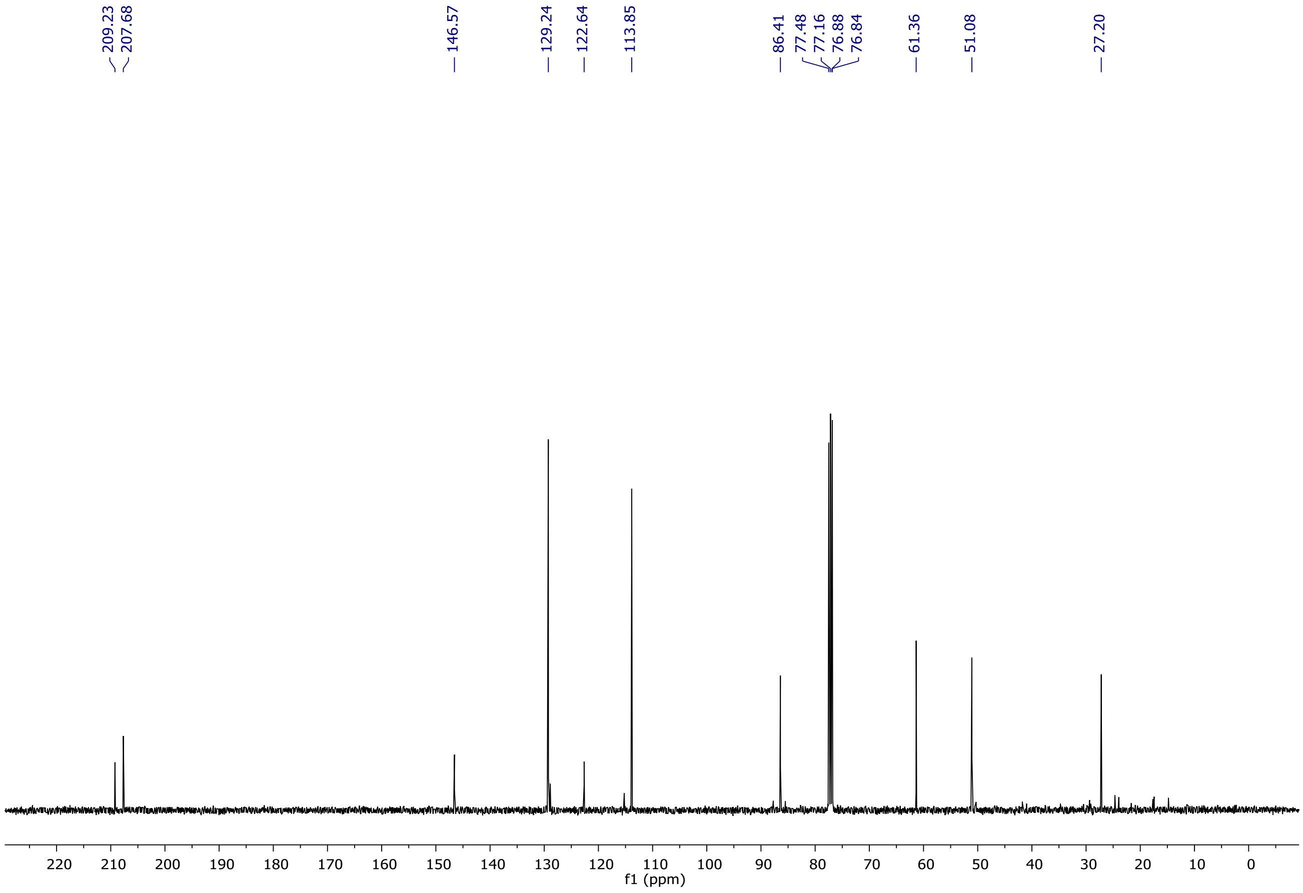
209.23
207.68
146.57
129.24
122.64
113.85
86.41
77.48
77.16
76.88
76.84
61.36
51.08
27.20
f1 (ppm)

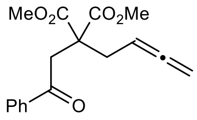
MeO2C
CO2Me
Ph
O

**4f**

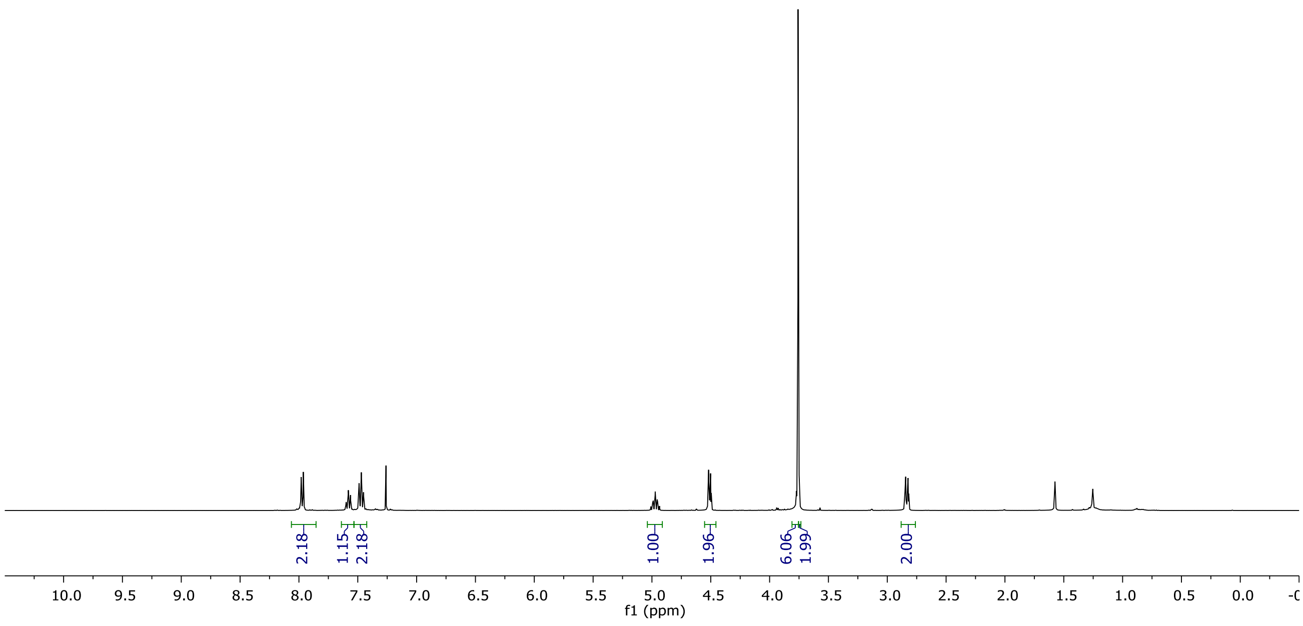
2.18
1.15
2.18
1.00
1.96
6.06
1.99
2.00
10.0 9.5 9.0 8.5 8.0 7.5 7.0 6.5 6.0 5.5 5.0 4.5 4.0 3.5 3.0 2.5 2.0 1.5 1.0 0.5 0.0
f1 (ppm)

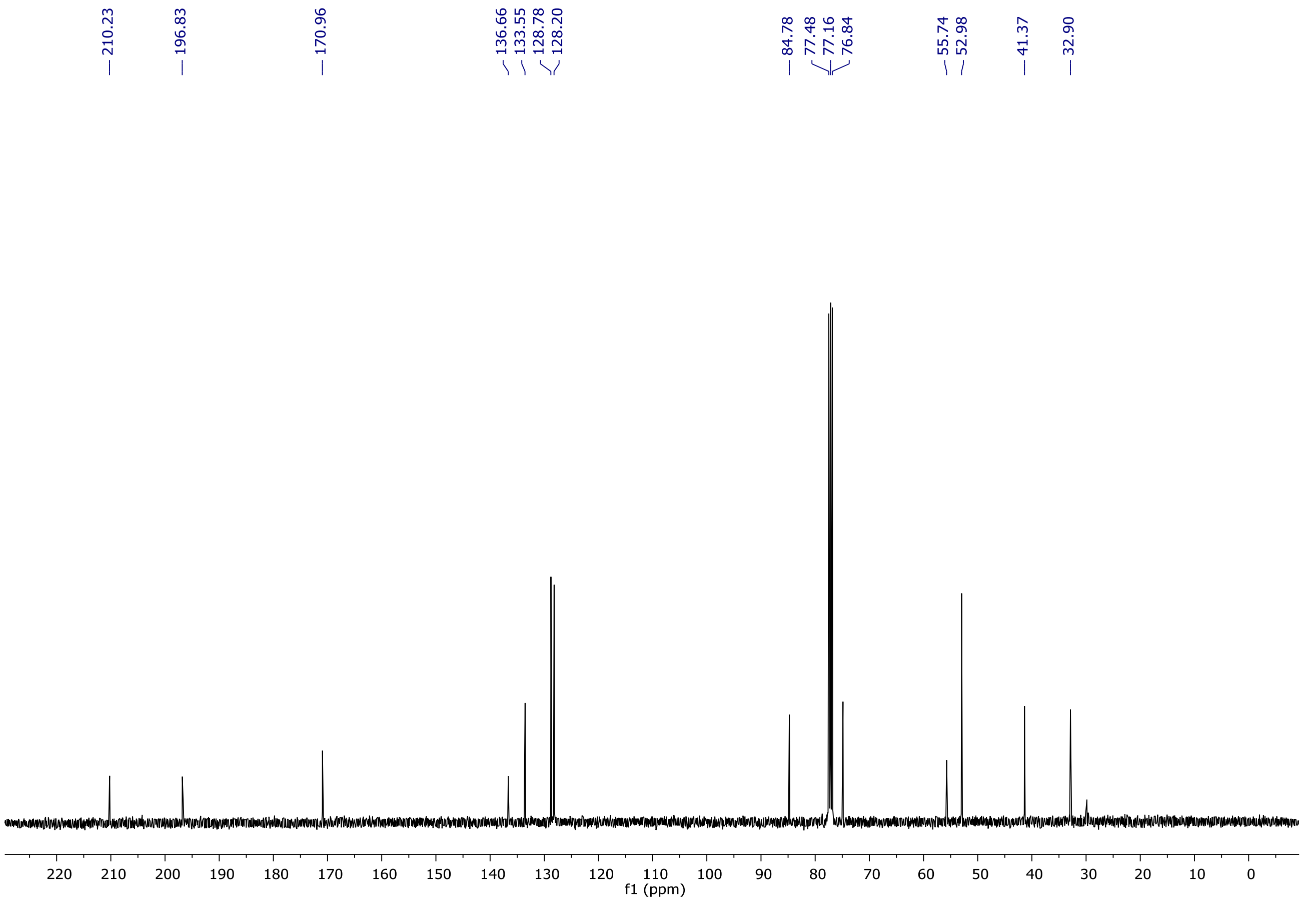
210.23
196.83
170.96
136.66
133.55
128.78
128.20
84.78
77.48
77.16
76.84
55.74
52.98
41.37
32.90
220 210 200 190 180 170 160 150 140 130 120 110 100 90 80 70 60 50 40 30 20 10 0
f1 (ppm)

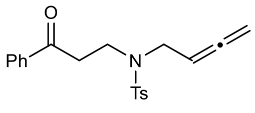
O
Ph
N
Ts

**4g**

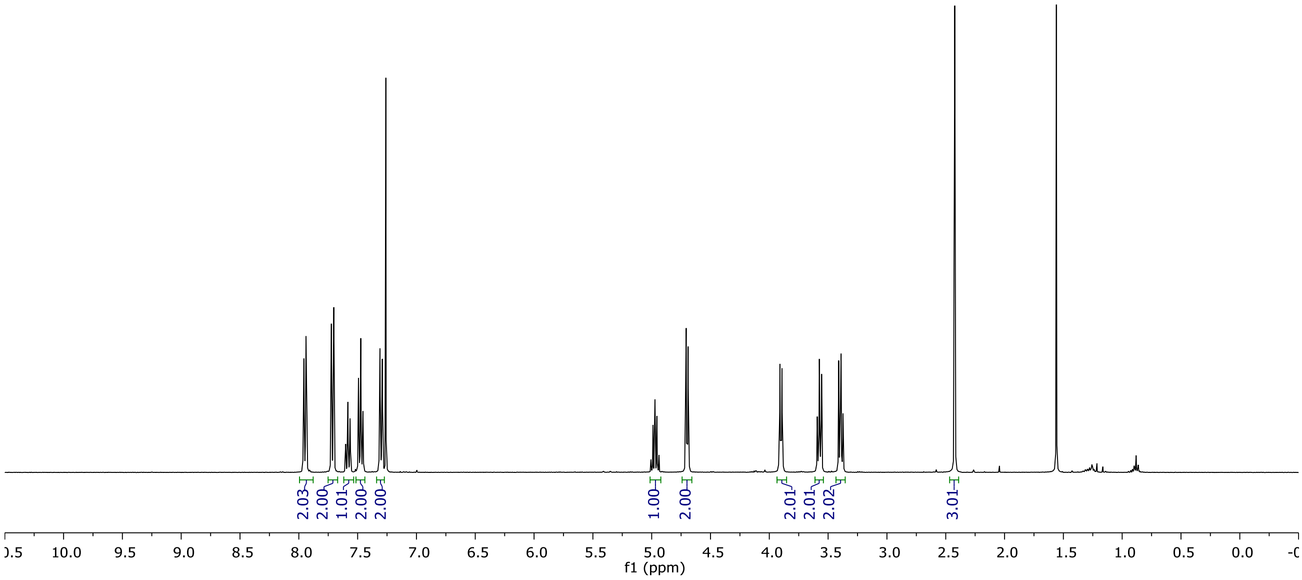
2.03
2.00
1.01
2.00
2.00
1.00
2.00
2.01
2.01
2.02
3.01
f1 (ppm)

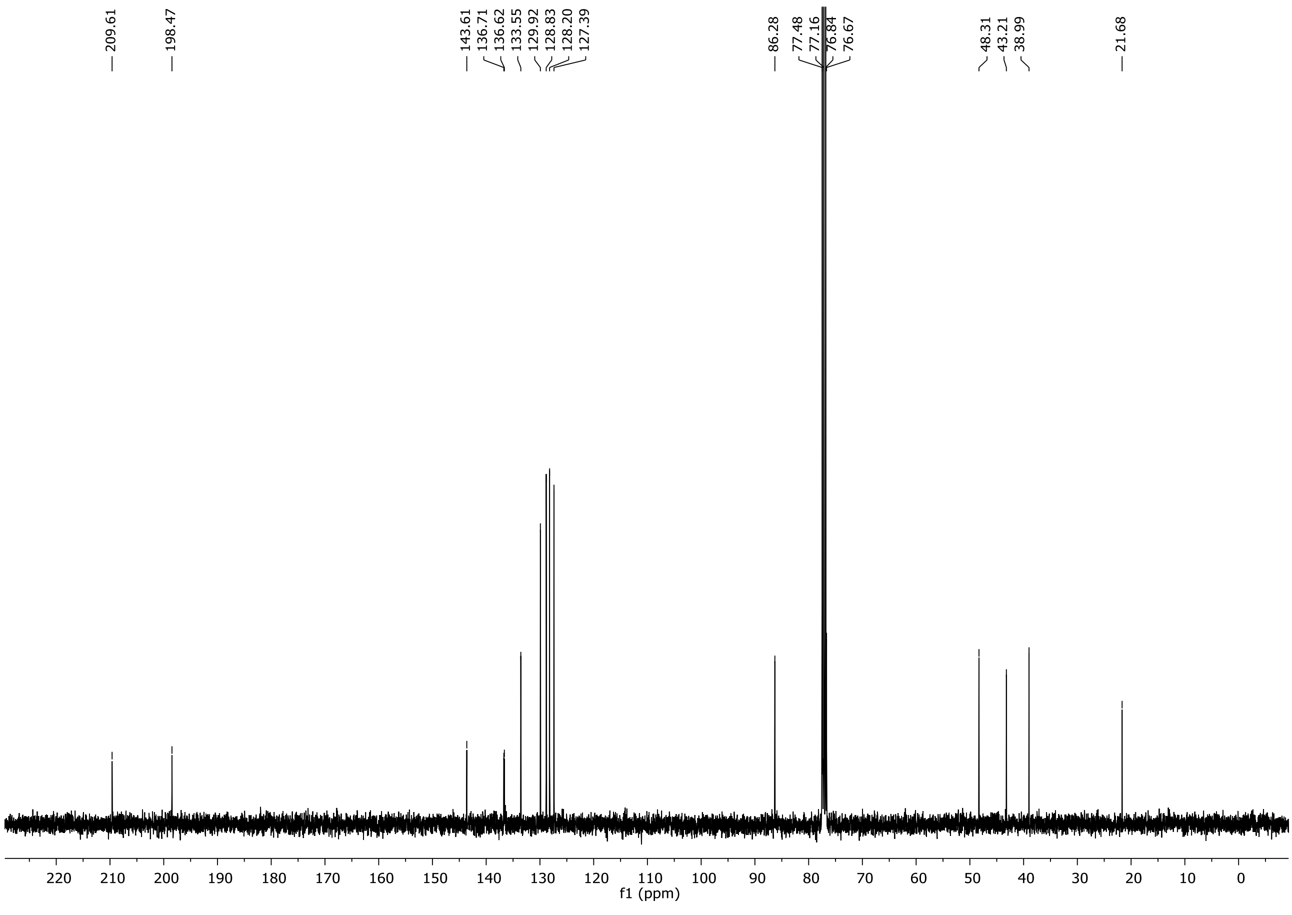
209.61
198.47
143.61
136.71
136.62
133.55
129.92
128.83
128.20
127.39
86.28
77.48
77.16
76.84
76.67
48.31
43.21
38.99
21.68
f1 (ppm)

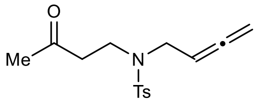
O
Me
N
Ts

**4h**

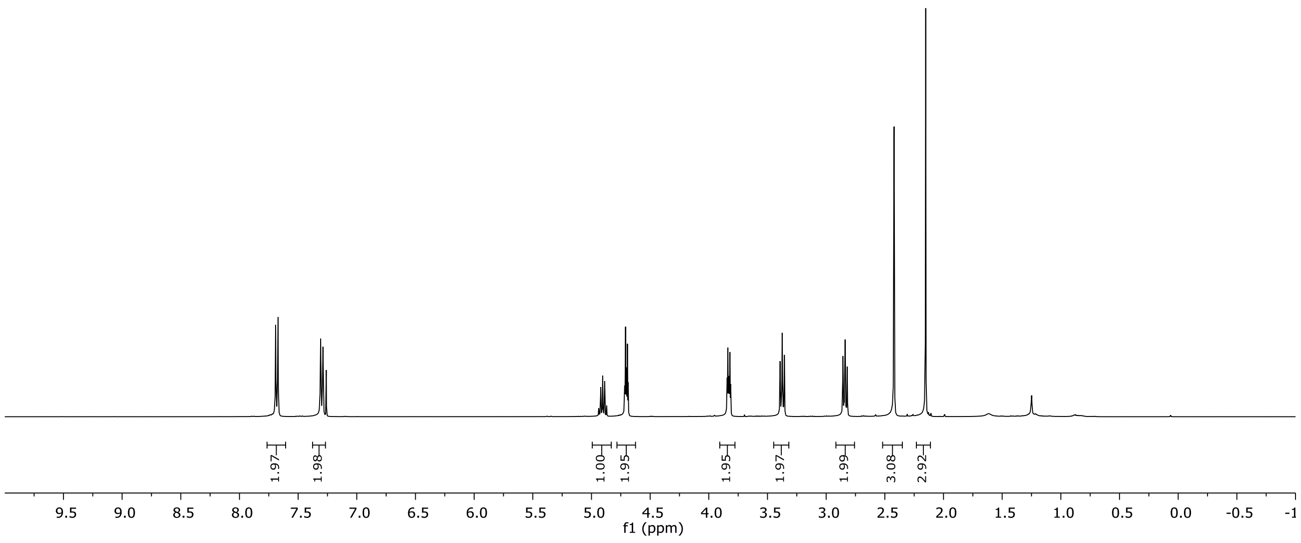
1.97
1.98
1.00
1.95
1.95
1.97
1.99
3.08
2.92
9.5
9.0
8.5
8.0
7.5
7.0
6.5
6.0
5.5
5.0
4.5
4.0
3.5
3.0
2.5
2.0
1.5
1.0
0.5
0.0
-0.5
f1 (ppm)

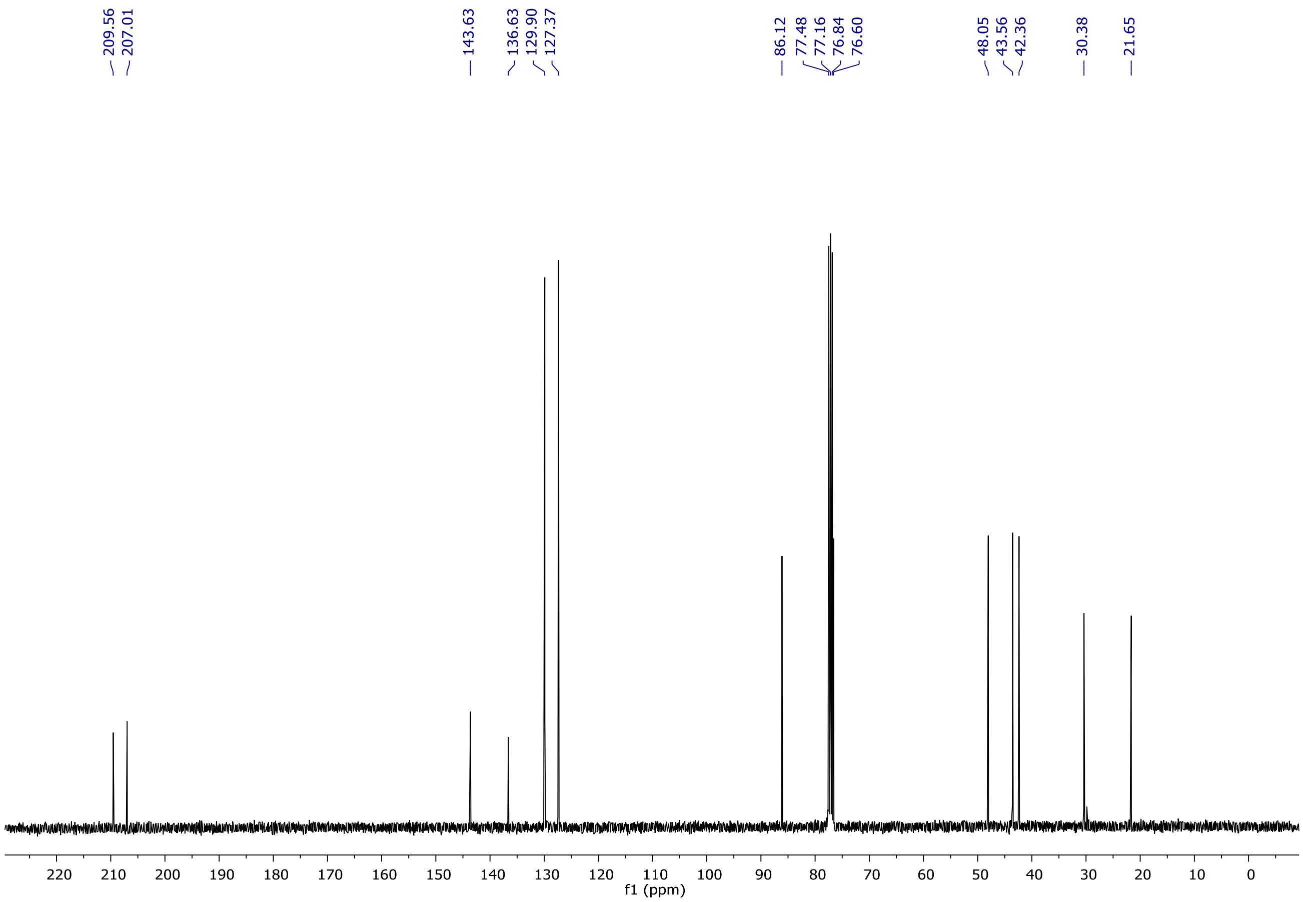
209.56
207.01
143.63
136.63
129.90
127.37
86.12
77.48
77.16
76.84
76.60
48.05
43.56
42.36
30.38
21.65
220
210
200
190
180
170
160
150
140
130
120
110
100
90
80
70
60
50
40
30
20
10
0
f1 (ppm)

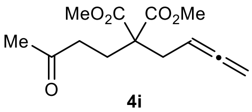

**4i**

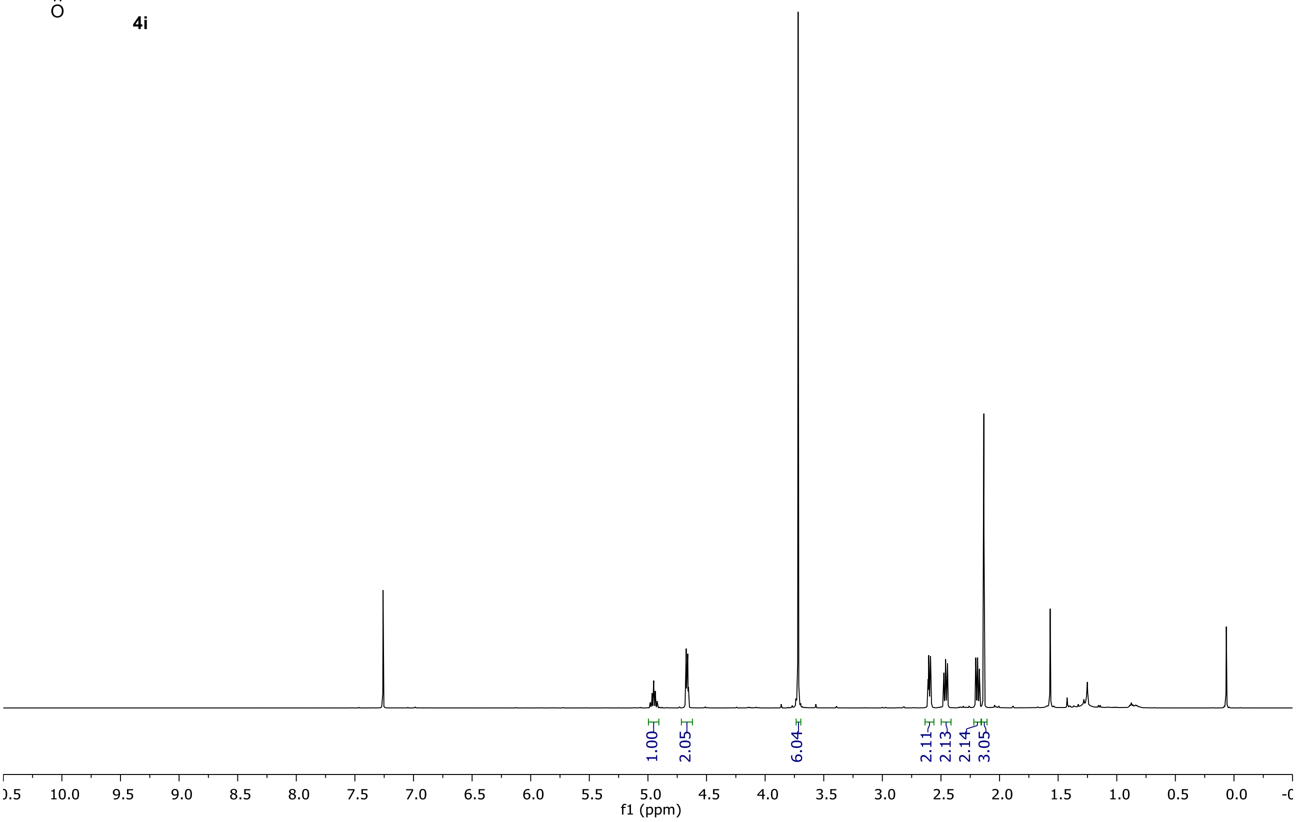
1.00
2.05
6.04
2.11
2.13
2.14
3.05
f1 (ppm)

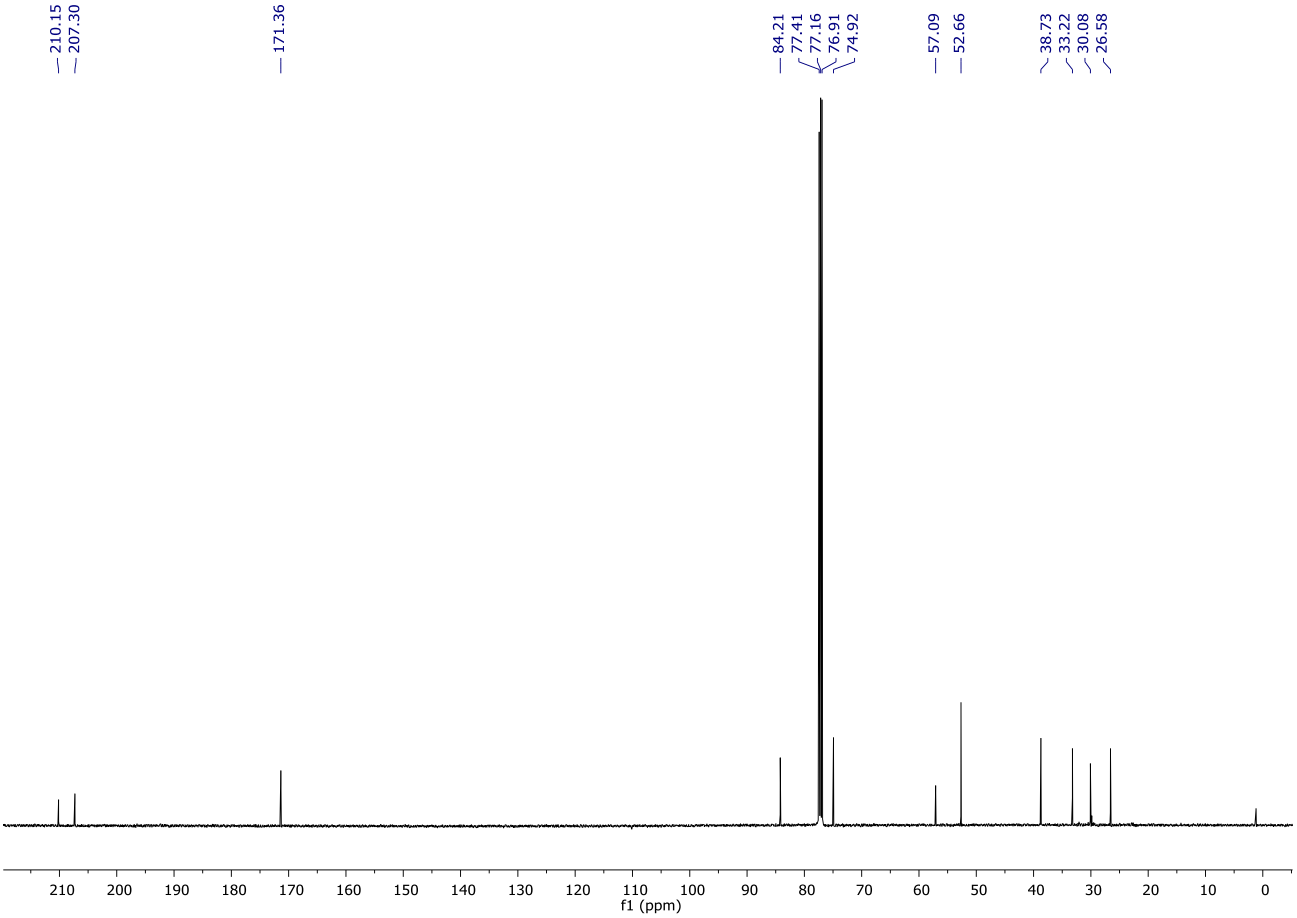
210.15
207.30
171.36
84.21
77.41
77.16
76.91
74.92
57.09
52.66
38.73
33.22
30.08
26.58
f1 (ppm)

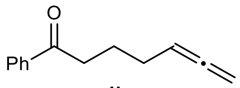
O
Ph

**4j**

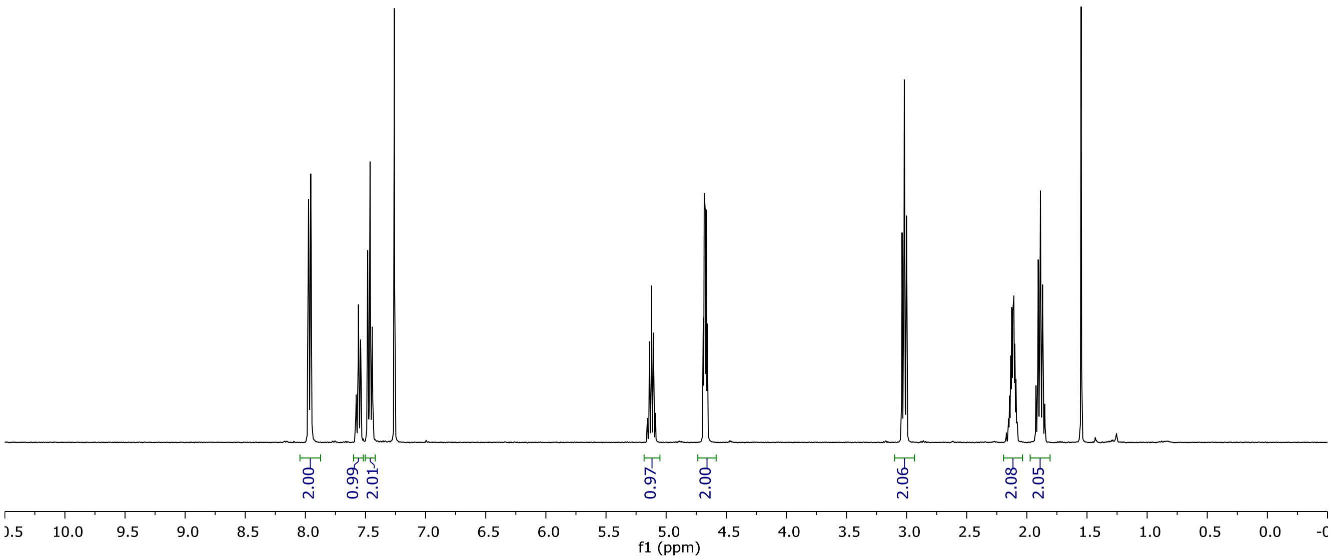
2.00
0.99
2.01
0.97
2.00
2.06
2.08
2.05
f1 (ppm)

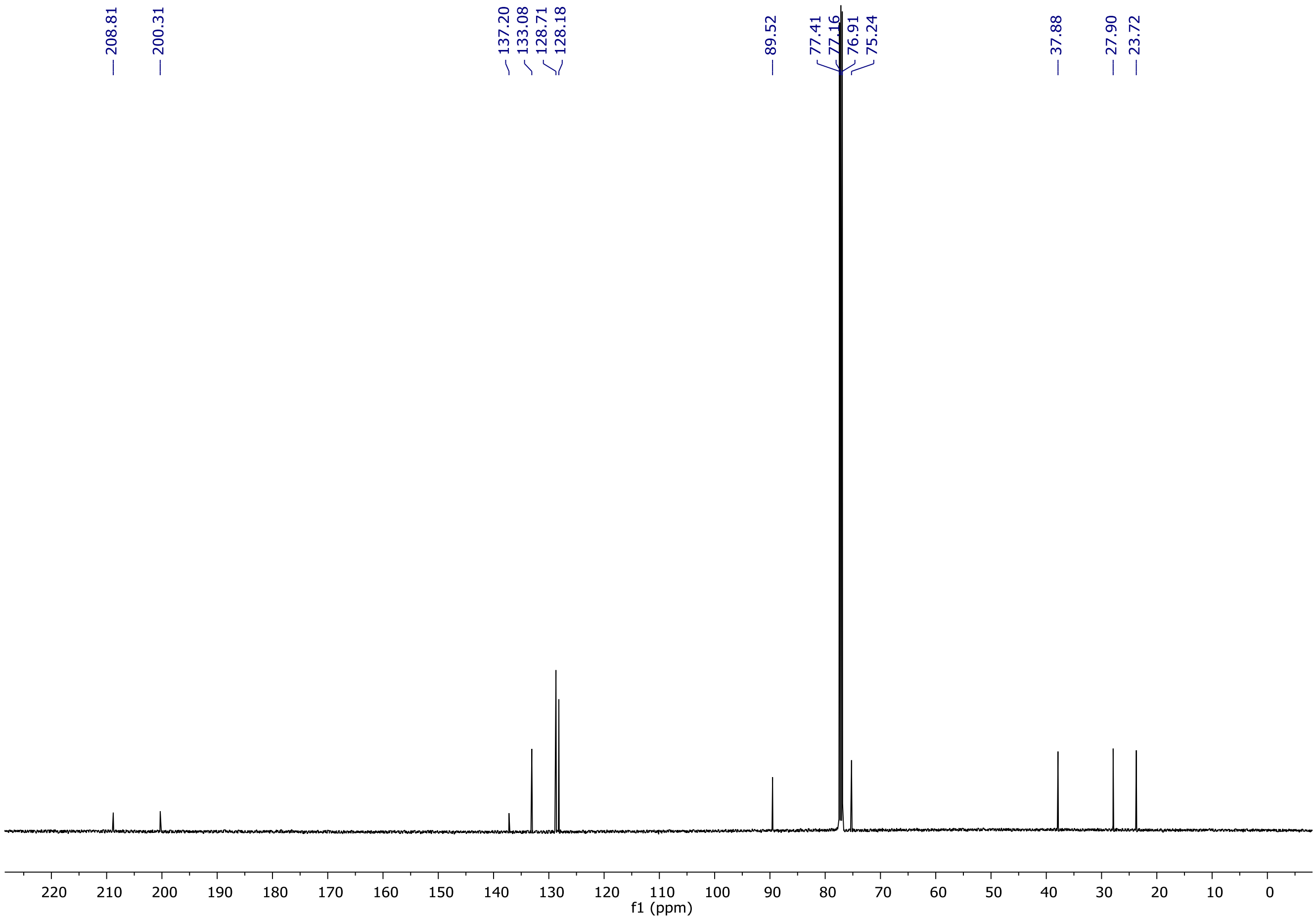
208.81
200.31
137.20
133.08
128.71
128.18
89.52
77.41
77.16
76.91
75.24
37.88
27.90
23.72
f1 (ppm)

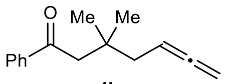
O
Me
Me
Ph

**4k**

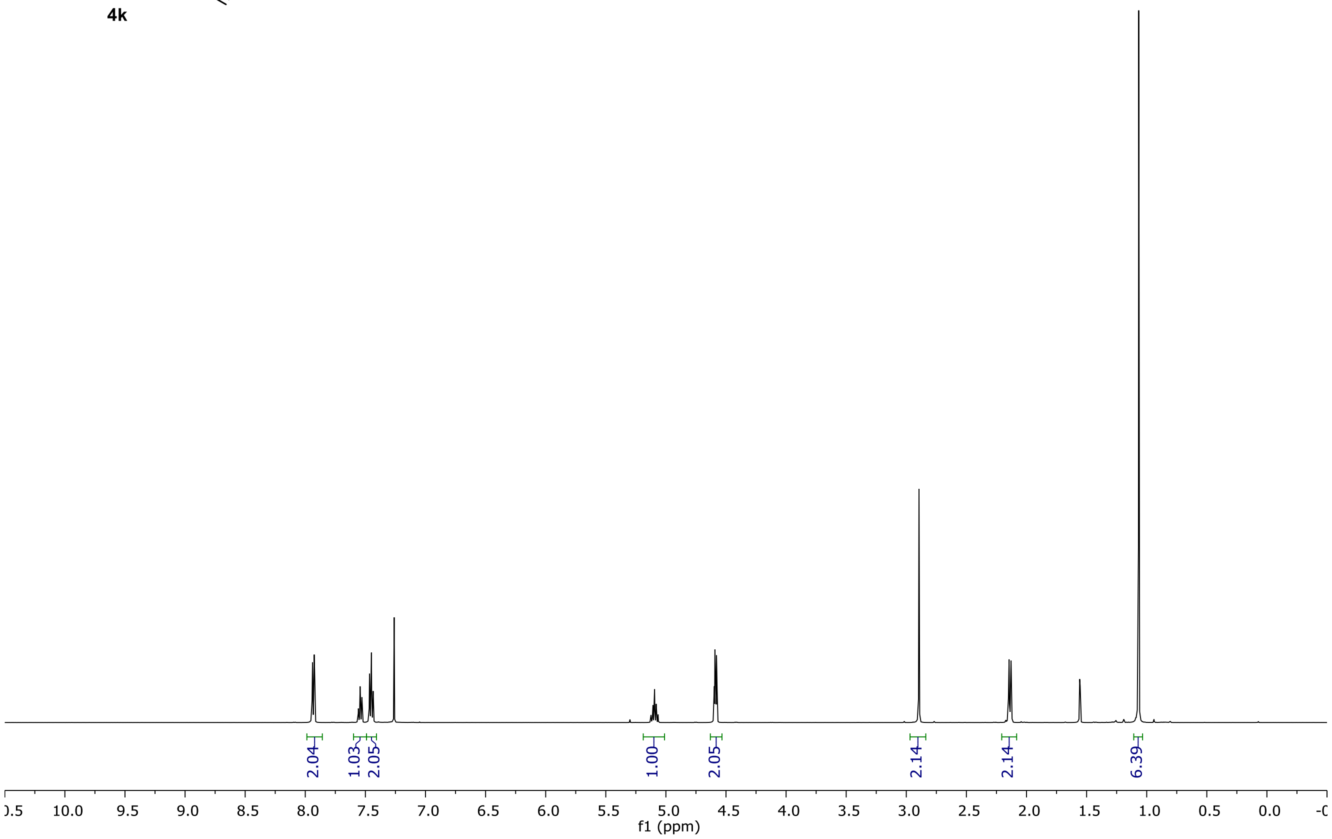
2.04
1.03
2.05
1.00
2.05
2.14
2.14
6.39
f1 (ppm)

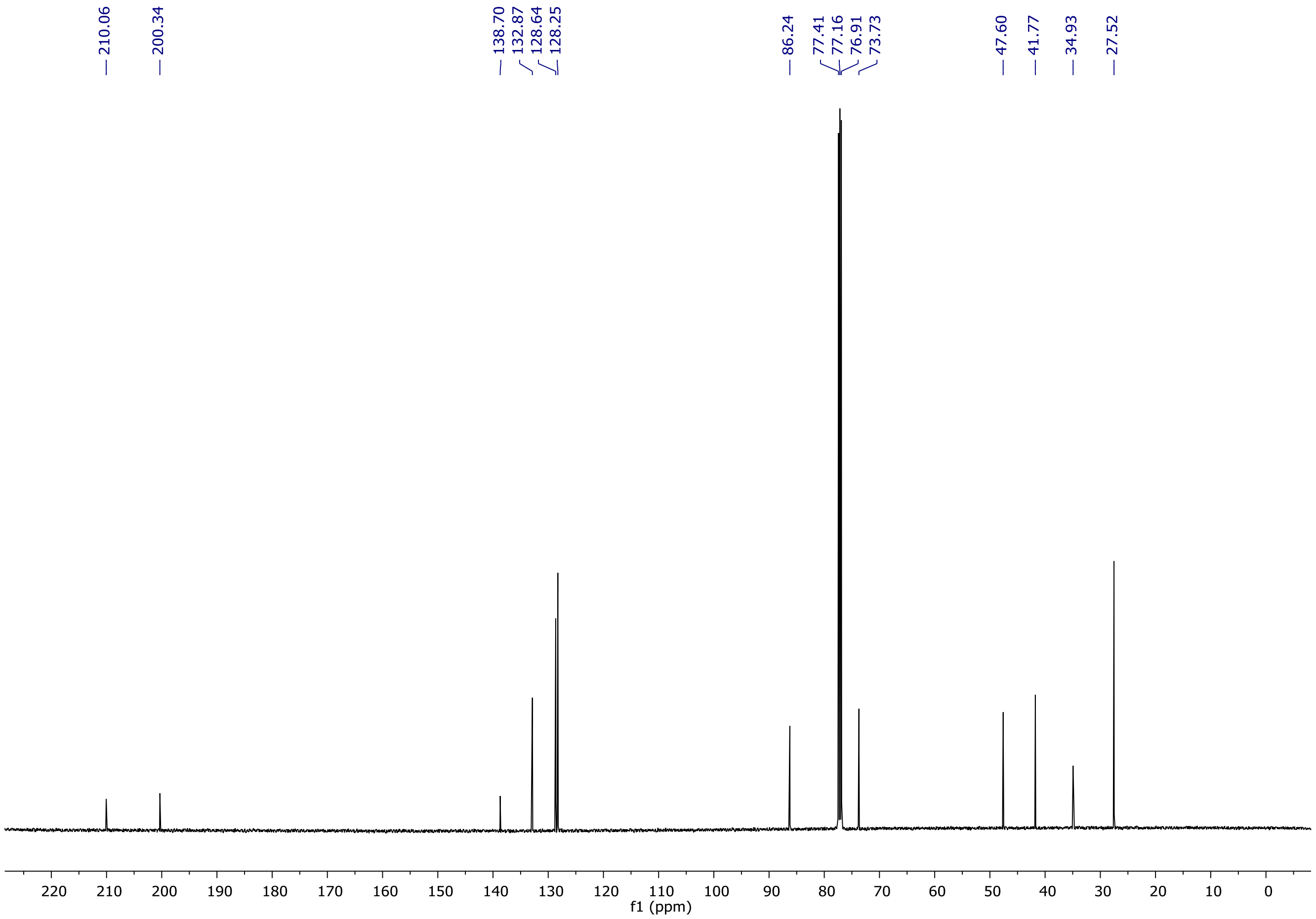
210.06
200.34
138.70
132.87
128.64
128.25
86.24
77.41
77.16
76.91
73.73
47.60
41.77
34.93
27.52
f1 (ppm)

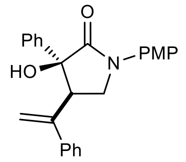
O
Ph
N
PMP
HO
Ph

**2a**

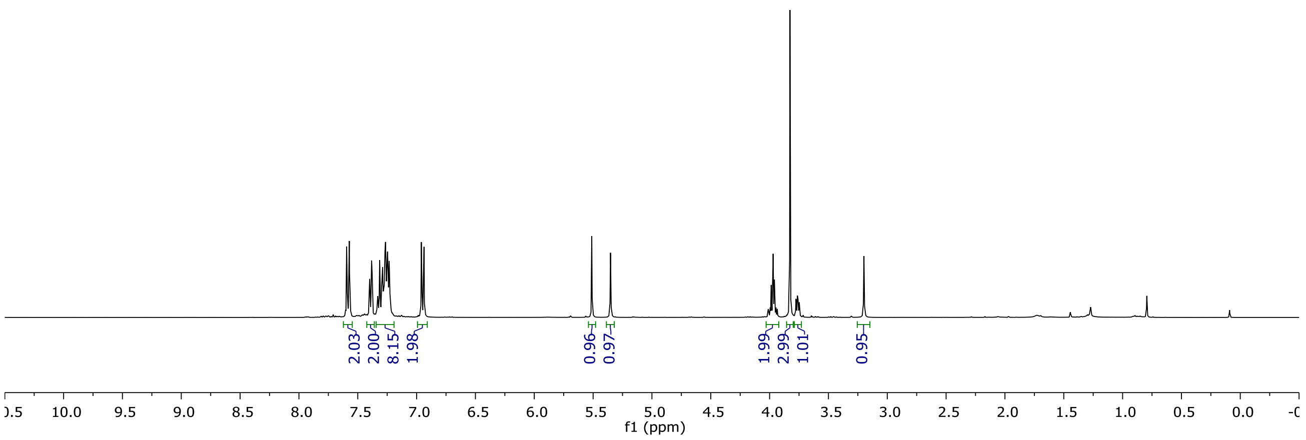
2.03
2.00
8.15
1.98
0.96
0.97
1.99
2.99
1.01
0.95
f1 (ppm)

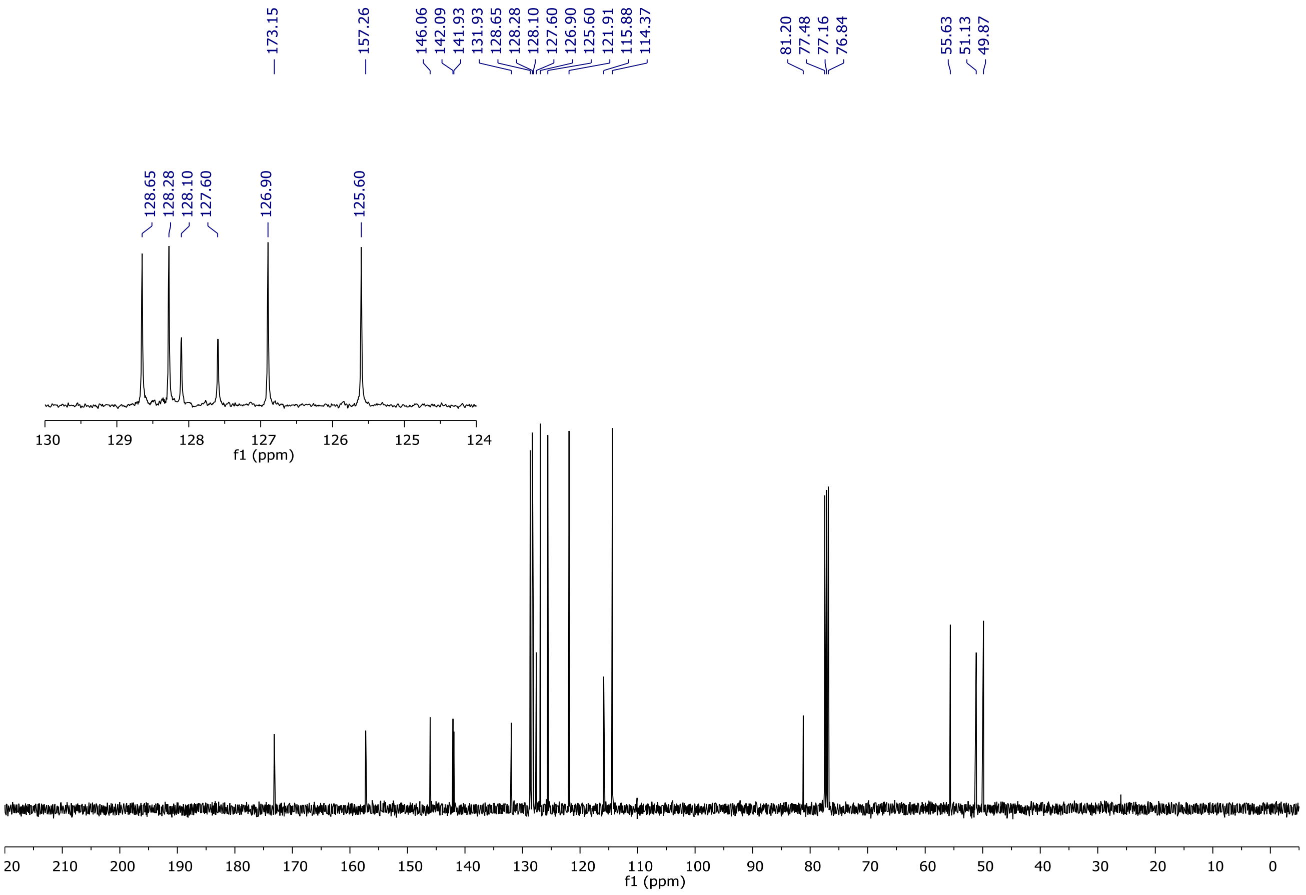
173.15
157.26
146.06
142.09
141.93
131.93
128.65
128.28
128.10
127.60
126.90
125.60
121.91
115.88
114.37
81.20
77.48
77.16
76.84
55.63
51.13
49.87
f1 (ppm)

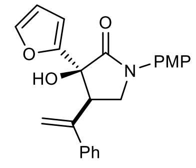
O
O
HO
N
PMP
Ph

**2b**

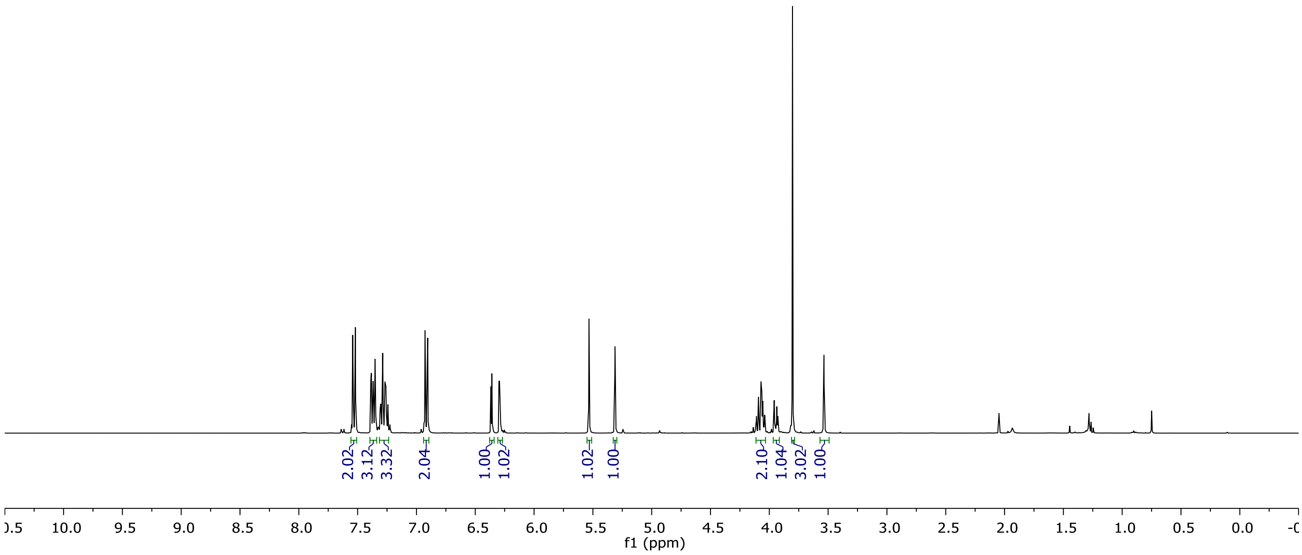
2.02
3.12
3.32
2.04
1.00
1.02
1.02
1.00
2.10
1.04
3.02
1.00
f1 (ppm)

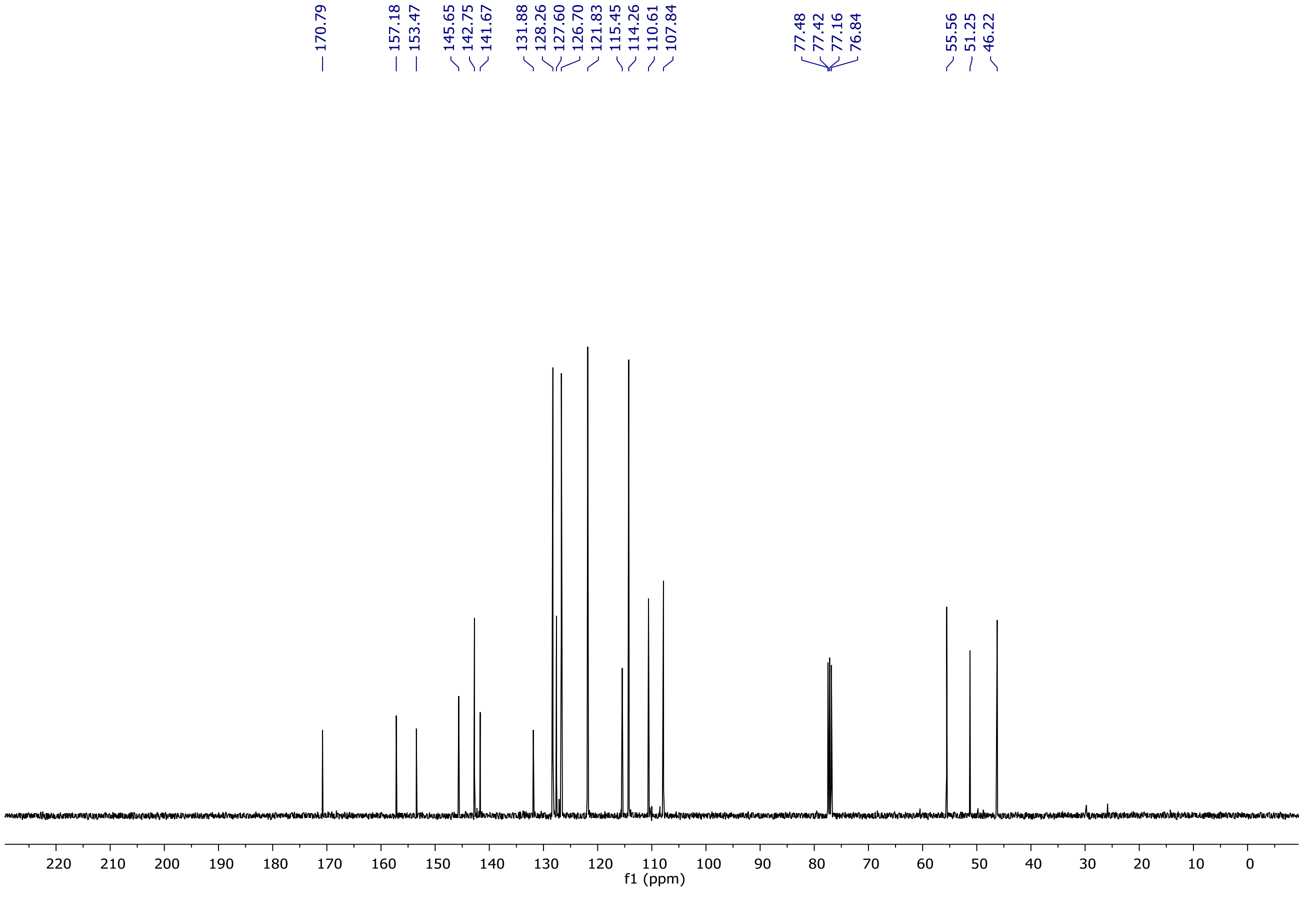
170.79
157.18
153.47
145.65
142.75
141.67
131.88
128.26
127.60
126.70
121.83
115.45
114.26
110.61
107.84
77.48
77.42
77.16
76.84
55.56
51.25
46.22
f1 (ppm)

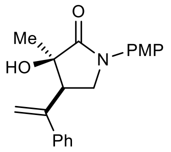

Me
HO
O
N
PMP
Ph


**2c**

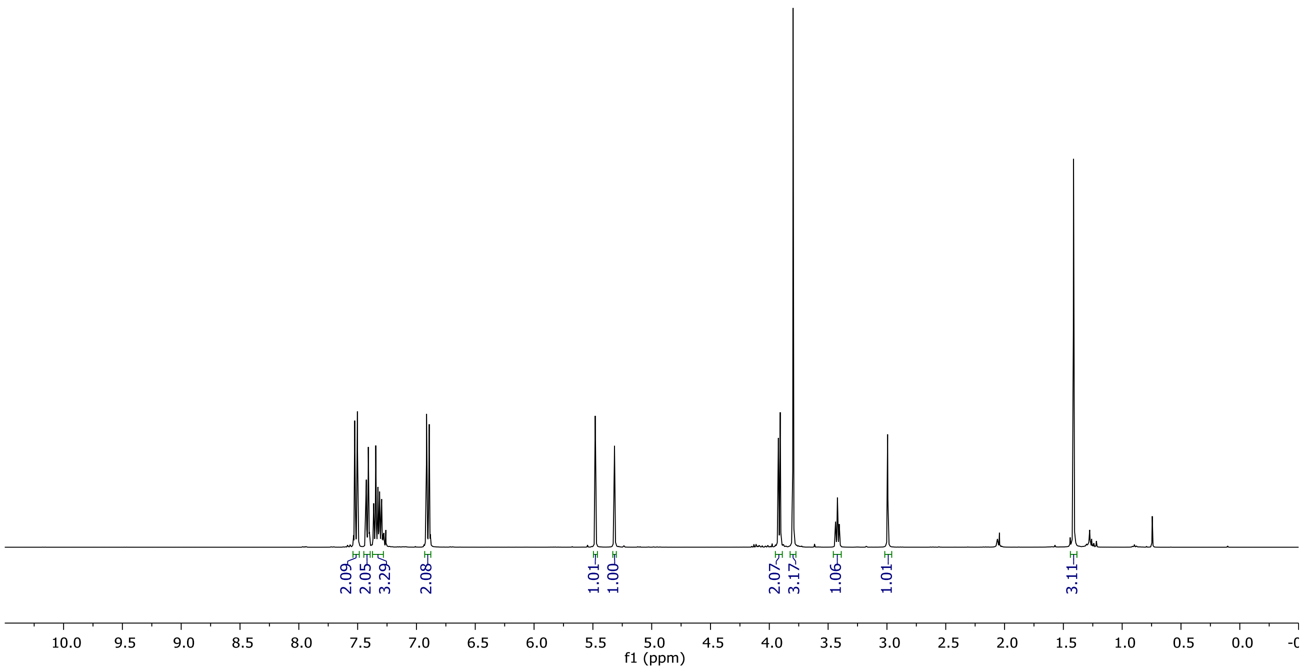

2.09
2.05
3.29
2.08
1.01
1.00
2.07
3.17
1.06
1.01
3.11
10.0 9.5 9.0 8.5 8.0 7.5 7.0 6.5 6.0 5.5 5.0 4.5 4.0 3.5 3.0 2.5 2.0 1.5 1.0 0.5 0.0
f1 (ppm)


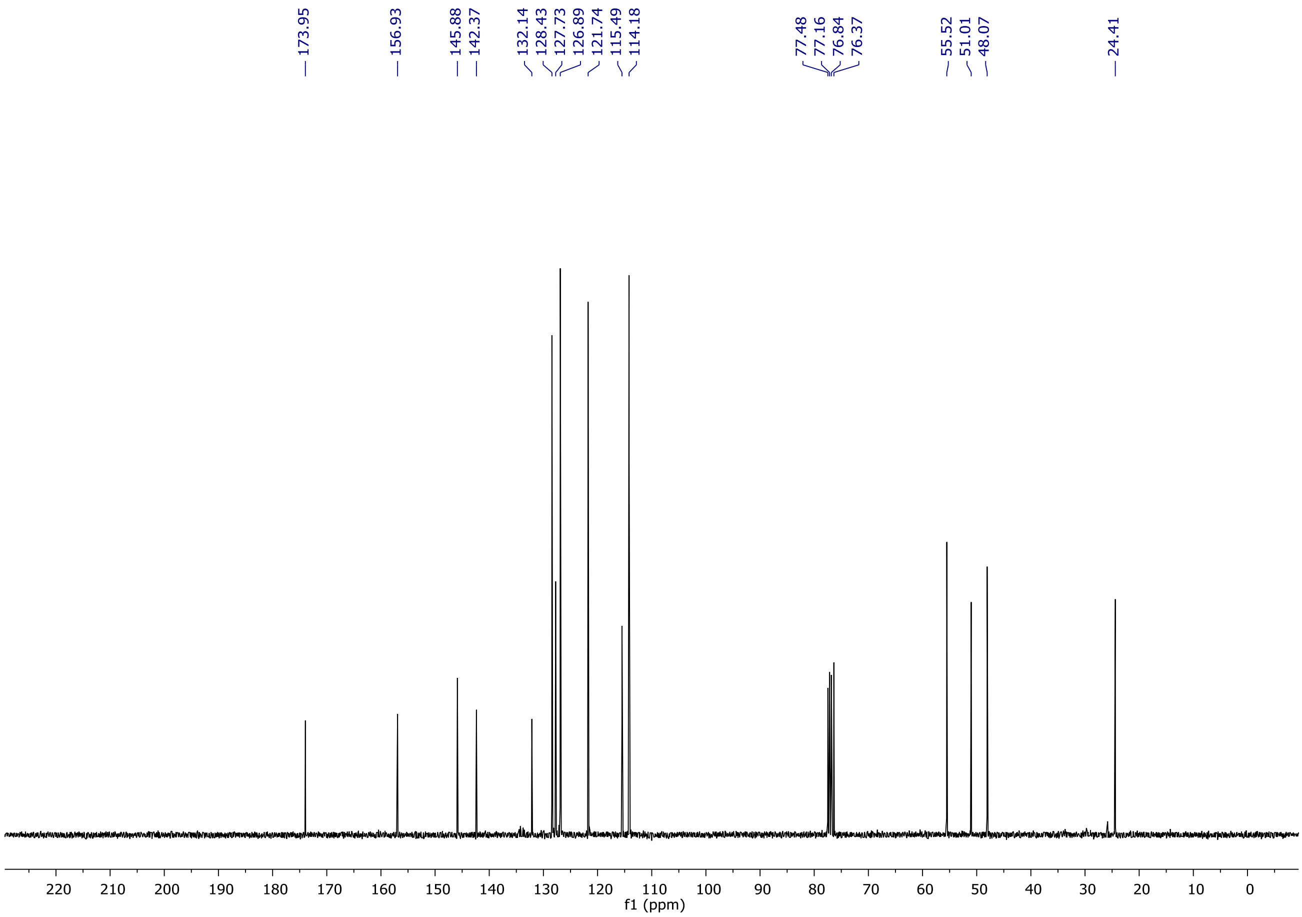

173.95
156.93
145.88
142.37
132.14
128.43
127.73
126.89
121.74
115.49
114.18
77.48
77.16
76.84
76.37
55.52
51.01
48.07
24.41
220 210 200 190 180 170 160 150 140 130 120 110 100 90 80 70 60 50 40 30 20 10 0
f1 (ppm)

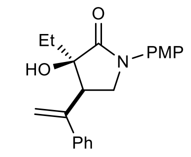
O
Et
HO
N
PMP
Ph

**2d**

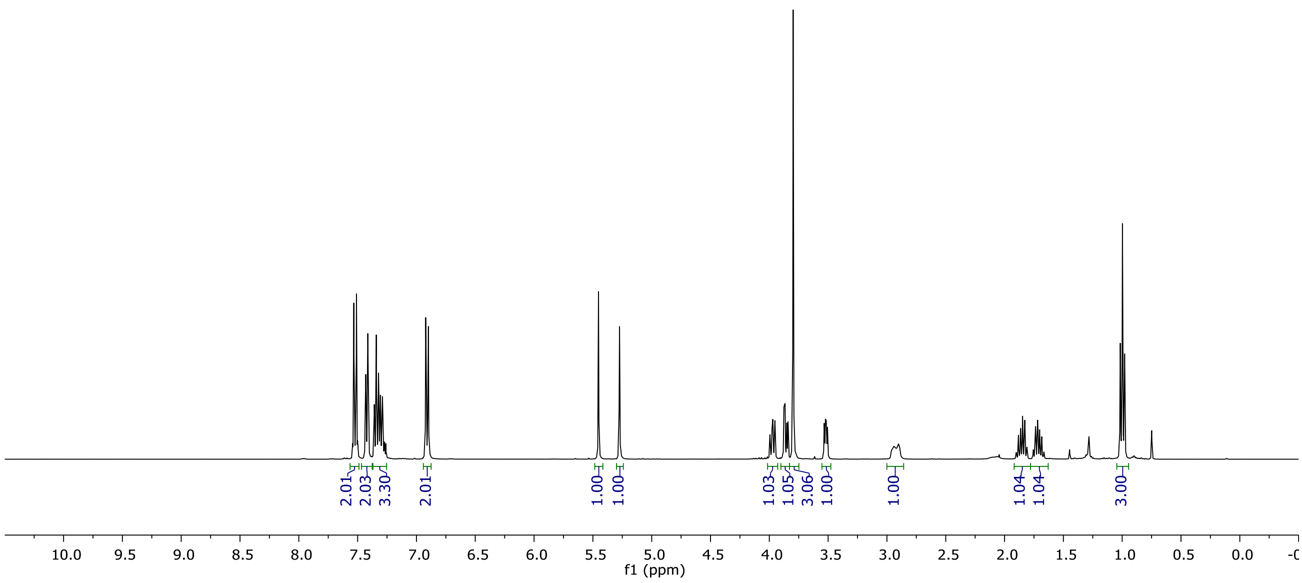
2.01
2.03
3.30
2.01
1.00
1.00
1.03
1.05
3.06
1.00
1.00
1.04
1.04
3.00
10.0 9.5 9.0 8.5 8.0 7.5 7.0 6.5 6.0 5.5 5.0 4.5 4.0 3.5 3.0 2.5 2.0 1.5 1.0 0.5 0.0
f1 (ppm)

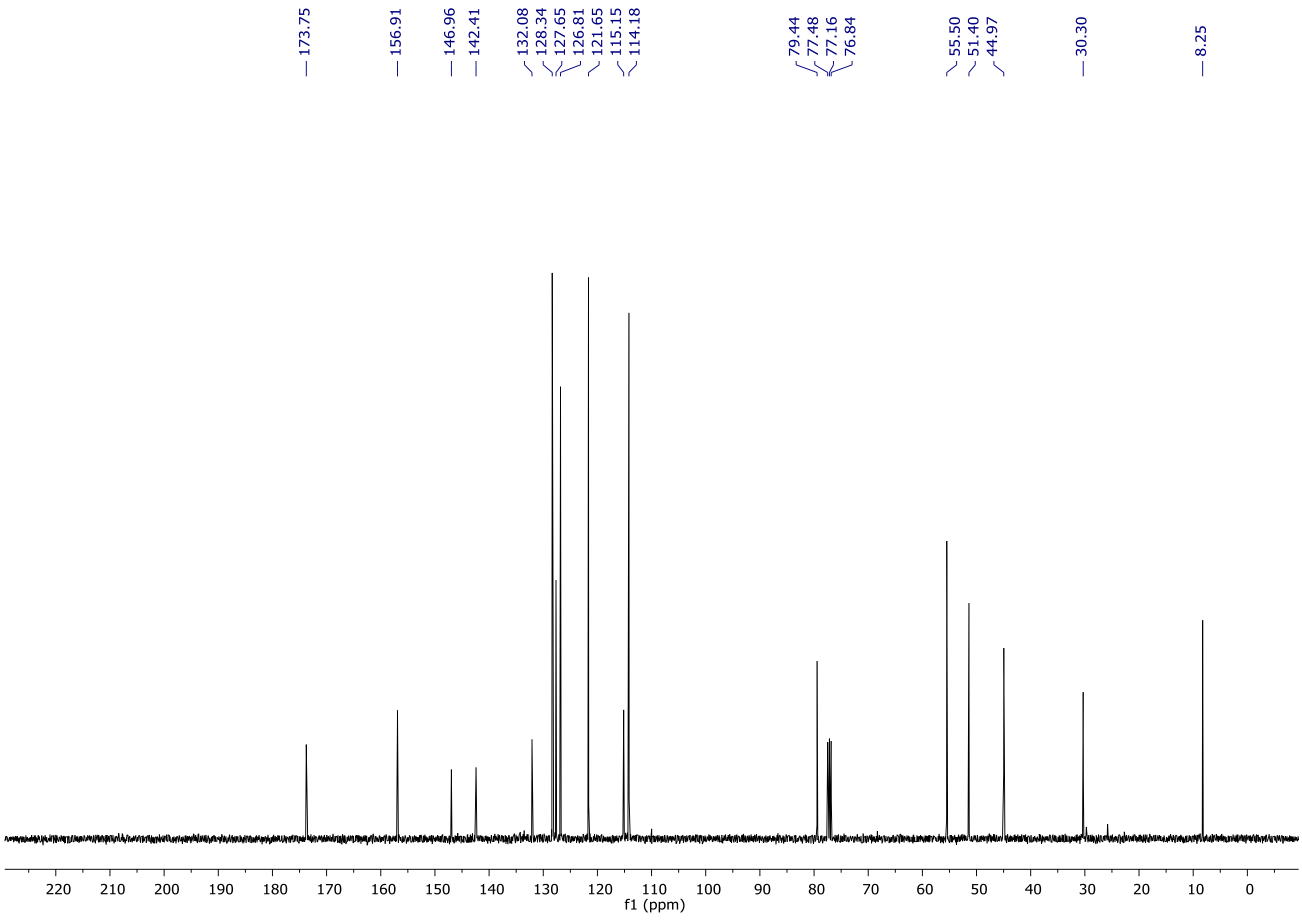
173.75
156.91
146.96
142.41
132.08
128.34
127.65
126.81
121.65
115.15
114.18
79.44
77.48
77.16
76.84
55.50
51.40
44.97
30.30
8.25
220 210 200 190 180 170 160 150 140 130 120 110 100 90 80 70 60 50 40 30 20 10 0
f1 (ppm)

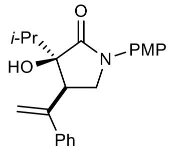
i-Pr
HO
O
N
PMP
Ph

**2e**

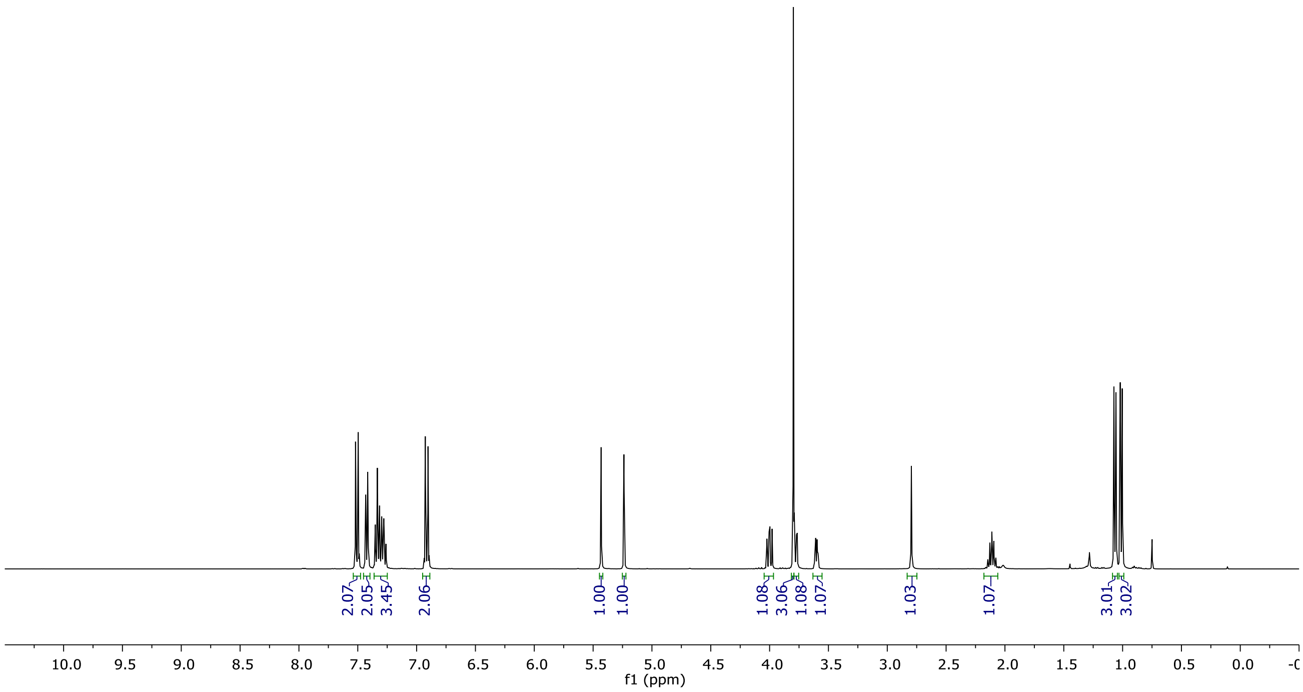
2.07
2.05
3.45
2.06
1.00
1.00
1.08
3.06
1.08
1.07
1.03
1.07
3.01
3.02
f1 (ppm)

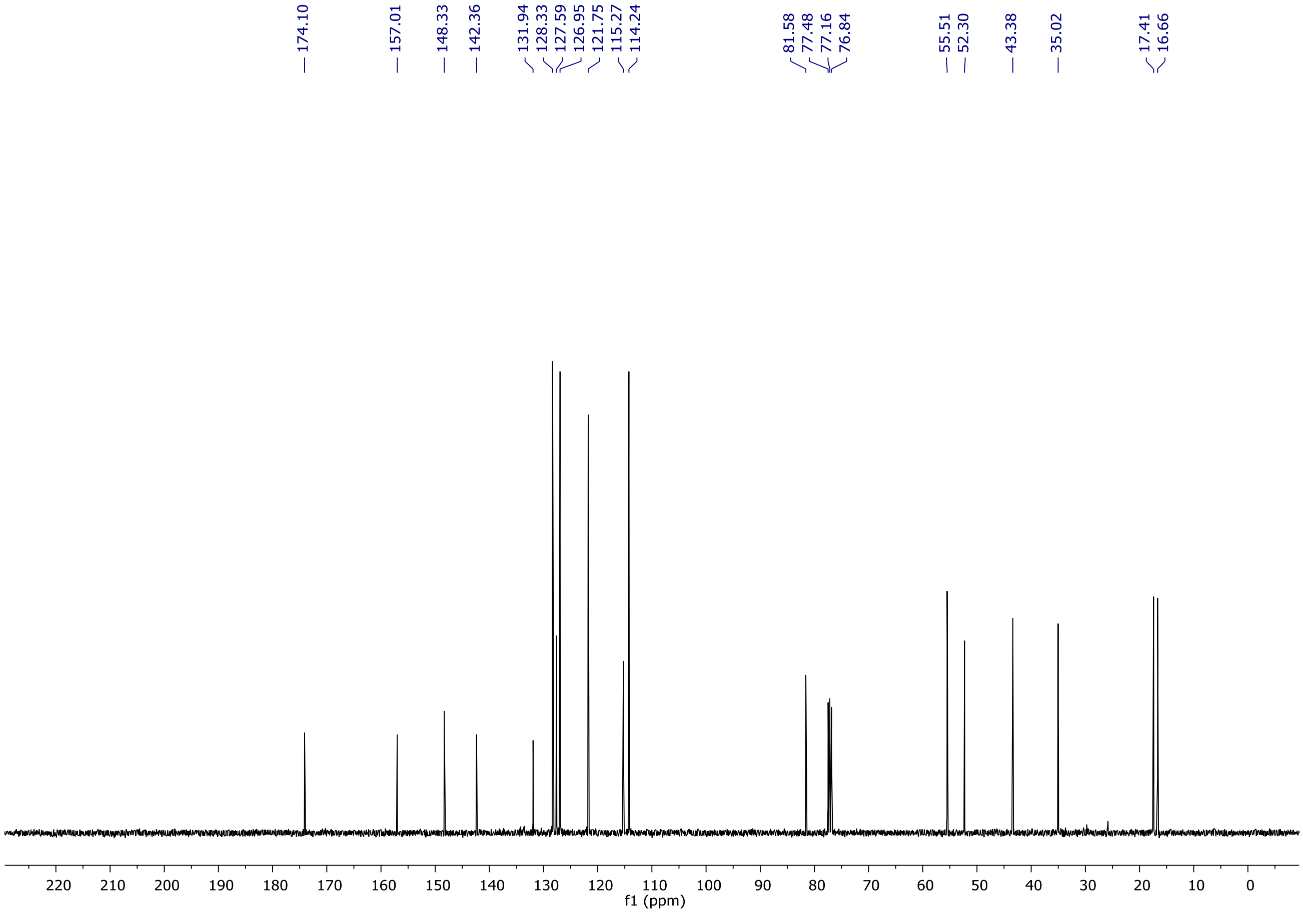
174.10
157.01
148.33
142.36
131.94
128.33
127.59
126.95
121.75
115.27
114.24
81.58
77.48
77.16
76.84
55.51
52.30
43.38
35.02
17.41
16.66
f1 (ppm)

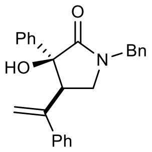
O
Ph
HO
N
Bn
Ph

**2f**

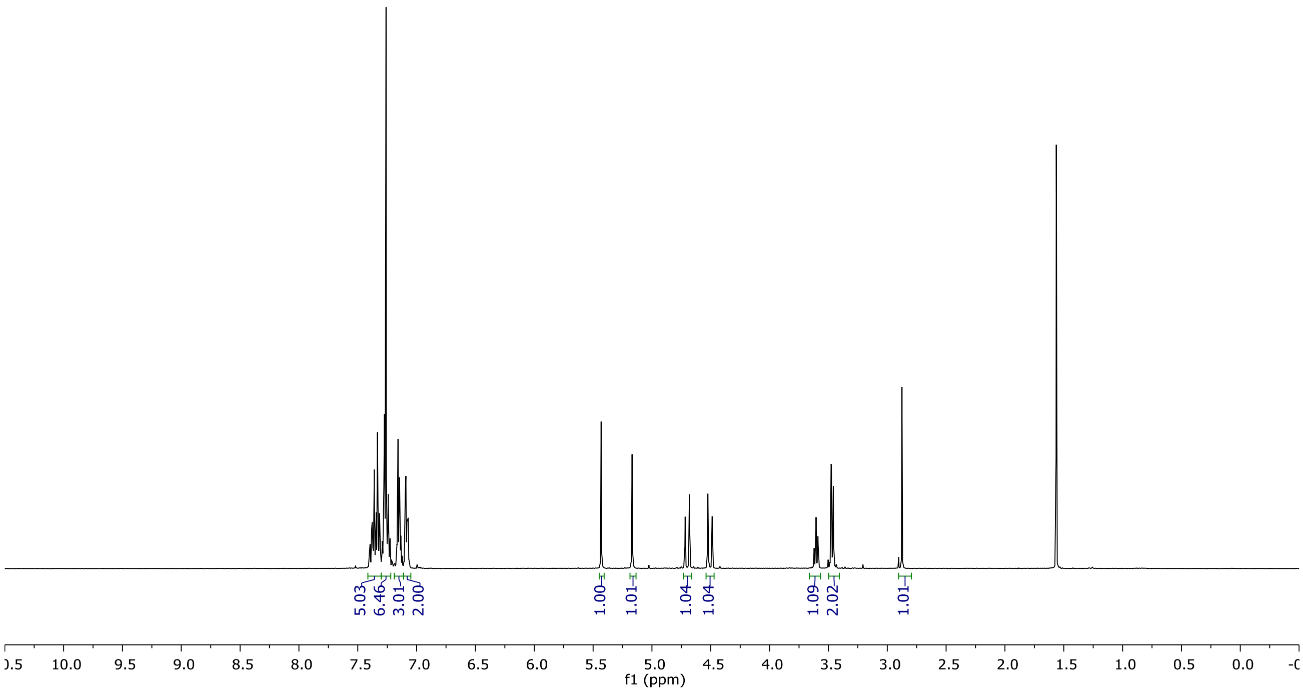
5.03
6.46
3.01
2.00
1.00
1.01
1.04
1.04
1.09
2.02
1.01
10.0 9.5 9.0 8.5 8.0 7.5 7.0 6.5 6.0 5.5 5.0 4.5 4.0 3.5 3.0 2.5 2.0 1.5 1.0 0.5 0.0
f1 (ppm)

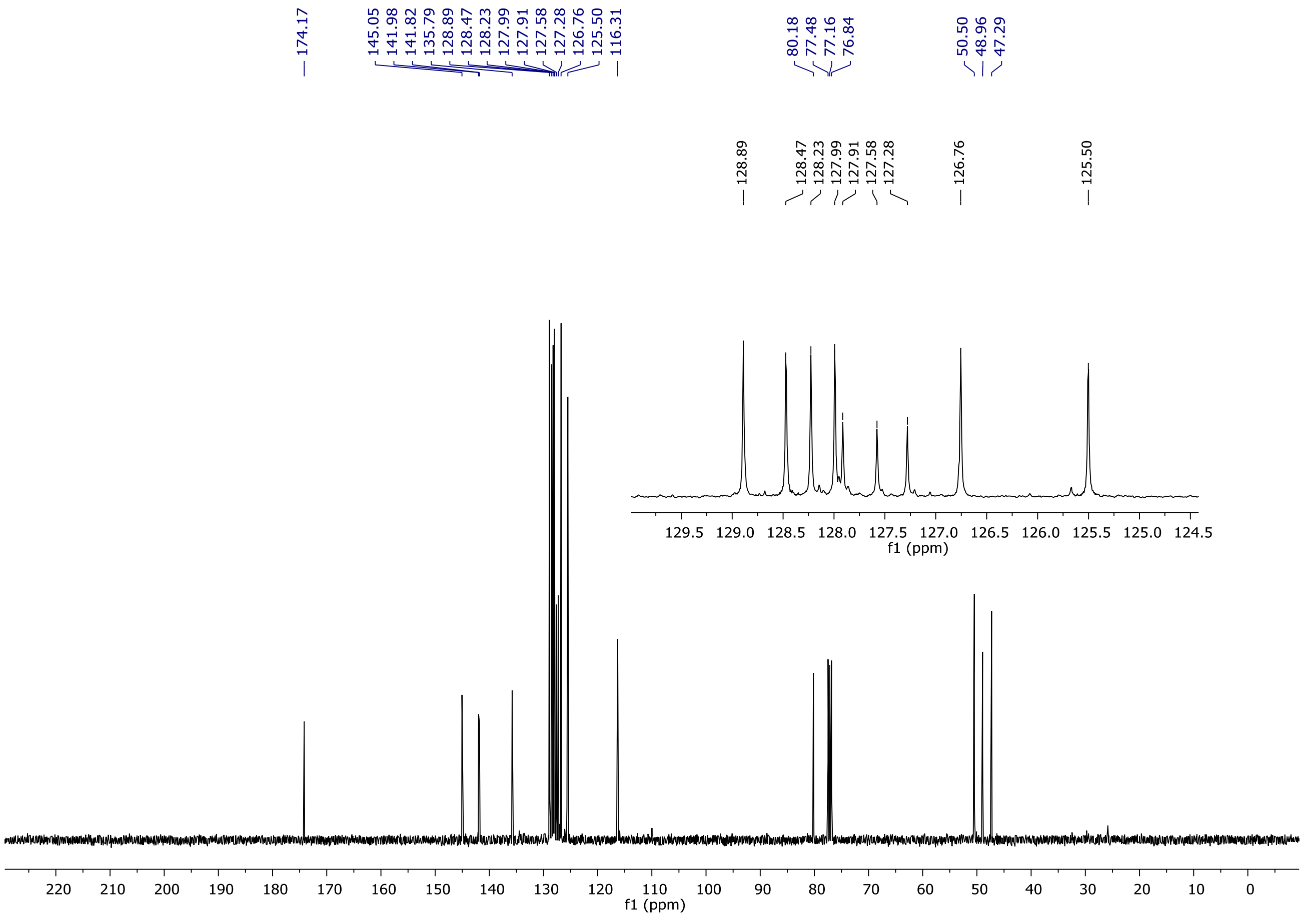
174.17
145.05
141.98
141.82
135.79
128.89
128.47
128.23
127.99
127.91
127.58
127.28
126.76
125.50
116.31
80.18
77.48
77.16
76.84
50.50
48.96
47.29
128.89
128.47
128.23
127.99
127.91
127.58
127.28
126.76
125.50
129.5 129.0 128.5 128.0 127.5 127.0 126.5 126.0 125.5 125.0 124.5
f1 (ppm)
220 210 200 190 180 170 160 150 140 130 120 110 100 90 80 70 60 50 40 30 20 10 0
f1 (ppm)

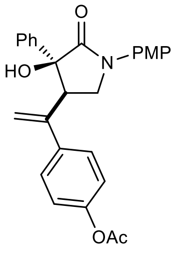
O
Ph
HO
N
PMP
OAc

**2g**

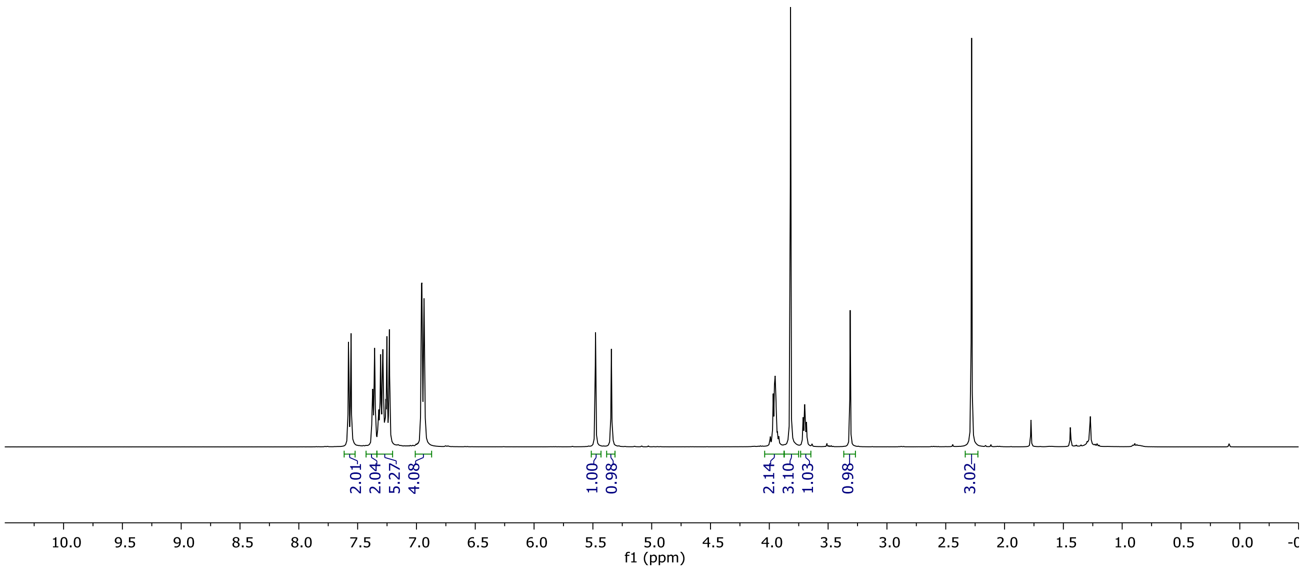
2.01
2.04
5.27
4.08
1.00
0.98
2.14
3.10
1.03
0.98
3.02
10.0 9.5 9.0 8.5 8.0 7.5 7.0 6.5 6.0 5.5 5.0 4.5 4.0 3.5 3.0 2.5 2.0 1.5 1.0 0.5 0.0
f1 (ppm)

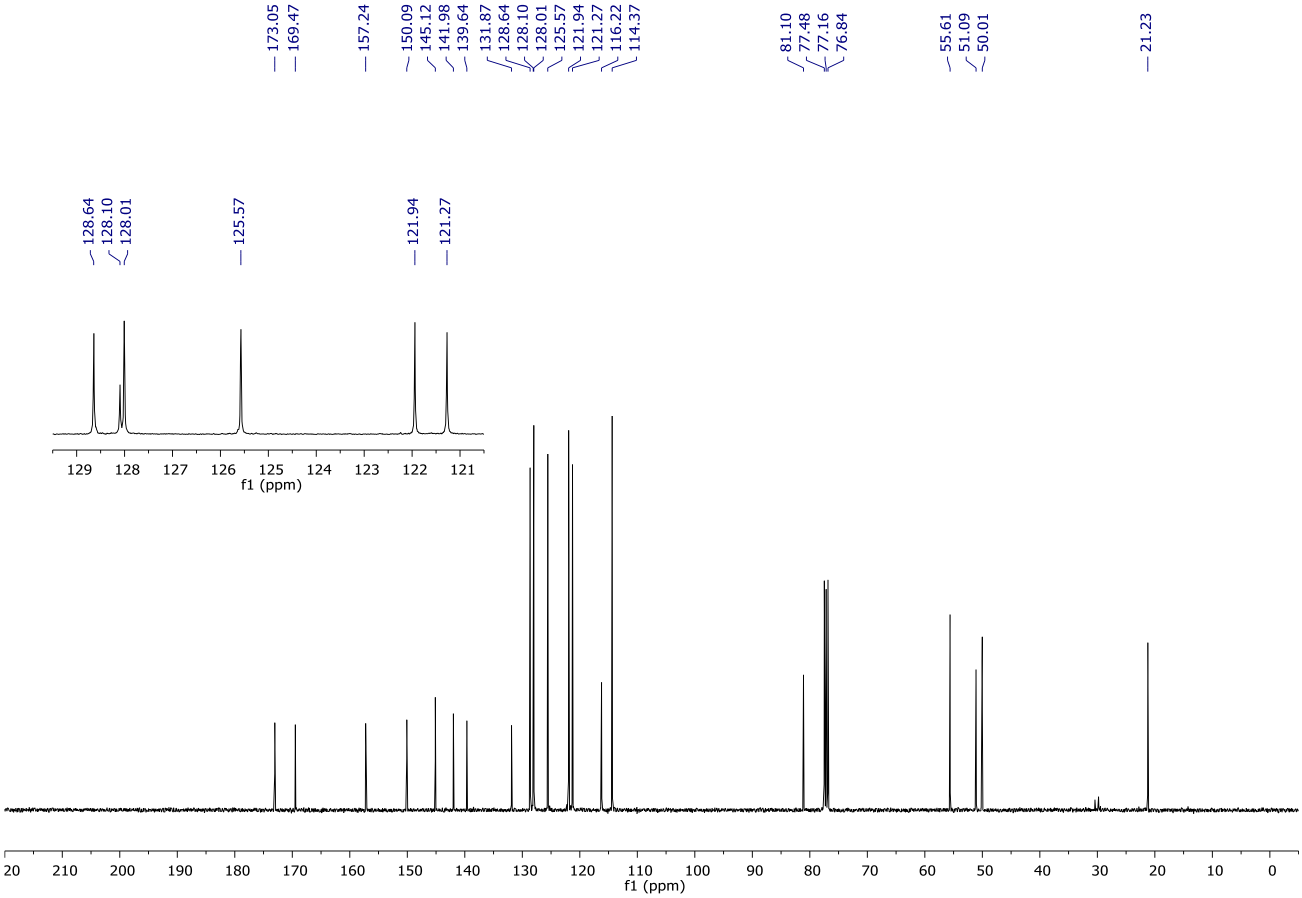
173.05
169.47
157.24
150.09
145.12
141.98
139.64
131.87
128.64
128.10
128.01
125.57
121.94
121.27
116.22
114.37
81.10
77.48
77.16
76.84
55.61
51.09
50.01
21.23
128.64
128.10
128.01
125.57
121.94
121.27
129 128 127 126 125 124 123 122 121
f1 (ppm)
210 200 190 180 170 160 150 140 130 120 110 100 90 80 70 60 50 40 30 20 10 0
f1 (ppm)

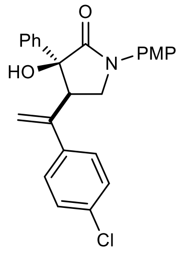
O
Ph
HO
N
PMP
Cl

**2h**

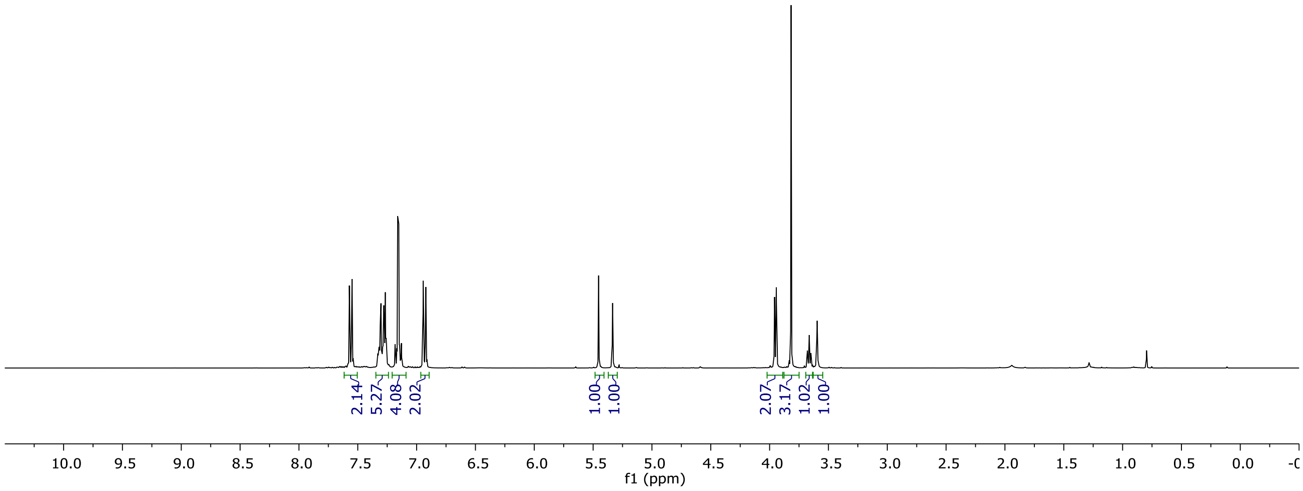
2.14
5.27
4.08
2.02
1.00
1.00
2.07
3.17
1.02
1.00
10.0 9.5 9.0 8.5 8.0 7.5 7.0 6.5 6.0 5.5 5.0 4.5 4.0 3.5 3.0 2.5 2.0 1.5 1.0 0.5 0.0
f1 (ppm)

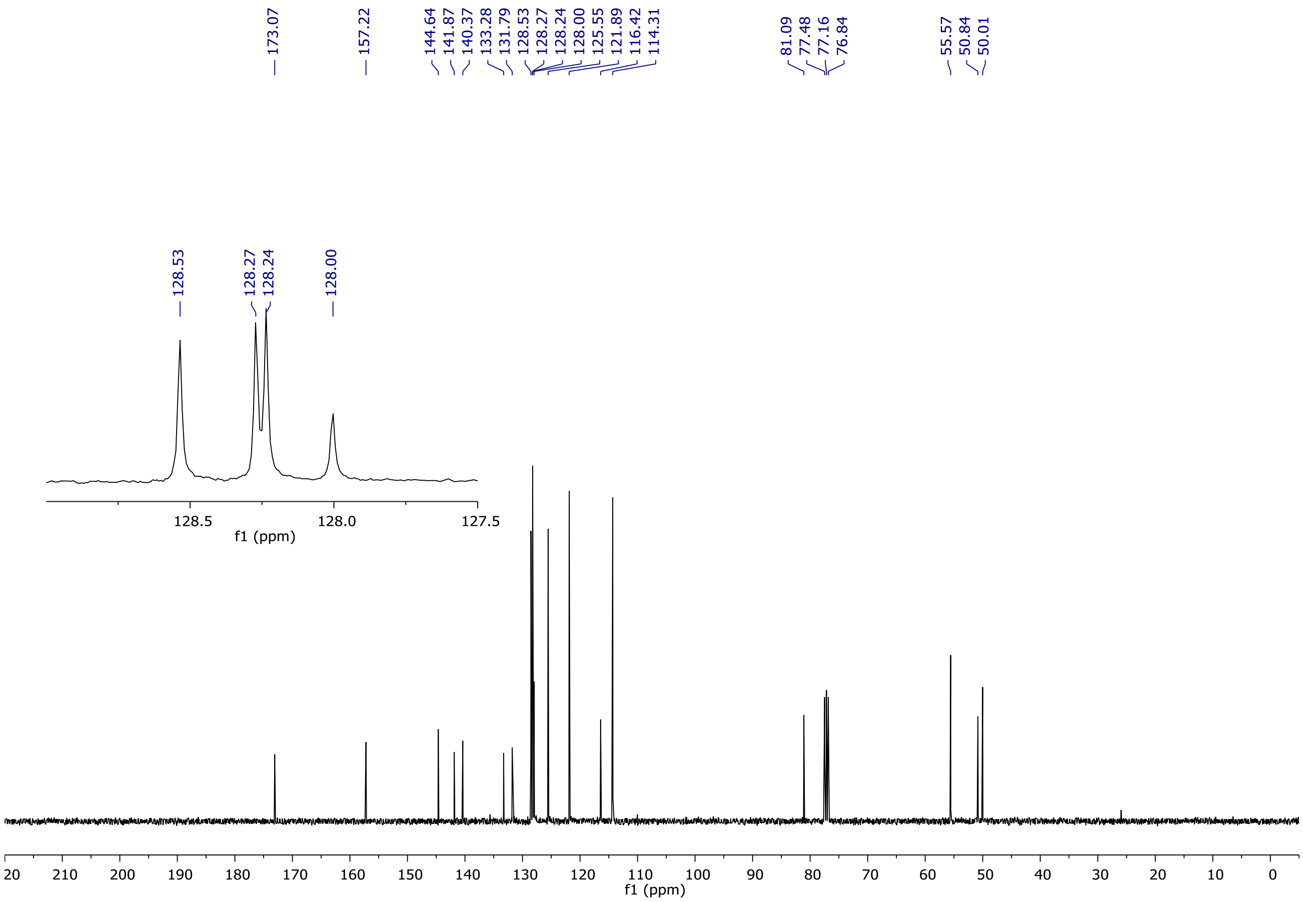
173.07
157.22
144.64
141.87
140.37
133.28
131.79
128.53
128.27
128.24
128.00
125.55
121.89
116.42
114.31
81.09
77.48
77.16
76.84
55.57
50.84
50.01
128.53
128.27
128.24
128.00
128.5 128.0 127.5
f1 (ppm)
20 210 200 190 180 170 160 150 140 130 120 110 100 90 80 70 60 50 40 30 20 10 0
f1 (ppm)

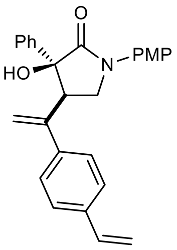
O
Ph
HO
N
PMP

**2i**

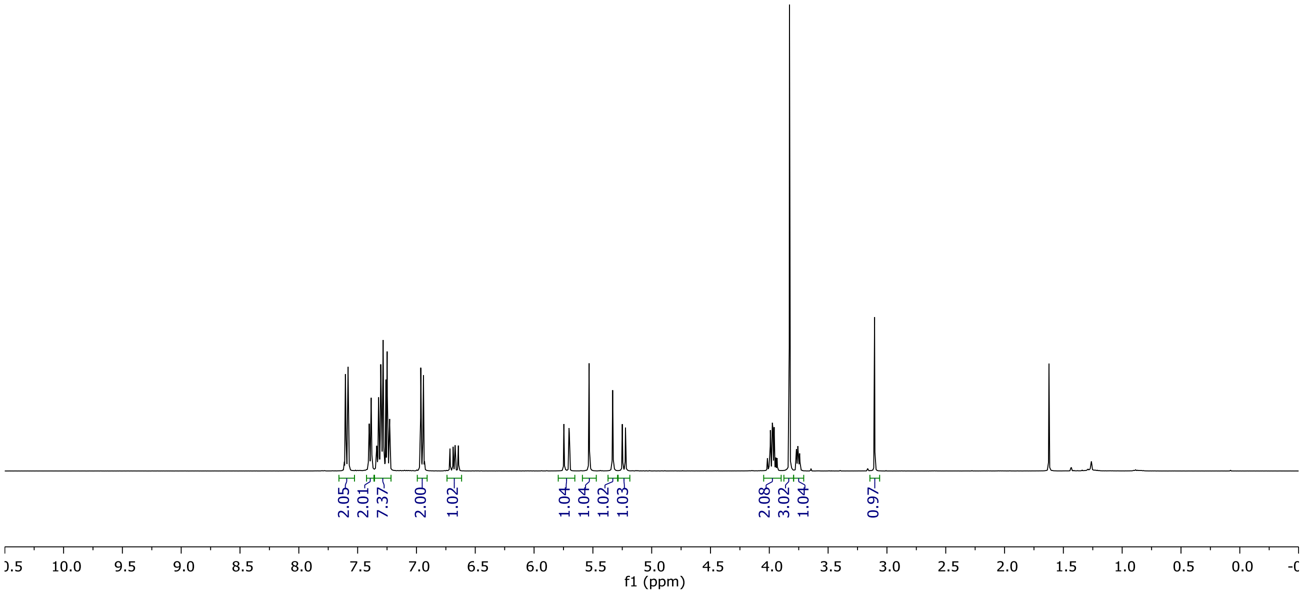
2.05
2.01
7.37
2.00
1.02
1.04
1.04
1.02
1.03
2.08
3.02
1.04
0.97
f1 (ppm)

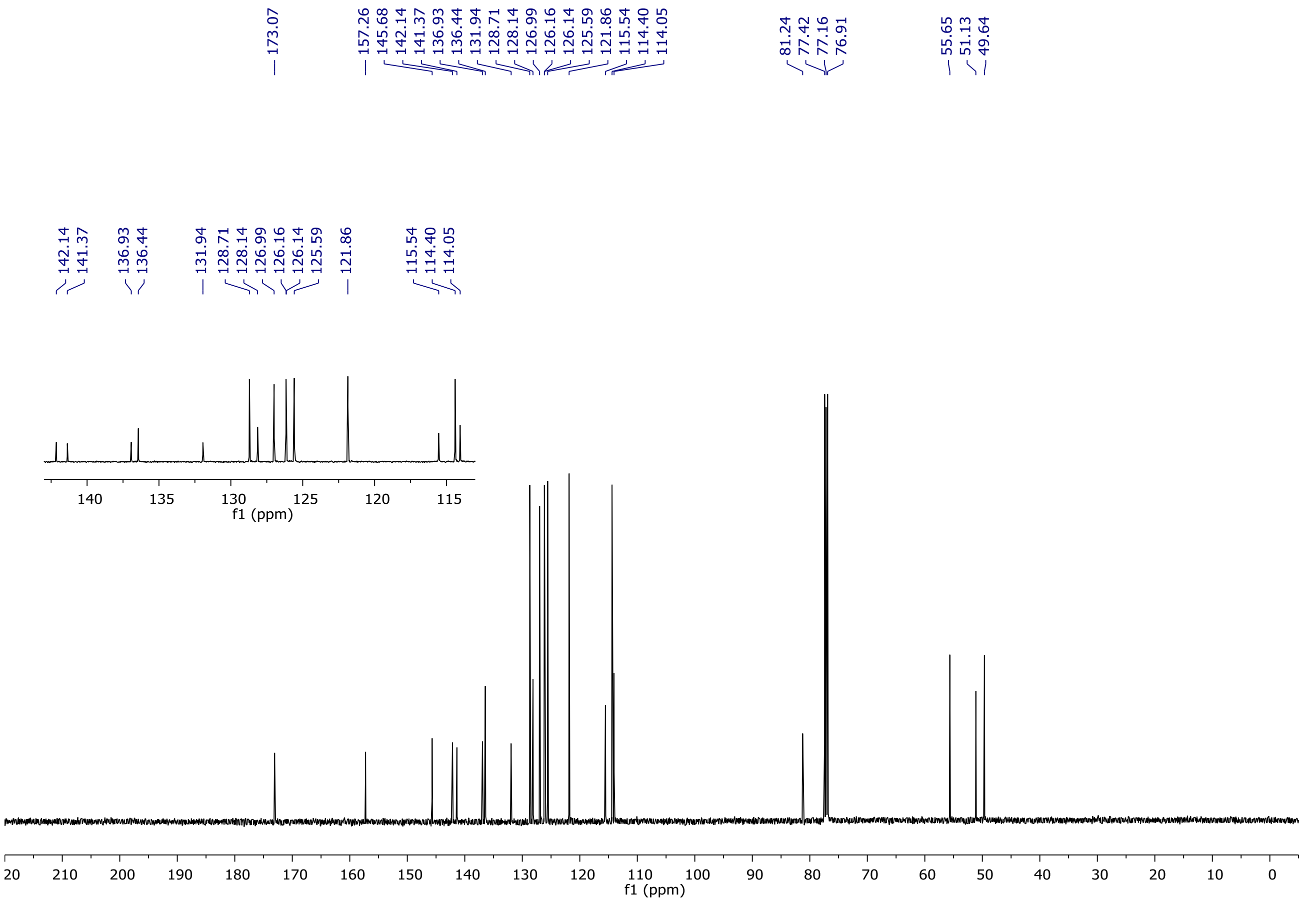
173.07
157.26
145.68
142.14
141.37
136.93
136.44
131.94
128.71
128.14
126.99
126.16
126.14
125.59
121.86
115.54
114.40
114.05
81.24
77.42
77.16
76.91
55.65
51.13
49.64
f1 (ppm)

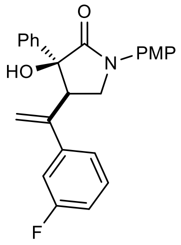
O
Ph
HO
N
PMP
F

**2j**

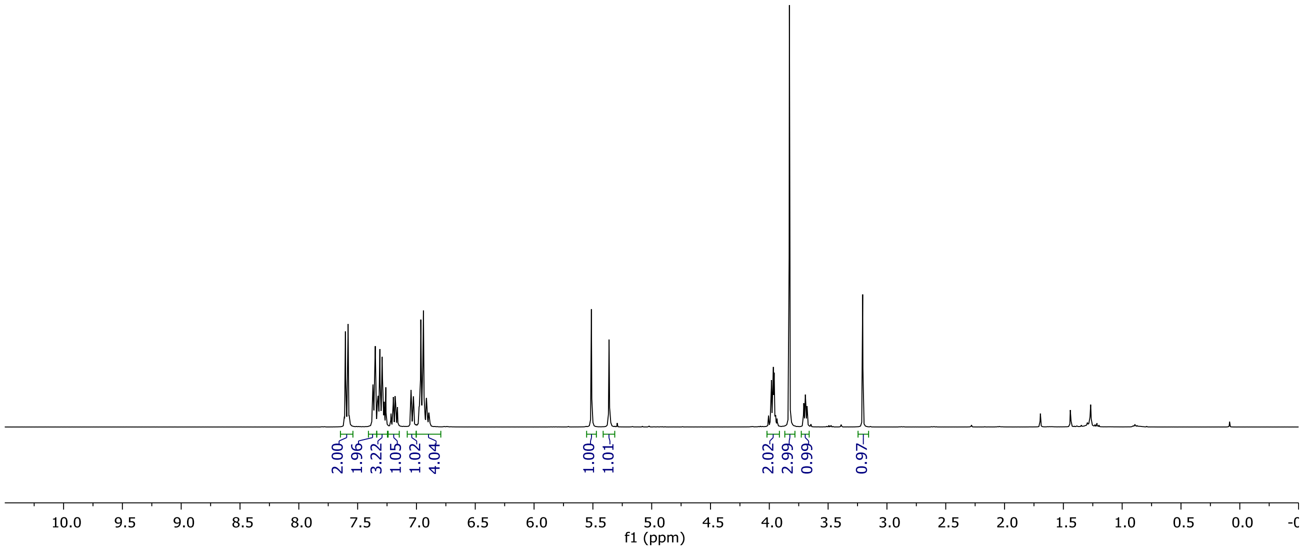
2.00
1.96
3.22
1.05
1.02
4.04
1.00
1.01
2.02
2.99
0.99
0.97
10.0 9.5 9.0 8.5 8.0 7.5 7.0 6.5 6.0 5.5 5.0 4.5 4.0 3.5 3.0 2.5 2.0 1.5 1.0 0.5 0.0
f1 (ppm)

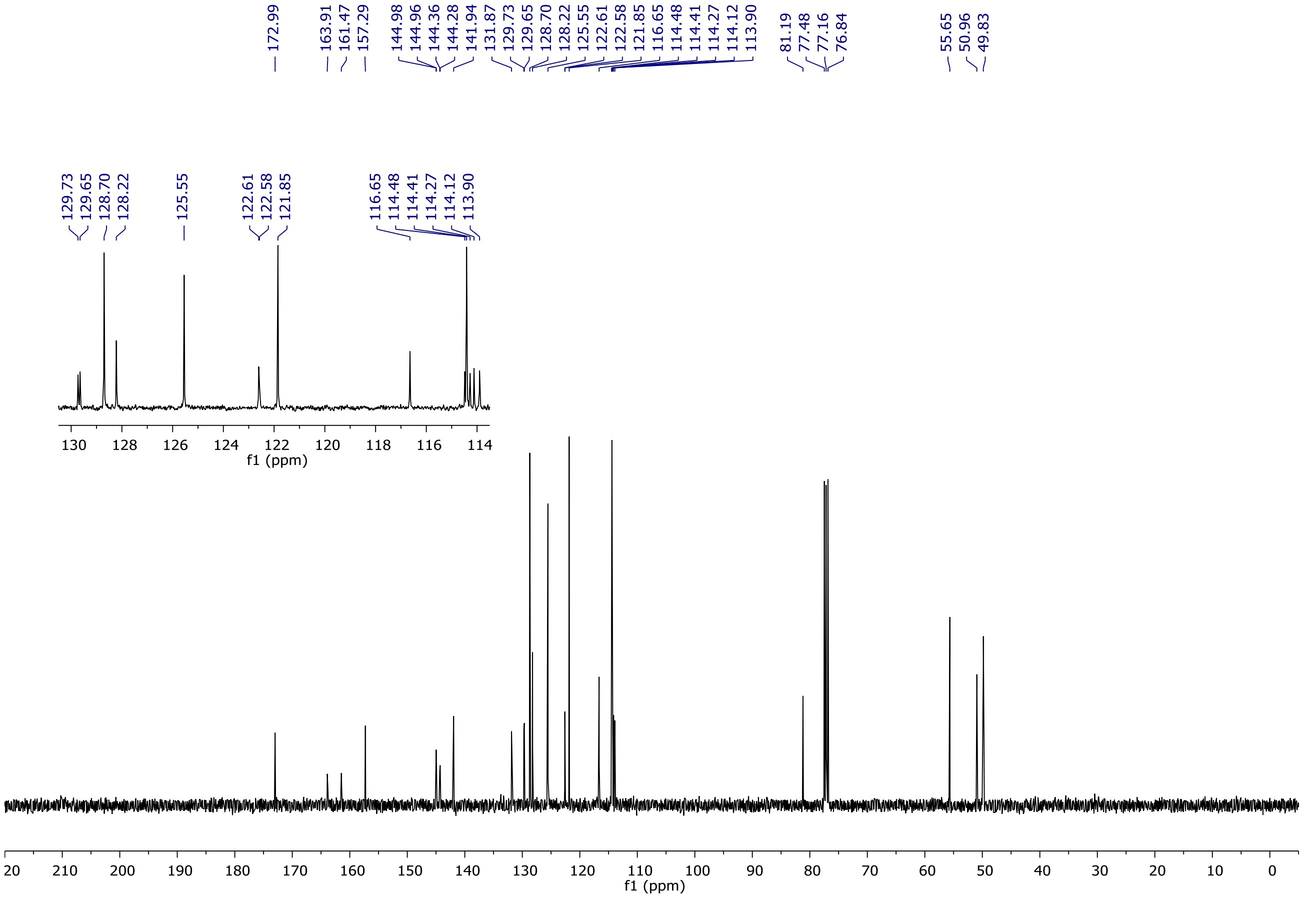
172.99
163.91
161.47
157.29
144.98
144.96
144.36
144.28
141.94
131.87
129.73
129.65
128.70
128.22
125.55
122.61
122.58
121.85
116.65
114.48
114.41
114.27
114.12
113.90
81.19
77.48
77.16
76.84
55.65
50.96
49.83
130 128 126 124 122 120 118 116 114
f1 (ppm)
210 200 190 180 170 160 150 140 130 120 110 100 90 80 70 60 50 40 30 20 10 0
f1 (ppm)

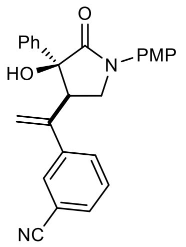
Ph
HO
O
N
PMP
NC

**2k**

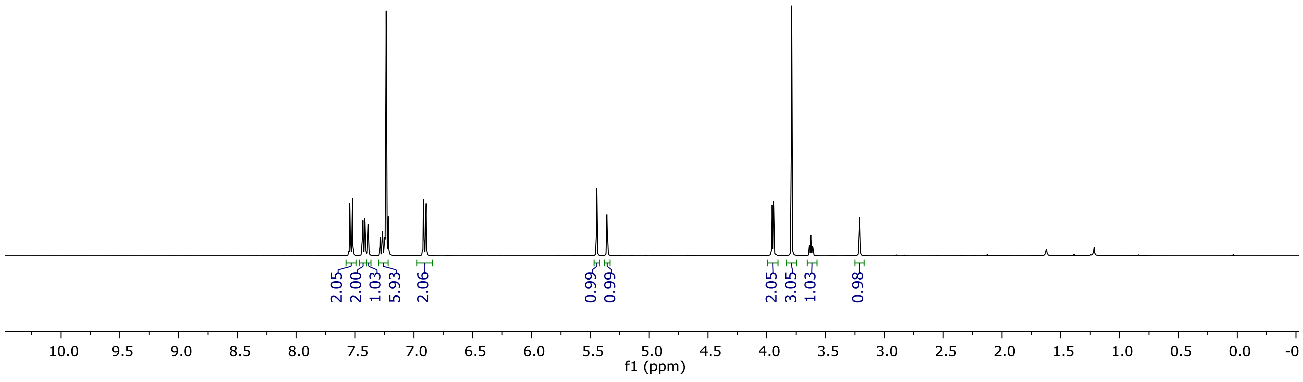
2.05
2.00
1.03
5.93
2.06
0.99
0.99
2.05
3.05
1.03
0.98
10.0 9.5 9.0 8.5 8.0 7.5 7.0 6.5 6.0 5.5 5.0 4.5 4.0 3.5 3.0 2.5 2.0 1.5 1.0 0.5 0.0 -0
f1 (ppm)

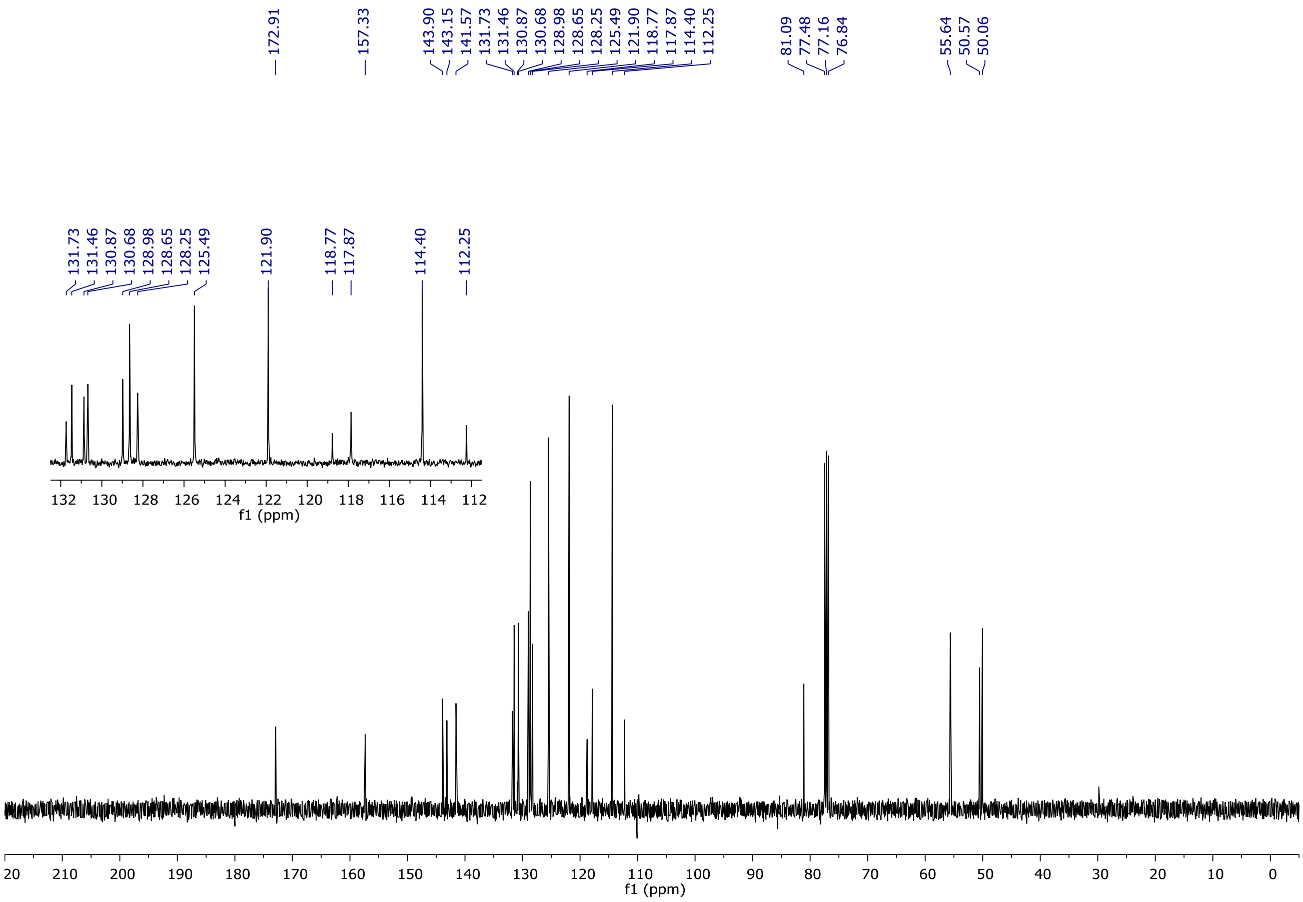
172.91
157.33
143.90
143.15
141.57
131.73
131.46
130.87
130.68
128.98
128.65
128.25
125.49
121.90
118.77
117.87
114.40
112.25
81.09
77.48
77.16
76.84
55.64
50.57
50.06
132 130 128 126 124 122 120 118 116 114 112
f1 (ppm)
20 210 200 190 180 170 160 150 140 130 120 110 100 90 80 70 60 50 40 30 20 10 0
f1 (ppm)

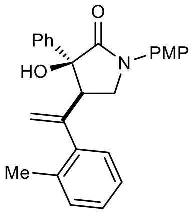
O
Ph
HO
N
PMP
Me

**2l**

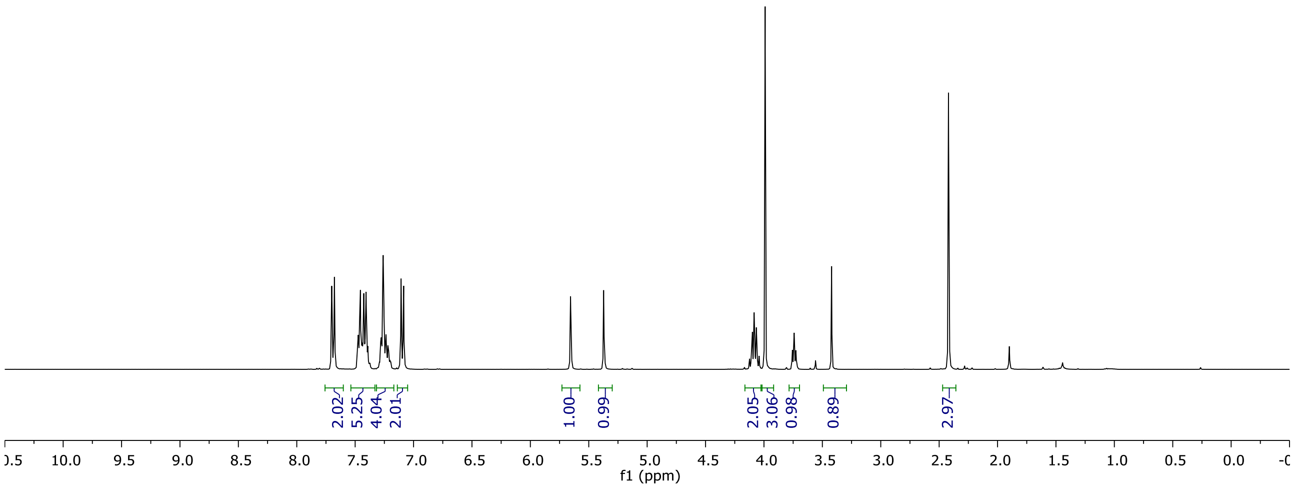
2.02
5.25
4.04
2.01
1.00
0.99
2.05
3.06
0.98
0.89
2.97
f1 (ppm)

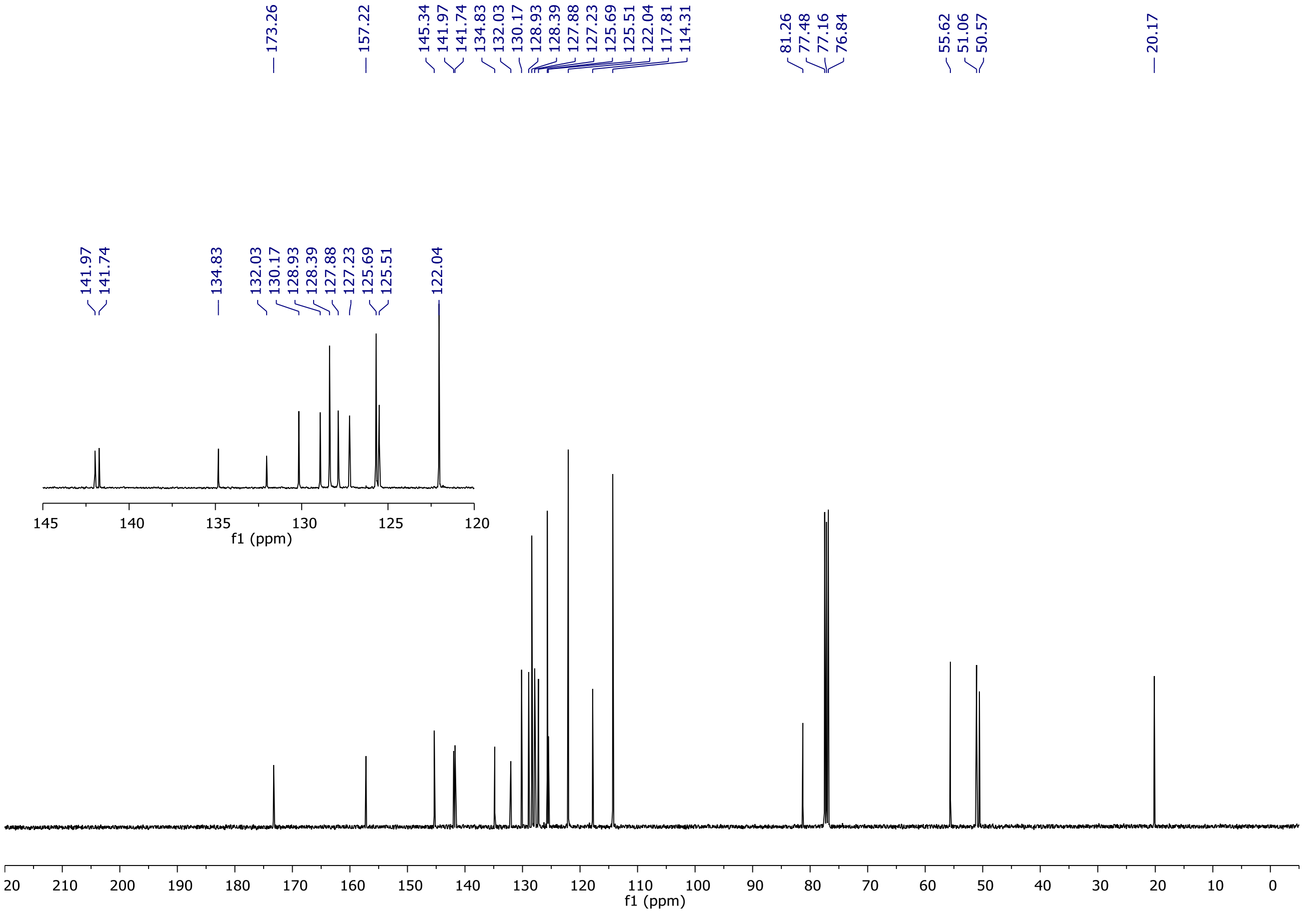
173.26
157.22
145.34
141.97
141.74
134.83
132.03
130.17
128.93
128.39
127.88
127.23
125.69
125.51
122.04
117.81
114.31
81.26
77.48
77.16
76.84
55.62
51.06
50.57
20.17
f1 (ppm)

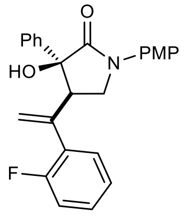
O
Ph
HO
N
PMP
F

**2m**

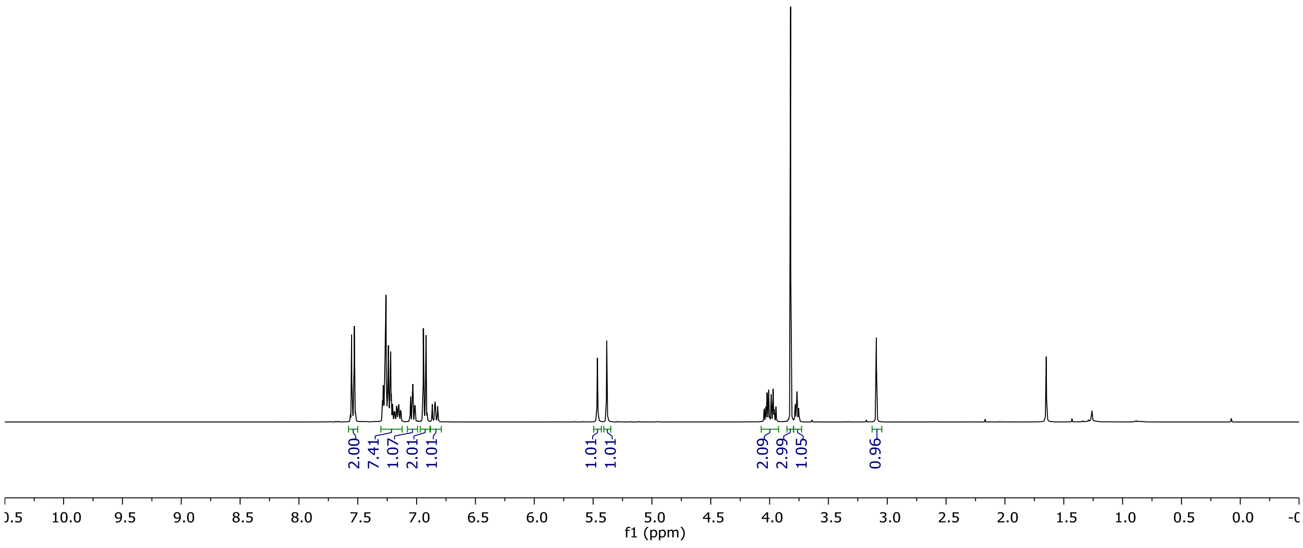
2.00
7.41
1.07
2.01
1.01
1.01
1.01
2.09
2.99
1.05
0.96
f1 (ppm)

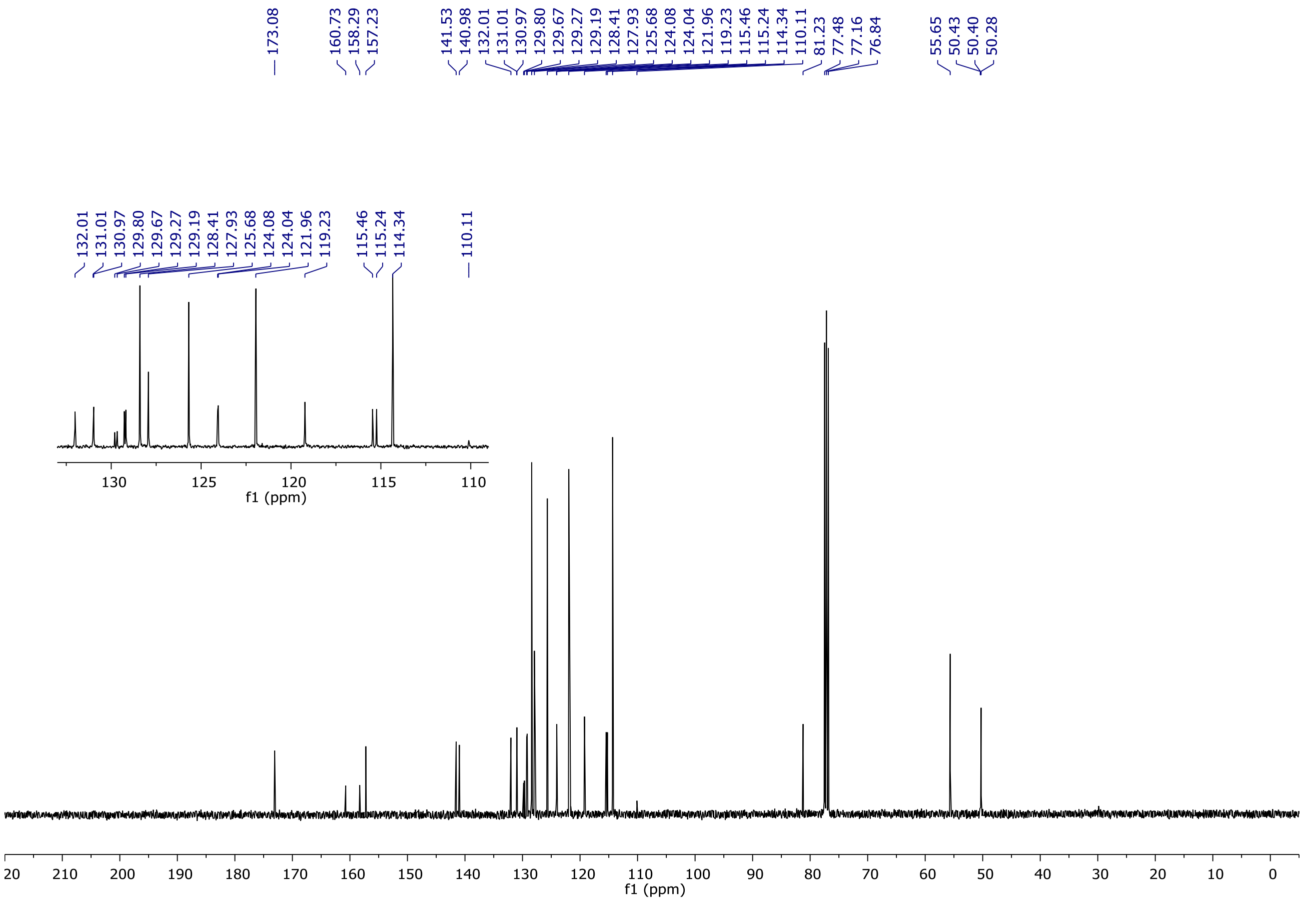
173.08
160.73
158.29
157.23
141.53
140.98
132.01
131.01
130.97
129.80
129.67
129.27
129.19
128.41
127.93
125.68
124.08
124.04
121.96
119.23
115.46
115.24
114.34
110.11
81.23
77.48
77.16
76.84
55.65
50.43
50.40
50.28
f1 (ppm)
f1 (ppm)

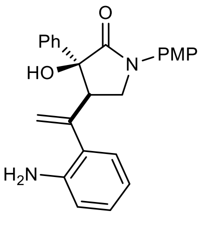

Ph
HO
O
N
PMP
H2N


**2n**

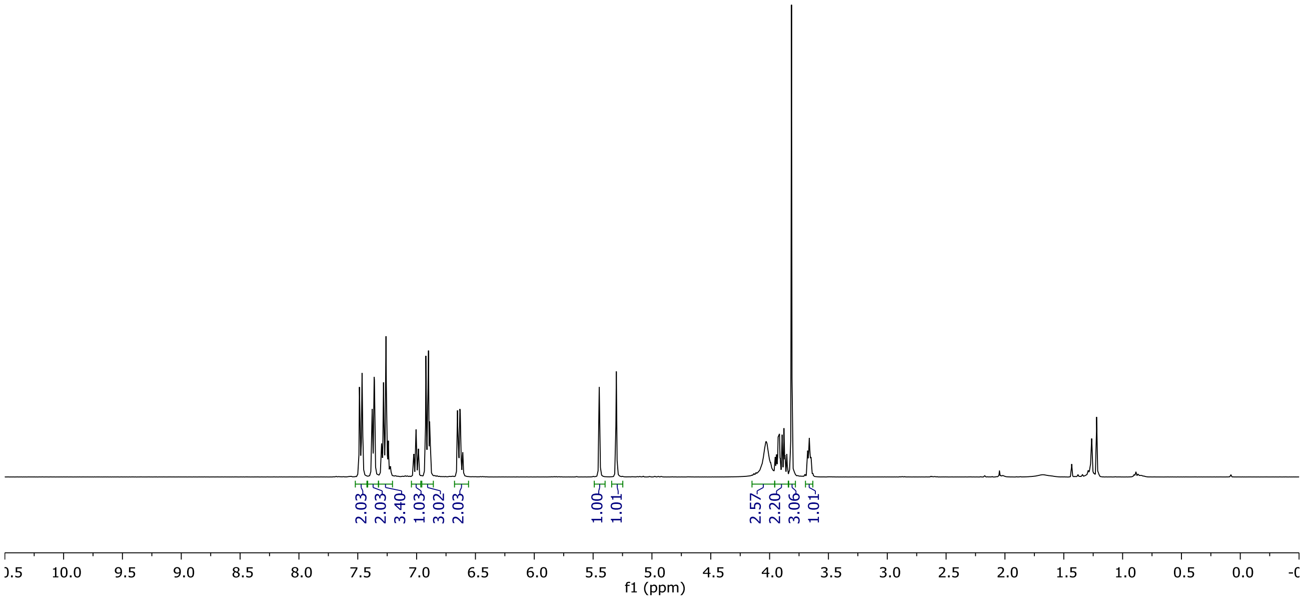

2.03
2.03
3.40
1.03
3.02
2.03
1.00
1.01
2.57
2.20
3.06
1.01
f1 (ppm)


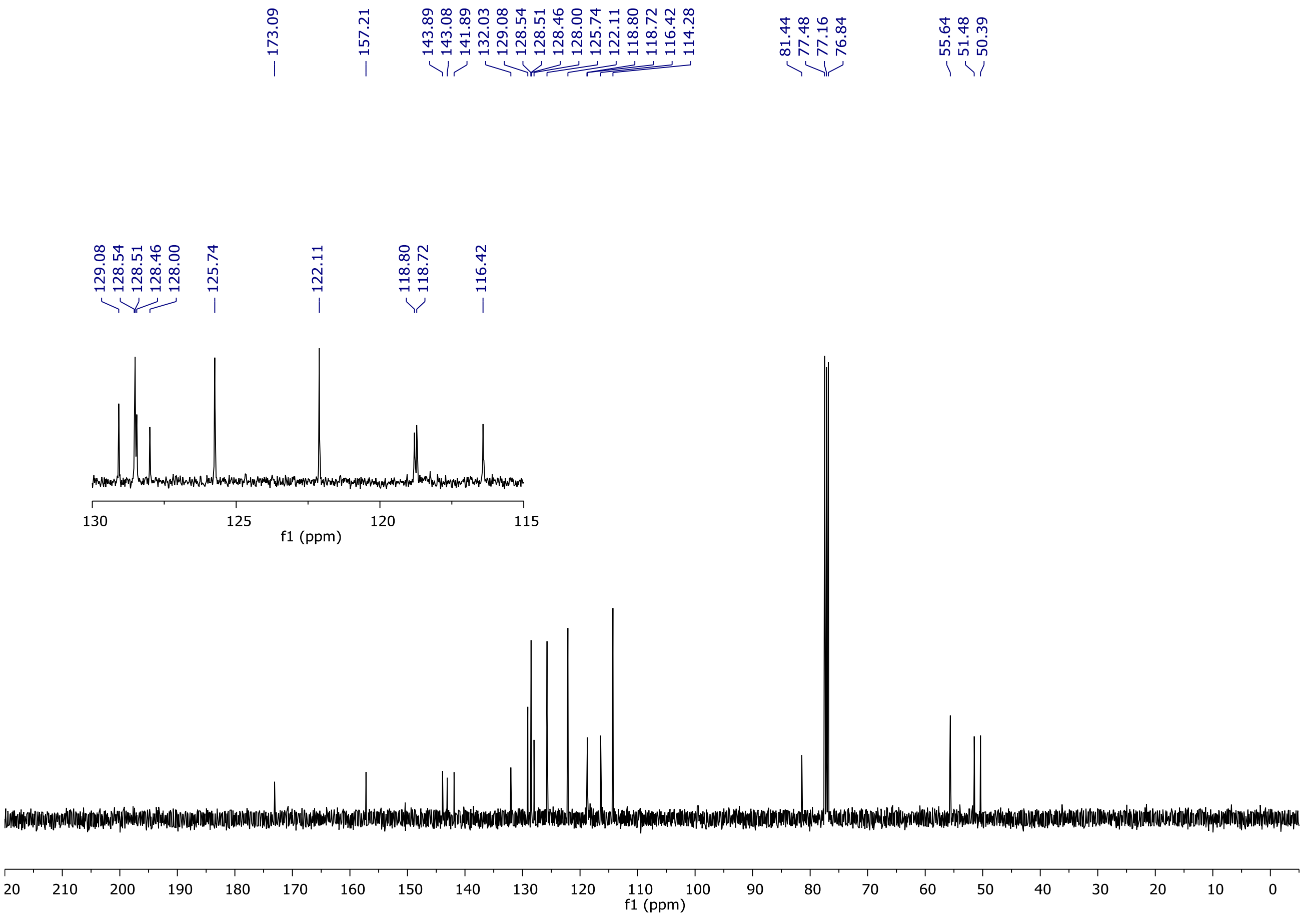

173.09
157.21
143.89
143.08
141.89
132.03
129.08
128.54
128.51
128.46
128.00
125.74
122.11
118.80
118.72
116.42
114.28
81.44
77.48
77.16
76.84
55.64
51.48
50.39
f1 (ppm)

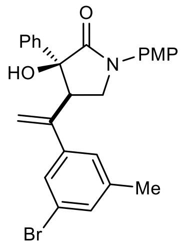
O
Ph
HO
N
PMP
Me
Br

**2o**

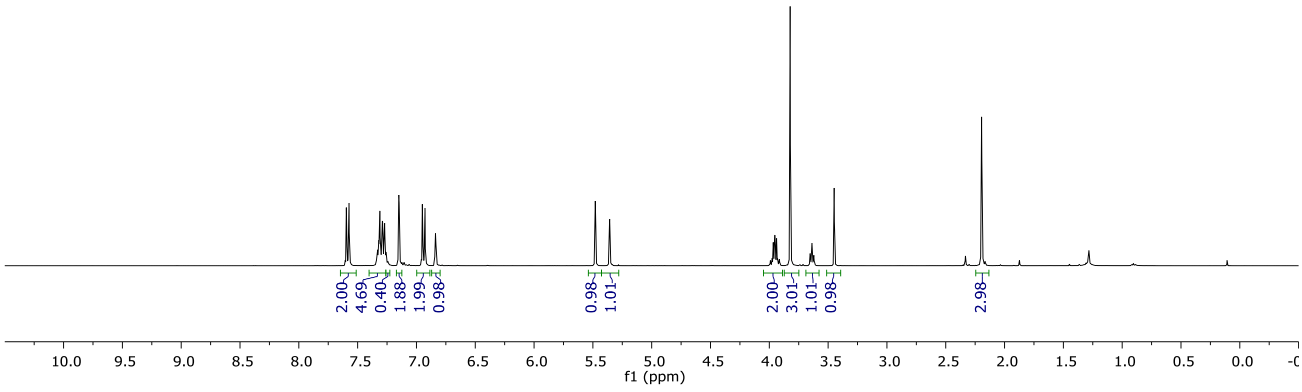
2.00
4.69
0.40
1.88
1.99
0.98
0.98
1.01
2.00
3.01
1.01
0.98
2.98
10.0 9.5 9.0 8.5 8.0 7.5 7.0 6.5 6.0 5.5 5.0 4.5 4.0 3.5 3.0 2.5 2.0 1.5 1.0 0.5 0.0
f1 (ppm)

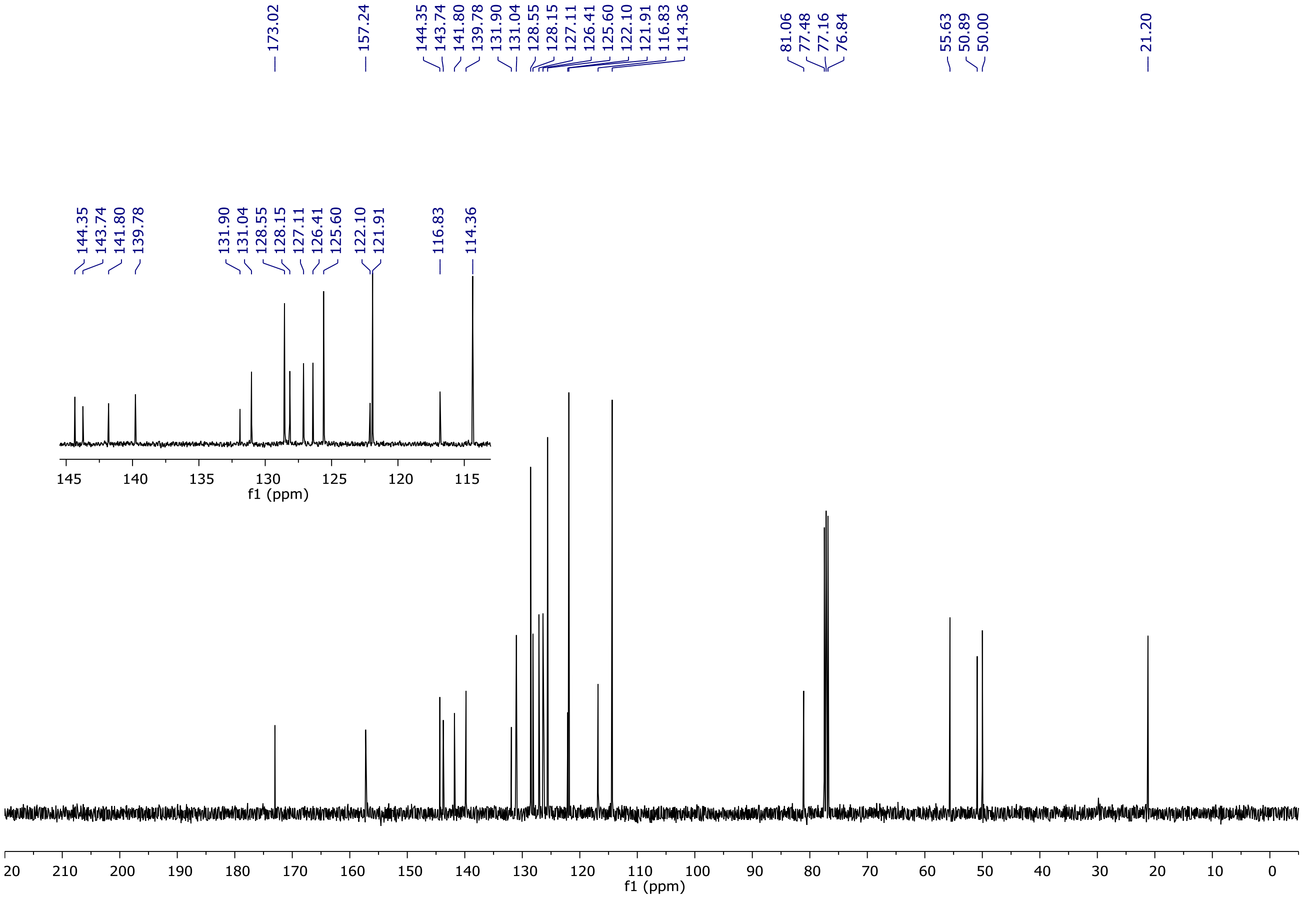
173.02
157.24
144.35
143.74
141.80
139.78
131.90
131.04
128.55
128.15
127.11
126.41
125.60
122.10
121.91
116.83
114.36
81.06
77.48
77.16
76.84
55.63
50.89
50.00
21.20
145 140 135 130 125 120 115
f1 (ppm)
210 200 190 180 170 160 150 140 130 120 110 100 90 80 70 60 50 40 30 20 10 0
f1 (ppm)

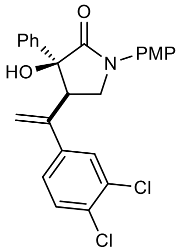
O
Ph
N
PMP
HO
Cl
Cl

**2p**

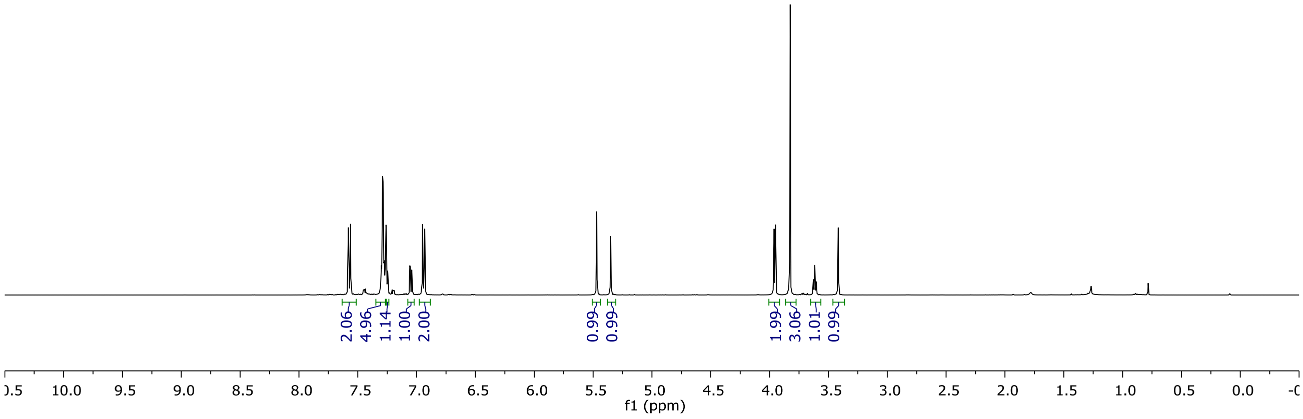
2.06
4.96
1.14
1.00
2.00
0.99
0.99
1.99
3.06
1.01
0.99
f1 (ppm)

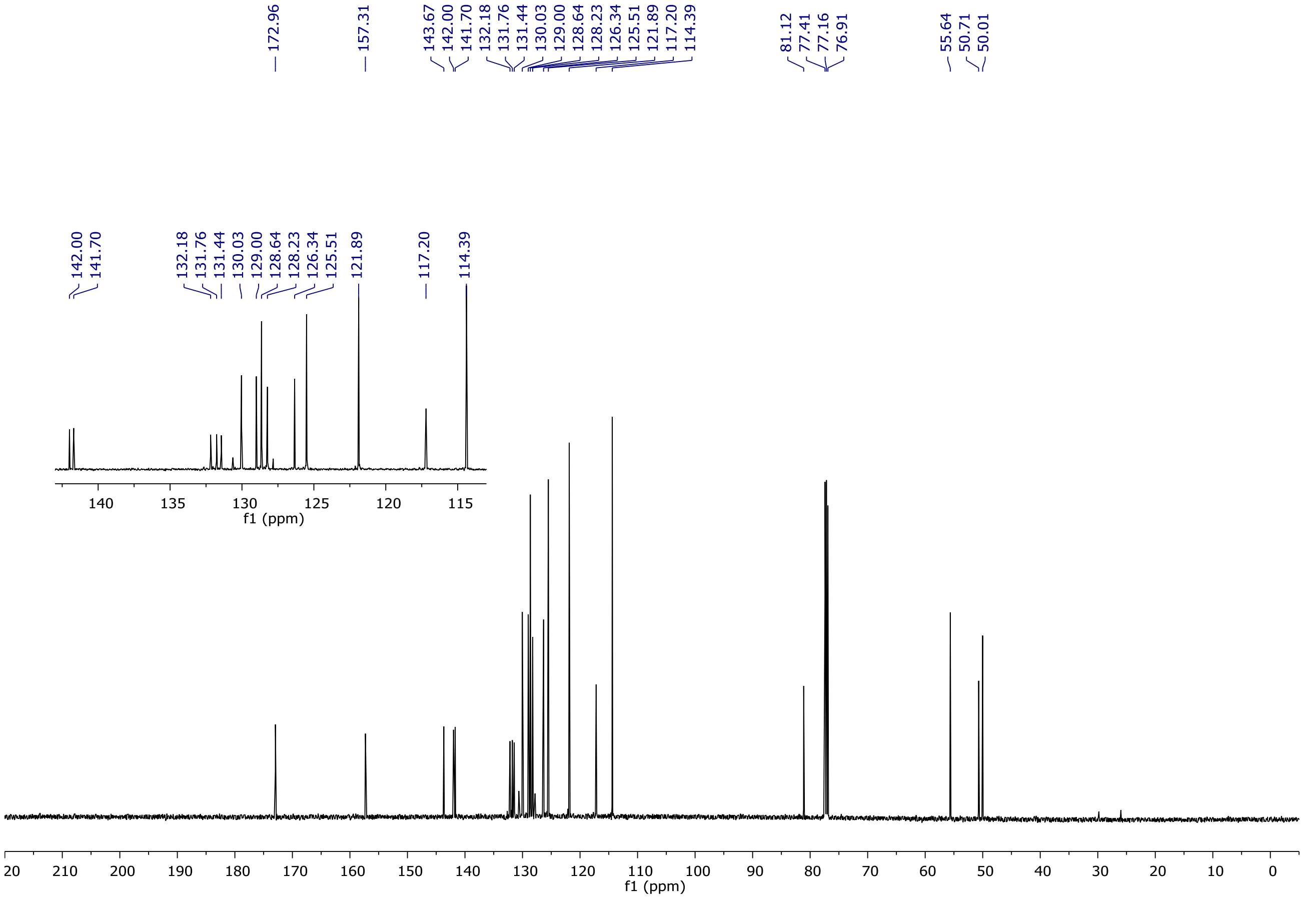
172.96
157.31
143.67
142.00
141.70
132.18
131.76
131.44
130.03
129.00
128.64
128.23
126.34
125.51
121.89
117.20
114.39
81.12
77.41
77.16
76.91
55.64
50.71
50.01
f1 (ppm)

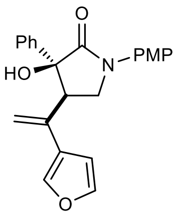
O
Ph
HO
N
PMP
O

**2q**

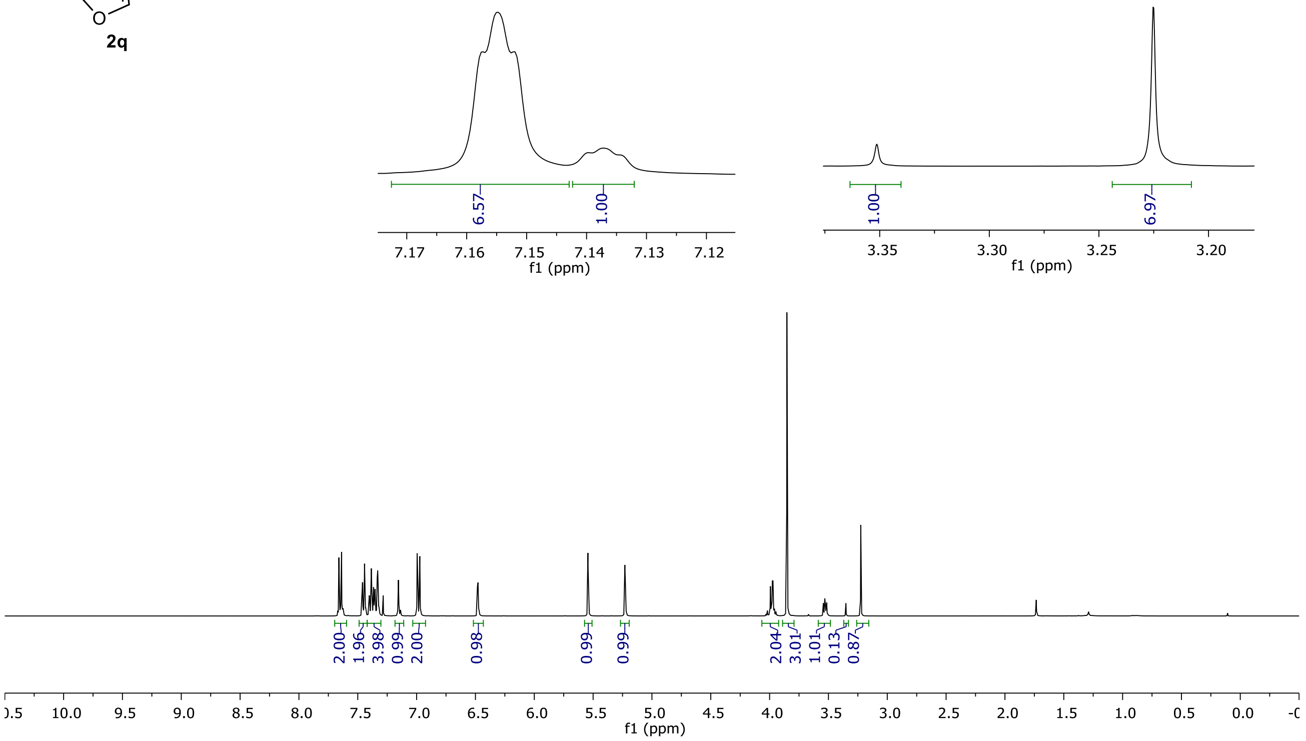
6.57
1.00
7.17 7.16 7.15 7.14 7.13 7.12
f1 (ppm)
1.00
6.97
3.35 3.30 3.25 3.20
f1 (ppm)
2.00 1.96 3.98 0.99 2.00 0.98 0.99 0.99 2.04 3.01 1.01 0.13 0.87
10.0 9.5 9.0 8.5 8.0 7.5 7.0 6.5 6.0 5.5 5.0 4.5 4.0 3.5 3.0 2.5 2.0 1.5 1.0 0.5 0.0
f1 (ppm)

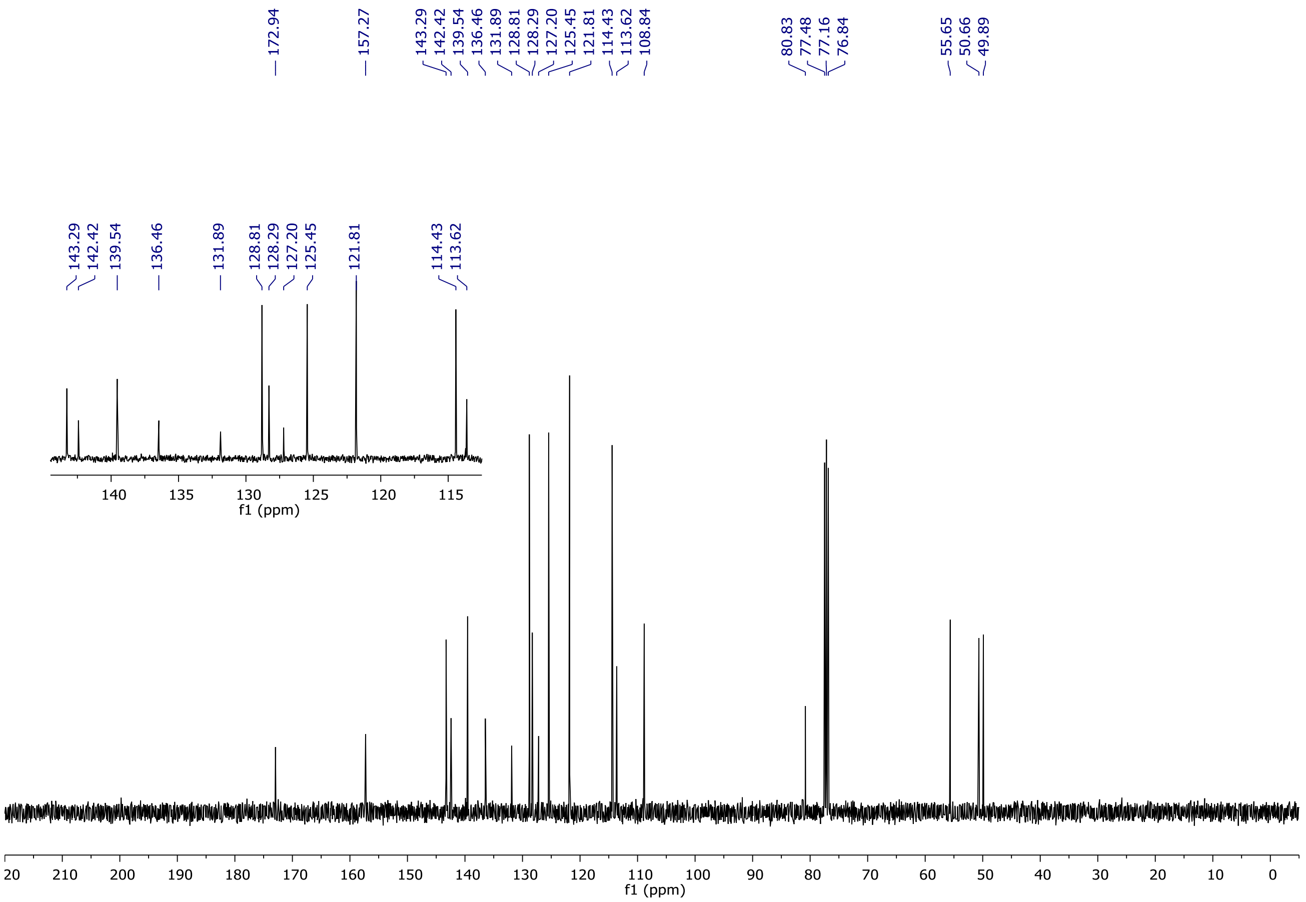
172.94
157.27
143.29 142.42 139.54 136.46 131.89 128.81 128.29 127.20 125.45 121.81 114.43 113.62 108.84
80.83 77.48 77.16 76.84
55.65 50.66 49.89
143.29 142.42 139.54 136.46 131.89 128.81 128.29 127.20 125.45 121.81 114.43 113.62
140 135 130 125 120 115
f1 (ppm)
210 200 190 180 170 160 150 140 130 120 110 100 90 80 70 60 50 40 30 20 10 0
f1 (ppm)

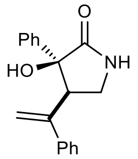
O
Ph
HO
NH
Ph

**2r**

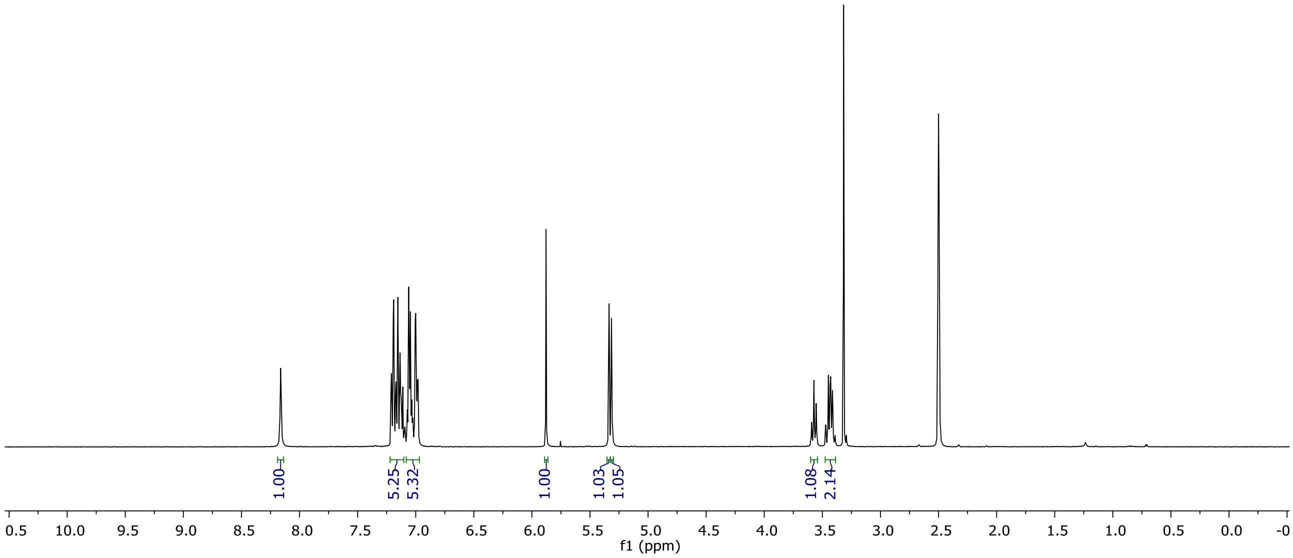
1.00
5.25
5.32
1.00
1.03
1.05
1.08
2.14
f1 (ppm)

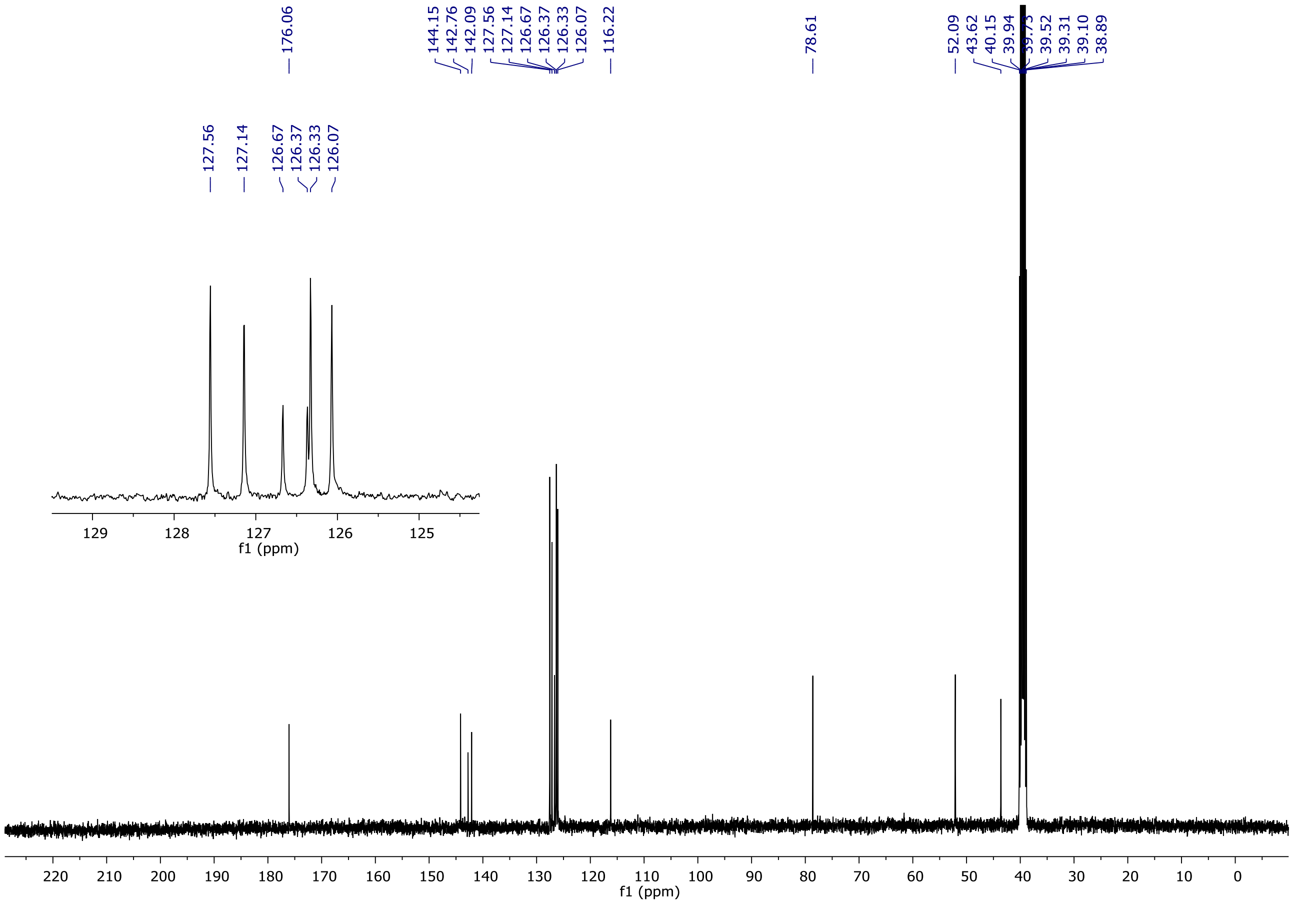
176.06
144.15
142.76
142.09
127.56
127.14
126.67
126.37
126.33
126.07
116.22
78.61
52.09
43.62
40.15
39.94
39.73
39.52
39.31
39.10
38.89
f1 (ppm)

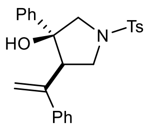
Ph
HO
N
Ts
Ph

**5a**

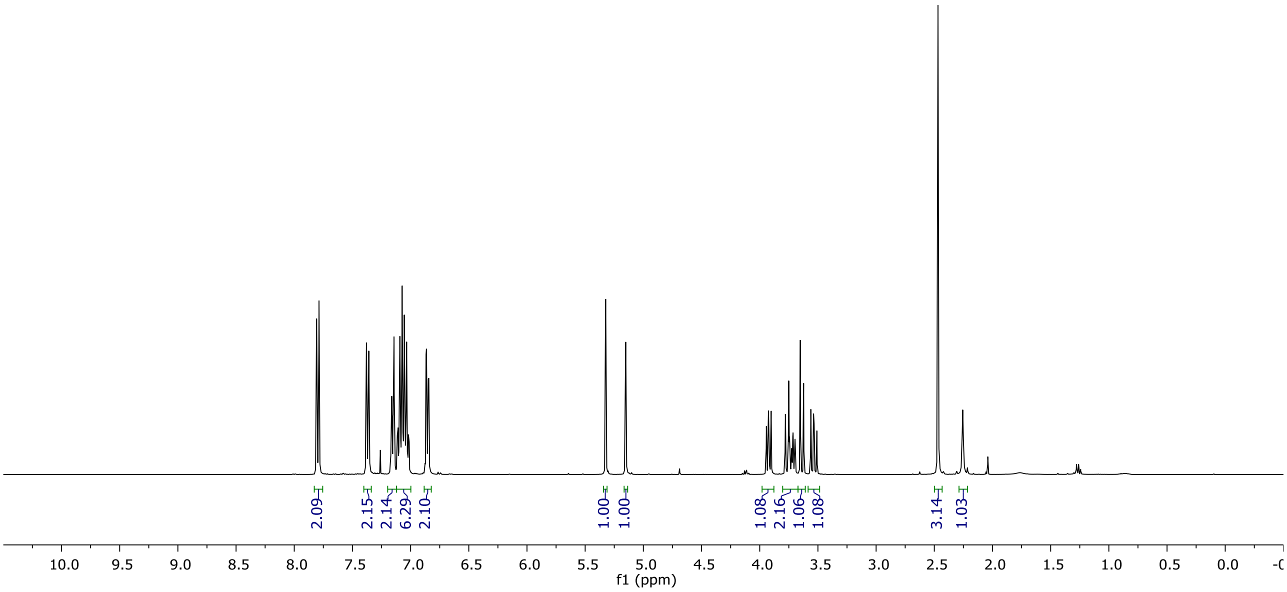
2.09
2.15
2.14
6.29
2.10
1.00
1.00
1.08
2.16
1.06
1.08
3.14
1.03
10.0 9.5 9.0 8.5 8.0 7.5 7.0 6.5 6.0 5.5 5.0 4.5 4.0 3.5 3.0 2.5 2.0 1.5 1.0 0.5 0.0
f1 (ppm)

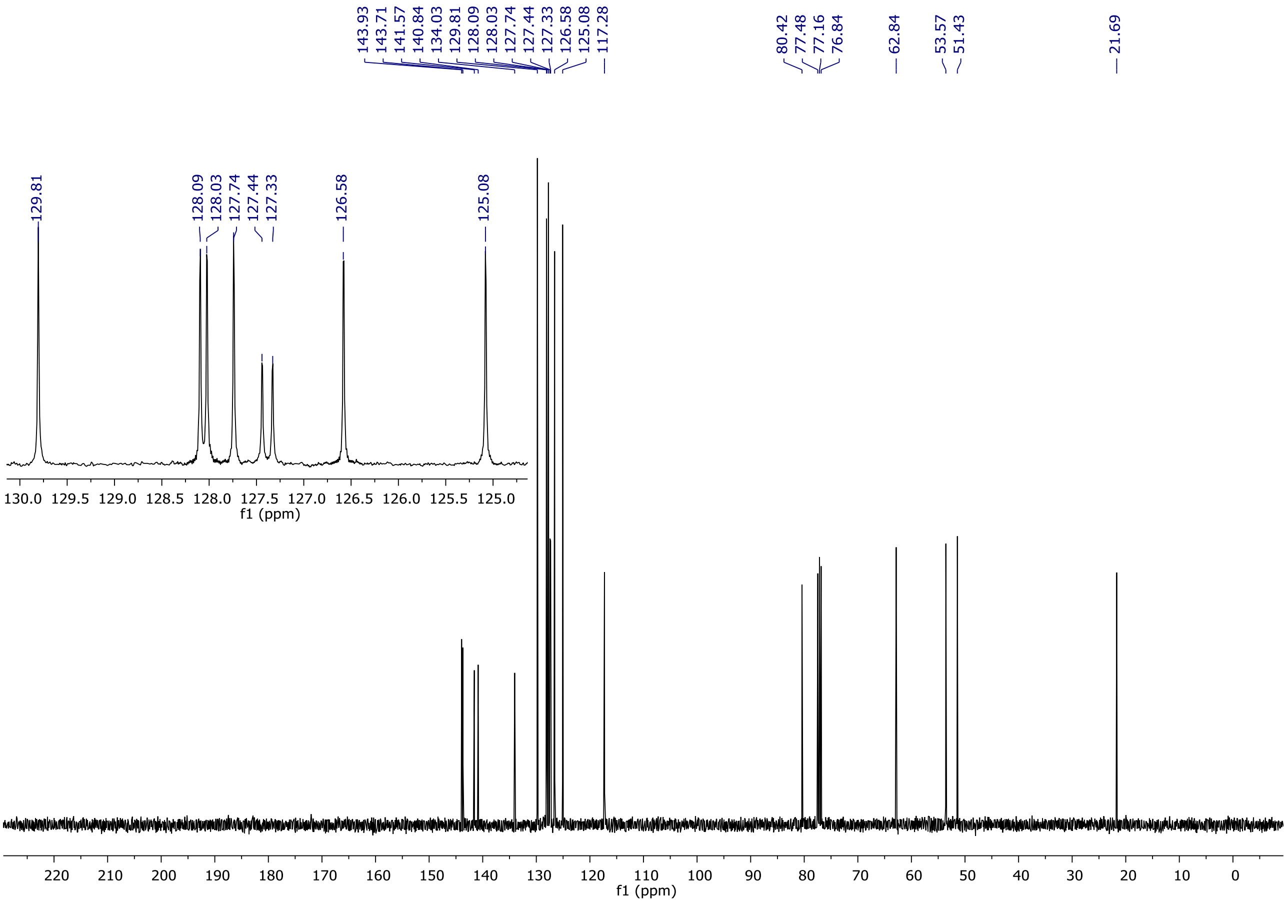
143.93
143.71
141.57
140.84
134.03
129.81
128.09
128.03
127.74
127.44
127.33
126.58
125.08
117.28
80.42
77.48
77.16
76.84
62.84
53.57
51.43
21.69
129.81
128.09
128.03
127.74
127.44
127.33
126.58
125.08
130.0 129.5 129.0 128.5 128.0 127.5 127.0 126.5 126.0 125.5 125.0
f1 (ppm)
220 210 200 190 180 170 160 150 140 130 120 110 100 90 80 70 60 50 40 30 20 10 0
f1 (ppm)

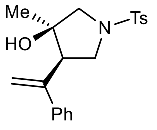
Me
HO
N
Ts
Ph

**5b**

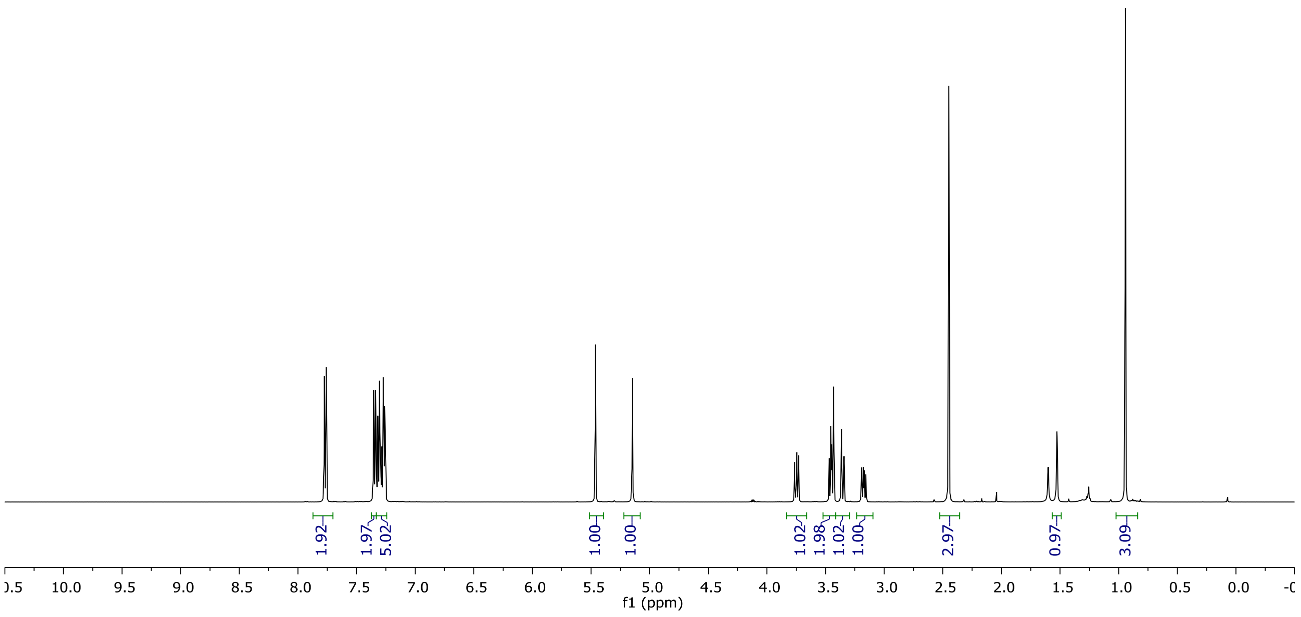
1.92
1.97
5.02
1.00
1.00
1.02
1.98
1.02
1.00
2.97
0.97
3.09
f1 (ppm)

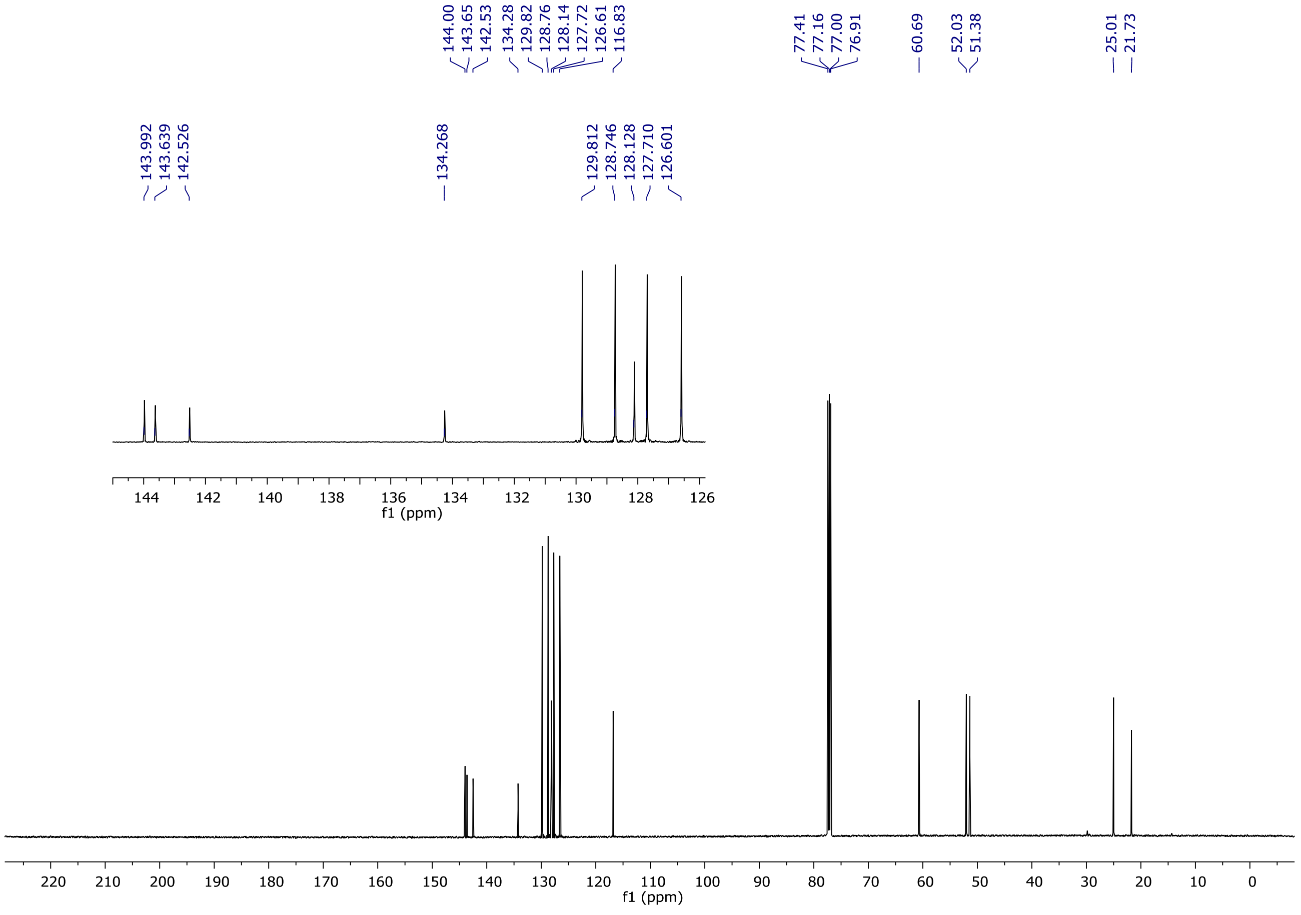
144.00
143.65
142.53
134.28
129.82
128.76
128.14
127.72
126.61
116.83
77.41
77.16
77.00
76.91
60.69
52.03
51.38
25.01
21.73
143.992
143.639
142.526
134.268
129.812
128.746
128.128
127.710
126.601
f1 (ppm)
f1 (ppm)

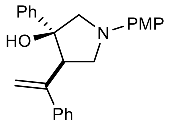
Ph
HO
N
PMP
Ph

**5c**

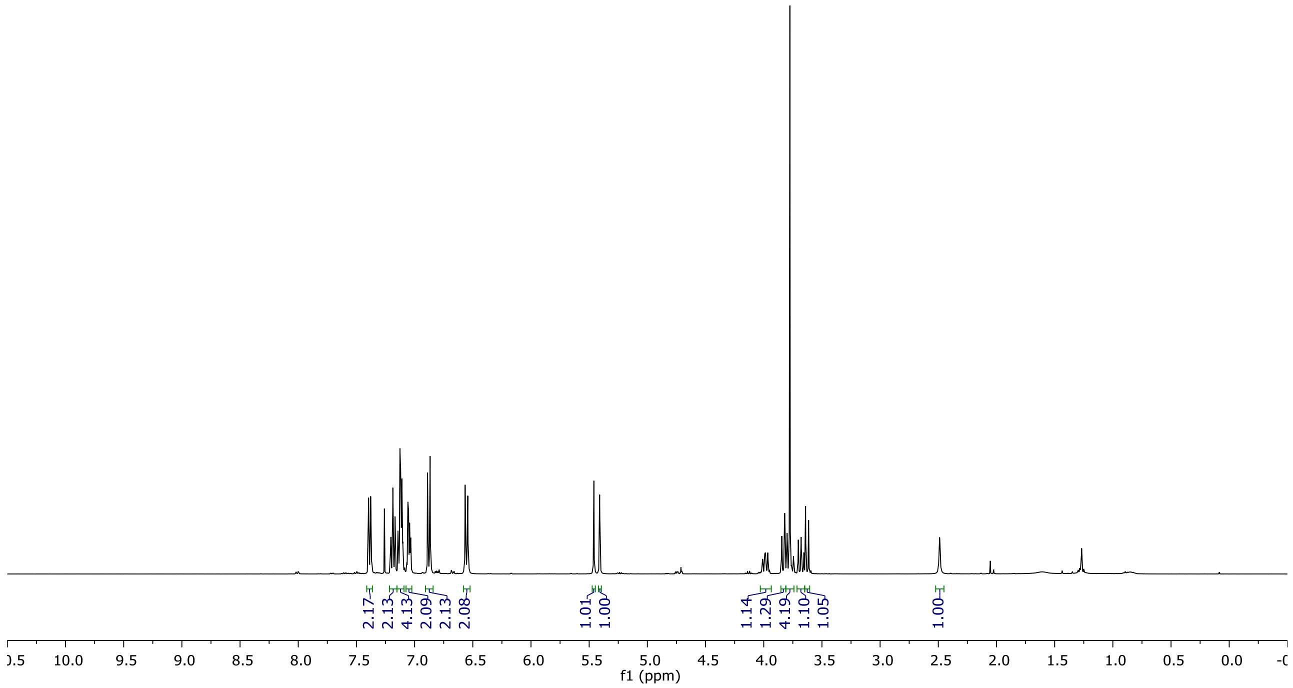
2.17
2.13
4.13
2.09
2.13
2.08
1.01
1.00
1.14
1.29
4.19
1.10
1.05
1.00
f1 (ppm)

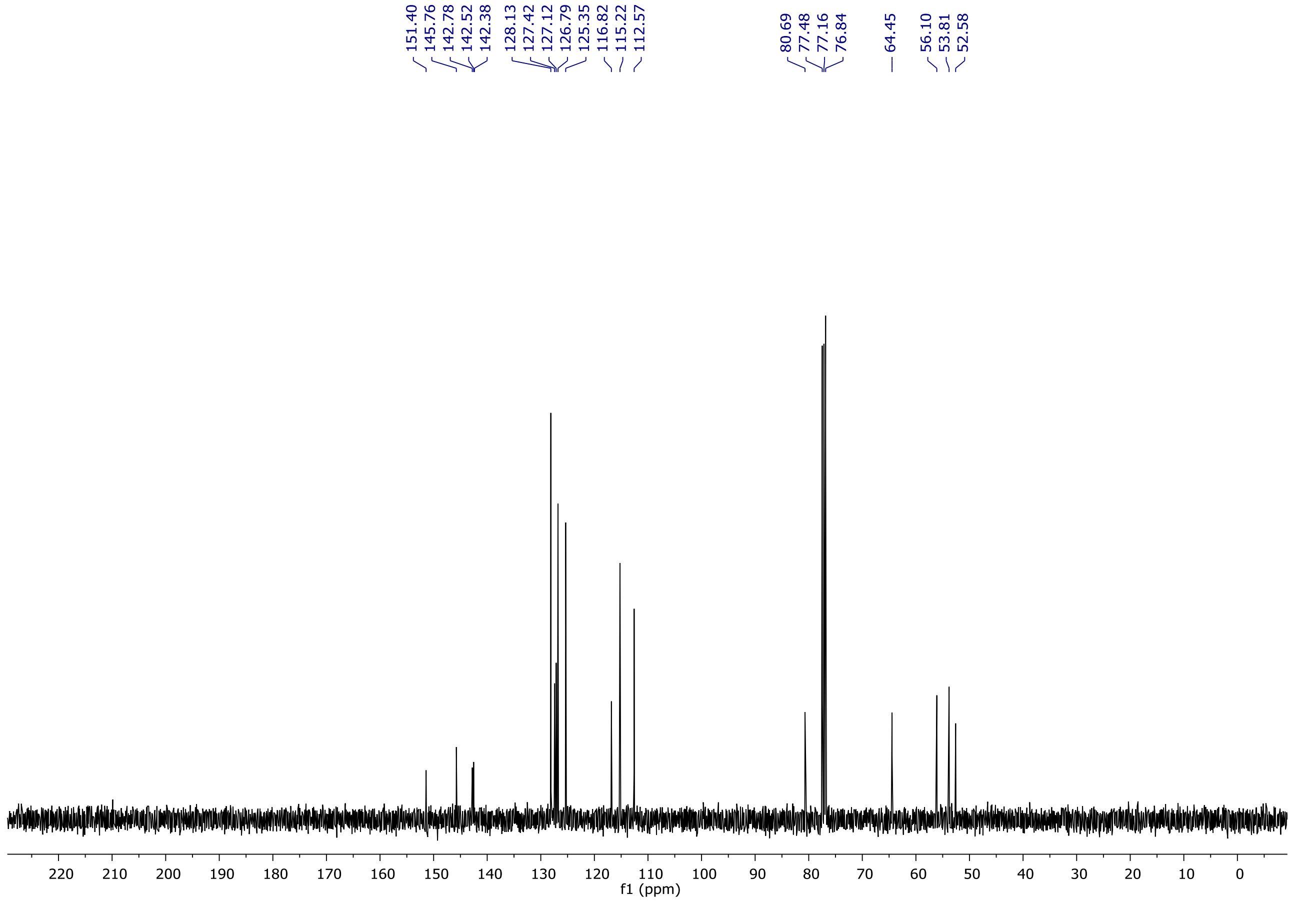
151.40
145.76
142.78
142.52
142.38
128.13
127.42
127.12
126.79
125.35
116.82
115.22
112.57
80.69
77.48
77.16
76.84
64.45
56.10
53.81
52.58
f1 (ppm)

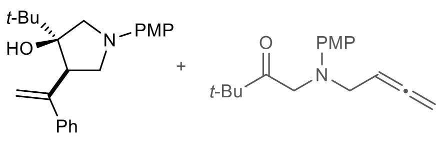


**5d** **4d**

7.7:1 inseparable mixture

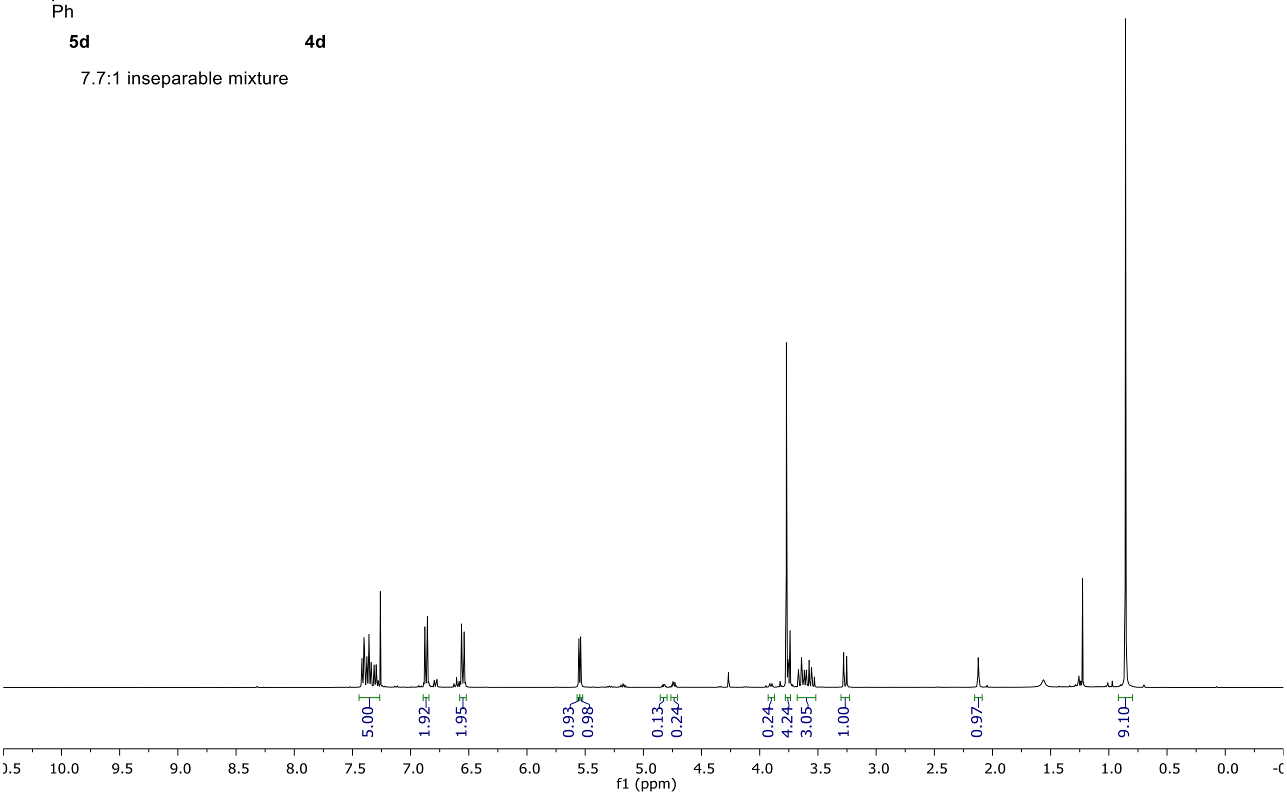


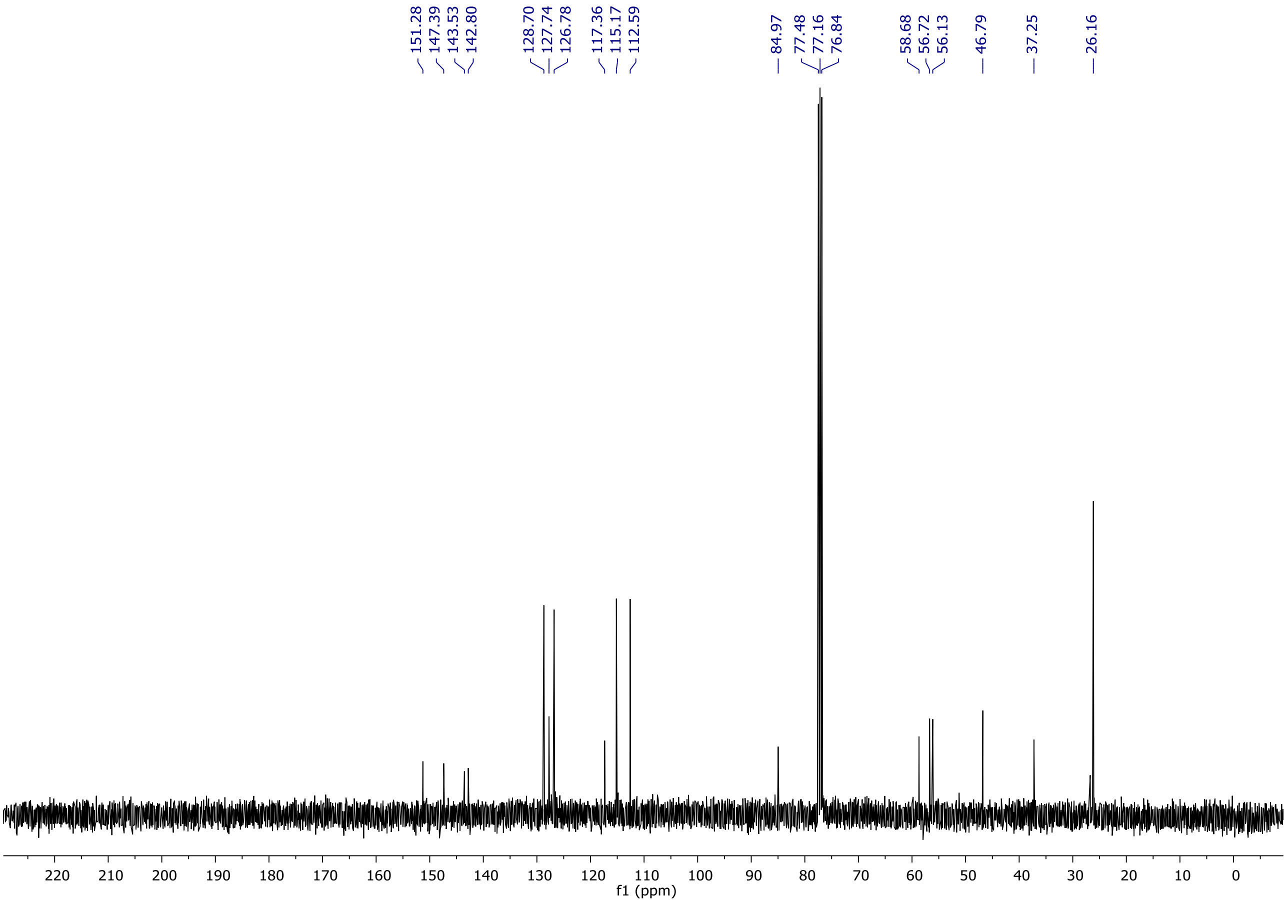

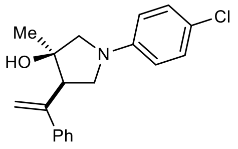
Me
HO
N
Cl
Ph

**5e**

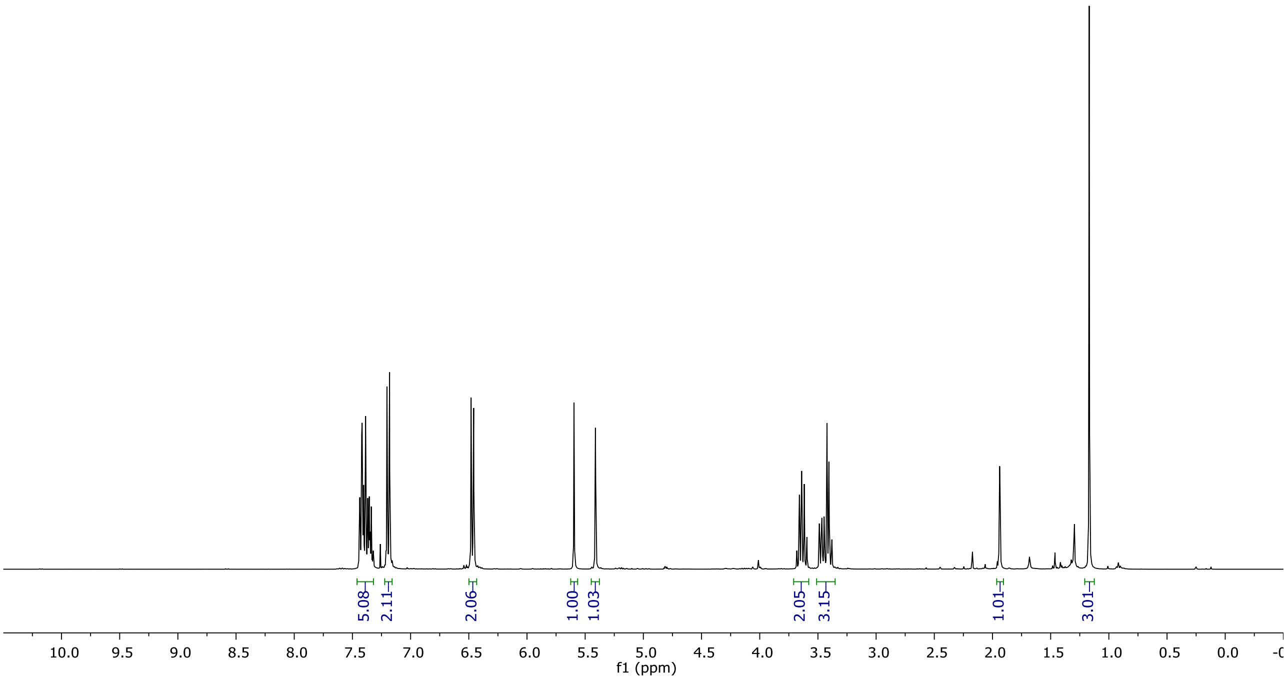
5.08
2.11
2.06
1.00
1.03
2.05
3.15
1.01
3.01
10.0 9.5 9.0 8.5 8.0 7.5 7.0 6.5 6.0 5.5 5.0 4.5 4.0 3.5 3.0 2.5 2.0 1.5 1.0 0.5 0.0
f1 (ppm)

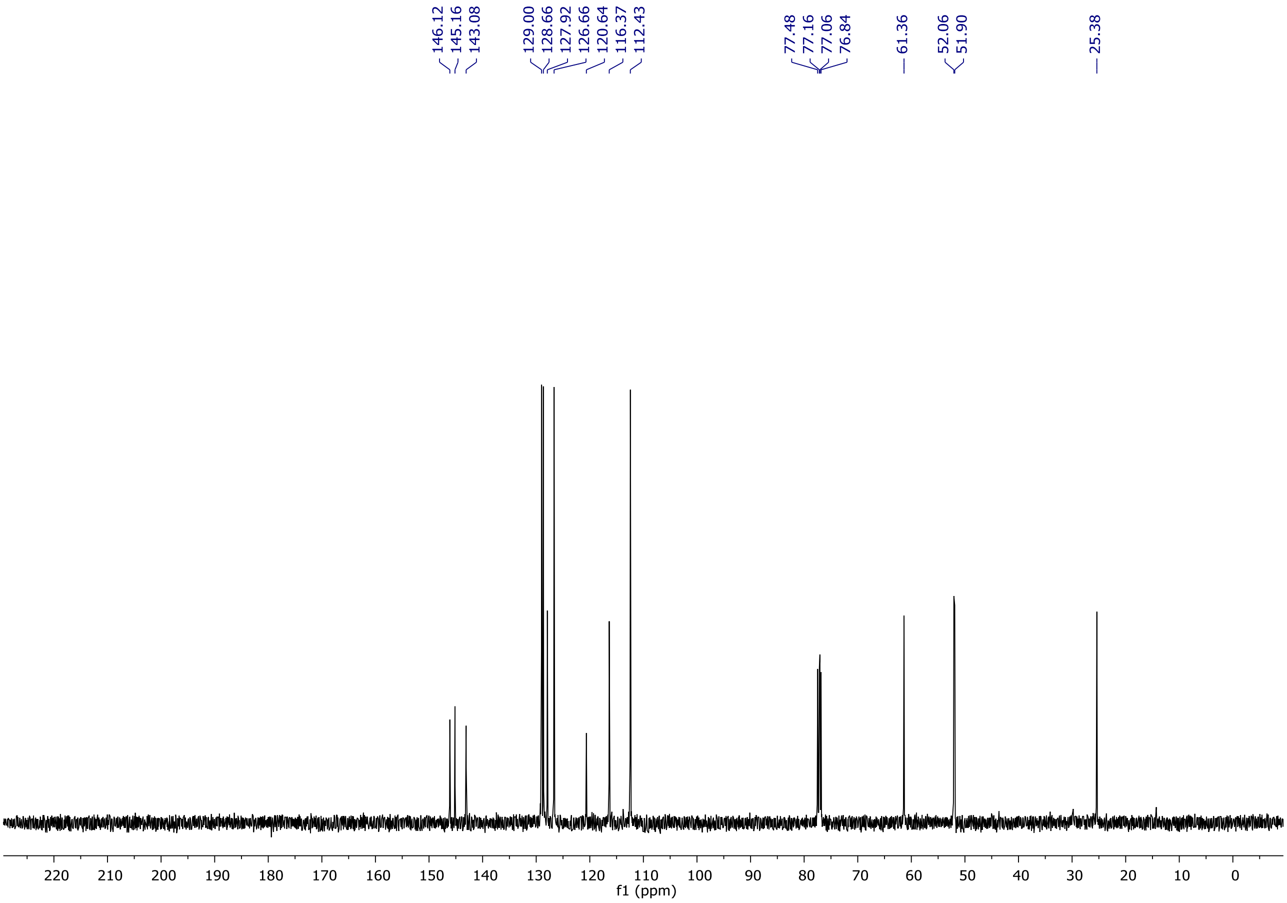
146.12
145.16
143.08
129.00
128.66
127.92
126.66
120.64
116.37
112.43
77.48
77.16
77.06
76.84
61.36
52.06
51.90
25.38
220 210 200 190 180 170 160 150 140 130 120 110 100 90 80 70 60 50 40 30 20 10 0
f1 (ppm)

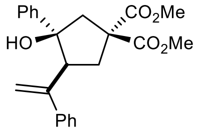
Ph
HO
CO2Me
CO2Me
Ph

**5f**

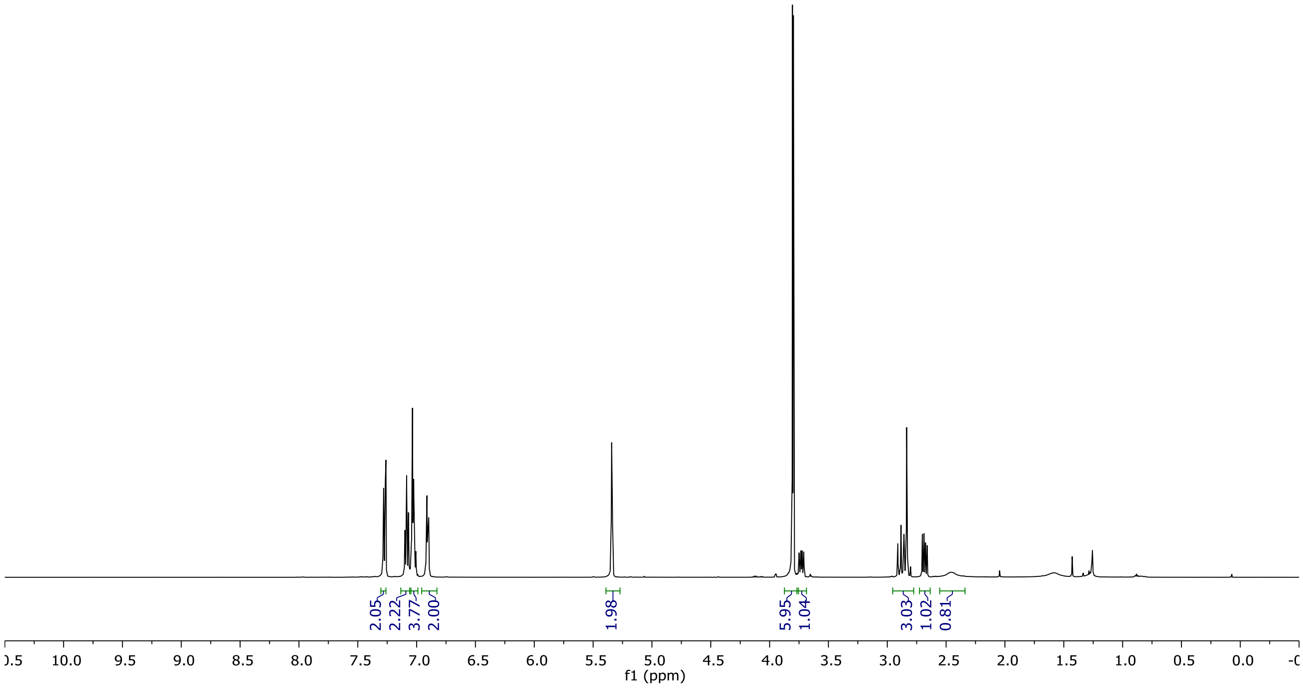
2.05
2.22
3.77
2.00
1.98
5.95
1.04
3.03
1.02
0.81
f1 (ppm)

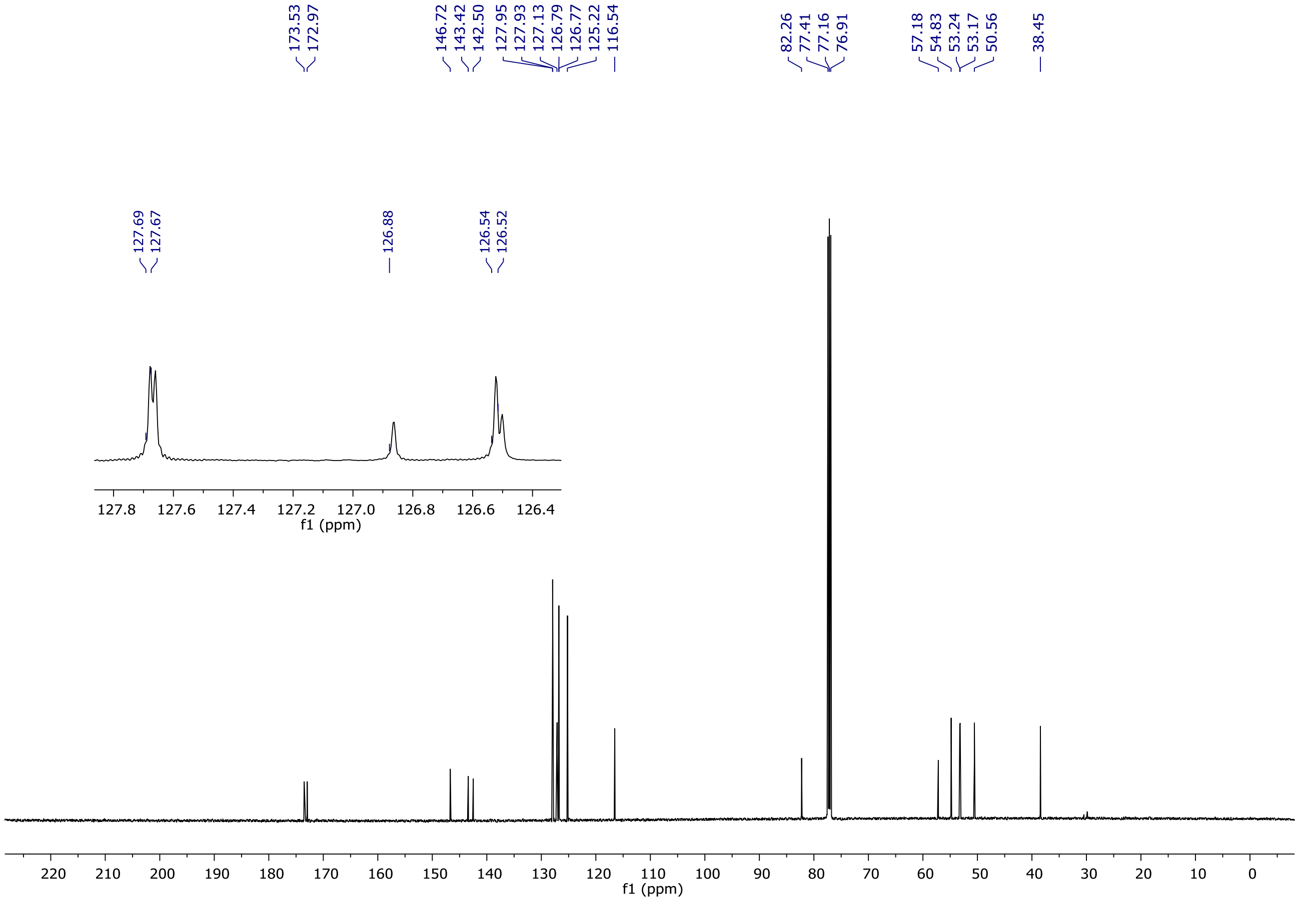
173.53
172.97
146.72
143.42
142.50
127.95
127.93
127.13
126.79
126.77
125.22
116.54
82.26
77.41
77.16
76.91
57.18
54.83
53.24
53.17
50.56
38.45
127.69
127.67
126.88
126.54
126.52
f1 (ppm)
f1 (ppm)

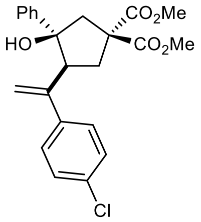
Ph
HO
$CO_2Me$
$CO_2Me$
Cl

**5g**

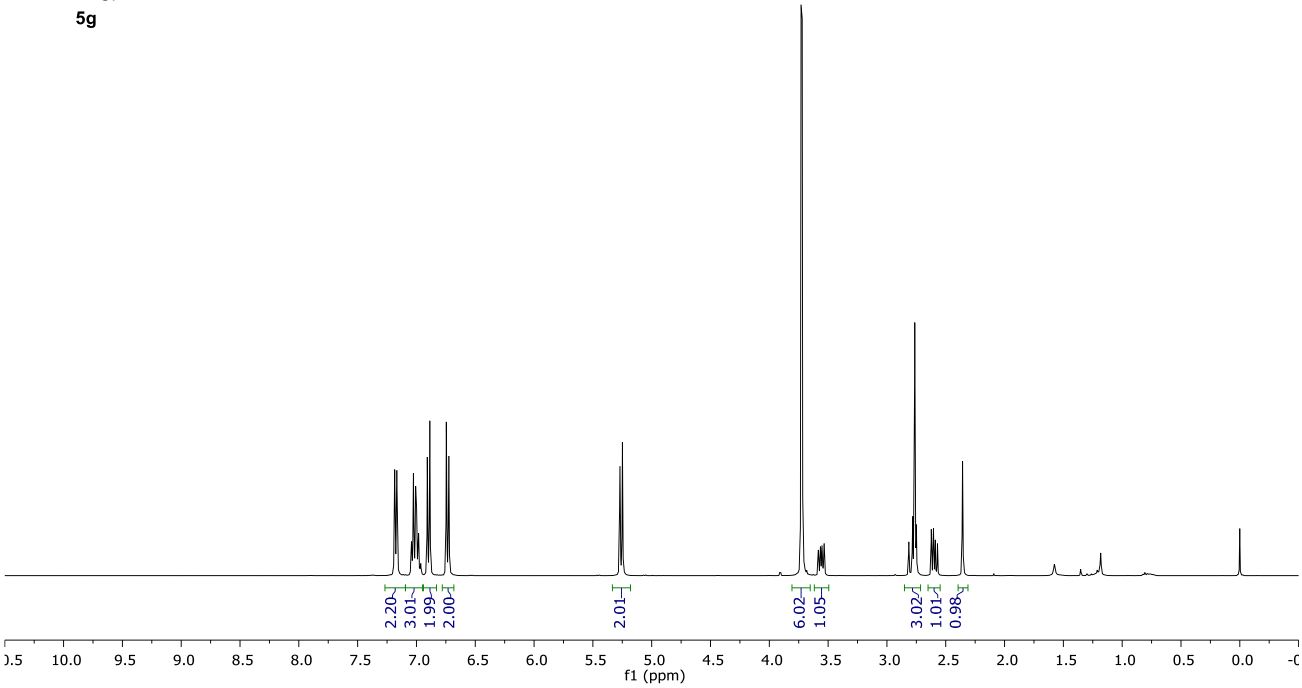
2.20
3.01
1.99
2.00
2.01
6.02
1.05
3.02
1.01
0.98
f1 (ppm)

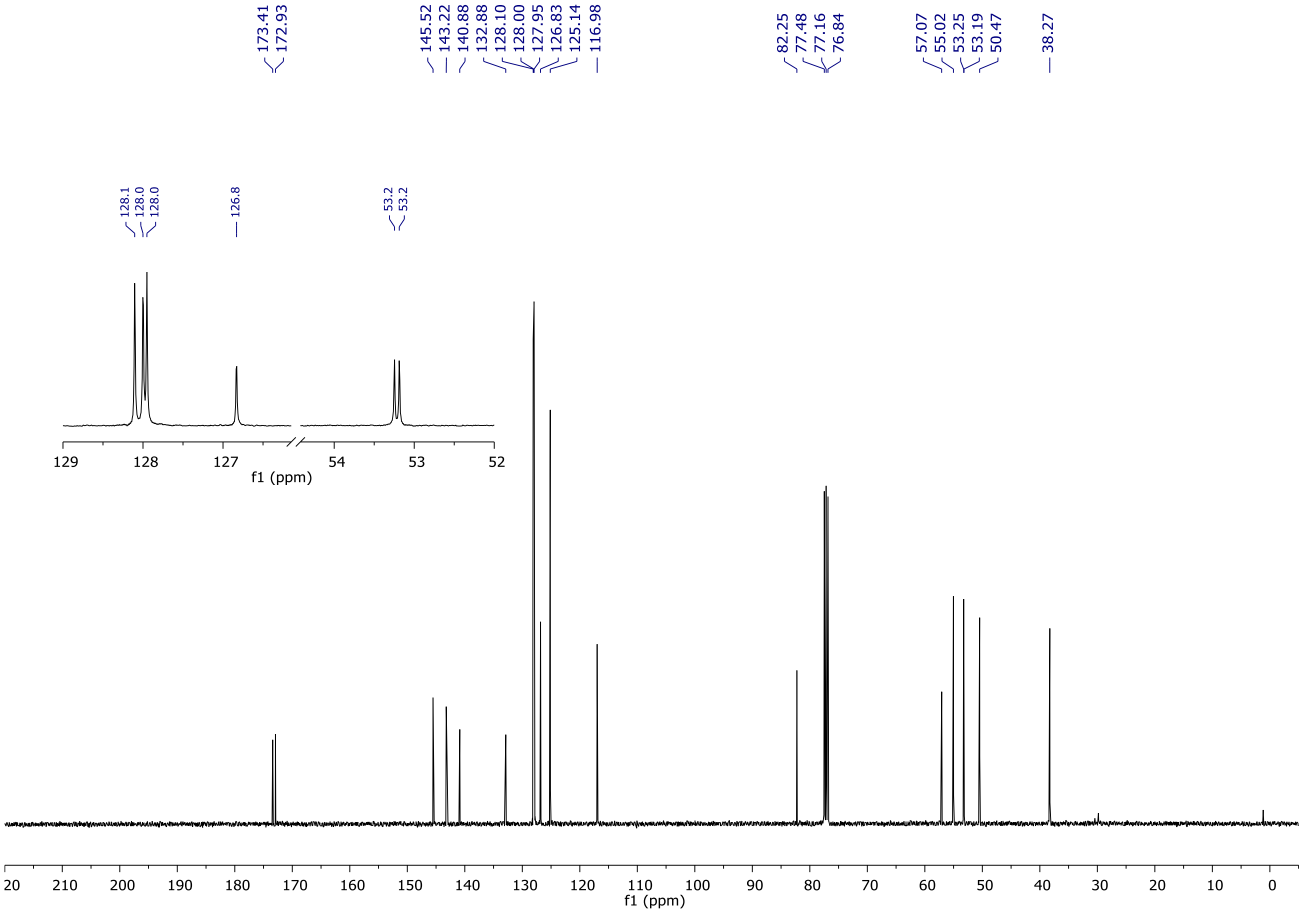
173.41
172.93
145.52
143.22
140.88
132.88
128.10
128.00
127.95
126.83
125.14
116.98
82.25
77.48
77.16
76.84
57.07
55.02
53.25
53.19
50.47
38.27
128.1
128.0
128.0
126.8
53.2
53.2
f1 (ppm)
f1 (ppm)

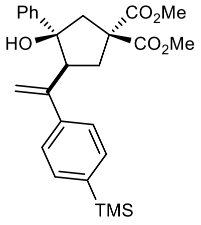
Ph
HO
$CO_2Me$
$CO_2Me$
TMS

**5h**

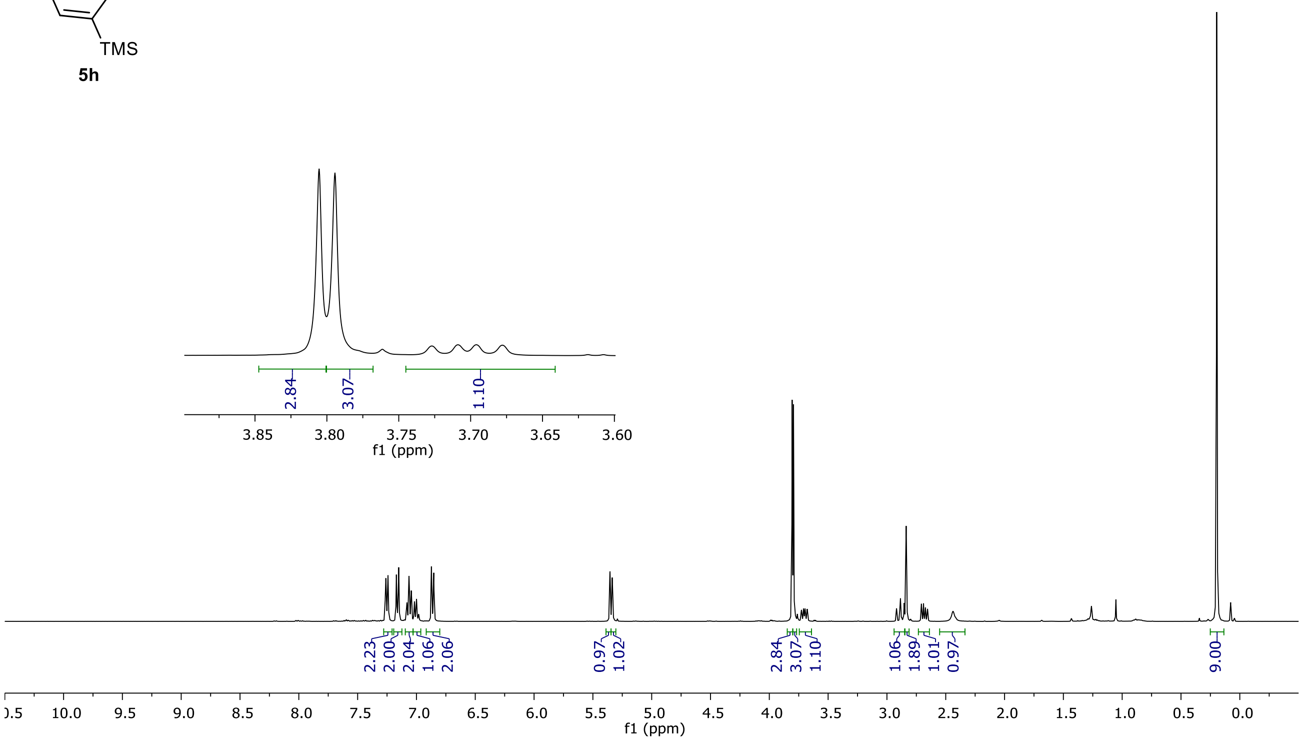
2.84 3.07 1.10
3.85 3.80 3.75 3.70 3.65 3.60
f1 (ppm)
2.23 2.00 2.04 1.06 2.06
0.97 1.02
2.84 3.07 1.10
1.06 1.89 1.01 0.97
9.00
10.0 9.5 9.0 8.5 8.0 7.5 7.0 6.5 6.0 5.5 5.0 4.5 4.0 3.5 3.0 2.5 2.0 1.5 1.0 0.5 0.0
f1 (ppm)

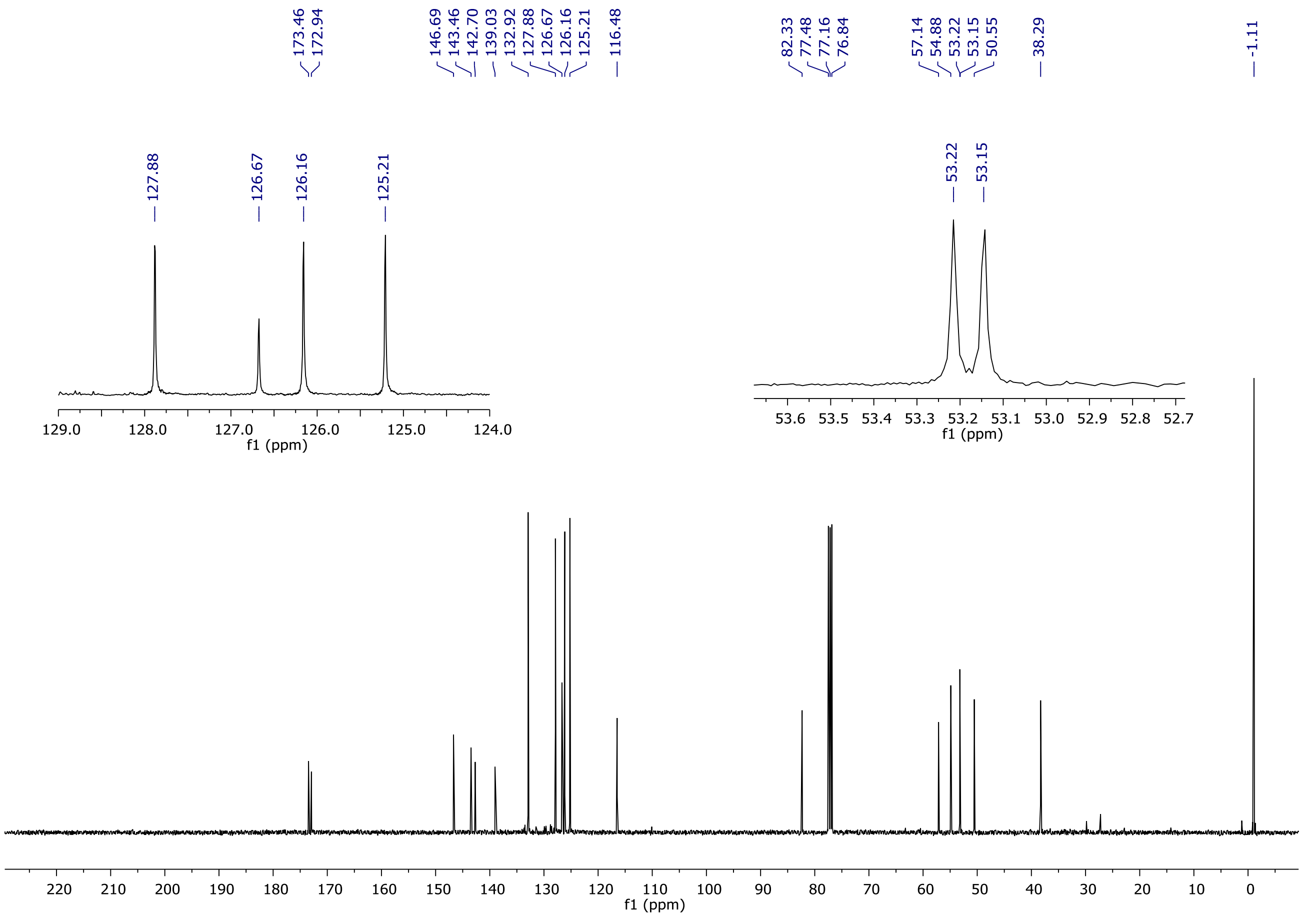
173.46 172.94
146.69 143.46 142.70 139.03 132.92 127.88 126.67 126.16 125.21
116.48
82.33 77.48 77.16 76.84
57.14 54.88 53.22 53.15 50.55
38.29
-1.11
127.88 126.67 126.16 125.21
129.0 128.0 127.0 126.0 125.0 124.0
f1 (ppm)
53.22 53.15
53.6 53.5 53.4 53.3 53.2 53.1 53.0 52.9 52.8 52.7
f1 (ppm)
220 210 200 190 180 170 160 150 140 130 120 110 100 90 80 70 60 50 40 30 20 10 0
f1 (ppm)

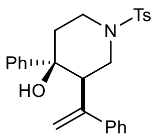
Ts
N
Ph
HO
Ph

**5i**

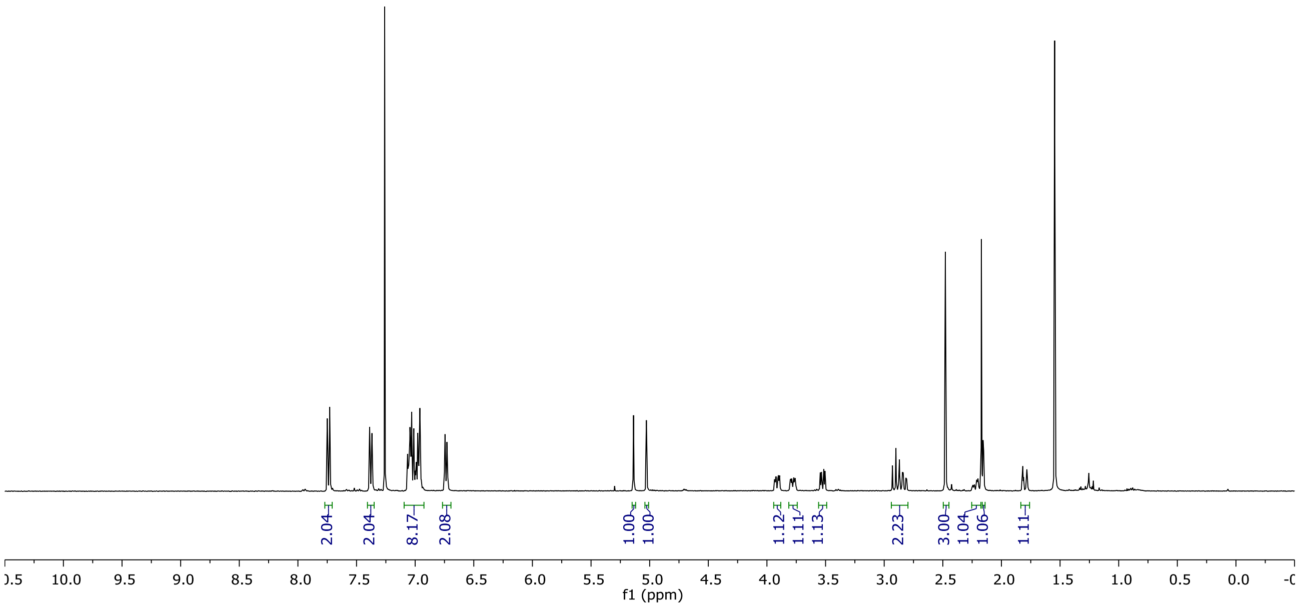
2.04
2.04
8.17
2.08
1.00
1.00
1.12
1.11
1.13
2.23
3.00
1.04
1.06
1.11
f1 (ppm)

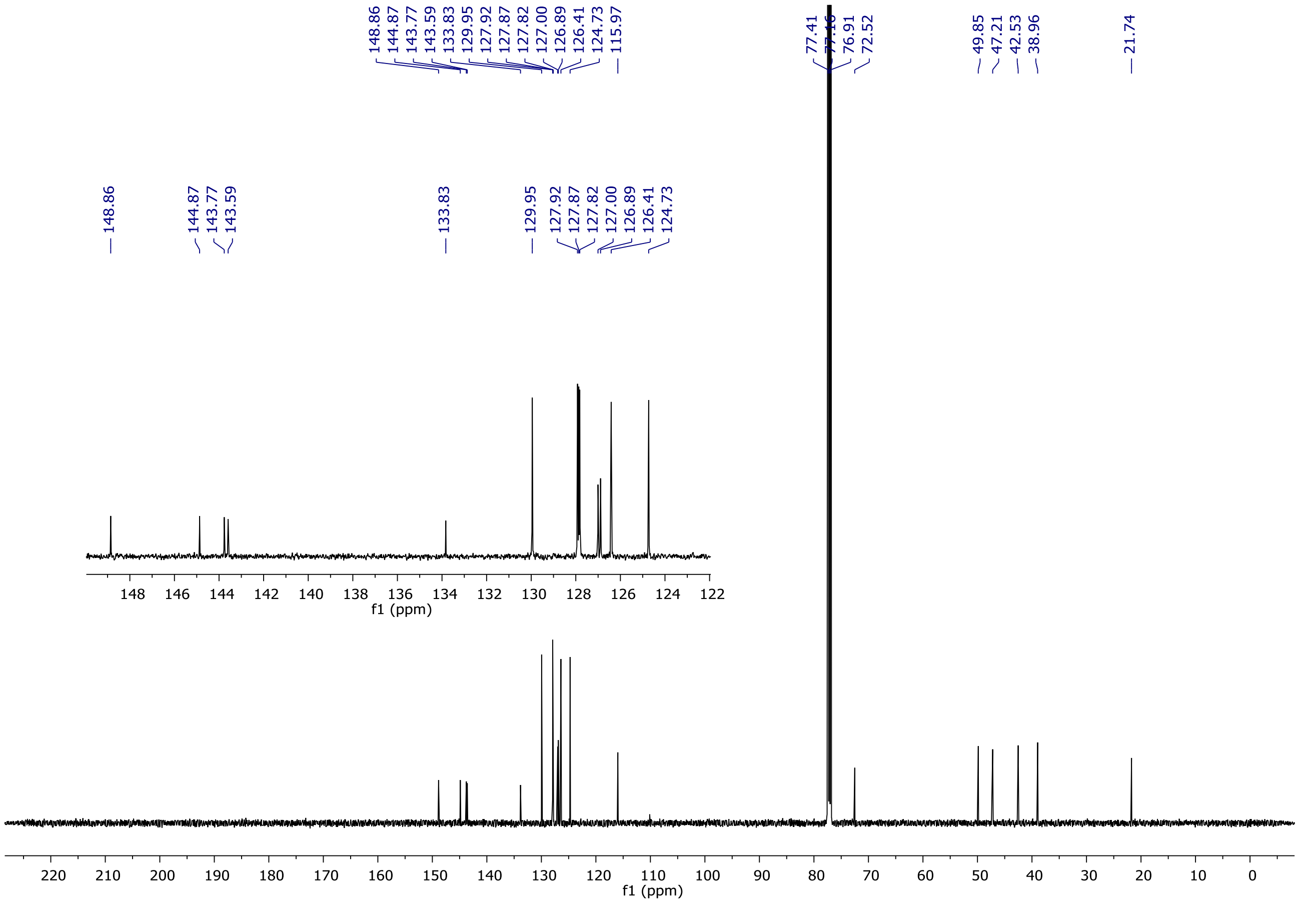
148.86
144.87
143.77
143.59
133.83
129.95
127.92
127.87
127.82
127.00
126.89
126.41
124.73
115.97
77.41
77.16
76.91
72.52
49.85
47.21
42.53
38.96
21.74
f1 (ppm)
f1 (ppm)

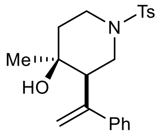
Ts
N
Me
HO
Ph

**5j**

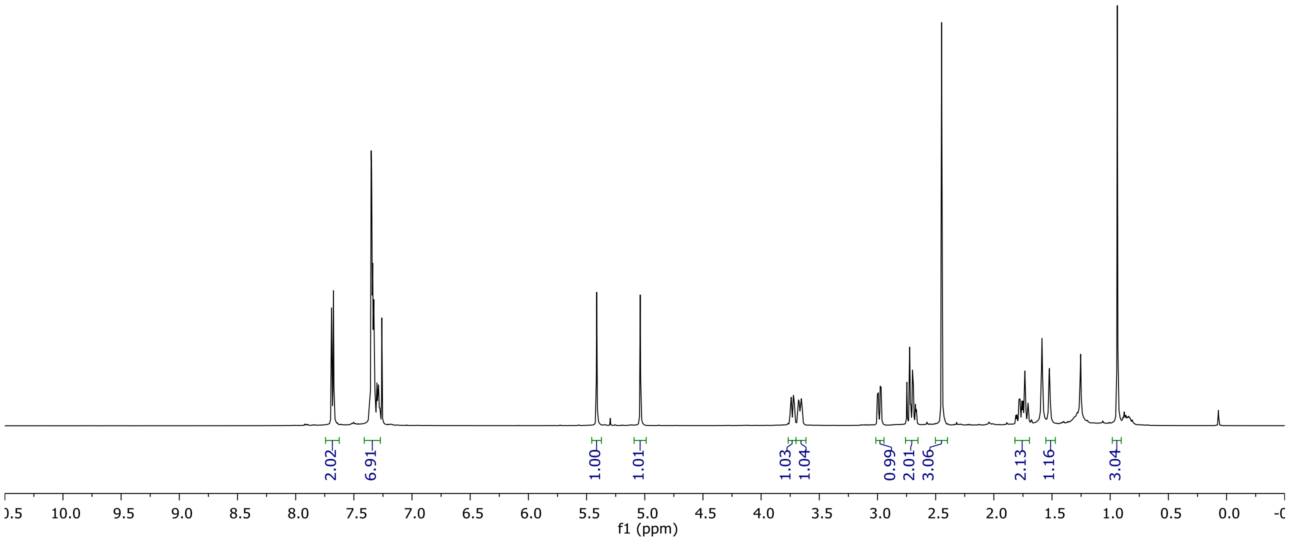
2.02
6.91
1.00
1.01
1.03
1.04
0.99
2.01
3.06
2.13
1.16
3.04
f1 (ppm)

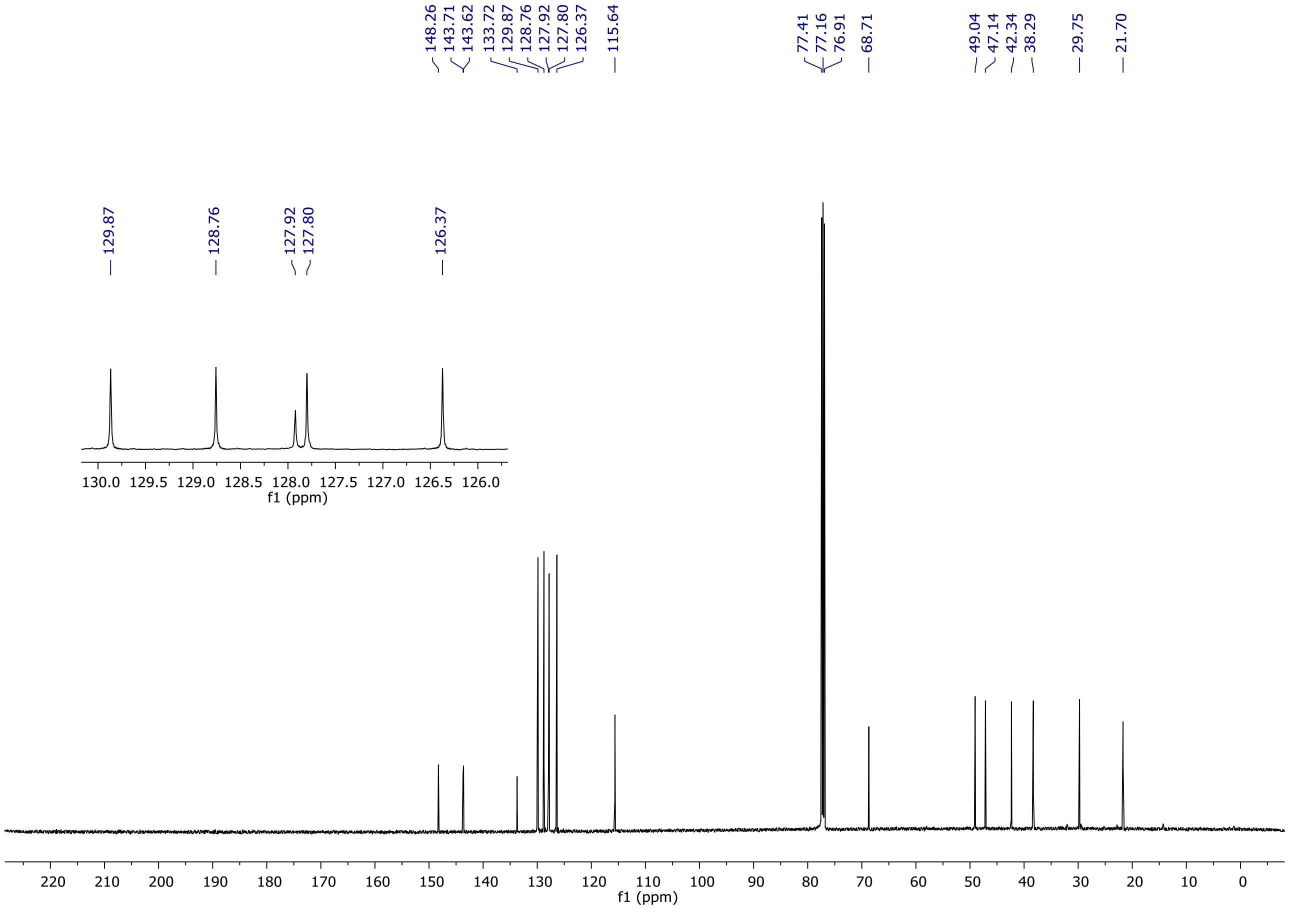
148.26
143.71
143.62
133.72
129.87
128.76
127.92
127.80
126.37
115.64
77.41
77.16
76.91
68.71
49.04
47.14
42.34
38.29
29.75
21.70
129.87
128.76
127.92
127.80
126.37
f1 (ppm)
f1 (ppm)

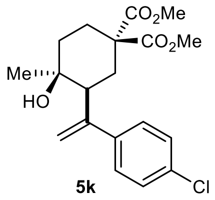
CO2Me
CO2Me
Me
HO
Cl
5k

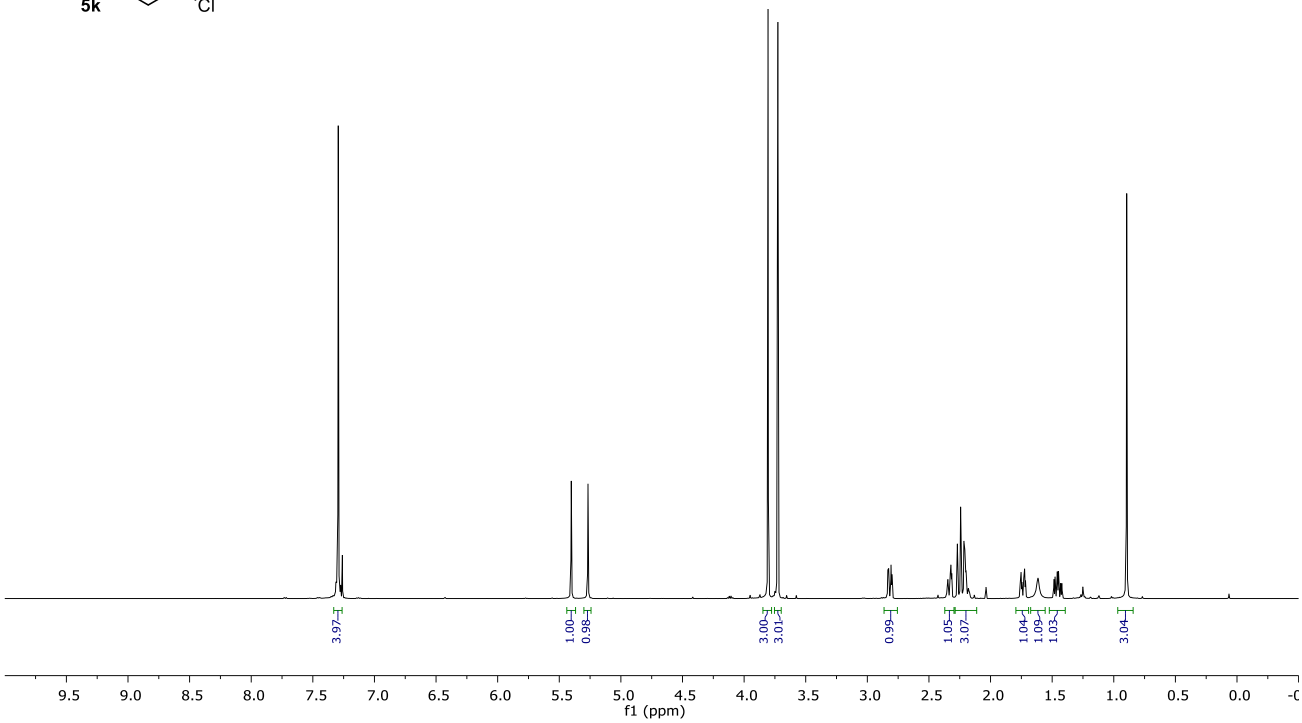
3.97
1.00
0.98
3.00
3.01
0.99
1.05
3.07
1.04
1.09
1.03
3.04
9.5 9.0 8.5 8.0 7.5 7.0 6.5 6.0 5.5 5.0 4.5 4.0 3.5 3.0 2.5 2.0 1.5 1.0 0.5 0.0
f1 (ppm)

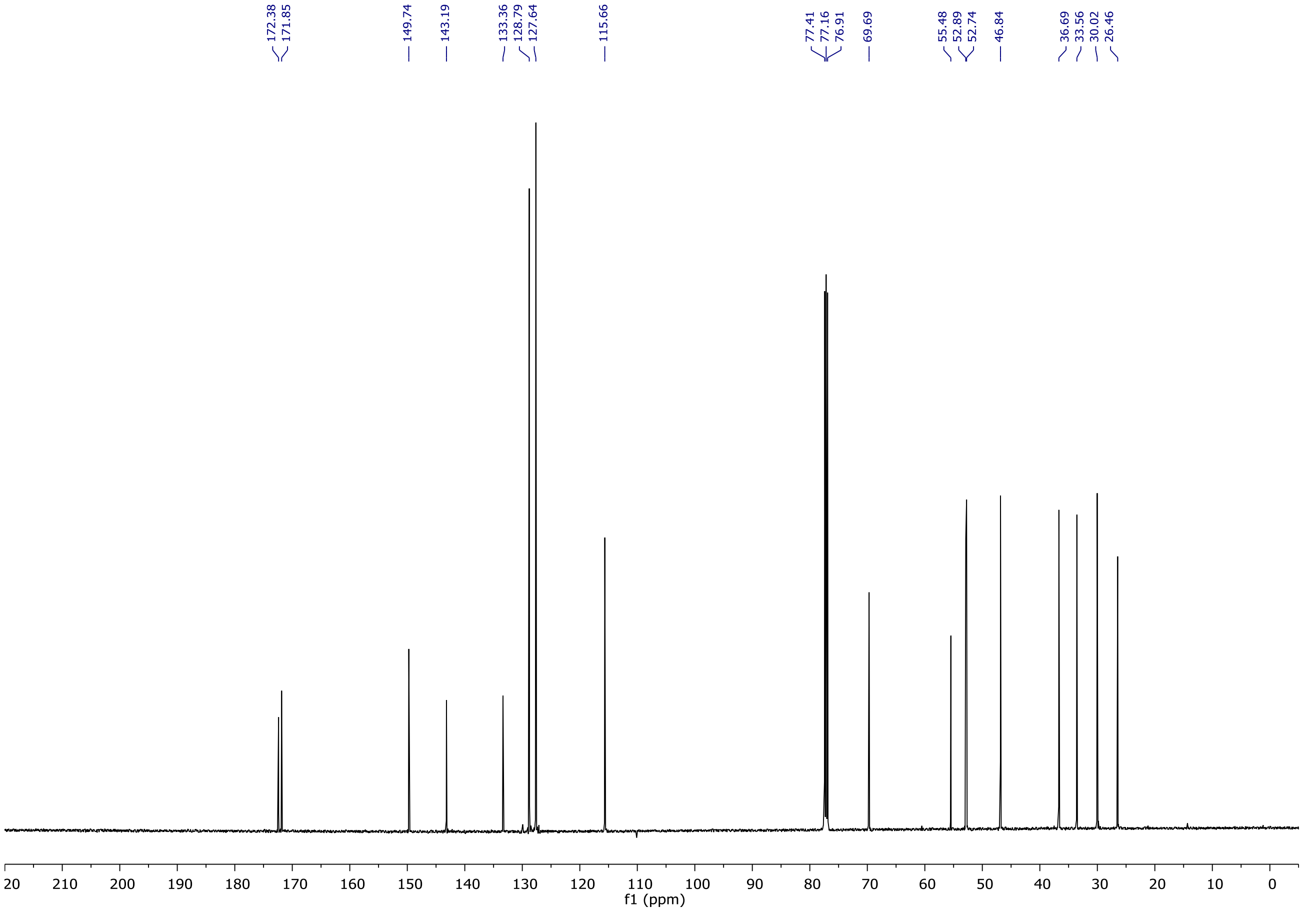
172.38
171.85
149.74
143.19
133.36
128.79
127.64
115.66
77.41
77.16
76.91
69.69
55.48
52.89
52.74
46.84
36.69
33.56
30.02
26.46
210 200 190 180 170 160 150 140 130 120 110 100 90 80 70 60 50 40 30 20 10 0
f1 (ppm)

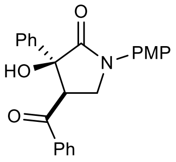
O
Ph
HO
N
PMP
O
Ph

**12**

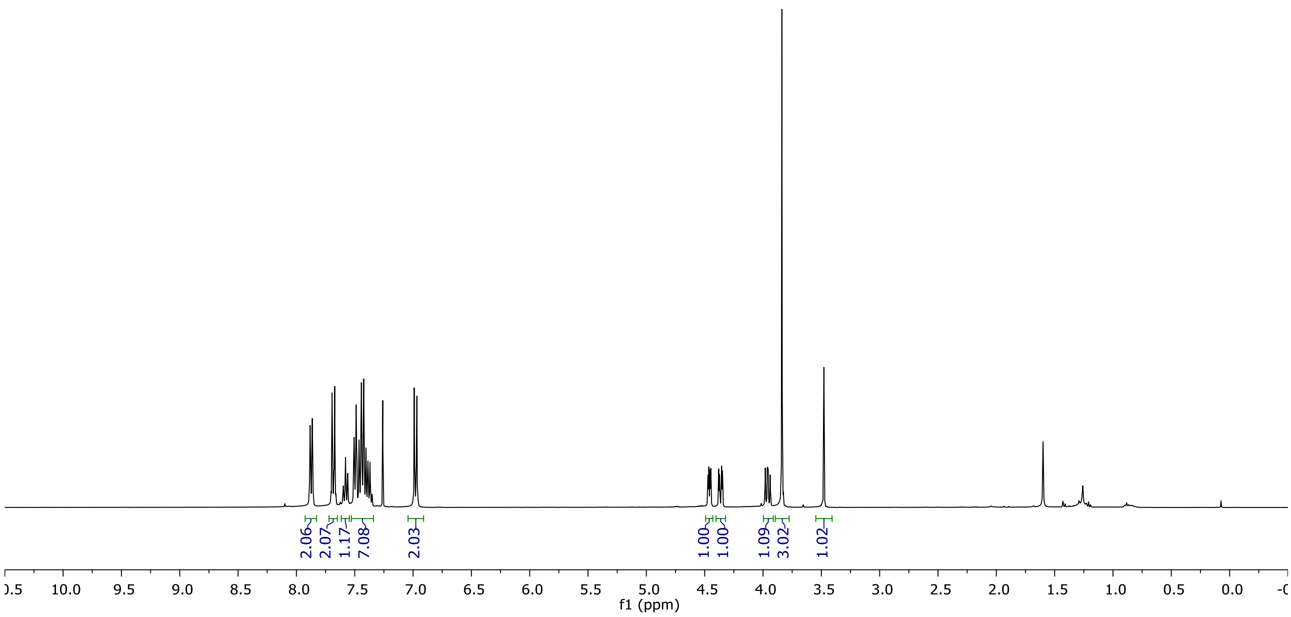
2.06
2.07
1.17
7.08
2.03
1.00
1.00
1.09
3.02
1.02
f1 (ppm)

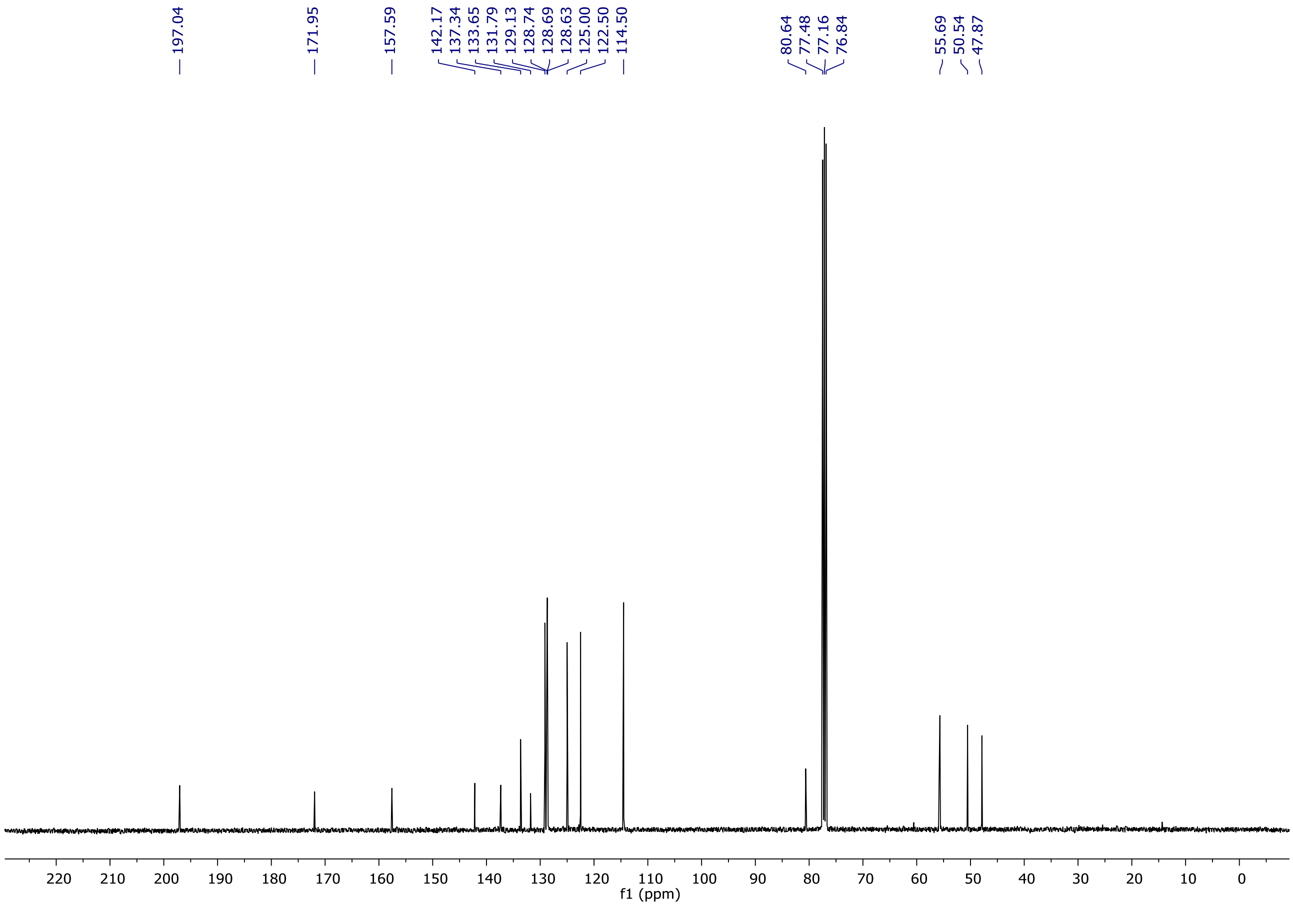
197.04
171.95
157.59
142.17
137.34
133.65
131.79
129.13
128.74
128.69
128.63
125.00
122.50
114.50
80.64
77.48
77.16
76.84
55.69
50.54
47.87
f1 (ppm)

## HPLC Traces

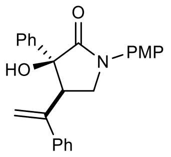

**2a**

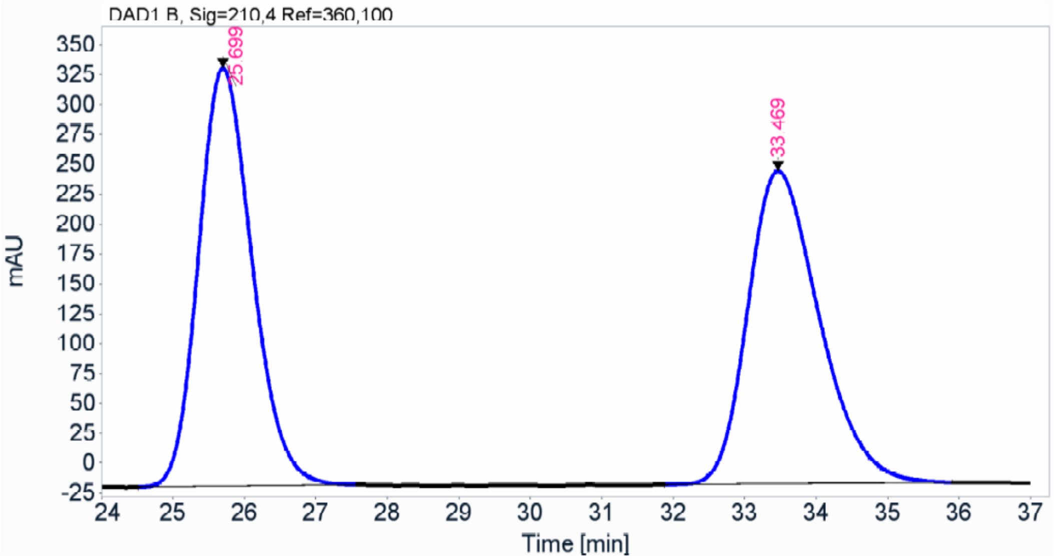


| Signal: | DAD1 B, Sig=210,4 Ref=360,100 | | | | |
|---|---|---|---|---|---|
| RT [min] | Type | Width [min] | Area | Height | Area% |
| 25.699 | BB | 0.8036 | 18213.279 | 349.7194 | 50.03 |
| 33.469 | BB | 1.0646 | 18192.375 | 261.8304 | 49.97 |

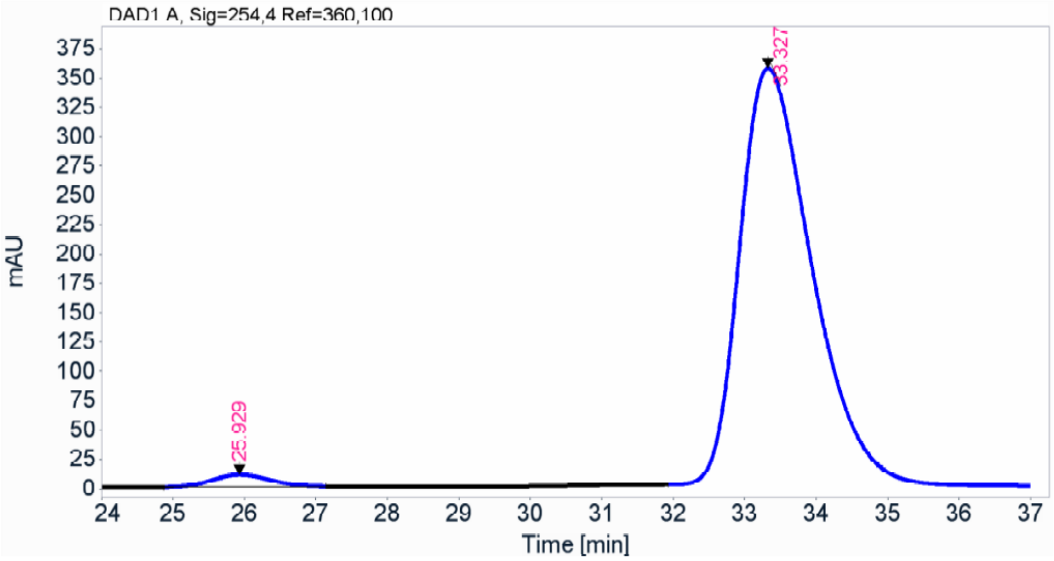


| Signal: | DAD1 A, Sig=254,4 Ref=360,100 | | | | |
|---|---|---|---|---|---|
| RT [min] | Type | Width [min] | Area | Height | Area% |
| 25.929 | MM | 0.9225 | 557.040 | 10.0644 | 2.19 |
| 33.327 | BB | 1.0700 | 24847.635 | 355.2706 | 97.81 |

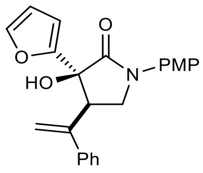


**2b**

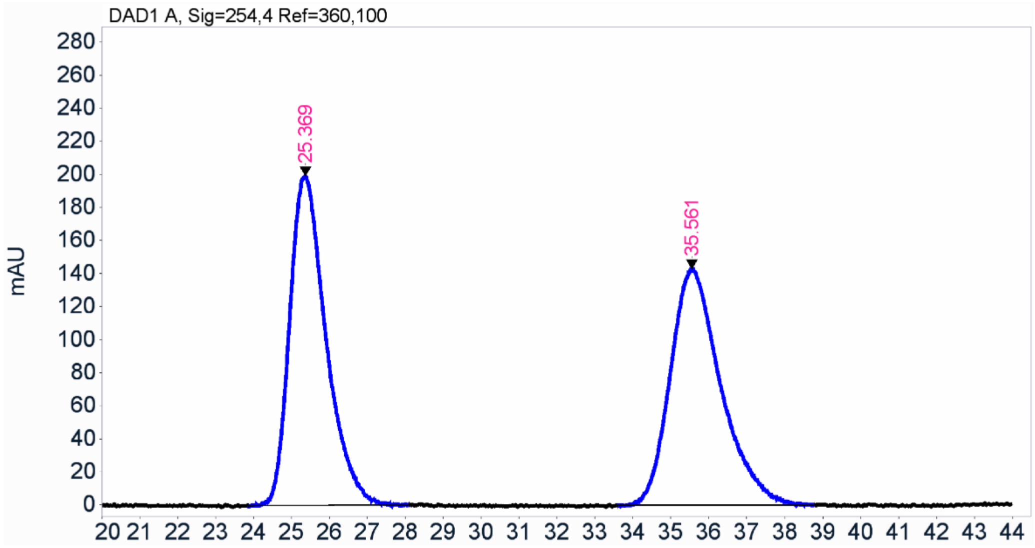


| **Signal:** | DAD1 A, Sig=254,4 Ref=360,100 | | | | |
|---|---|---|---|---|---|
| **RT [min]** | **Type** | **Width [min]** | **Area** | **Height** | **Area%** |
| 25.369 | MM | 1.1079 | 13234.310 | 199.0919 | 50.04 |
| 35.561 | MM | 1.5401 | 13212.133 | 142.9782 | 49.96 |

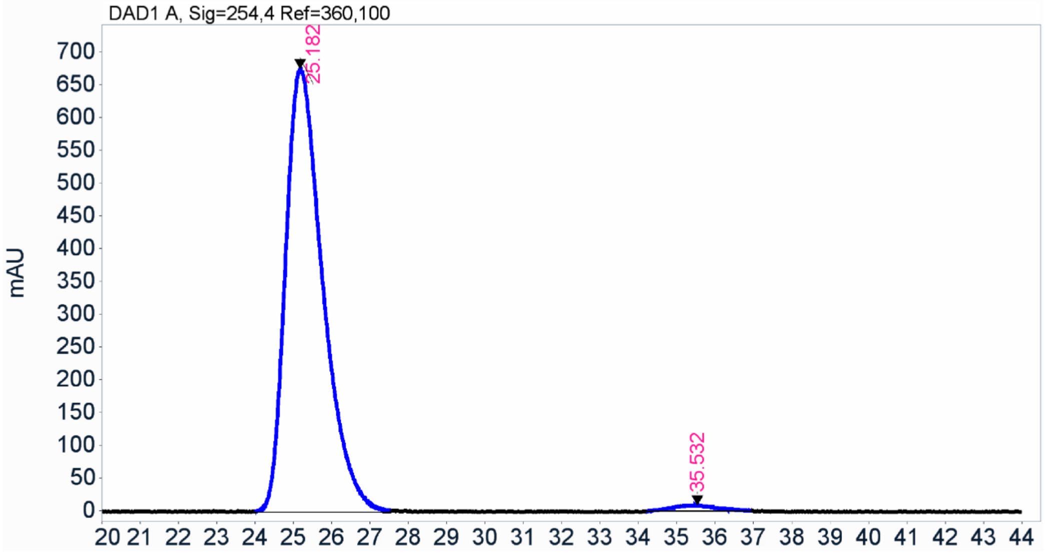


| **Signal:** | DAD1 A, Sig=254,4 Ref=360,100 | | | | |
|---|---|---|---|---|---|
| **RT [min]** | **Type** | **Width [min]** | **Area** | **Height** | **Area%** |
| 25.182 | MM | 1.1224 | 45534.895 | 676.1537 | 98.46 |
| 35.532 | MM | 1.3408 | 712.000 | 8.8502 | 1.54 |

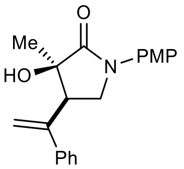


**2c**

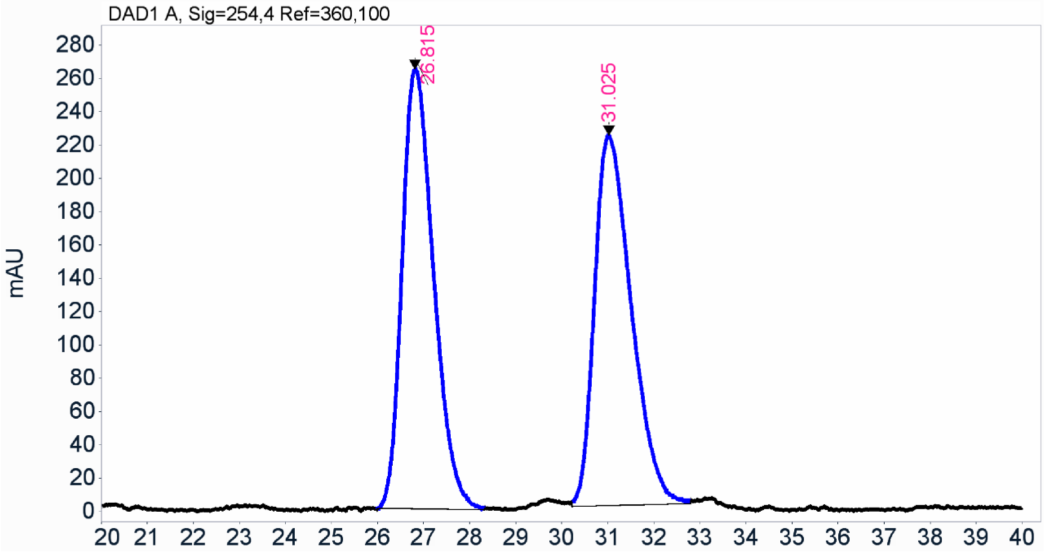


| Signal: | DAD1 A, Sig=254,4 Ref=360,100 | | | | |
|---|---|---|---|---|---|
| **RT [min]** | **Type** | **Width [min]** | **Area** | **Height** | **Area%** |
| 26.815 | MM | 0.7933 | 12564.892 | 263.9943 | 50.44 |
| 31.025 | MM | 0.9252 | 12344.252 | 222.3730 | 49.56 |

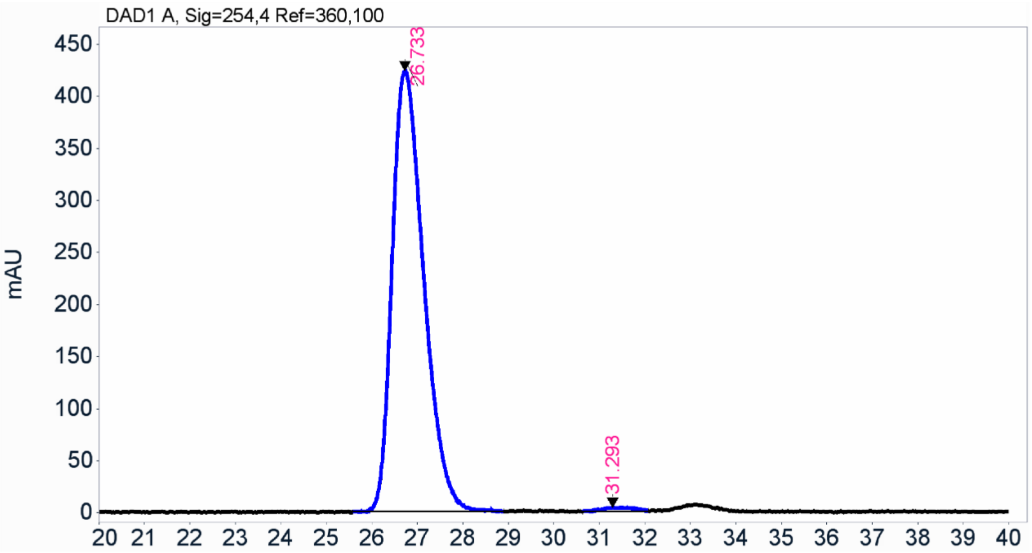


| Signal: | DAD1 A, Sig=254,4 Ref=360,100 | | | | |
|---|---|---|---|---|---|
| **RT [min]** | **Type** | **Width [min]** | **Area** | **Height** | **Area%** |
| 26.733 | MM | 0.8037 | 20415.271 | 423.3376 | 99.03 |
| 31.293 | MM | 0.8312 | 199.672 | 4.0035 | 0.97 |

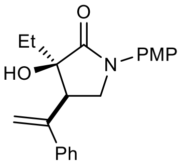


**2d**

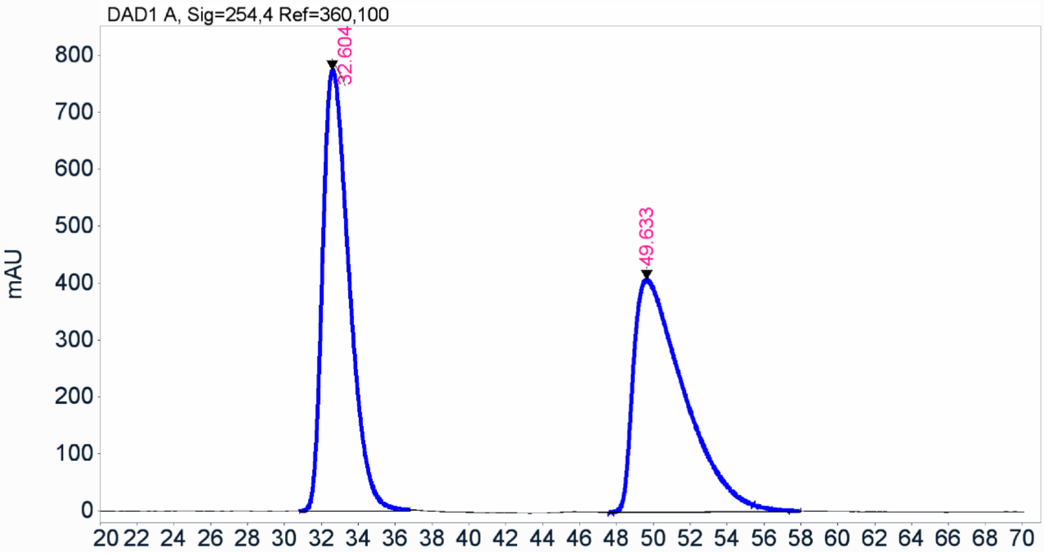


**Signal:** DAD1 A, Sig=254,4 Ref=360,100

| RT [min] | Type | Width [min] | Area | Height | Area% |
|---|---|---|---|---|---|
| 32.604 | MM | 1.6617 | 77293.281 | 775.2402 | 49.81 |
| 49.633 | MM | 3.1803 | 77878.516 | 408.1319 | 50.19 |

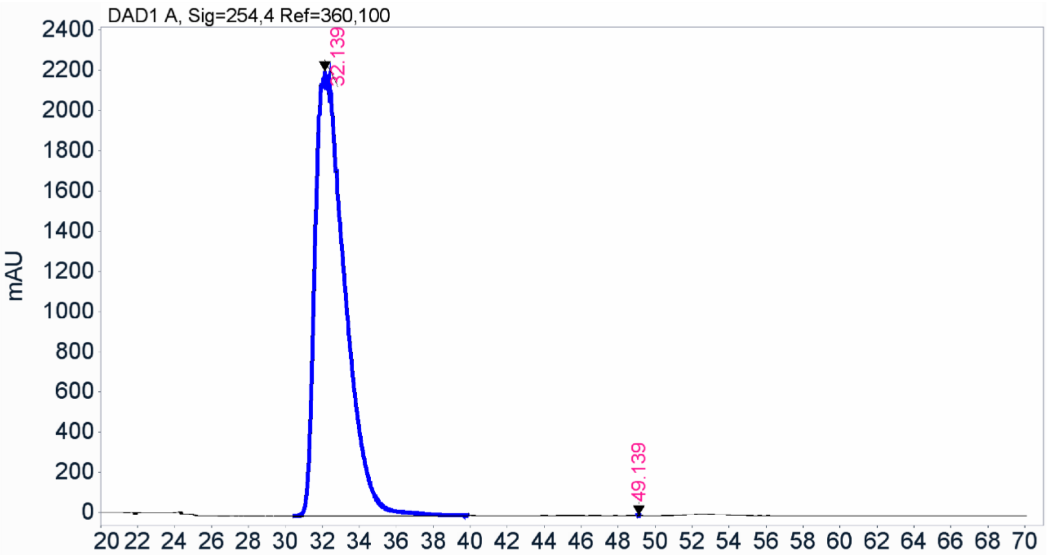


**Signal:** DAD1 A, Sig=254,4 Ref=360,100

| RT [min] | Type | Width [min] | Area | Height | Area% |
|---|---|---|---|---|---|
| 32.139 | MM | 1.8484 | 245207.156 | 2210.9839 | 100.00 |
| 49.139 | MM | 0.1087 | 10.474 | 1.6057 | 0.00 |

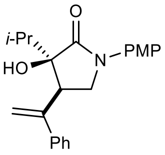


**2e**

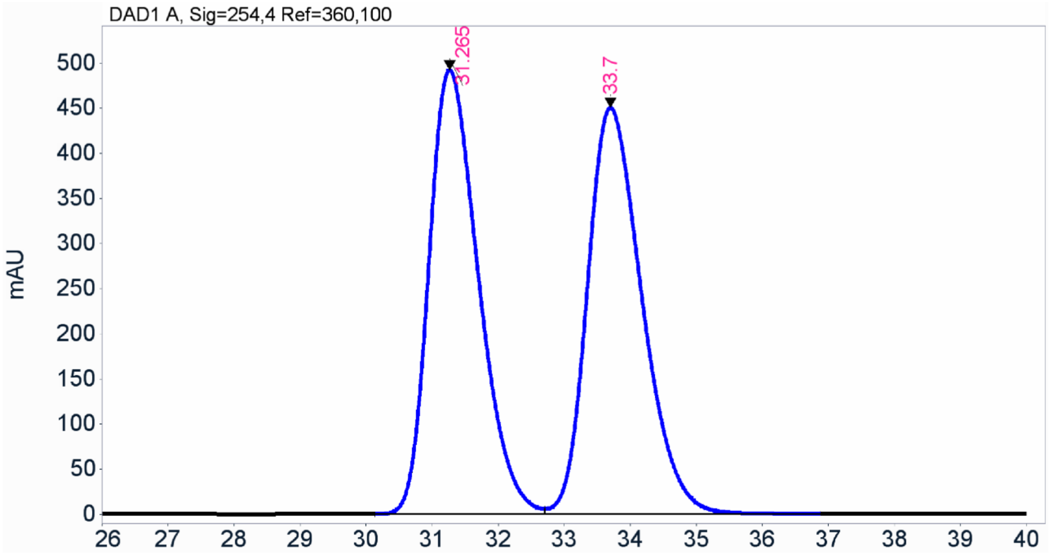


| Signal: | DAD1 A, Sig=254,4 Ref=360,100 | | | | |
|---|---|---|---|---|---|
| RT [min] | Type | Width [min] | Area | Height | Area% |
| 31.265 | BV | 0.8073 | 25531.139 | 492.1060 | 49.81 |
| 33.700 | VB | 0.8844 | 25727.092 | 450.4184 | 50.19 |

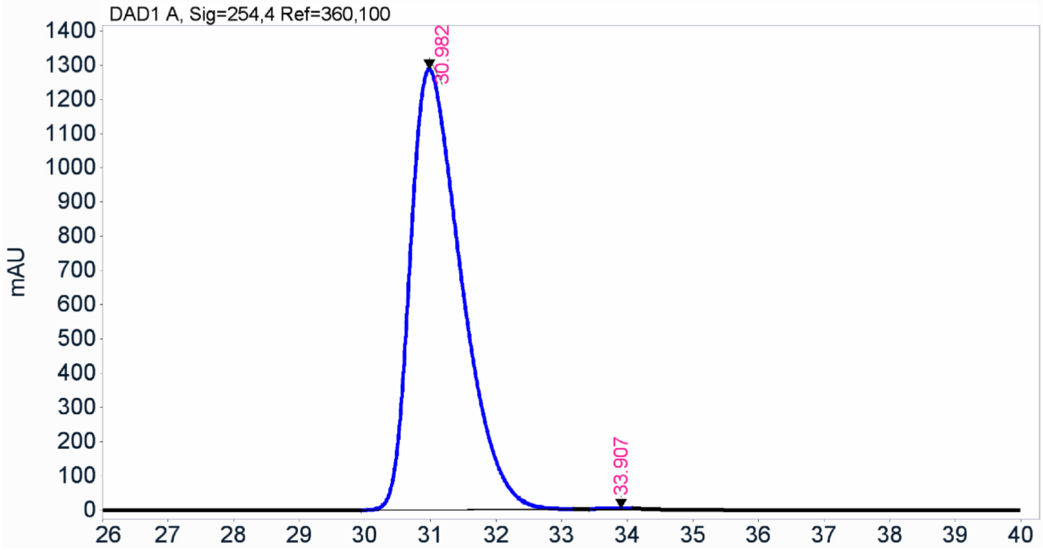


| Signal: | DAD1 A, Sig=254,4 Ref=360,100 | | | | |
|---|---|---|---|---|---|
| RT [min] | Type | Width [min] | Area | Height | Area% |
| 30.982 | BB | 0.8392 | 69180.984 | 1286.7573 | 99.86 |
| 33.907 | MM | 0.5544 | 95.963 | 2.8850 | 0.14 |

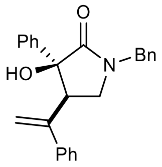


**2f**

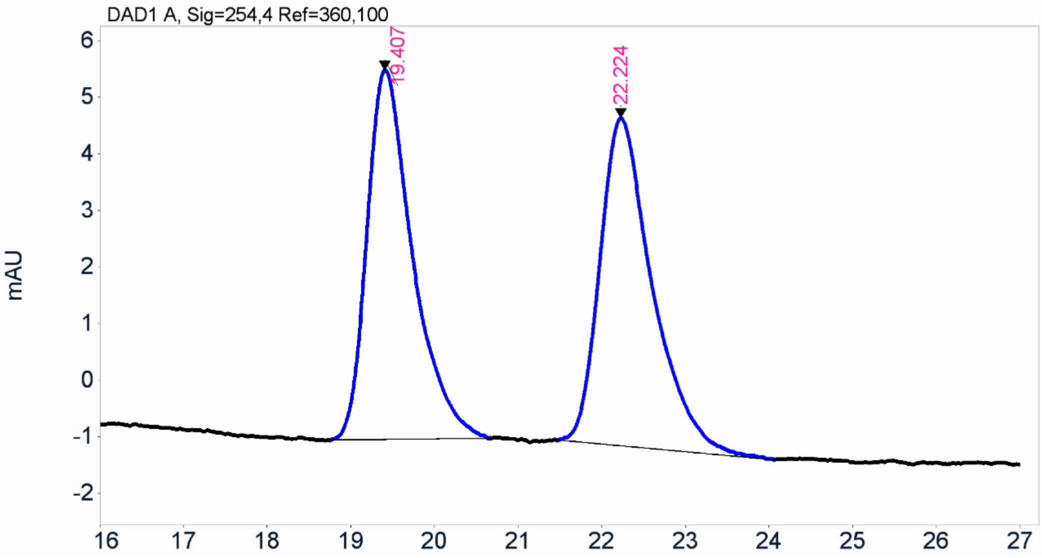


| **Signal:** | DAD1 A, Sig=254,4 Ref=360,100 | | | | |
|---|---|---|---|---|---|
| **RT [min]** | **Type** | **Width [min]** | **Area** | **Height** | **Area%** |
| 19.407 | BB | 0.5537 | 247.088 | 6.5257 | 49.47 |
| 22.224 | BB | 0.6222 | 252.377 | 5.7940 | 50.53 |

Reaction carried out in TFE:

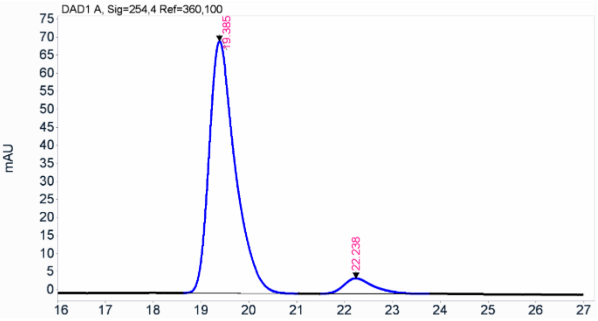


| **Signal:** | DAD1 A, Sig=254,4 Ref=360,100 | | | | |
|---|---|---|---|---|---|
| **RT [min]** | **Type** | **Width [min]** | **Area** | **Height** | **Area%** |
| 19.385 | BB | 0.5612 | 2642.818 | 69.8879 | 93.47 |
| 22.238 | BB | 0.5829 | 184.562 | 4.2647 | 6.53 |

Reaction as carried out in MeCN:

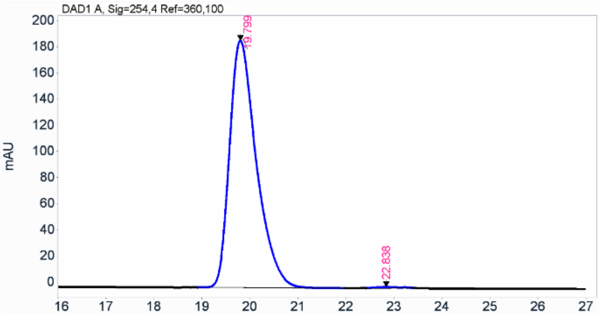


| **Signal:** | DAD1 A, Sig=254,4 Ref=360,100 | | | | |
|---|---|---|---|---|---|
| **RT [min]** | **Type** | **Width [min]** | **Area** | **Height** | **Area%** |
| 19.799 | BB | 0.5828 | 7301.608 | 188.9537 | 99.51 |
| 22.838 | MM | 0.4612 | 36.165 | 0.9340 | 0.49 |

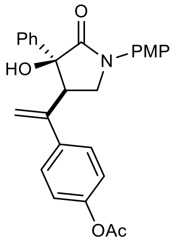


**2g**

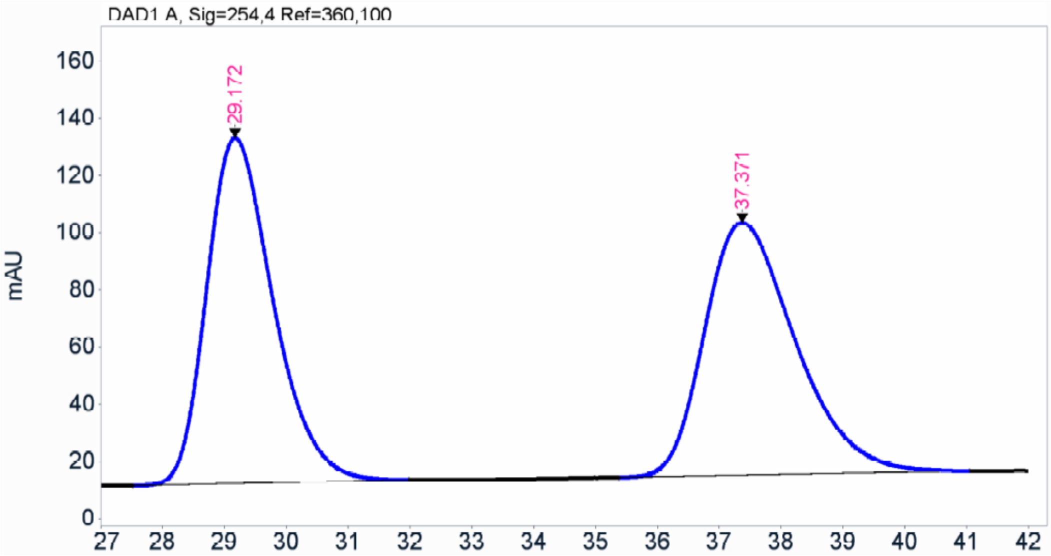


**Signal:** DAD1 A, Sig=254,4 Ref=360,100

| RT [min] | Type | Width [min] | Area | Height | Area% |
|---|---|---|---|---|---|
| 29.172 | BB | 1.1454 | 9026.544 | 120.8490 | 49.96 |
| 37.371 | BB | 1.5009 | 9041.647 | 88.2804 | 50.04 |

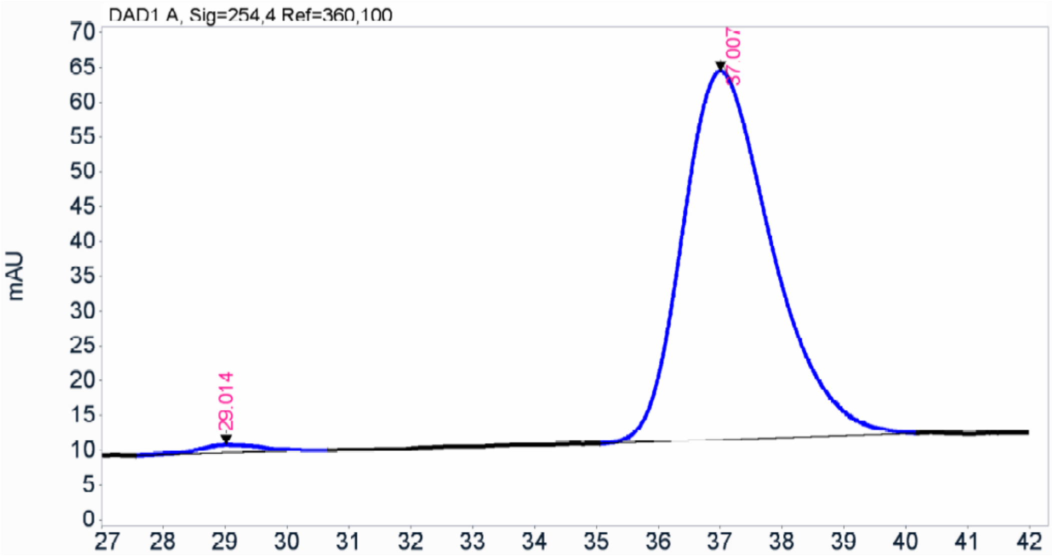


**Signal:** DAD1 A, Sig=254,4 Ref=360,100

| RT [min] | Type | Width [min] | Area | Height | Area% |
|---|---|---|---|---|---|
| 29.014 | MM | 1.2323 | 87.398 | 1.1820 | 1.63 |
| 37.007 | BB | 1.4234 | 5289.287 | 52.9490 | 98.37 |

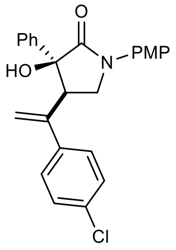


**2h**

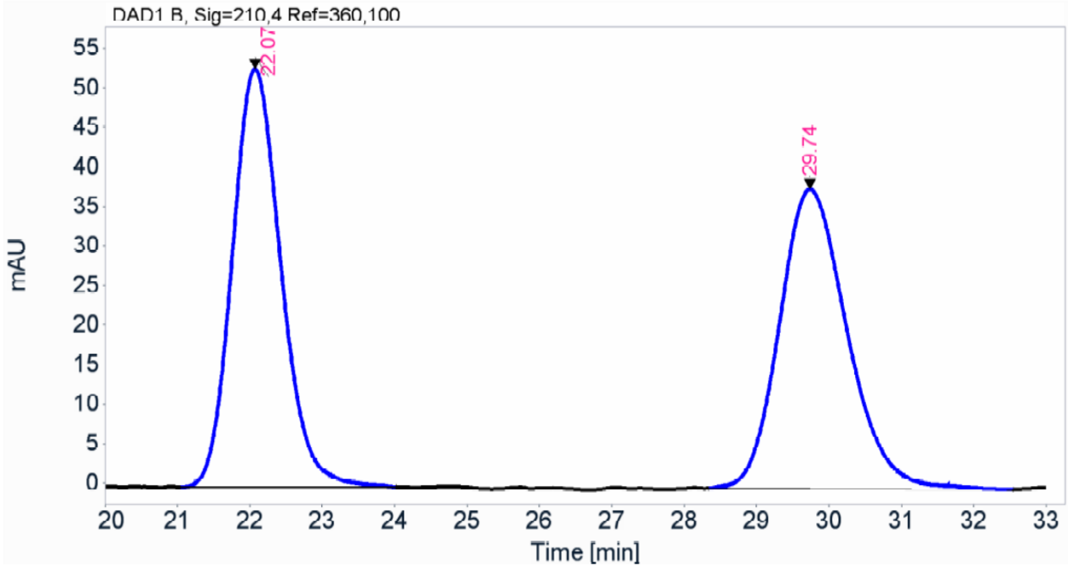


| Signal: | DAD1 B, Sig=210,4 Ref=360,100 | | | | |
|---|---|---|---|---|---|
| RT [min] | Type | Width [min] | Area | Height | Area% |
| 22.070 | BB | 0.7230 | 2471.330 | 52.9043 | 50.09 |
| 29.740 | BB | 0.9536 | 2462.733 | 37.9243 | 49.91 |

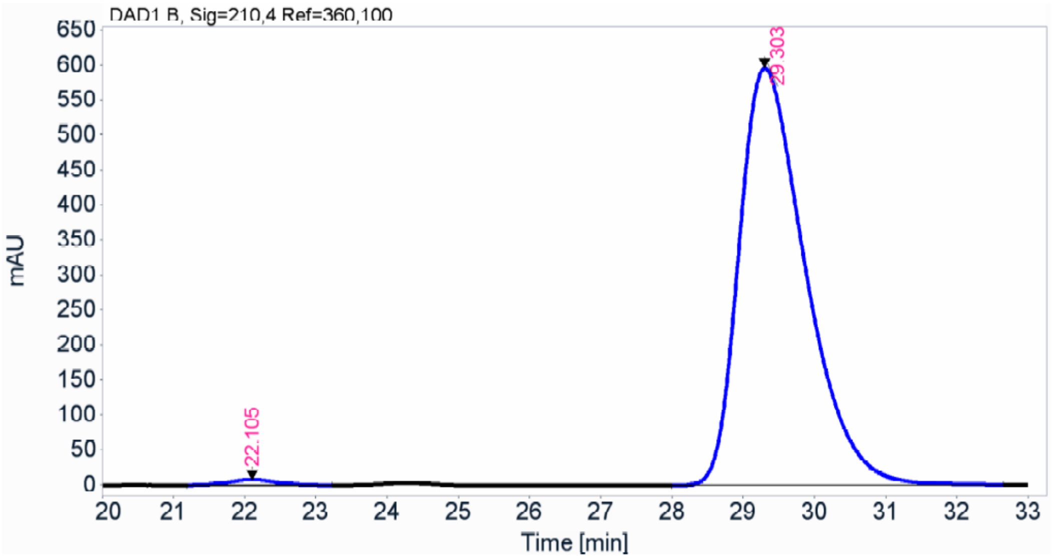


| Signal: | DAD1 B, Sig=210,4 Ref=360,100 | | | | |
|---|---|---|---|---|---|
| RT [min] | Type | Width [min] | Area | Height | Area% |
| 22.105 | BB | 0.5936 | 370.359 | 8.0981 | 0.95 |
| 29.303 | BB | 0.9912 | 38502.746 | 596.3964 | 99.05 |

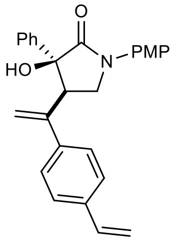


**2i**

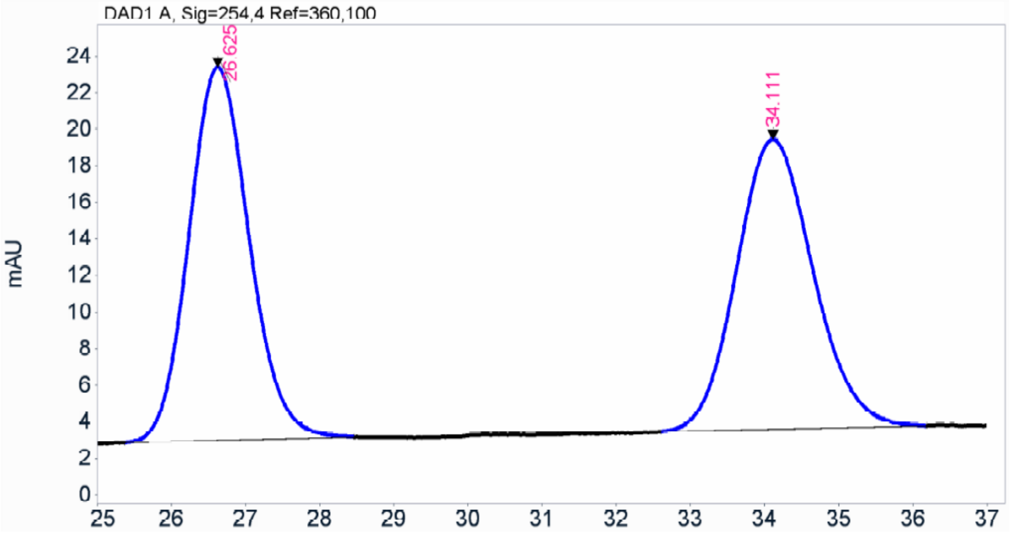


| Signal: | DAD1 A, Sig=254,4 Ref=360,100 | | | | |
|---|---|---|---|---|---|
| RT [min] | Type | Width [min] | Area | Height | Area% |
| 26.625 | BB | 0.8565 | 1151.465 | 20.4002 | 50.29 |
| 34.111 | BB | 0.9306 | 1138.096 | 15.8342 | 49.71 |

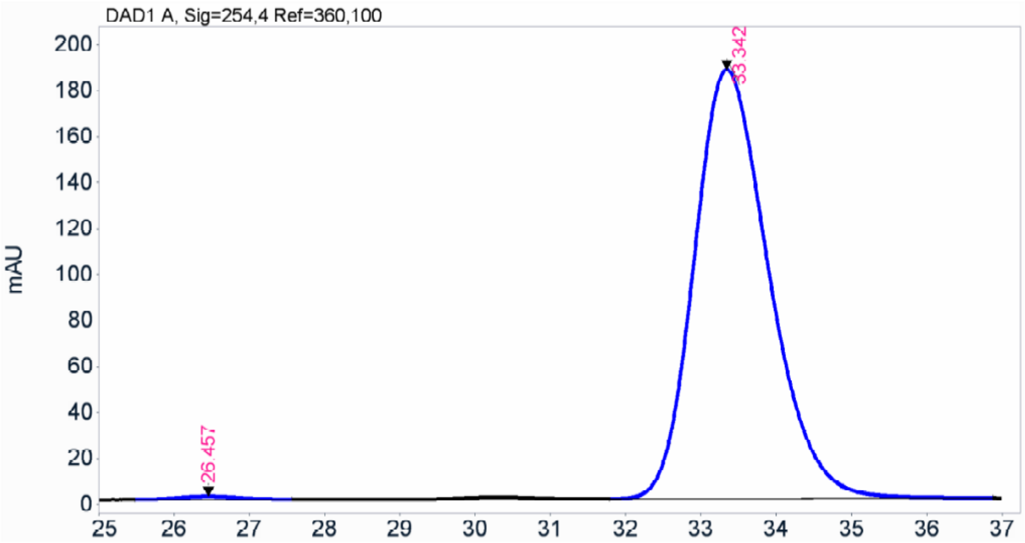


| Signal: | DAD1 A, Sig=254,4 Ref=360,100 | | | | |
|---|---|---|---|---|---|
| RT [min] | Type | Width [min] | Area | Height | Area% |
| 26.457 | MM | 0.9859 | 92.070 | 1.5564 | 0.71 |
| 33.342 | BB | 1.0721 | 12830.535 | 186.6275 | 99.29 |

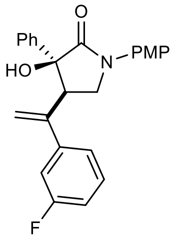


**2j**

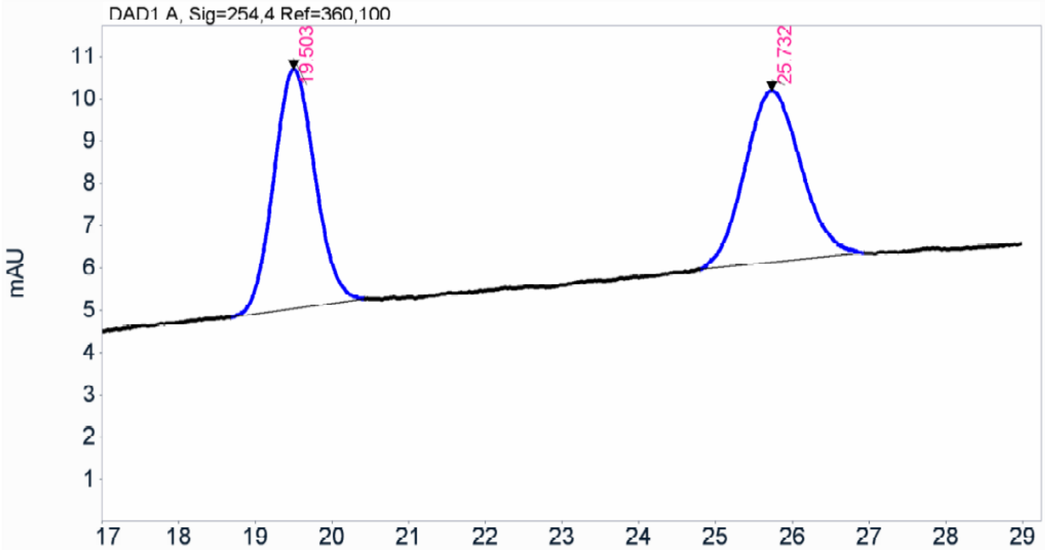


| **Signal:** | DAD1 A, Sig=254,4 Ref=360,100 | | | | |
|---|---|---|---|---|---|
| **RT [min]** | **Type** | **Width [min]** | **Area** | **Height** | **Area%** |
| 19.503 | BB | 0.5129 | 211.861 | 5.6577 | 50.58 |
| 25.732 | BB | 0.6118 | 207.035 | 4.0542 | 49.42 |

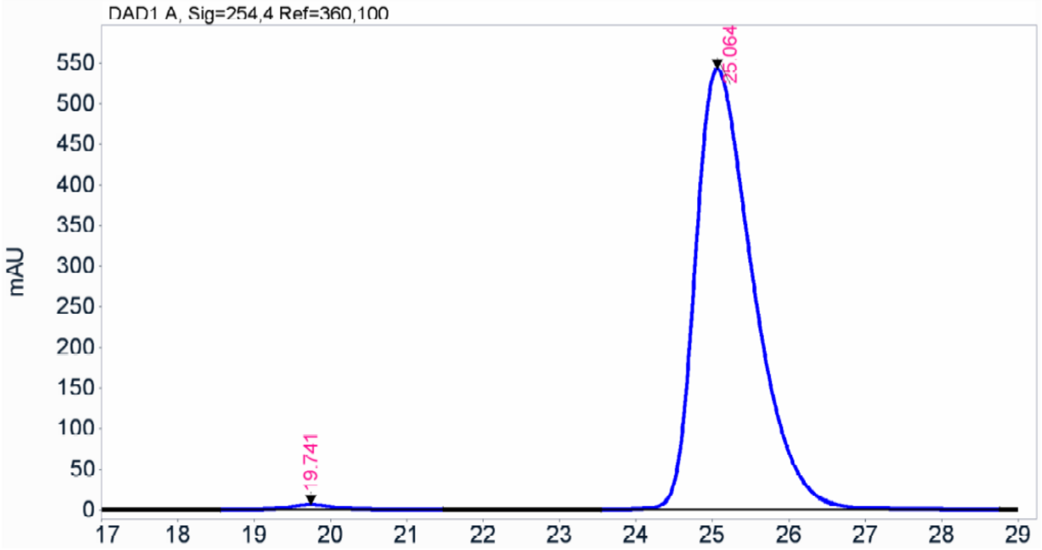


| **Signal:** | DAD1 A, Sig=254,4 Ref=360,100 | | | | |
|---|---|---|---|---|---|
| **RT [min]** | **Type** | **Width [min]** | **Area** | **Height** | **Area%** |
| 19.741 | BB | 0.6121 | 269.926 | 6.2725 | 0.94 |
| 25.064 | BB | 0.7989 | 28405.742 | 542.4609 | 99.06 |

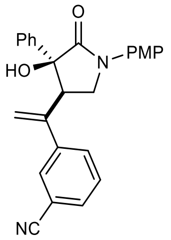


**2k**

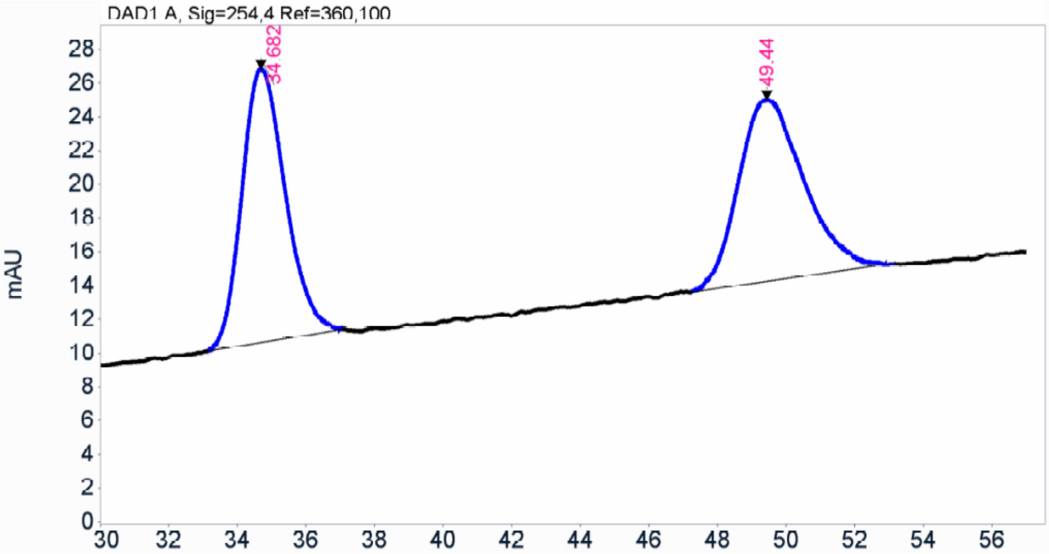


| **Signal:** | DAD1 A, Sig=254,4 Ref=360,100 | | | | |
|---|---|---|---|---|---|
| **RT [min]** | **Type** | **Width [min]** | **Area** | **Height** | **Area%** |
| 34.682 | BB | 1.0932 | 1425.551 | 16.1906 | 50.07 |
| 49.440 | MM | 1.5591 | 1421.671 | 10.7336 | 49.93 |

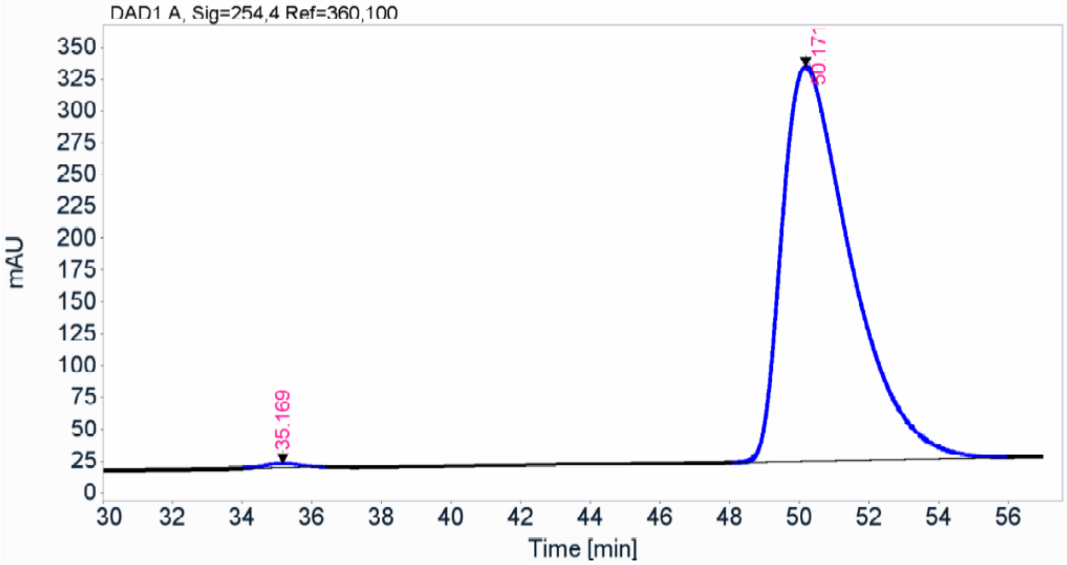


| **Signal:** | DAD1 A, Sig=254,4 Ref=360,100 | | | | |
|---|---|---|---|---|---|
| **RT [min]** | **Type** | **Width [min]** | **Area** | **Height** | **Area%** |
| 35.169 | MM | 0.8906 | 246.686 | 3.3919 | 0.58 |
| 50.171 | BV | 2.0299 | 42359.777 | 309.2750 | 99.42 |

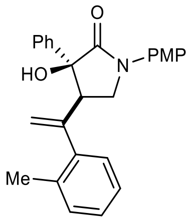


**2l**

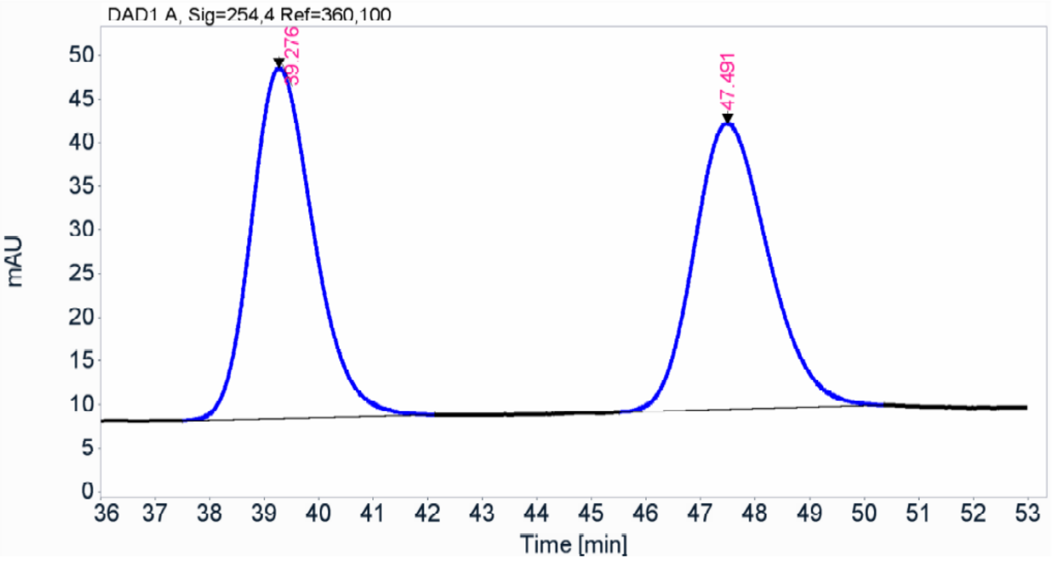


**Signal:** DAD1 A, Sig=254,4 Ref=360,100

| RT [min] | Type | Width [min] | Area | Height | Area% |
|---|---|---|---|---|---|
| 39.276 | BB | 1.1717 | 3183.034 | 40.1155 | 50.62 |
| 47.491 | BB | 1.3588 | 3104.945 | 32.7299 | 49.38 |

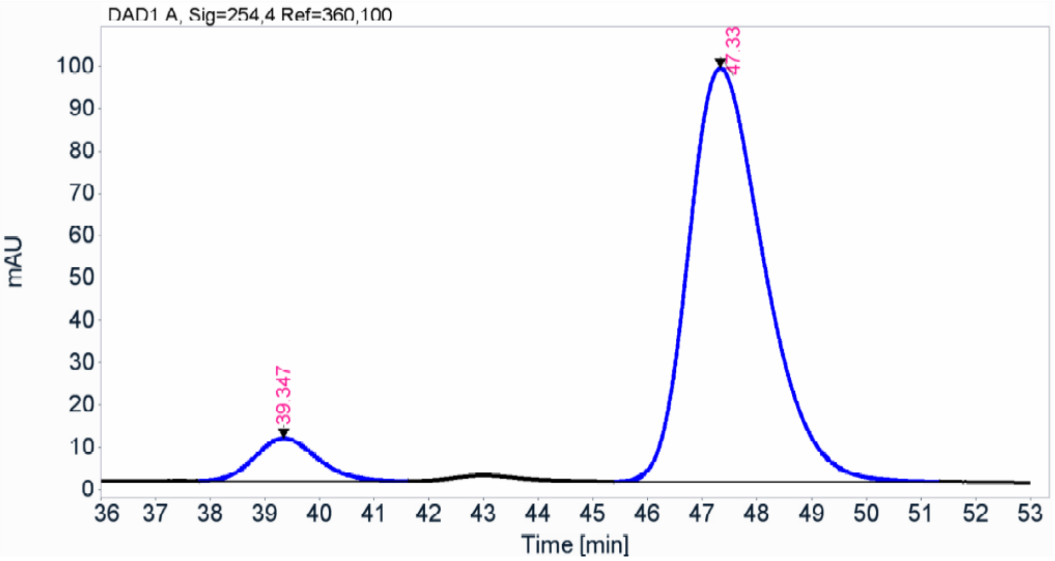


**Signal:** DAD1 A, Sig=254,4 Ref=360,100

| RT [min] | Type | Width [min] | Area | Height | Area% |
|---|---|---|---|---|---|
| 39.347 | BB | 0.9473 | 800.232 | 10.0459 | 7.86 |
| 47.330 | BB | 1.4280 | 9376.770 | 97.6720 | 92.14 |

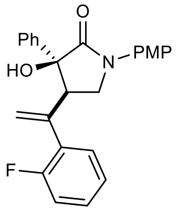


**2m**

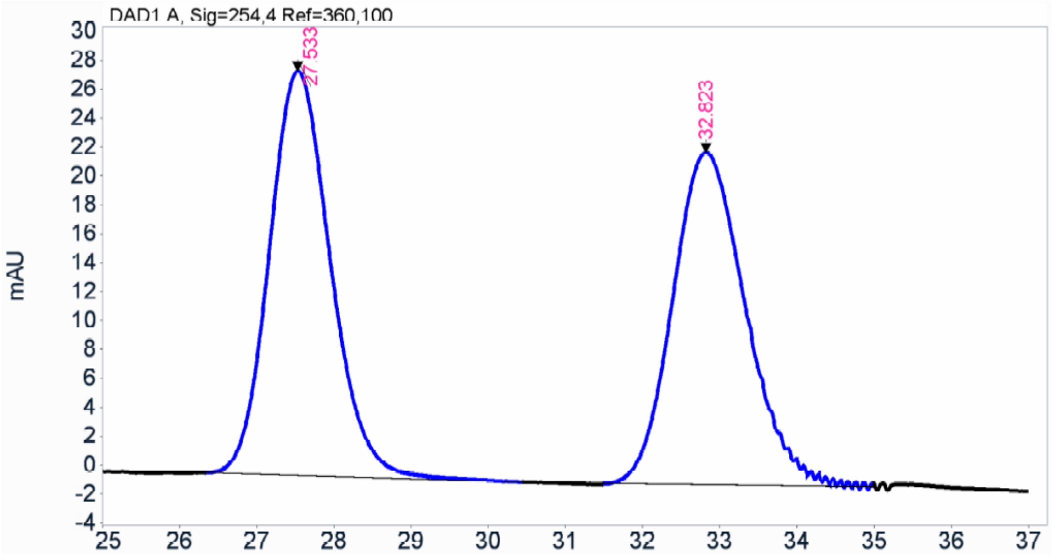


**Signal:** DAD1 A, Sig=254,4 Ref=360,100

| RT [min] | Type | Width [min] | Area | Height | Area% |
|---|---|---|---|---|---|
| 27.533 | BB | 0.8345 | 1540.565 | 27.9721 | 50.60 |
| 32.823 | MM | 1.0928 | 1504.198 | 22.9419 | 49.40 |

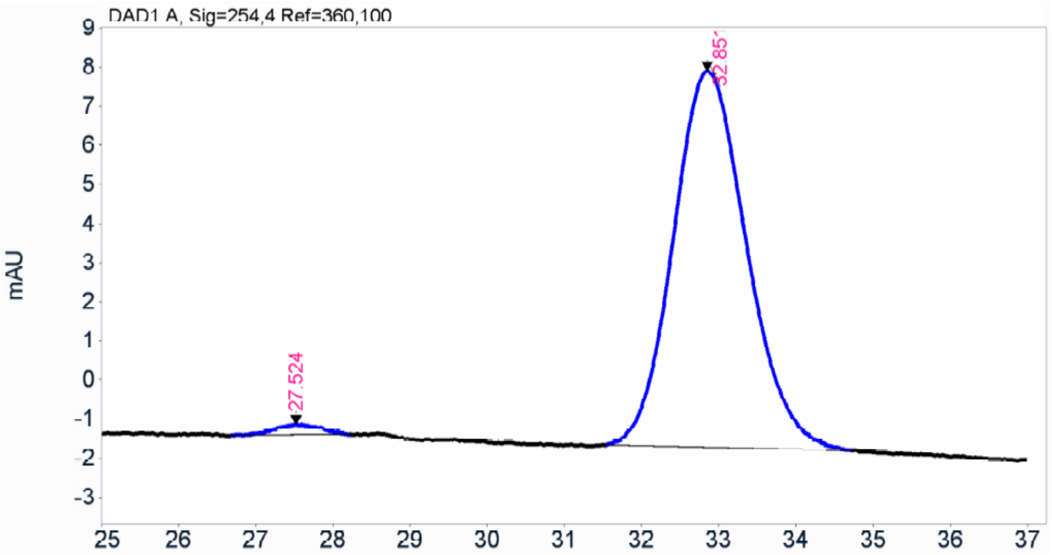


**Signal:** DAD1 A, Sig=254,4 Ref=360,100

| RT [min] | Type | Width [min] | Area | Height | Area% |
|---|---|---|---|---|---|
| 27.524 | MM | 0.6787 | 10.932 | 0.2685 | 1.69 |
| 32.851 | BB | 0.8387 | 635.358 | 9.6184 | 98.31 |

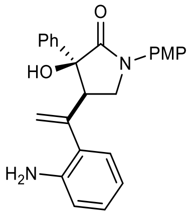

**2n**

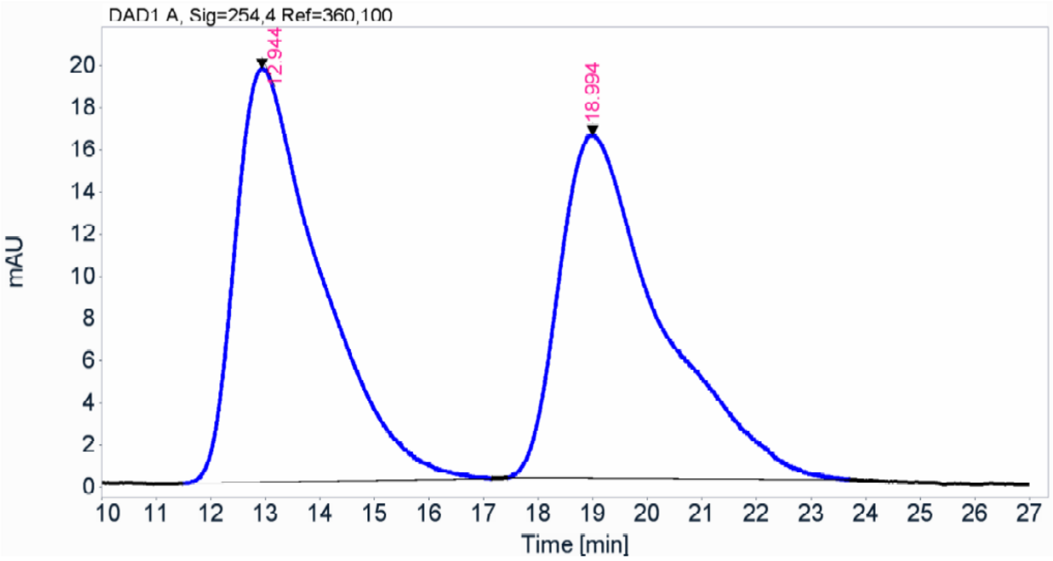

| Signal: | DAD1 A, Sig=254,4 Ref=360,100 | | | | |
|---|---|---|---|---|---|
| RT [min] | Type | Width [min] | Area | Height | Area% |
| 12.944 | BB | 1.4430 | 2120.079 | 19.6151 | 50.37 |
| 18.994 | BB | 1.5348 | 2089.147 | 16.2405 | 49.63 |

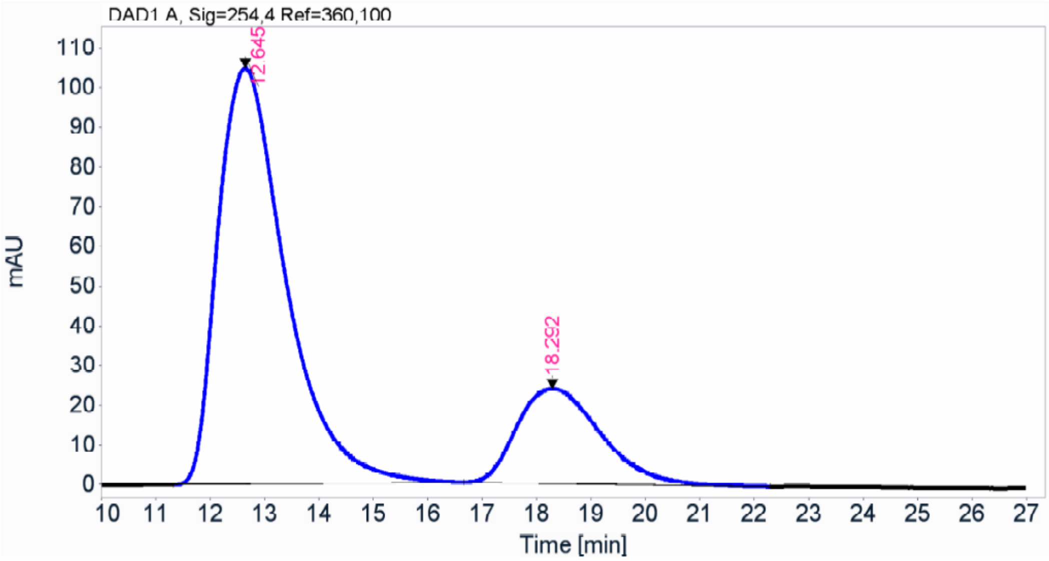

| Signal: | DAD1 A, Sig=254,4 Ref=360,100 | | | | |
|---|---|---|---|---|---|
| RT [min] | Type | Width [min] | Area | Height | Area% |
| 12.645 | BB | 1.3214 | 9063.034 | 104.7481 | 78.01 |
| 18.292 | BB | 1.4680 | 2555.103 | 23.9236 | 21.99 |

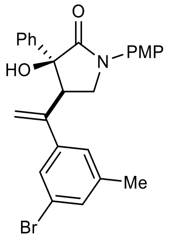

**2o**

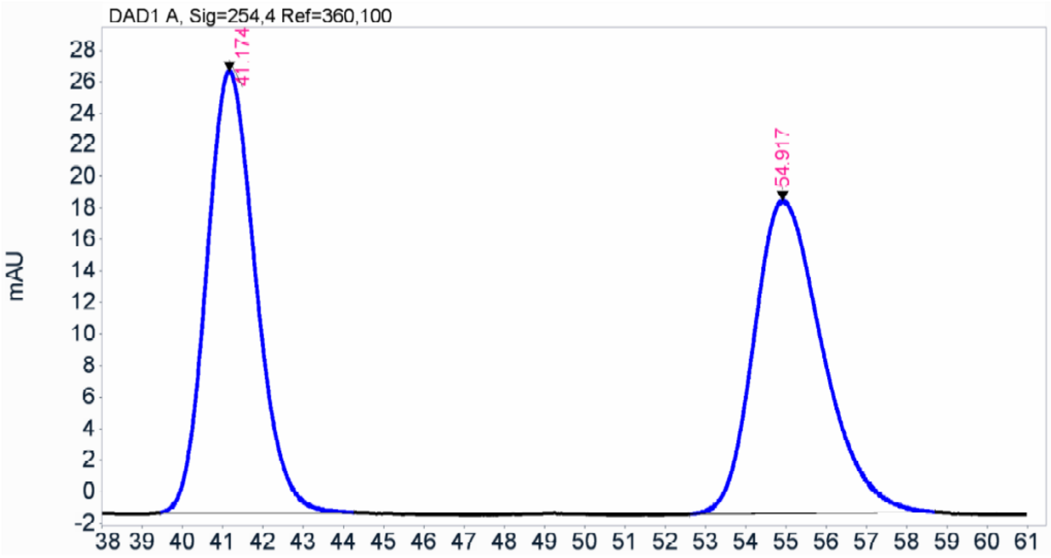


| Signal: | DAD1 A, Sig=254,4 Ref=360,100 | | | | |
|---|---|---|---|---|---|
| RT [min] | Type | Width [min] | Area | Height | Area% |
| 41.174 | BB | 1.1917 | 2359.033 | 27.9776 | 50.17 |
| 54.917 | BB | 1.4701 | 2343.136 | 19.8078 | 49.83 |

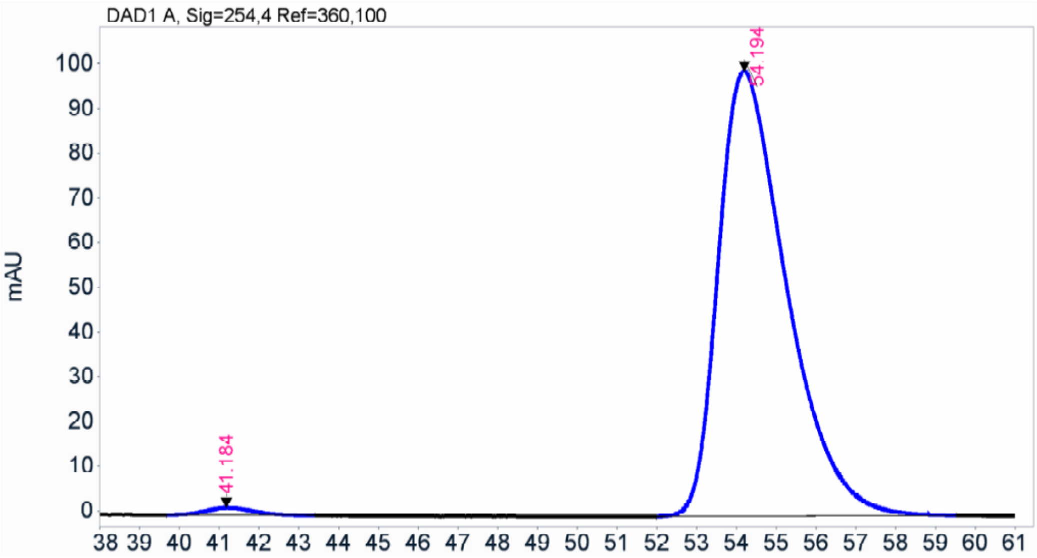


| Signal: | DAD1 A, Sig=254,4 Ref=360,100 | | | | |
|---|---|---|---|---|---|
| RT [min] | Type | Width [min] | Area | Height | Area% |
| 41.184 | MM | 1.3828 | 134.670 | 1.6232 | 1.13 |
| 54.194 | BB | 1.6949 | 11835.738 | 99.5656 | 98.87 |

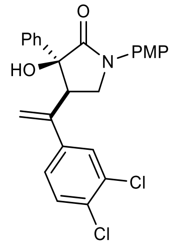


**2p**

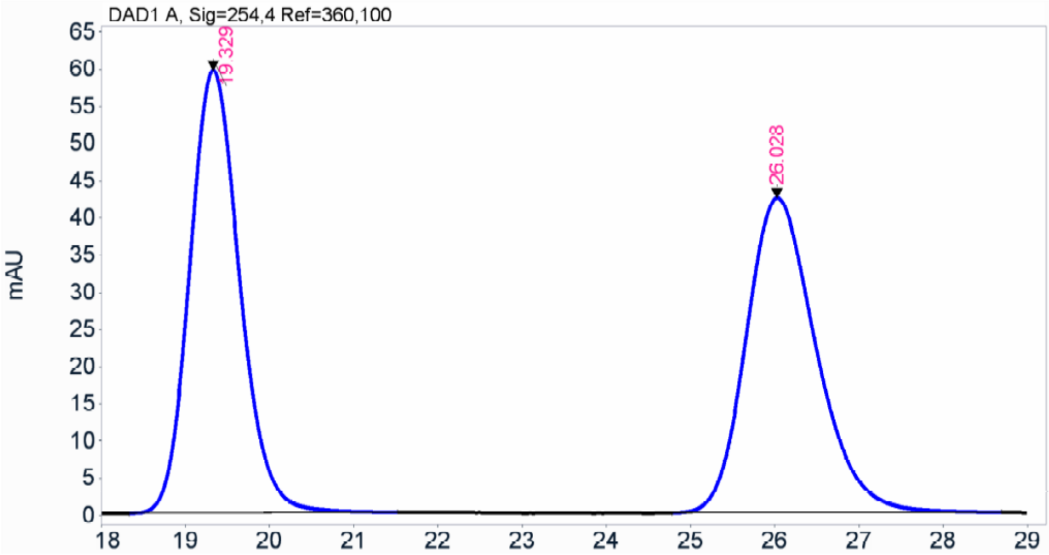


| **Signal:** | DAD1 A, Sig=254,4 Ref=360,100 | | | | |
|---|---|---|---|---|---|
| **RT [min]** | **Type** | **Width [min]** | **Area** | **Height** | **Area%** |
| 19.329 | BB | 0.6405 | 2451.615 | 59.5295 | 50.08 |
| 26.028 | BB | 0.8895 | 2444.082 | 42.3390 | 49.92 |

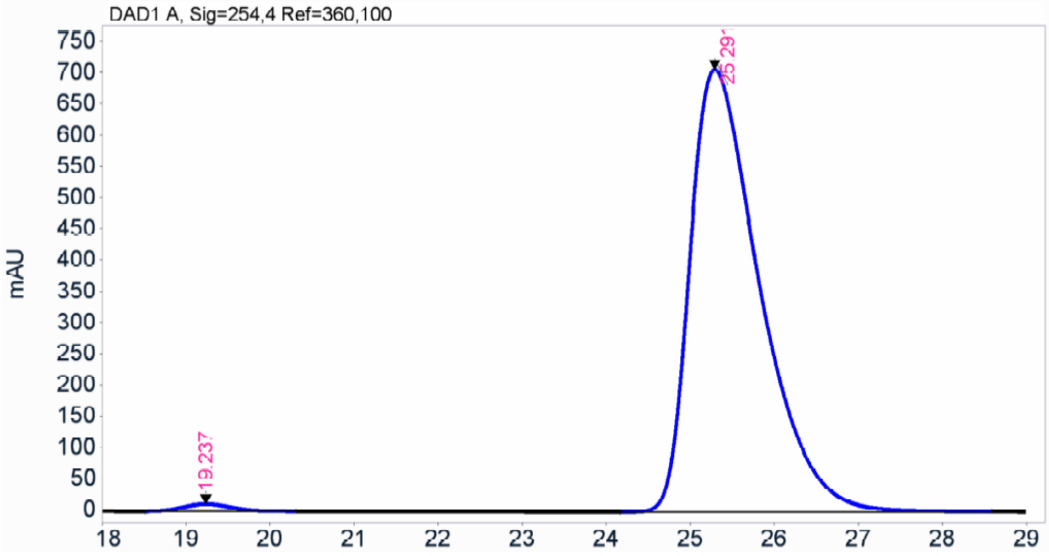


| **Signal:** | DAD1 A, Sig=254,4 Ref=360,100 | | | | |
|---|---|---|---|---|---|
| **RT [min]** | **Type** | **Width [min]** | **Area** | **Height** | **Area%** |
| 19.237 | MM | 0.6670 | 468.863 | 11.7162 | 1.14 |
| 25.291 | MM | 0.9564 | 40631.875 | 708.0849 | 98.86 |

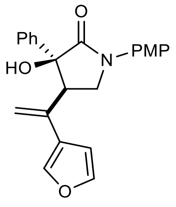


**2q**

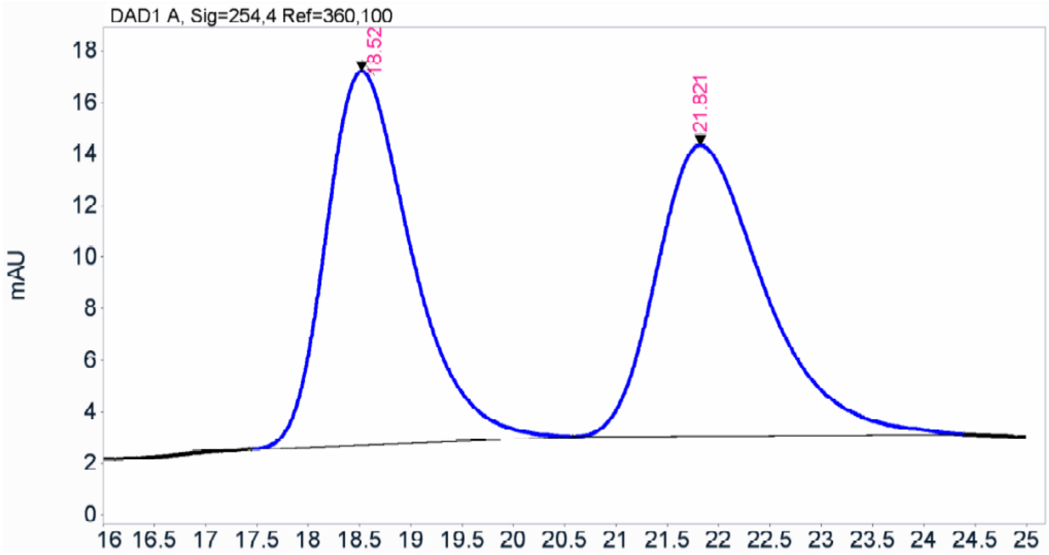


| Signal: | DAD1 A, Sig=254,4 Ref=360,100 | | | | |
|---|---|---|---|---|---|
| RT [min] | Type | Width [min] | Area | Height | Area% |
| 18.520 | BB | 0.8359 | 837.584 | 14.4996 | 50.36 |
| 21.821 | BB | 0.9937 | 825.528 | 11.2812 | 49.64 |

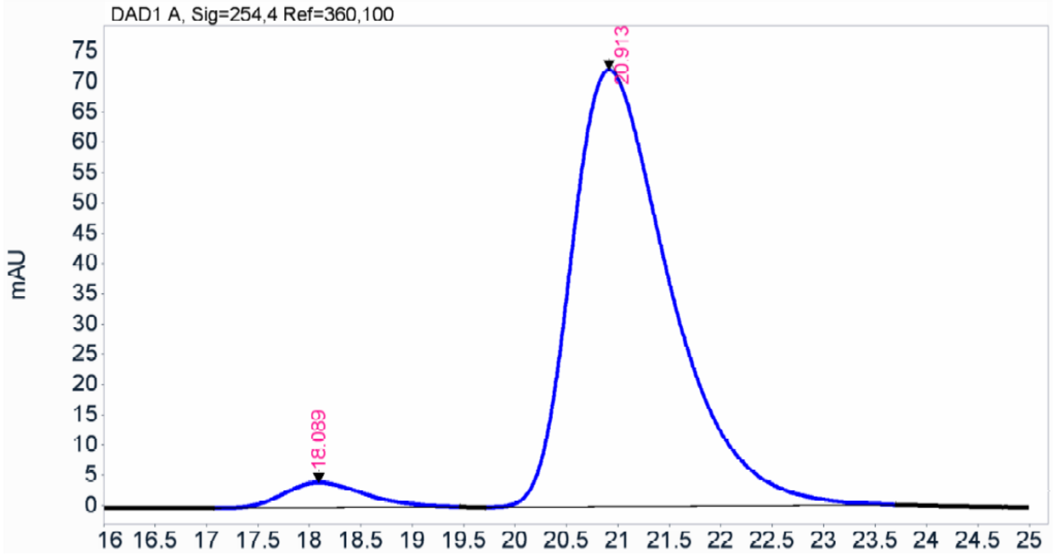


| Signal: | DAD1 A, Sig=254,4 Ref=360,100 | | | | |
|---|---|---|---|---|---|
| RT [min] | Type | Width [min] | Area | Height | Area% |
| 18.089 | MM | 0.9076 | 222.466 | 4.0851 | 4.44 |
| 20.913 | BB | 1.0110 | 4790.959 | 72.1197 | 95.56 |

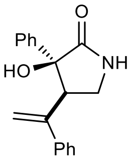


**2r**

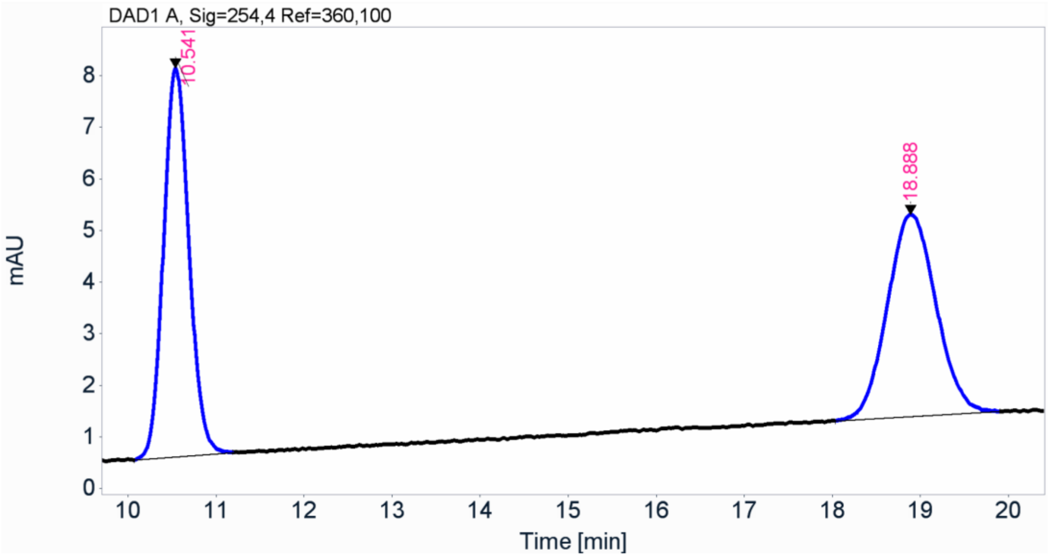


| Signal: | DAD1 A, Sig=254,4 Ref=360,100 | | | | |
|---|---|---|---|---|---|
| RT [min] | Type | Width [min] | Area | Height | Area% |
| 10.541 | BB | 0.3147 | 151.400 | 7.5259 | 50.11 |
| 18.888 | BB | 0.5333 | 150.721 | 3.9134 | 49.89 |

Arylative cyclization to produce **2r**:

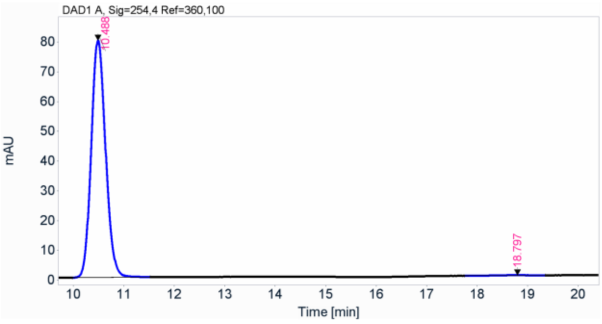


| Signal: | DAD1 A, Sig=254,4 Ref=360,100 | | | | |
|---|---|---|---|---|---|
| RT [min] | Type | Width [min] | Area | Height | Area% |
| 10.488 | BB | 0.3062 | 1570.278 | 79.5666 | 99.63 |
| 18.797 | MM | 0.7085 | 5.818 | 0.1369 | 0.37 |

Deprotection of **2a** (96% ee) to produce **2r**:

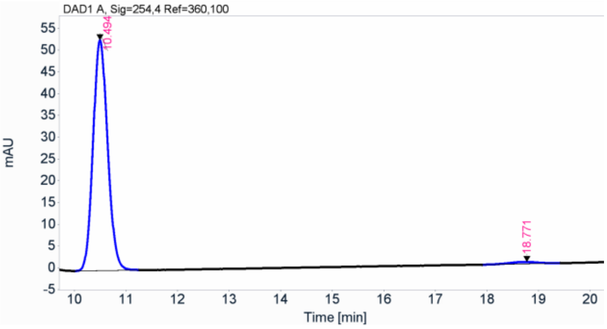


| Signal: | DAD1 A, Sig=254,4 Ref=360,100 | | | | |
|---|---|---|---|---|---|
| RT [min] | Type | Width [min] | Area | Height | Area% |
| 10.494 | BB | 0.3074 | 1040.981 | 52.9311 | 98.24 |
| 18.771 | MM | 0.6890 | 18.649 | 0.4511 | 1.76 |

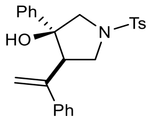

**5a**

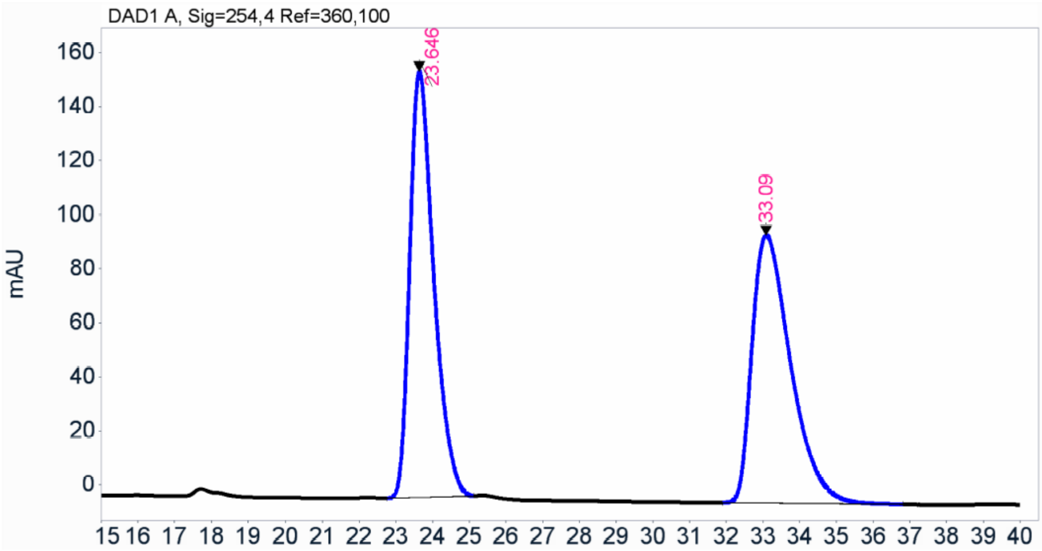


Signal: DAD1 A, Sig=254,4 Ref=360,100

| RT [min] | Type | Width [min] | Area | Height | Area% |
|---|---|---|---|---|---|
| 23.646 | BB | 0.6878 | 7175.295 | 157.9983 | 49.66 |
| 33.090 | BB | 1.1129 | 7272.276 | 99.2466 | 50.34 |

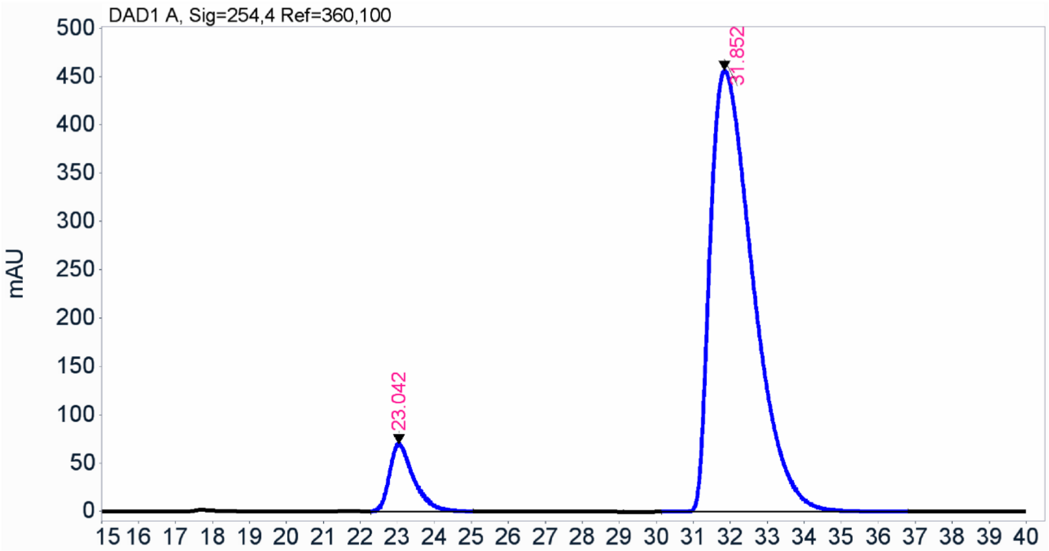


Signal: DAD1 A, Sig=254,4 Ref=360,100

| RT [min] | Type | Width [min] | Area | Height | Area% |
|---|---|---|---|---|---|
| 23.042 | MM | 0.7648 | 3200.952 | 69.7592 | 8.04 |
| 31.852 | MM | 1.3367 | 36632.145 | 456.7636 | 91.96 |

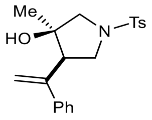


**5b**

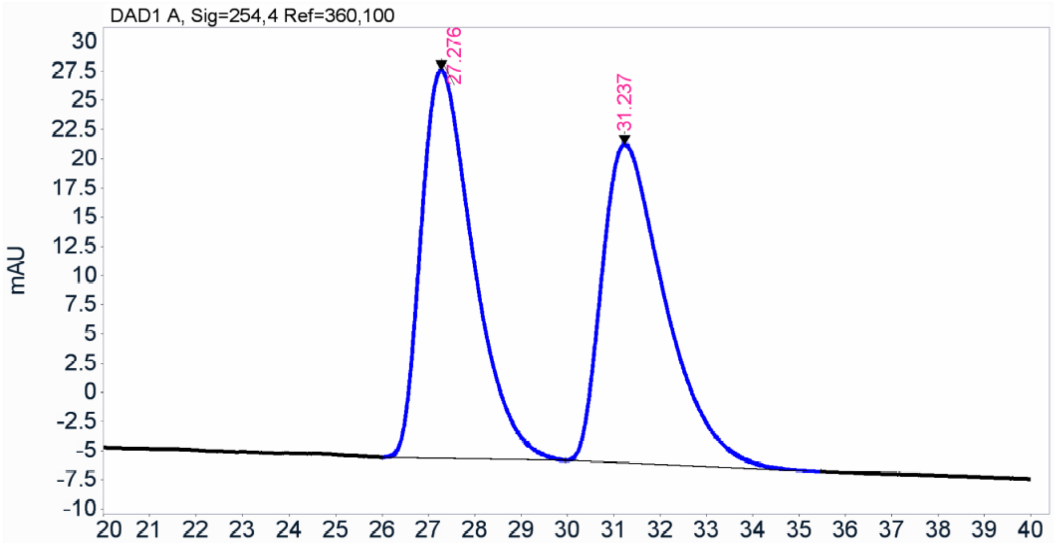


| Signal: | DAD1 A, Sig=254,4 Ref=360,100 | | | | |
|---|---|---|---|---|---|
| RT [min] | Type | Width [min] | Area | Height | Area% |
| 27.276 | BB | 1.1473 | 2596.034 | 33.2342 | 50.03 |
| 31.237 | BB | 1.3070 | 2593.351 | 27.2815 | 49.97 |

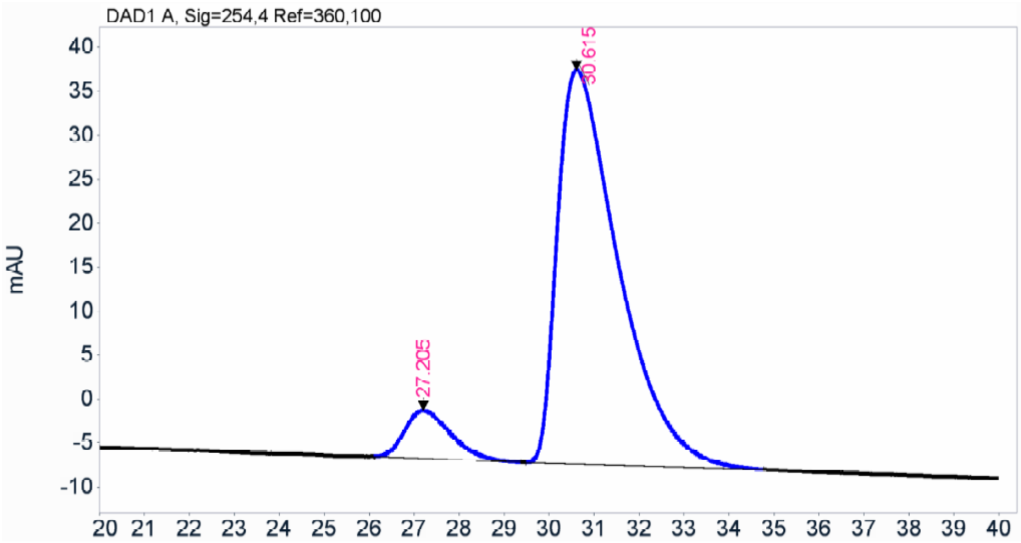


| Signal: | DAD1 A, Sig=254,4 Ref=360,100 | | | | |
|---|---|---|---|---|---|
| RT [min] | Type | Width [min] | Area | Height | Area% |
| 27.205 | BB | 0.8496 | 384.856 | 5.4519 | 8.34 |
| 30.615 | BB | 1.3208 | 4231.603 | 44.7677 | 91.66 |

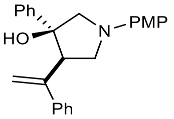


**5c**

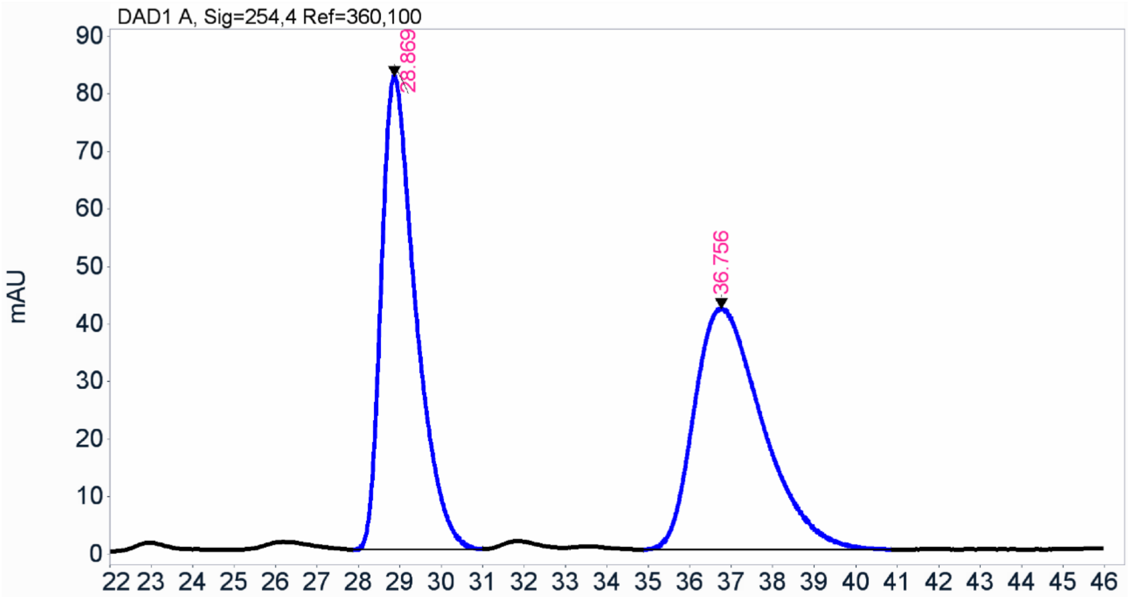


| **Signal:** | DAD1 A, Sig=254,4 Ref=360,100 | | | | |
|---|---|---|---|---|---|
| **RT [min]** | **Type** | **Width [min]** | **Area** | **Height** | **Area%** |
| 28.869 | BB | 0.8571 | 4739.494 | 82.1530 | 49.67 |
| 36.756 | BB | 1.6185 | 4802.273 | 41.7956 | 50.33 |

Reaction carried out in TFE:

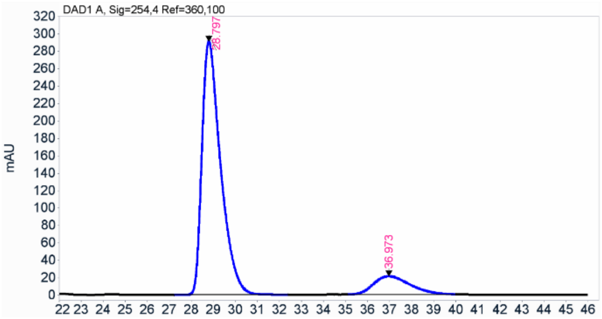


| **Signal:** | DAD1 A, Sig=254,4 Ref=360,100 | | | | |
|---|---|---|---|---|---|
| **RT [min]** | **Type** | **Width [min]** | **Area** | **Height** | **Area%** |
| 28.797 | MM | 0.9767 | 17031.924 | 290.6238 | 87.56 |
| 36.973 | MM | 1.9256 | 2419.486 | 20.9414 | 12.44 |

Reaction as carried out in MeCN:

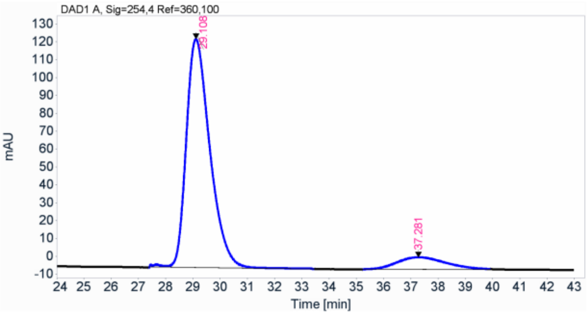


| **Signal:** | DAD1 A, Sig=254,4 Ref=360,100 | | | | |
|---|---|---|---|---|---|
| **RT [min]** | **Type** | **Width [min]** | **Area** | **Height** | **Area%** |
| 29.108 | MM | 1.0085 | 7737.827 | 127.8751 | 90.09 |
| 37.281 | MM | 2.0996 | 851.216 | 6.7571 | 9.91 |

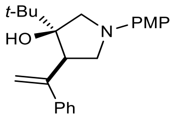


**5d**

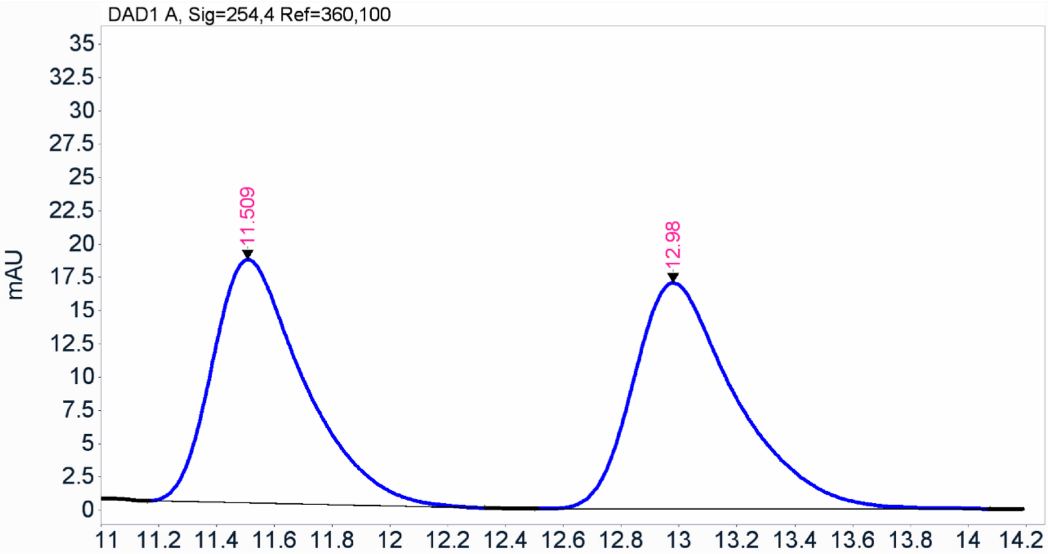


| **Signal:** | DAD1 A, Sig=254,4 Ref=360,100 | | | | |
|---|---|---|---|---|---|
| **RT [min]** | **Type** | **Width [min]** | **Area** | **Height** | **Area%** |
| 11.509 | BB | 0.3267 | 402.336 | 18.2870 | 49.06 |
| 12.980 | BB | 0.3631 | 417.676 | 16.9732 | 50.94 |

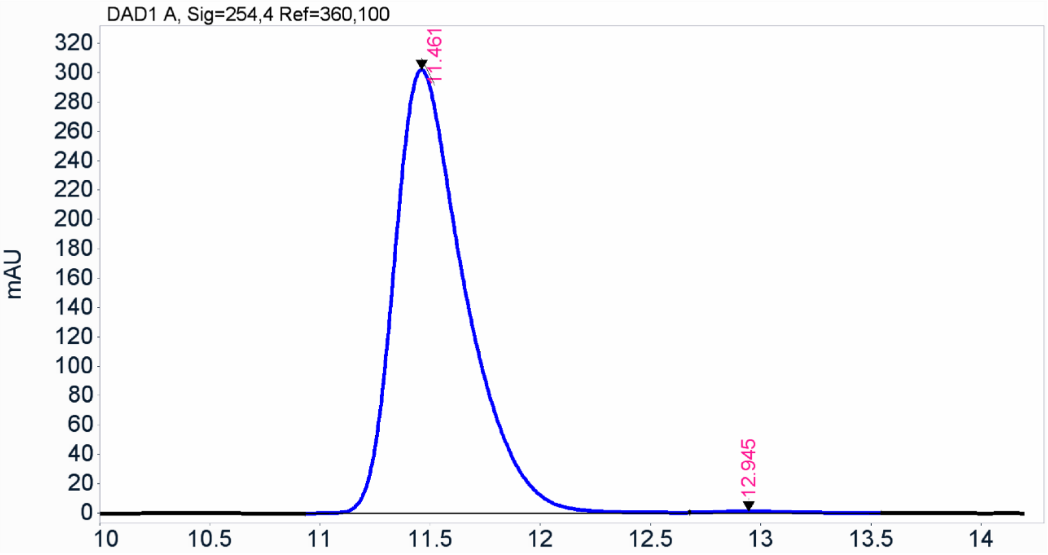


| **Signal:** | DAD1 A, Sig=254,4 Ref=360,100 | | | | |
|---|---|---|---|---|---|
| **RT [min]** | **Type** | **Width [min]** | **Area** | **Height** | **Area%** |
| 11.461 | MM | 0.3708 | 6724.485 | 302.2877 | 99.55 |
| 12.945 | MM | 0.4007 | 30.625 | 1.2740 | 0.45 |

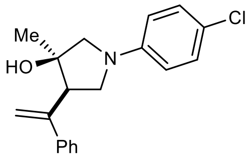


**5e**

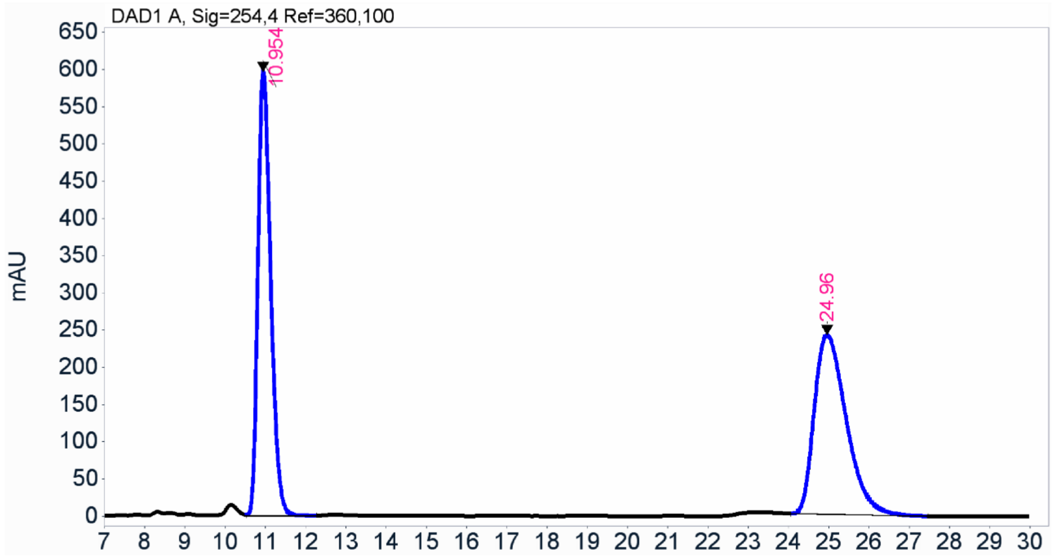


| Signal: | DAD1 A, Sig=254,4 Ref=360,100 | | | | |
|---|---|---|---|---|---|
| RT [min] | Type | Width [min] | Area | Height | Area% |
| 10.954 | VB | 0.3411 | 13139.221 | 596.1681 | 49.59 |
| 24.960 | BB | 0.8638 | 13356.048 | 241.2753 | 50.41 |

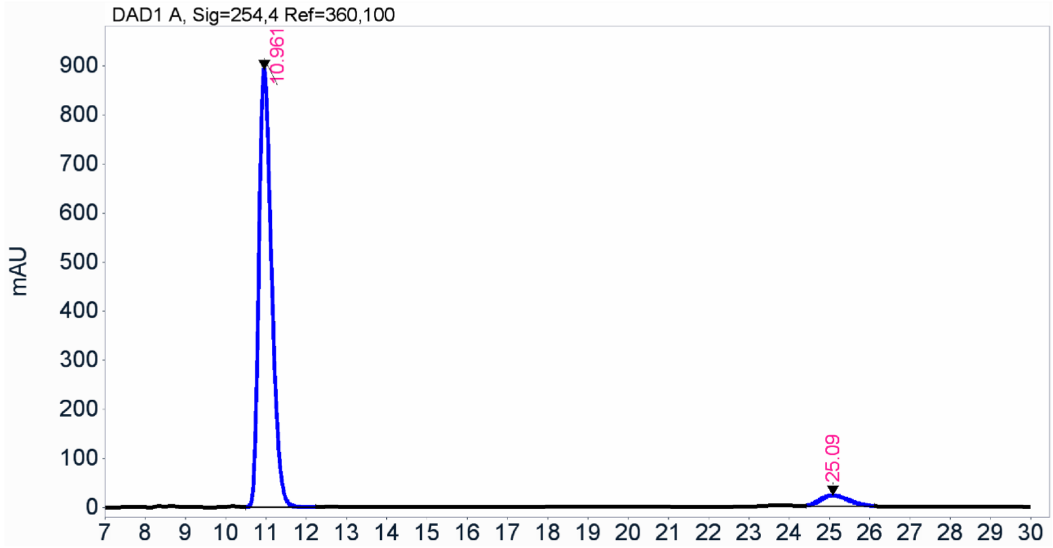


| Signal: | DAD1 A, Sig=254,4 Ref=360,100 | | | | |
|---|---|---|---|---|---|
| RT [min] | Type | Width [min] | Area | Height | Area% |
| 10.961 | VB | 0.3409 | 19644.066 | 892.2686 | 94.84 |
| 25.090 | MM | 0.8171 | 1069.034 | 21.8043 | 5.16 |

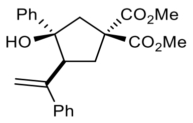


**5f**

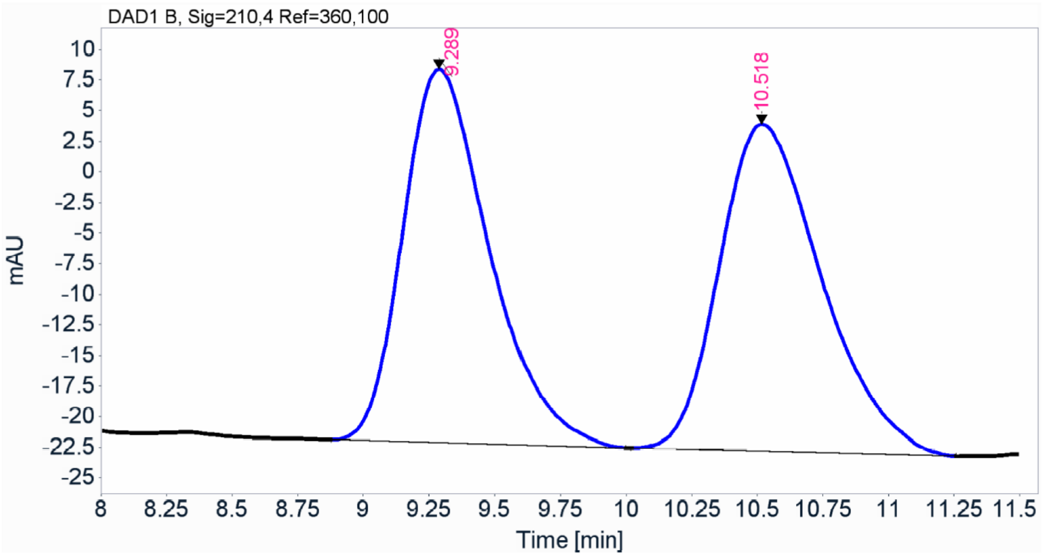


| **Signal:** | DAD1 B, Sig=210,4 Ref=360,100 | | | | |
|---|---|---|---|---|---|
| **RT [min]** | **Type** | **Width [min]** | **Area** | **Height** | **Area%** |
| 9.289 | BB | 0.3497 | 699.403 | 30.4857 | 49.20 |
| 10.518 | BB | 0.4158 | 722.033 | 26.6784 | 50.80 |

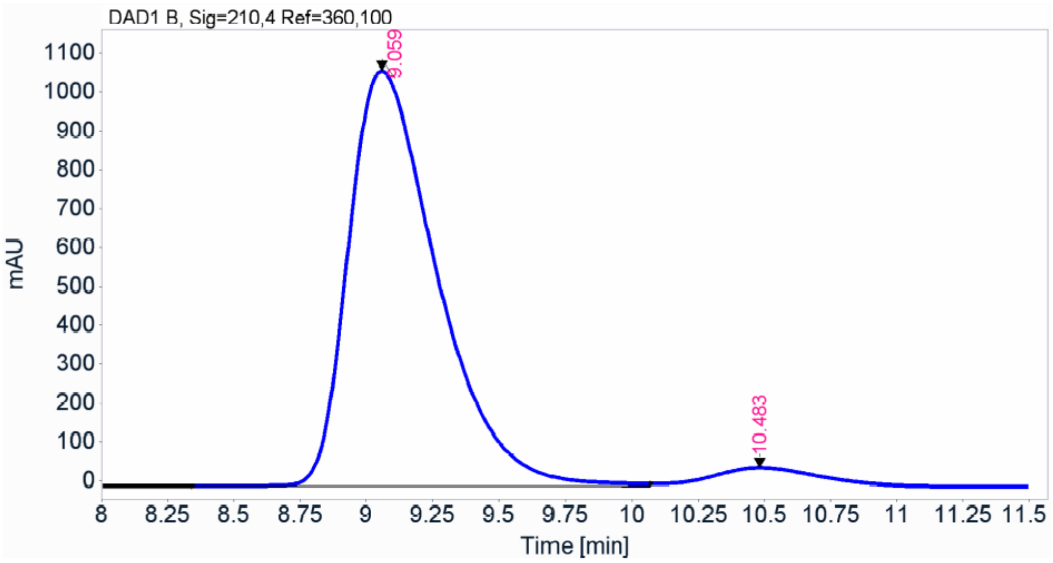


| **Signal:** | DAD1 B, Sig=210,4 Ref=360,100 | | | | |
|---|---|---|---|---|---|
| **RT [min]** | **Type** | **Width [min]** | **Area** | **Height** | **Area%** |
| 9.059 | BV | 0.3547 | 24784.941 | 1068.1560 | 94.89 |
| 10.483 | VB | 0.4321 | 1334.299 | 47.1622 | 5.11 |

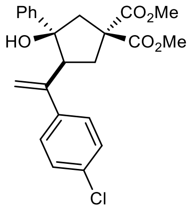


**5g**

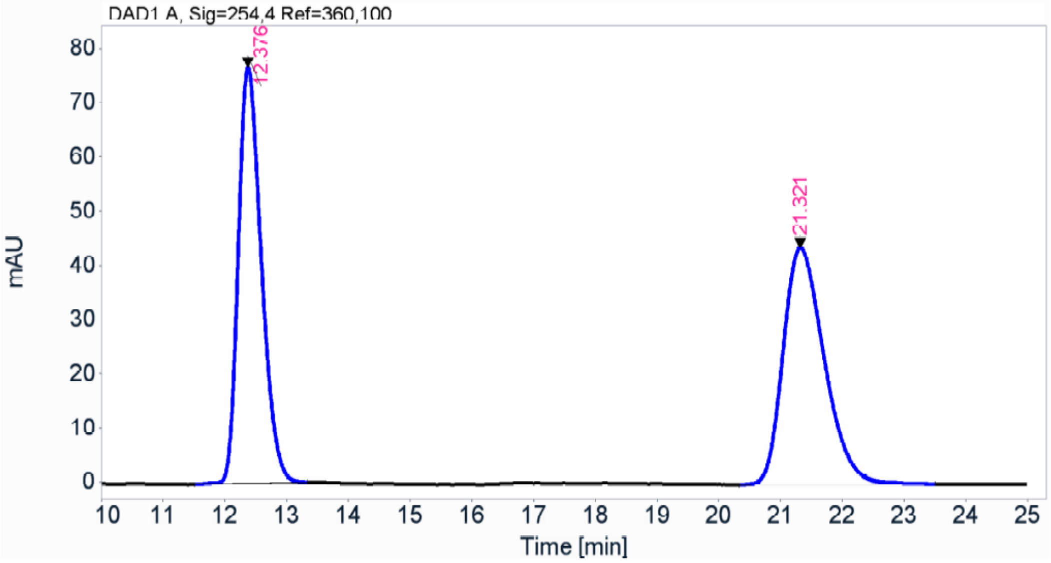


| Signal: | DAD1 A, Sig=254,4 Ref=360,100 | | | | |
|---|---|---|---|---|---|
| RT [min] | Type | Width [min] | Area | Height | Area% |
| 12.376 | BB | 0.3989 | 1991.814 | 76.7141 | 49.72 |
| 21.321 | BB | 0.7168 | 2013.975 | 43.6066 | 50.28 |

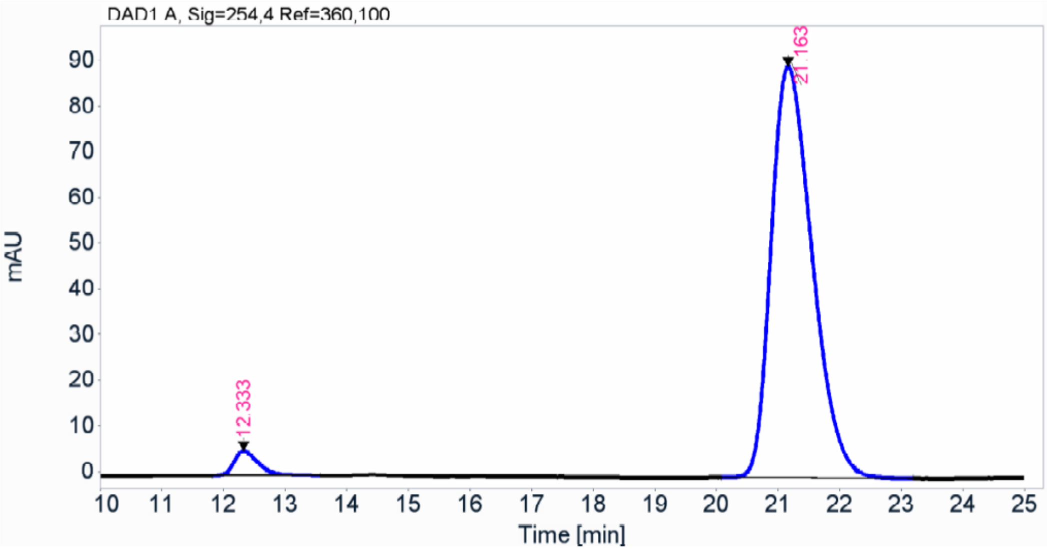


| Signal: | DAD1 A, Sig=254,4 Ref=360,100 | | | | |
|---|---|---|---|---|---|
| RT [min] | Type | Width [min] | Area | Height | Area% |
| 12.333 | BB | 0.4138 | 147.003 | 5.3973 | 3.41 |
| 21.163 | BB | 0.7238 | 4163.964 | 90.0011 | 96.59 |

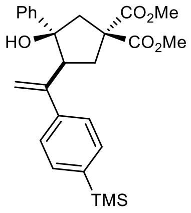


**5h**

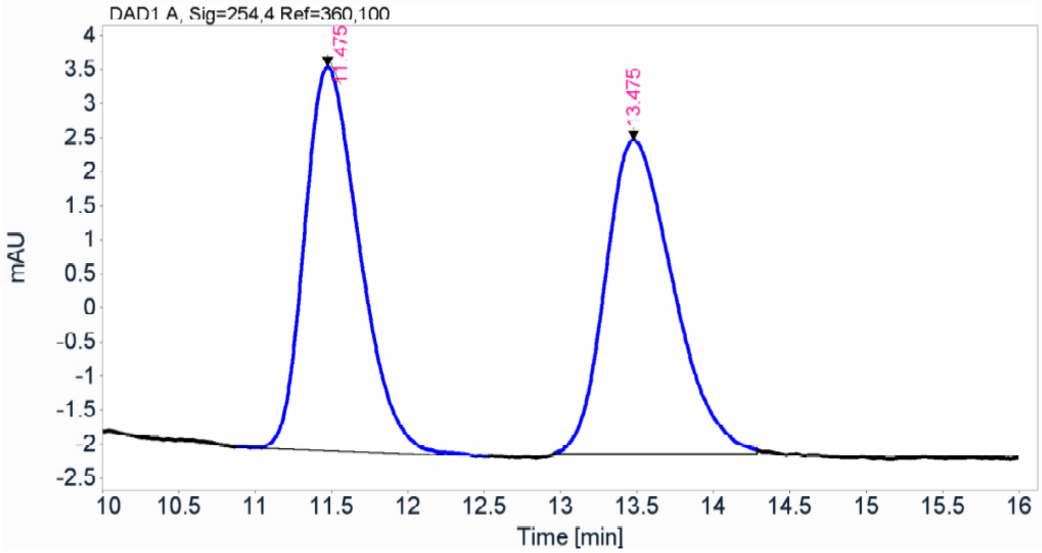


| Signal: | DAD1 A, Sig=254,4 Ref=360,100 | | | | |
|---|---|---|---|---|---|
| **RT [min]** | **Type** | **Width [min]** | **Area** | **Height** | **Area%** |
| 11.475 | MM | 0.4166 | 140.926 | 5.6384 | 49.91 |
| 13.475 | MM | 0.5123 | 141.455 | 4.6017 | 50.09 |

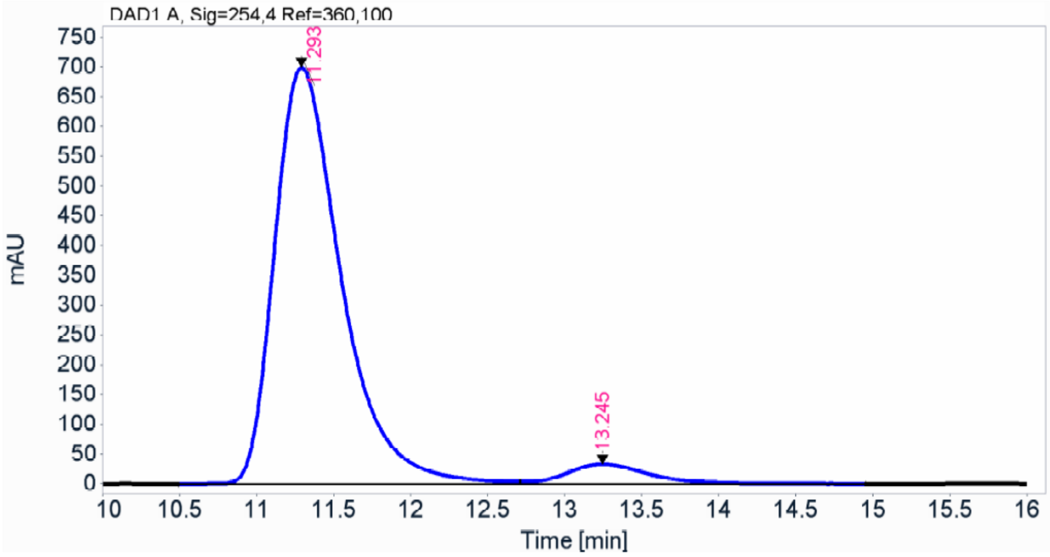


| Signal: | DAD1 A, Sig=254,4 Ref=360,100 | | | | |
|---|---|---|---|---|---|
| **RT [min]** | **Type** | **Width [min]** | **Area** | **Height** | **Area%** |
| 11.293 | BV | 0.4583 | 20997.943 | 699.3309 | 94.54 |
| 13.245 | VB | 0.5620 | 1212.658 | 32.9001 | 5.46 |

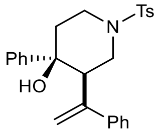

**5i**

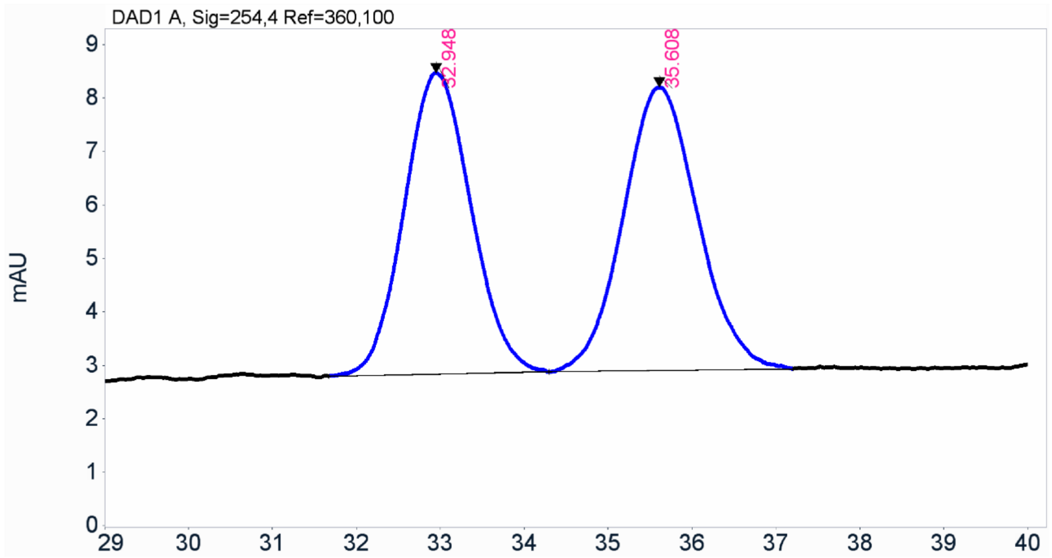


| Signal: | DAD1 A, Sig=254,4 Ref=360,100 | | | | |
|---|---|---|---|---|---|
| RT [min] | Type | Width [min] | Area | Height | Area% |
| 32.948 | BB | 0.7080 | 311.942 | 5.6333 | 48.83 |
| 35.608 | BB | 0.7360 | 326.890 | 5.2966 | 51.17 |

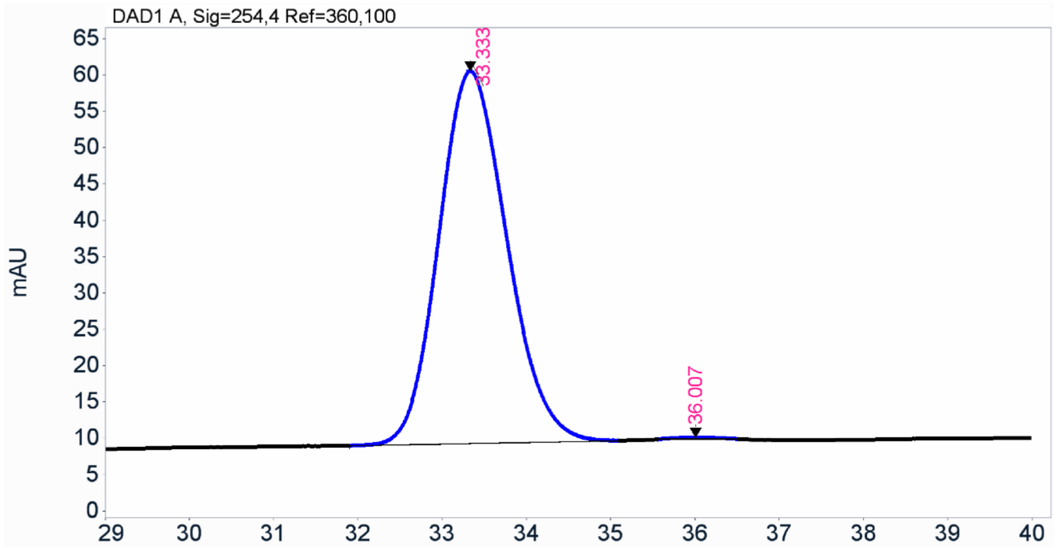


| Signal: | DAD1 A, Sig=254,4 Ref=360,100 | | | | |
|---|---|---|---|---|---|
| RT [min] | Type | Width [min] | Area | Height | Area% |
| 33.333 | BB | 0.8548 | 2876.049 | 51.2456 | 99.64 |
| 36.007 | MM | 0.6157 | 10.267 | 0.2779 | 0.36 |

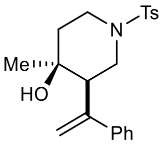


**5j**

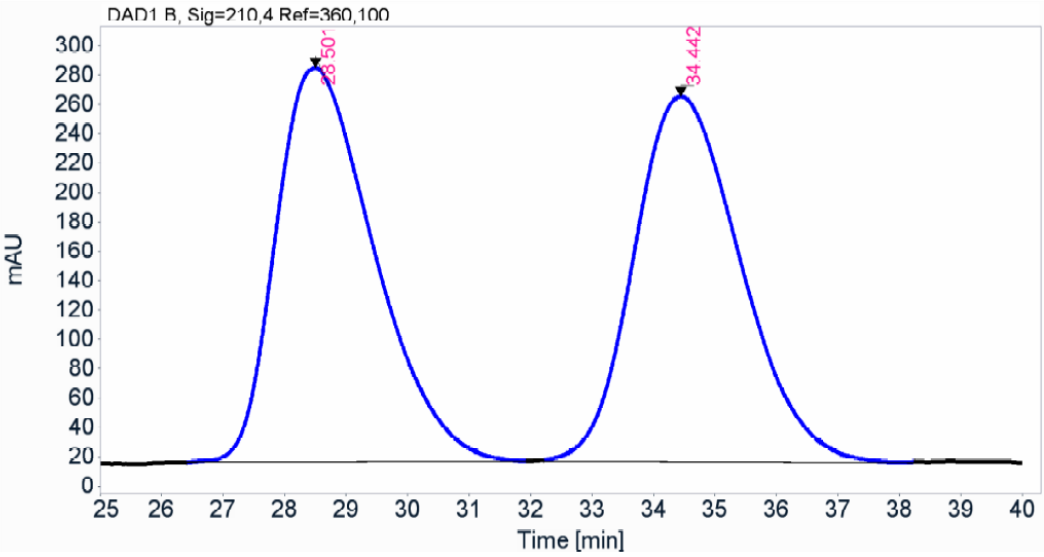


| Signal: | DAD1 B, Sig=210,4 Ref=360,100 | | | | |
|---|---|---|---|---|---|
| RT [min] | Type | Width [min] | Area | Height | Area% |
| 28.501 | BB | 1.6572 | 29771.641 | 268.5816 | 49.83 |
| 34.442 | MM | 2.0086 | 29977.330 | 248.7364 | 50.17 |

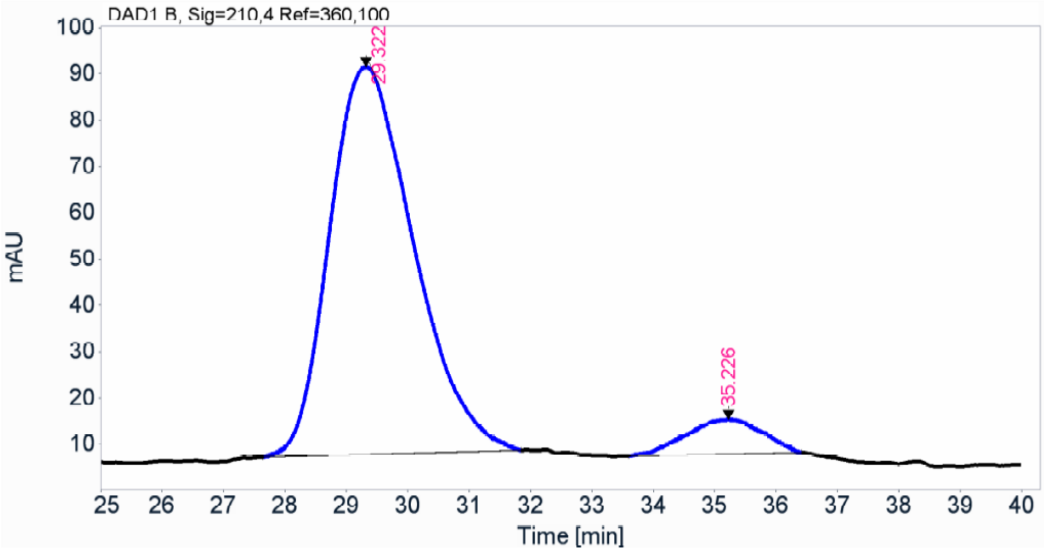


| Signal: | DAD1 B, Sig=210,4 Ref=360,100 | | | | |
|---|---|---|---|---|---|
| RT [min] | Type | Width [min] | Area | Height | Area% |
| 29.322 | BB | 1.3479 | 7956.773 | 83.6343 | 92.45 |
| 35.226 | BB | 1.0280 | 649.418 | 7.4721 | 7.55 |

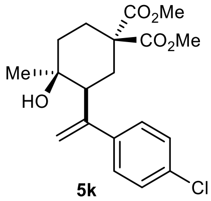


**5k**

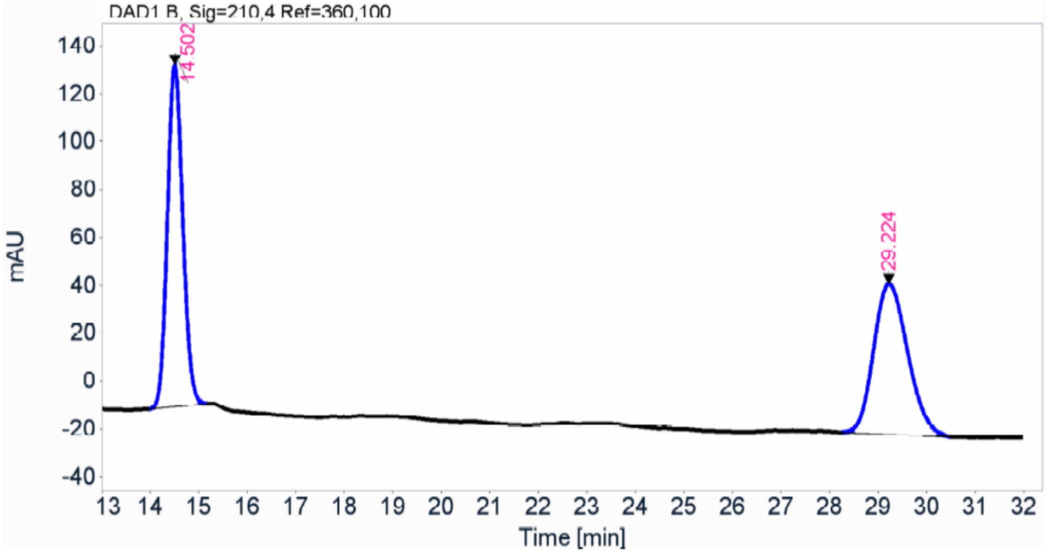


| **Signal:** | DAD1 B, Sig=210,4 Ref=360,100 | | | | |
|---|---|---|---|---|---|
| **RT [min]** | **Type** | **Width [min]** | **Area** | **Height** | **Area%** |
| 14.502 | BB | 0.3436 | 3152.104 | 142.7668 | 50.65 |
| 29.224 | BB | 0.7574 | 3071.185 | 62.9227 | 49.35 |

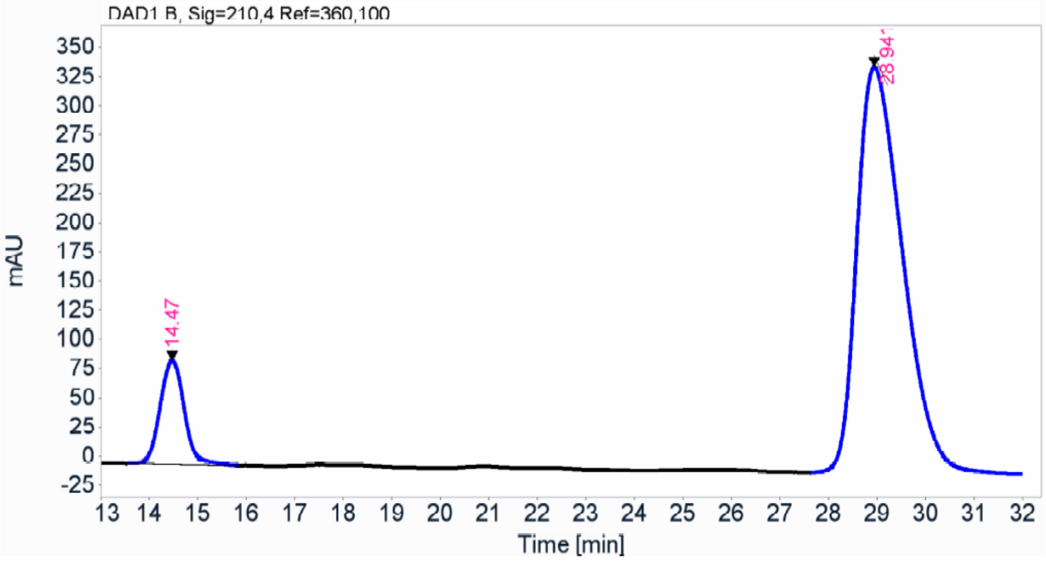


| **Signal:** | DAD1 B, Sig=210,4 Ref=360,100 | | | | |
|---|---|---|---|---|---|
| **RT [min]** | **Type** | **Width [min]** | **Area** | **Height** | **Area%** |
| 14.470 | BB | 0.5478 | 3065.188 | 88.5627 | 11.92 |
| 28.941 | BB | 1.0045 | 22649.174 | 347.4564 | 88.08 |

**References**


1. Prepared according to: N. Sakai, H. Hori, Y. Ogiwara, *Eur. J. Org. Chem.* **2015**, 1905-1909.
2. Prepared according to: M. W. Wright, T. L. Smalley, M. E. Welker, A. L. Rheingold, *J. Am. Chem. Soc.* **1994**, *116*, 6777-6791.
3. Prepared according to: W. Hess, J. W. Burton, *Chem. Eur. J.* **2010**, *16*, 12303-12306.
4. C.-M. Yu, Y.-T. Hong, J.-H. Lee, *J. Org. Chem.* **2004**, *69*, 8506-8509.
5. Prepared according to: Y. H. Shin, M. Maheswara, J. Y. Hwang, E. J. Kang, *Eur. J. Org. Chem.* **2014**, 2305-2311.
6. H. Tsukamoto, T. Matsumoto, Y. Kondo, *Org. Lett.* **2008**, *10*, 1047-1050.
7. Prepared according to: D. W. Low, G. Pattison, M. D. Wieczysty, G. H. Churchill, H. W. Lam, *Org. Lett.* **2012**, *14*, 2548-2551.
8. Prepared according to: F. Cambeiro, S. López, J. A. Varela, C. Saá, *Angew. Chem., Int. Ed.* **2014**, *53*, 5959-5963.
9. Prepared according to: A. G. Campaña, N. Fuentes, E. Gómez-Bengoa, C. Mateo, J. E. Oltra, A. M. Echavarren, J. M. Cuerva, *J. Org. Chem.* **2007**, *72*, 8127-8130.
10. Prepared according to: S. Kamijo, G. B. Dudley, *J. Am. Chem. Soc.* **2006**, *128*, 6499-6507.
